# Sex differences in plasma endocannabinoids and related lipids before and after single and repeated mTBI: an exploratory study of endolipid plasma biomarkers

**DOI:** 10.3389/fnmol.2026.1707732

**Published:** 2026-06-18

**Authors:** Emily Richter, Taylor J. Woodward, Praveen P. Kulkarni, Craig F. Ferris, Heather B. Bradshaw

**Affiliations:** 1 Department of Psychological and Brain Sciences, Indiana University, Bloomington, IN, United States; 2 Center for Translational Neuroimaging, Northeastern University, Boston, MA, United States; 3 Departments of Psychology and Pharmaceutical Sciences, Northeastern University, Boston, MA, United States

**Keywords:** endocannabinod system, lipidomics, mTBI (mild traumatic brain injury), plasma, sex differences

## Abstract

Sex differences in mild traumatic brain injury (mTBI) symptoms and severity are documented in both initial clinical presentation and long-term outcomes. Convergent data have demonstrated that the long-term outcomes for female patients with mTBI are worse overall than those in male patients. Somewhat paradoxically, female patients are less likely to be admitted to the intensive care unit (ICU) and to have significantly shorter stays when admitted. Taken together, these data have suggested that female patients may appear to present symptoms that are considered less life-threatening after the initial injury than male patients; however, this lack of symptoms may contribute to their poorer long-term outcomes. Previous studies have identified protein-derived plasma biomarkers for mTBI; however, the effects of mTBI on lipid signaling molecules and metabolites in blood are largely unknown. Endogenous lipids (endolipids) such as the endocannabinoids (eCBs) and their congeners are endolipids with a range of signaling properties that are associated with promoting neuroprotective responses after mTBI in animal models. Previously, we demonstrated sex-dependent changes in neuroinflammation after repeated head injury, in which female patients had increased levels of neuroinflammatory markers. In this study, we examined genetic sex and timing effects on the plasma lipidome using a rat model of single and repeated mTBI. This exploratory lipidomics screen showed that a single head injury drives significantly more changes in plasma endolipids in male rats (32 %) than female rats (8%), whereas an additional head injury on the following day showed only 11% change in male rats but 15% in female rats. Key endolipids upregulated in male rats after the single-hit injury are precursors for resolvin molecules (eicosapentaenoic acid and docosapentaenoic acid), and this was absent in female rats. Given that this repeated injury mTBI model in rats showed greater longitudinal CNS damage in female rats, we hypothesize that the increases in endolipids in male rats but not female rats on the first day of injury are protective.

## Introduction

Sex differences in mild traumatic brain injury (mTBI) symptoms and severity are documented in both initial clinical presentation and long-term outcomes (Bazarian et al., 2010; Dujardin et al., 2024; Beijer et al., 2023; Peterson et al., 2022; Starkey et al., 2022; Mikolic et al., 2021). Convergent data demonstrate that the long-term outcomes for female patients with mTBI are worse overall than those for male patients (Beijer et al., 2023; Starkey et al., 2022; Mikolic et al., 2021; Gupte et al., 2019). Somewhat paradoxically, female patients are less likely to be admitted to the ICU and have significantly shorter stays when admitted (Peterson et al., 2022; Mikolic et al., 2021). Taken together, these data suggest that female patients appear to present with symptoms that are considered less life-threatening after the initial injury than those of male patients; however, this lack of initial symptoms may contribute to their long-term negative outcomes. Previous research from the Ferris lab has demonstrated that, using a model of repeated mTBI in male and female rats, female rats showed lower levels of inflammatory mediators soon after injury. However, there was significantly greater anisotropy (a magnetic resonance imaging [MRI] measure of diffusion coefficient that relates to tissue damage) in female rats at 12 days post-injury, suggesting that some of the initial parameters that were interpreted as protection in female rats (e.g., lower inflammatory mediators and BDNF) were not associated with long-term neuroprotection (Dsouza et al., 2024; Bens et al., 2025). Currently, there are no established treatments for chronic mTBI symptoms and no clear predictive biometrics to determine which subjects are at increased risk at the initial clinical presentation (Dujardin et al., 2024). Previous studies have investigated the effects of mTBI on specific time-dependent proteins in both animal and clinical models, although their predictive value for long-term effects remains unclear (Cassidy et al., 2004; Agoston and Elsayed, 2012; Bahado-Singh et al., 2016; Gill et al., 2017; Fiandaca et al., 2018; Kikinis et al., 2017; Oresic et al., 2016). The effects of mTBI on small-molecule endogenous lipid (endolipid) signaling systems as biomarkers for mTBI are largely unknown.

Endogenous cannabinoids, or endocannabinoids (eCBs), are endolipids that are associated with promoting neuroprotective responses after mTBI in animal models (Schurman and Lichtman, 2017). The canonical eCBs, *N*-arachidonoyl ethanolamine (AEA; anandamide) and 2-arachidonyl glycerol (2-AG), are associated with modulating inflammatory responses and preventing excitotoxicity (Shohami et al., 2011). eCBs modulate cellular signaling throughout the nervous system and in the periphery, at least in part, via the cannabinoid CB1 and CB2 receptor signaling. AEA and 2-AG, however, are only two of over 100 congener endolipids that the Bradshaw lab and others have shown to share biosynthetic and metabolic pathways (Castel et al., 2024; Warren et al., 2024; Castel et al., 2023; Leishman et al., 2019; Leishman et al., 2018; Leishman et al., 2016; Bradshaw and Leishman, 2017), be modulated by environmental toxins (Leishman et al., 2017) and drugs of abuse (Leishman et al., 2018; Johnson et al., 2022; Miller et al., 2018), and have additional GPCR and TRP channel targets (Schurman and Lichtman, 2017; Miller et al., 2018; Raboune et al., 2014; Balakrishna et al., 2014; Bradshaw et al., 2013; Miller et al., 2016; Caldwell et al., 2013; McHugh et al., 2012; McHugh et al., 2012; McHugh et al., 2010; Rimmerman et al., 2008). We have also demonstrated changes in this class of endolipids in a rat model of mTBI, focusing primarily on the trigeminal nucleus (Leishman et al., 2019). Each of these prior studies evaluated these endolipids in CNS tissue; however, there is a need to understand how circulating endolipids in the bloodstream may be affected by mTBI to add to the knowledge of blood biomarkers in both rodents and humans. We have demonstrated the ability to reliably measure these endolipids in plasma from both human (Bashashati et al., 2023; Bashashati et al., 2021) and animal studies (Bradshaw and Johnson, 2023; Balaji et al., 2024). In this study, we tested the hypothesis that if plasma endolipids are changed after head injury and this regulation is sex dependent, then the amount and direction of change in plasma endolipids would likewise be sex dependent. To test this hypothesis, we used an mTBI rat model with a within-subject design to determine if there are plasma endolipid changes in a single or repeated head hit paradigm. The goal is to identify potential plasma lipid biomarkers that are differentiated by genetic sex and repeated head trauma.

## Methods

### Subjects

A total of 10 adult male and female Sprague–Dawley rats (*n* = 5/group), 2–3 months old, weighing between 250 and 400 g, underwent 2 days (Day 1, D1, and Day 2, D2) of head hits (HH, *n* = 10, *n* = 5/per genetic sex) to produce mTBI, performed by a closed-head momentum exchange model (*see details below*). This is a within-subject design wherein all subjects underwent four blood draws, two per day. Data were collected by noon during the light phase of the light/dark cycle. Hormonal levels were not evaluated. Subjects were obtained and maintained as described previously (Kulkarni et al., 2019). All methods and procedures described below were pre-approved by the Northeastern University Institutional Animal Care and Use Committee under protocol number 24-0517R-A1: MRI following mild head injury. Northeastern University’s animal care and use program and housing facilities are fully accredited by AAALAC International. The protocols used in this study followed the ARRIVE guidelines for reporting *in vivo* experiments in animal research (Kilkenny et al., 2010).

#### mTBI momentum exchange model procedure

The momentum exchange model that was used to produce a head hit has been detailed in a previous study (Viano et al., 2009). A pneumatic pressure drive 50 g compactor (Rowson et al., 2016) was used to reliably produce mild to severe head injuries in rats and then refined to test the behavioral effects of mild TBI, controlling for the axis of injury, rotational force, and head acceleration (Mychasiuk et al., 2016). Subjects were allowed to acclimate to animal handling 2 weeks before the head hit. This momentum exchange model used an impact velocity of 7.4 m/s, which was determined by using high-speed video recordings. For head hits, the animals were restrained, and the impact piston was directed to the top of the skull, midline, in the approximate area of the bregma. The selection of the impact region was characterized by a large body of data on the effects of acute and long-term mTBI in rodents (Shultz et al., 2012; Xiong et al., 2013; Aungst et al., 2014; Fidan et al., 2016). Hits were done under 2% isoflurane anesthesia, with a 24-h interval between each impact. Rats were returned to their cages after the final mTBI. All blood draws via tail vein and tests occurred during the light phase of a normal light–dark cycle.

In detail, head impacts were generated using a pneumatic pressure drive, a 50 g compactor described by Viano et al. (2009) and refined by Mychasiuk et al. (2016), to reliably produce 7.4 m/s impact velocities described for mild head injury in rats. The kinetic energy at impact is 1.37 joules. We have used this model to publish on the neuroradiological effects of rmTBI in rats (Kulkarni et al., 2019; Cai et al., 2021; Leaston et al., 2021). The impact piston was directed to the top of the skull, midline, in the approximate area of the bregma while rats were fully awake. This model is comparable to CHIMERA, developed for mouse mild TBI (Namjoshi et al., 2014; Namjoshi et al., 2017). All rats showed normal ambulatory behavior within seconds of being placed into their home cage after head impact. There were no mortalities, and there was no evidence of skull damage or contusion. Sham and head-impacted rats were pretreated with one dose of slow-release buprenorphine (0.1 mg/kg IP) 30 min prior to the first head impact to minimize the pain over the 2-day protocol.

**Figure 1:**
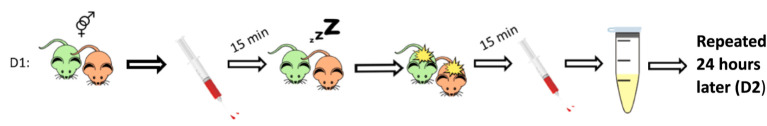
Visual representation of experimental procedure: On day 1 (D1), rats were removed from their cages during the light phase of their light–dark cycle. Fifteen minutes prior to being anesthetized and subsequently receiving impact, whole blood was drawn from their tail veins. Once they were anesthetized, they were placed in the momentum exchange apparatus and received a single heat hit. After 15 min, they once again had their whole blood drawn from the tail vein. The blood was then centrifuged for 5 min, and the plasma was removed and stored at −80 °C. This process was repeated after 24 h for day 2 (D2).

Whole blood samples were collected before the first head hit on each day to establish a baseline and then 15 min after a hit. Samples were centrifuged for 5 min, and the plasma was removed and stored at −80 °C until shipped on dry ice to Indiana University Bloomington, where it was returned to −80 °C until further processing. A visual representation of the experimental procedure is shown in Figure 1.

### Lipid extraction and partial purification

Lipidomics analysis of plasma was performed as outlined in our recent Methods chapter on analyzing eCBs and related lipids in biological fluids (Bradshaw and Johnson, 2023) and as previously described in human plasma samples (Bashashati et al., 2023; Bashashati et al., 2021). In brief, plasma samples (75 μL) were vortexed and added to 2 mL of high-performance lipid chromatography (HPLC)-grade methanol (MeOH) + 5 μL of 1 μM 500 picomolar (pM) deuterium-labeled anandamide (d8AEA) as an internal standard to determine extraction efficiency. The samples were incubated in the dark on ice for 30 min before centrifugation (19,000 x *g* for 20 min at 20 °C). The supernatant was added to 8 mL of HPLC-grade water for an approximately 25% organic solution and partially purified using Agilent C-18 solid phase extraction (SPE) columns (Santa Clara, CA, USA), as previously described (Balaji et al., 2024). The following 1.0 mL elutions of 65, 75, and 100% methanol were collected for high-performance lipid chromatography/mass spectrometry/mass spectrometry (HPLC/MS/MS) and stored in a −80 °C freezer until further processing. The samples were de-identified and recorded by processing day.

**Figure 2:**
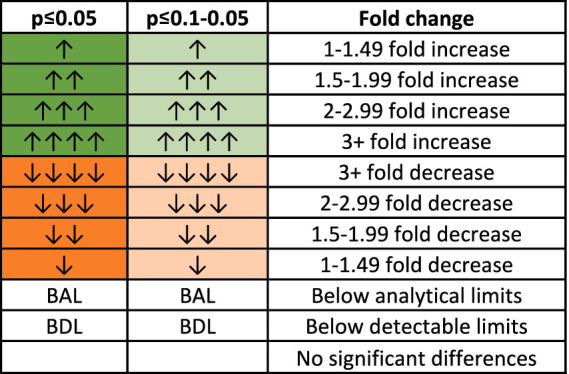
Heatmap legend heatmaps are used to summarize mean data and statistical significance to provide a shorthand for large-scale lipidomics datasets. Dark green represents either significant increases (after treatment) or levels that are significantly higher (sex differences), *p* ≤ 0.05, while light green represents significance levels of *p* ≤ 0.1–0.05. Dark orange represents significant decreases (after treatment) or levels that are significantly lower (sex differences) wherein *p* ≤ 0.05, while light orange represents significance levels of *p* ≤ 0.1–0.05. Fold change is indicated by the number of arrows, where 1 arrow corresponds to a 1–1.49-fold difference, 2 arrows to a 1.5–1.99-fold difference, 3 arrows to a 2–2.99-fold difference, 4 arrows to a 3–9.99-fold difference, and 5 arrows to a difference of tenfold or more. BAL (below analytical limits) refers to endolipids that were measured in at least one sample but less than 4, making statistical analysis unreliable. BDL (below detectable limits) refers to endolipids that were not detected in any samples. Blank cells indicate that endolipids were present in at least 4 samples in each treatment group being analyzed and that no significant differences were present.

### HPLC/MS/MS analysis

HPLC/MS/MS analyses, including multiple reaction monitoring (MRM) parameters and chromatograms, were identical to those established in our lab over 15 years (Leishman et al., 2016; Smoum et al., 2010; Leishman et al., 2016; Tortoriello et al., 2013). In brief, the samples were removed from the −80 °C freezer and allowed to warm to room temperature. They were then vortexed for approximately 1 min before being placed into the autosampler held at 24 °C (Agilent 1,100 series autosampler, Palo Alto, CA, USA) for LC/MS/MS analysis. A 20 μL injection volume from each sample was injected into a C18 Analytical Column (Agilent Technologies, Santa Clara, CA, USA) to scan for compounds. Gradient elution (200 μL/min) then occurred under the pressure created by two Shimadzu 10AdVP pumps (Columbia, MD, USA) (mobile phase A: 20% HPLC methanol, 80% HPLC water, and 1 mM ammonium acetate; mobile phase B: 100% HPLC methanol and 1 mM ammonium acetate). Next, electrospray ionization was accomplished using an Applied Biosystems/MDS Sciex (Foster City, CA, USA) API3000 triple quadrupole mass spectrometer. A multiple reaction monitoring (MRM) setting on the LC/MS/MS was then used to analyze levels of each compound present in the sample injection. A total of 16 lipid families were screened for a total of 86 individual compounds, one of which was the internal standard d8AEA. Synthetic standards were used to generate optimized MRM methods and standard curves for analysis (Leishman et al., 2019; Leishman et al., 2016; Johnson et al., 2022).

### Statistical analysis

The goal of this exploratory study was to examine the following:

1) Do subjects show a change in plasma endolipids immediately after a head hit?
2) If subjects showed any changes in plasma endolipids after a single head hit, is this change repeated in the same subjects after a second head hit 24 h later?
3) If there is a change in plasma endolipids after a single head hit, is this change the same in male and female rats?
4) If there is a change in plasma endolipids after a second head hit 24 h after the initial head hit, is this change the same in male and female rats?
5) If there are sex differences in endolipids in each of these categories.

Due to the within-subject study design that does not include a separate set of subjects with no treatment, the goals of this study do not include the following:

1) If levels of endolipids change over time within and between subjects.
2) If levels of a specific endolipid change as a function of another specific endolipid.

**Figure 3:**
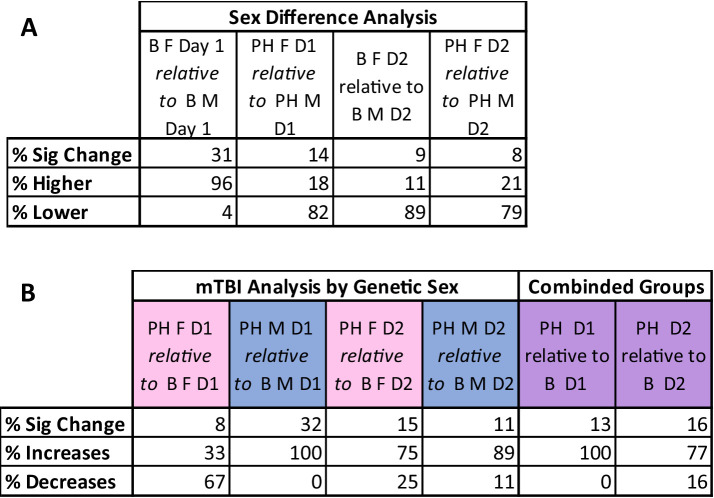
Summary of percent change in endolipids within and between groups. **(A)** shows percent changes (overall significant, higher, or lower) between female and male rats at each plasma evaluation time point in the study. **(B)** shows percent changes (overall significant, increases, or decreases to treatment) within genetic sex and combined female and male rats, comparing pre- and post-head hit.

**Figure 4:**
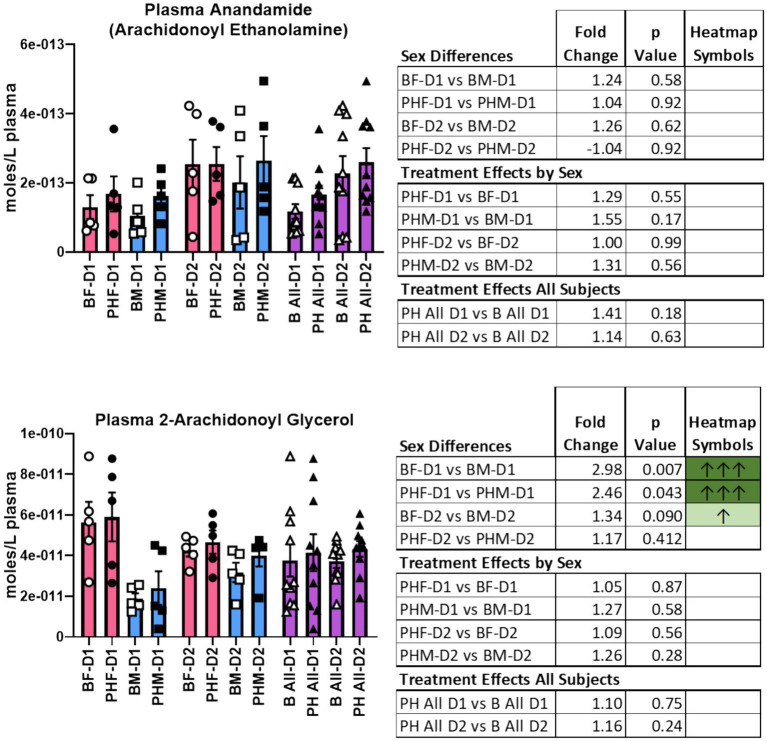
Levels of anandamide and 2-arachidonoyl glycerol (2-AG) in plasma in eight different treatment conditions. The bar graphs on the left side are the mean ± SEM of the levels of endogenous lipids in plasma in each treatment group [female (F) rats in pink, male (M) rats in blue, and combined (all) in purple]. Baseline (B), post-hit (PH), and days 1 and 2 (D1, D2). The table on the right side of the figure lists the analytical data from the interactions of the listed comparisons. *See Methods for a description of data analysis.*

**Figure 5:**
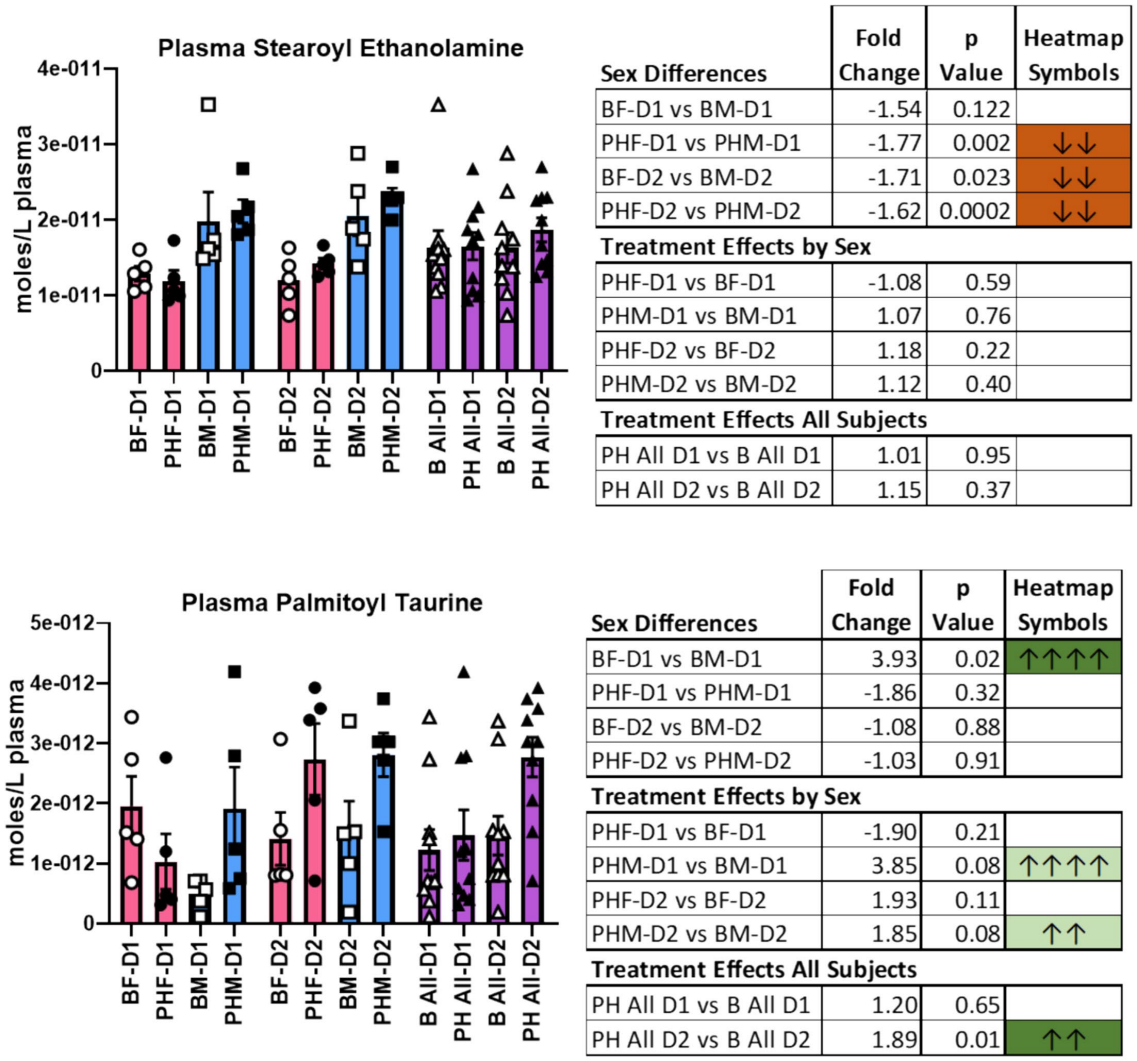
Levels of stearoyl ethanolamine and palmitoyl taurine in plasma in eight different treatment conditions. The bar graphs on the left side are the mean ± SEM of the levels of endogenous lipids in plasma in each treatment group (female (F) rats in pink, male (M) rats in blue, and combined (All) in purple). Baseline (B), post-hit (PH), and days 1 and 2 (D1, D2). The table on the right side of the figure lists the analytical data from the interactions of the listed comparisons. *See Methods for* a *description of data analysis*.

Initial analysis of variances (ANOVAs) for all treatments as a function of the individual endolipids is provided in Supplementary Data. Post-hoc analysis of individual endogenous lipids in plasma in the following comparison groups was analyzed using Student’s t-tests set to two tails and Type 2: male vs. female baseline (F B) day 1, M vs. F post-hit (PH) D1; M vs. F B Day 2, M vs. F PH D2; F B vs. F PH D1, M B vs. M PH D1, F B vs. F PH D2, and M B vs. M PH D2. Statistical analyses were completed using IBM SPSS Statistics 29 (Chicago, IL, USA) as previously described (Leishman et al., 2016). There was no analysis of one baseline across multiple time points, nor were there comparisons of one species of lipid across time points and genetic sex. All statistical comparisons were single, individual comparisons of pre–post head hits on a specific day. Figures 2–5 illustrate these specific comparisons for six different endolipids. Statistical significance for all tests was set at a *p*-value of < 0.05, and trending significance was set at 0.05 < *p* < 0.10. Descriptive and inferential statistics were used to create heatmaps for visualizing changes in the concentration of each lipid analyte for every condition (Figure 2). Briefly, the direction of changes for each analysis group compared to the others is depicted by color, with green representing an increase and orange representing a decrease. The level of significance is shown by color shade, wherein *p* < 0.05 is a dark shade and 0.05 < *p* < 0.1 is a light shade. The direction of the change compared to the vehicle group is represented by up (increase) or down (decrease) arrows. The effect size is represented by the number of arrows, where 1 arrow corresponds to a 1–1.49-fold difference, 2 arrows to a 1.5–1.99-fold difference, 3 arrows to a 2–2.99-fold difference, 4 arrows to a 3–9.99-fold difference, and 5 arrows to a difference of tenfold or more (Stuart et al., 2013). An abbreviation of ‘BDL’ indicates that the lipid concentration that was present in the sample was below the detectable levels of our equipment, while ‘BAL’ indicates below analytical levels. To calculate the effect size of a lipid that was significantly higher in a particular group (male and female rats by baseline or post-hit by day for sex difference analysis and baseline and post-hit conditions by day of the same sex for the genetic sex analysis), the average concentration of the respective comparison group was divided by the average concentration of the vehicle group. To calculate the effect size of a compound that was significantly lower in the treatment group, the same calculation was conducted, except the inverse of the resulting ratio was used to represent a fold decrease. Finally, bar graphs show data as mean ± SEM.

**Figure 6:**
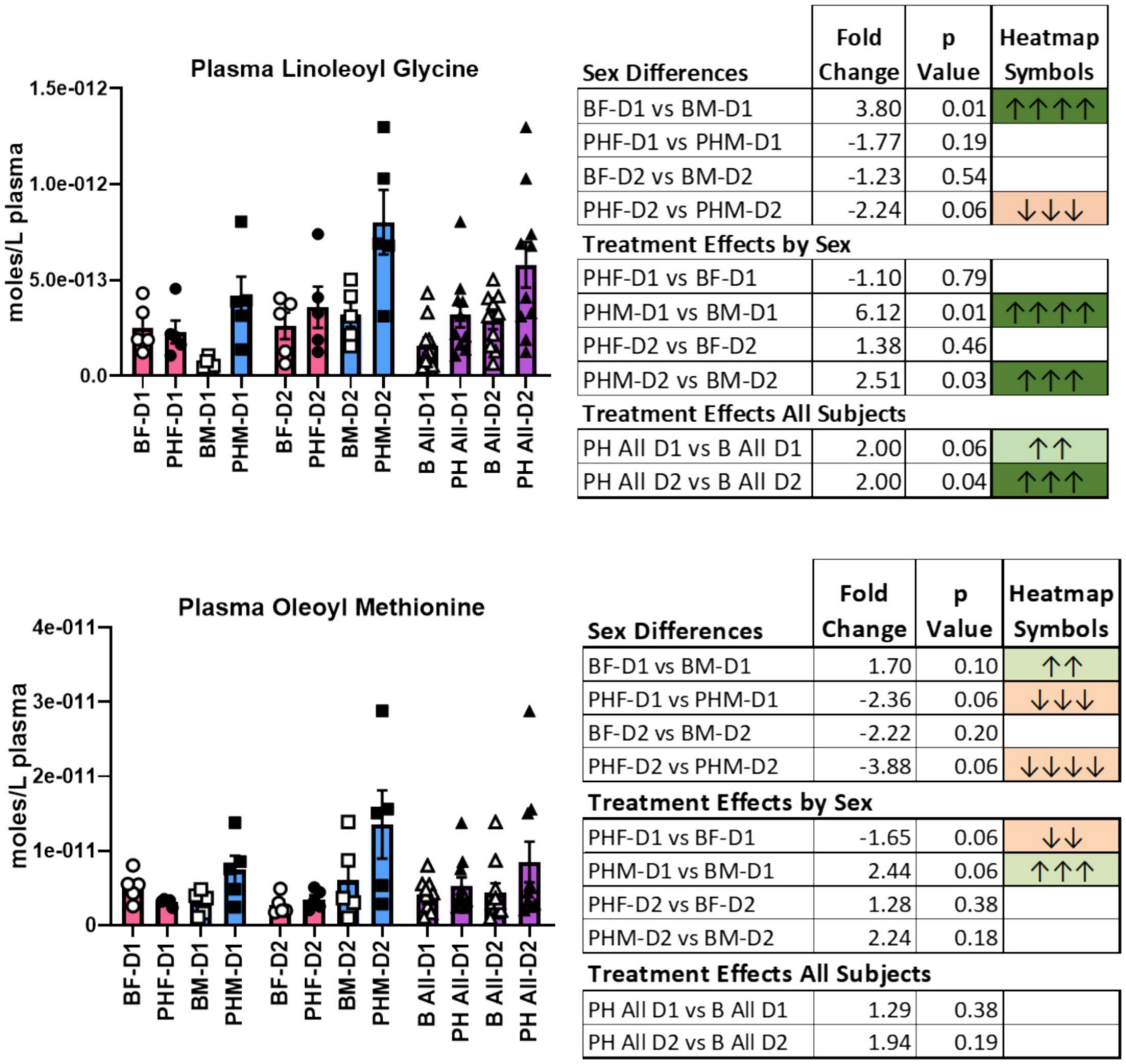
Levels of linoleoyl glycine and oleoyl methionine in plasma in eight different treatment conditions. The bar graphs on the left side are the mean ± SEM of the levels of endogenous lipids in plasma in each treatment group (female (F) rats in pink, male (M) rats in blue, and combined (all) in purple). Baseline (B), post-hit (PH), and days 1 and 2 (D1, D2). The table on the right side of the figure lists the analytical data from the interactions of the listed comparisons. *See Methods for a description of data analysis*.

## Results

### Overall effects of differences between and within sex and treatment groups on plasma lipids

Between the 2 days of head hits and subject groups (female: F and male: M), 86 endolipids in the screening library were detected in at least one plasma sample (see Supplementary Figures S1, S2 for a *full list of endolipids screened and p-values for all interactions analyzed*). Of these 86 compounds, 87.2% (75/86) met analytical limits for an exploratory study (i.e., detected in at least three out of five subjects). Of those 75, 69% (52/75) demonstrated significant differences in at least one comparison across all potential interactions.

Figure 3 provides a summary of the percentage and direction of change between the subject and treatment groups. These are organized as sex differences at each of the four blood collection time points (3A), then as treatment effects within genetic sex subject groups, and then finally as combined (F and M) for each of the 2 days of head-hit treatments (3B). A summary of the sex difference analysis shows that levels of 31% of plasma endolipids at baseline on day 1 were significantly different, with 96% of those endolipids being higher in female rats. The percentage of endolipids showing significant sex differences after the first head hit dropped to 14%, and the direction was reversed, with 82% being lower in female rats. Sex differences at baseline on day 2 of testing dropped to 9% and then 8% after the head hit. In both cases, the levels were lower in female rats: 89 and 79%, respectively.

The summary of single and repeated head hit effects by genetic sex (Figure 3B) revealed that on day 1 of testing, a single head hit caused only 8% of plasma lipids to change in female rats but 32% in male rats. Whereas the majority (67%) of these changes in female rats were decreases, in contrast, 100% of the changes in male rats were increases. On day 2 of testing, female rats showed a 15% difference in plasma lipids after the second head hit (with 75% increases) within 24 h, and male rats 11% (with 89% increases). Combining the female and male rats provided a different outcome for day 1 data, with a total of 13% of lipids changed (closer to the female percentage) after the day 1 head hit and 100% being increased (aligning with the male data). The day 2 combined results were more closely aligned with both female and male data, with a 16% overall change and 77% of that being increases.

### Lipidomics evaluations between the subject and treatment group

**Figure 7:**
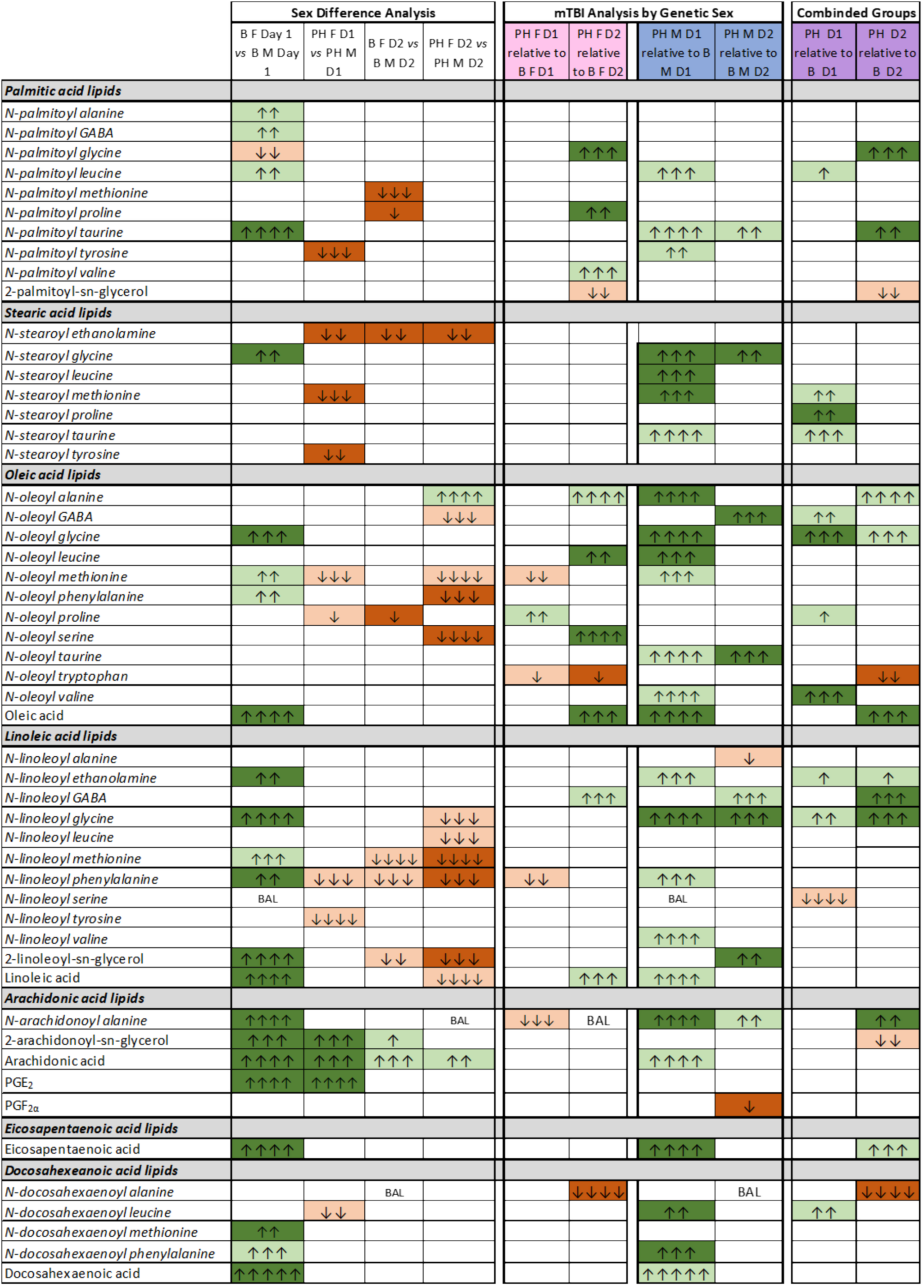
Heatmap of plasma endolipids across all treatment conditions. Data are represented as comparisons of genetic sex in eight different treatment conditions (far left columns 1–4); comparisons of pre–post mTBI head hit within genetic sex (columns 5–8); and comparisons of pre–post mTBI head hit with genetic sex combined (columns 9, 10). Analytical groups are female (F) rats, male (M) rats, baseline (B), post-hit (PH), and days 1 and 2 (D1, D2). *See Figure* *2* *for a detailed explanation of heatmap fields.* Endogenous lipids are organized by acyl group (fatty acid side chain) and are listed from the shortest acyl chain and lowest number of double bonds (palmitic acid C:18:0) to the longest acyl chain and the highest number of double bonds (docosahexaenoic acid C:22:6). Lipoamines/lipo amino acids are designated as the *N*-acyl group amine, followed by 2-acyl glycerols and free fatty acids.

Figures 4–6 illustrate the levels of six different endolipids in each subject and treatment type point and then with the female and male data combined pre– and post–head hit on consecutive days. These examples include the endocannabinoids anandamide (4A) and 2-AG (4B) and their endogenous congeners stearoyl ethanolamine (5A), palmitoyl taurine (5B), linoleoyl glycine (6A), and oleoyl methionine (6B). Each of these examples is shown again in Figure 7 as part of the larger heatmap. For each endolipid illustrated, the mean with SEM is shown on the left. On the right, a table shows the analytical interactions that are used to generate the larger heatmaps, including fold change difference, *p*-value, and heatmap icon between the listed interaction (e.g., baseline female Day 1: BF D1 versus baseline male Day 1: BM D1; Supplementary Figures for all analyses).

Levels of anandamide (4A) showed no sex differences or treatment differences within each treatment day. While the bar graphs suggest that levels of AEA increased overall on day 2 of the treatment, we do not have sufficient data to attribute this solely to the mTBI treatment, as there is not a day 1 control group receiving only restraint without a head hit (*see Methods for further explanation of analysis protocols*). Therefore, all data are analyzed and reported as the interactions outlined in the tables in Figures 3–5 and are within the day of treatment. Comparisons between treatment days are of the outcomes of these within-day analyses. As an example, Figure 3B provides evidence that BF D1 vs. BM D1 and PHF D1 and PHM D1 are significantly different, with female 2-AG levels being significantly higher in both conditions. Likewise, BF D2 levels of 2-AG are significantly higher; however, in the *p* < 0.10–0.051 range (*p* = 0.09). In contrast, the PHF D2 levels of 2-AG are equivalent to PHM D2 (*p* = 0.412). By contrast, all the within-genetic-sex comparisons and the combination of female and male data of pre– and post–hit on both D1 and D2 are equivalent.

The close structural congener to anandamide, stearoyl ethanolamine (SEA), demonstrates very different expression patterns compared with either AEA or 2-AG (5A). Levels of SEA are significantly lower in female rats than in male rats at 3 of the 4 time points evaluated. However, there was no effect of treatment. Another sex difference pattern is demonstrated where palmitoyl taurine (5B) levels are significantly higher in female rats at baseline on D1 than in male rats, with no differences at any other time point. Palmitoyl taurine shows increases with head hits in male rats (*p* = 0.08 on both D1 and D2), and female rats show a non-significant trend to increasing after the D2 hit (*p* = 0.11), which then translates to a significant increase of *p* = 0.01 on D2 when female and male data are combined.

Plasma levels of linoleoyl glycine (6A) and oleoyl methionine (6B) demonstrate similar patterns to each other; however, they are different from the first four endolipids described here. In both cases, female levels are significantly higher at baseline on D1 than male levels and then become more equivalent on D2. Likewise, female levels are lower than male levels after a head hit on both days, with fold change levels being more pronounced and p-levels lower for linoleoyl glycine. It is this more pronounced increase in male rats after a head hit that drives the significant increase in linoleoyl glycine in the combined data, rather than any trending increases in female rats. These increases were not as pronounced for oleoyl methionine in males when female levels remained unaffected, leading to equivalent levels of oleoyl methionine in the combined data analysis.

We provide these examples to highlight that the heatmap data offer an important snapshot of the overall effects of treatment and genetic sex; however, as with all large-scale data analyses, especially those combining female and male data, it should be interpreted with these types of nuances. Figure 7 illustrates the heatmap for the endolipids that showed significant changes in at least one analytical interaction. *The heatmap for all lipids screened and the p-values for all interactions are provided in* *Supplementary Data*. The data are organized by the acyl chain (e.g., palmitic acid and linoleic acid). This Supplementary Data provides fold change, significance range, and direction of change for all the lipids summarized in Figure 6. Of note, the direction of difference between female and male rats reverses after the head hit on day 1; however, the within-female analysis shows that no plasma lipids analyzed within 15 min of the head hit on D1 were significant in the *p* < 0.05 range, and this interaction (PH F D1 relative to B F D1) represents the comparison with the least overall effect. By dramatic contrast, the effect of head hit on day 1 in male rats shows the most overall effect of these comparisons, and all comparisons show increases in these endolipids. While D2 in male rats still shows significant increases, there are fewer overall and two lipids that decrease. Likewise, D2 in female rats has an overall expression that resembles that of D2 in male rats, with mostly increases and three decreases.

## Discussion

These data are an exploratory evaluation of over 80 plasma endolipids, including the eCBs, anandamide and 2-AG, in plasma directly before and after single and then repeated mTBI injury in female and male rats. These findings provide novel data for evaluating blood biomarkers for predicting mTBI injury effects, as this endogenous lipid class is abundant in human plasma as well (Bashashati et al., 2023; Bashashati et al., 2021). Key findings show that there are significant sex differences in both the baseline levels of these endolipids and how these are modified after injury. Importantly, the largest modulations of plasma endolipids measured after mTBI are primarily increases in plasma lipid concentrations and are primarily observed in male rats.

### Sex differences in the effects of mTBI

The notion of biological sex being a modulator of mTBI outcomes is not a new idea. Epidemiological data suggest that females tend to report more negative outcomes over time than males after mTBI; however, the majority of direct comparisons of how male and females differ are after acute insult (Peterson et al., 2022; Mikolic et al., 2021; Albanese et al., 2017; Styrke et al., 2013). Bazarian et al. have suggested that mTBI affects the regulation of hormones, progesterone, and estrogen, as a potential explanation for poorer mTBI outcomes for women (Bazarian et al., 2010). Men do produce these hormones as well, but in much smaller amounts (Han et al., 2009), suggesting there may be a production- or concentration-dependent effect. In addition to our previous study showing an increase in diffusion coefficients primarily in female rats over time after repeated mTBI (Bens et al., 2025), Wright et al. have shown that MRI analysis on the subjects after repeated mTBI found that female rats experienced greater atrophy of the prefrontal cortex and that there were also differences in the expression of mRNA for various proteins, including tau protein, which is commonly associated with Alzheimer’s disease (Wright et al., 2017).

Male rats can also show deficits after mTBI. Tucker et al. have investigated the long-term effects of repeated mTBI in mice and showed that impairments remained for up to 6 months after injury, with male rats showing more significant motor deficits than female rats (Tucker et al., 2019). Meanwhile, the study by Wright et al. has also conducted cognitive-behavioral tests over time and found that mTBI led to depressive-like behavior in female rats but cognitive deficits in male rats (Wright et al., 2017). The effect of aging is also an important factor to consider. A recent study that utilized only male rats found that when compared to older rats, young mice showed worse physiological outcomes and postulated that it may be due to an incomplete development of an immune or inflammatory response (Islam et al., 2021). An alternative explanation could be that there was a much more robust immune response due to more collateral damage from microglia (Chen and Trapp, 2016). A clinical report somewhat converges on these reports, as it suggests that a single mTBI event can begin neurodegeneration that will persist with aging, which can lead to increased vulnerability for mTBI at older ages (Tremblay et al., 2019). In this study, we also introduce the idea that the baseline sex differences in endolipids may be an additional avenue to explore when investigating how mTBI-inducing injuries can have differential outcomes. However, given that the hormonal cycle was not evaluated in this exploratory study, this baseline difference could be a function of changes across the cycle. Future studies would benefit from this level of analysis.

### Changes in eCBs and their congeners with mTBI

Anandamide and 2-AG are the most well-known eCBs and have a variety of functions, including neuroprotective roles, especially relevant for mTBI. Data in this current study show that male and female rats experienced no overall effect from single mTBI in plasma eCBs. However, this does not preclude eCBs changing in other parts of the CNS and body. Previous findings from Panikashvili et al. have shown 2-AG increases in the brain during periods of injury (Panikashvili et al., 2001) and in plasma during inflammation (Panikashvili et al., 2006). In this study, we observed no immediate changes in plasma within the time frame of acute injury.

#### Changes in FFAs with mTBI

In addition to the cognitive changes due to head injury, there are also metabolic changes that have yet to be fully characterized. Specifically, the metabolic changes involving free fatty acids can also lead to prolonged metabolic dysfunction (Lai et al., 2022), as many of their oxidative products, excluding resolvins (Anthonymuthu et al., 2017), are pro-inflammatory (Dyall, 2017). All five FFAs measured increased after head hits in male rats on day 1, with no increases measured in female rats. Importantly, baseline levels of FFAs on day 1 of the head hits show that female rats have significantly higher levels of FFAs in all five species evaluated. This difference was not present at baseline on day 2 for all FFAs except arachidonic acid. Essentially, these data indicate that levels of FFAs were only affected in male rats after the initial head hit. While this may seem counterintuitive to a protection mechanism as many FFAs are converted to pro-inflammatory mediators, the FFAs eicosapentaenoic and docosahexaenoic acids can be converted to compounds such as resolvins, which may act to protect against inflammatory damage (Dyall, 2017; Serhan and Petasis, 2011). The fact that the significantly higher levels of FFAs in female rats at baseline were not present in day-2 baseline could be an effect of the treatment on day one, of overall stress of the procedure, of hormonal cycle differences, or a combination of all of these factors. As an exploratory study, the aim was to first determine if single and repeated head hits could alter plasma endolipid signaling molecules and if this is sex dependent. Given that the data here clearly illustrate that they do with day 1 analysis alone, the complexity of repeated head hits with potential stress and hormonal modulations of the lipids can be addressed in future studies.

### Changes in lipo amino acids with mTBI

Lipo amino acids (*a.k.a. lipoamines, N-acyl amino acids, and N-acyl amides*) refer to the endolipid congeners of *N*-acyl ethanolamines that are conjugations of free fatty acids and amino acids. The data here highlight modulations in multiple lipo amino acids, including *N*-acyl glycine, *N*-acyl taurine, *N*-acyl phenylalanine, and *N*-acyl leucine. Prior data from our lab showed that *N*-acyl glycines are upregulated after mTBI in the CNS (Leishman et al., 2019), which agrees with the data in the current study for male rats (as shown in Figures 5, 7). Previous data were collected from a male-only cohort, so we cannot determine if female CNS levels are likewise affected. In this study, data show that the increases in *N*-acyl glycine concentrations after head hits are observed exclusively in male rats. Recent evidence has supported the importance of *N*-acyl glycines for neuroprotective effects after traumatic brain injury (Viano et al., 2009; Rowson et al., 2016) and reperfusion after ischemic stroke (Mychasiuk et al., 2016). TBI, or head injury, has the potential to injure vascular networks in the brain (Shultz et al., 2012), and those mechanistic responses involve small-molecule lipids (Xiong et al., 2013), which offers a potential explanation as to why so many different endolipid species were so affected due to mTBI.

This pattern of increasing levels of plasma endolipids in male rats and not female rats in this study was the same for *N*-acyl taurines, *N*-acyl leucines, and *N*-acyl methionines. Prior research from our group has shown that *N*-arachidonoyl-taurine (A-taur) increased in the CNS after mTBI (Leishman et al., 2019); however, plasma levels of A-taur were not in the analytical range in plasma to make this determination. Previous screens from our lab did not evaluate additional *N*-acyl taurines in the CNS, so the data here indicating that the more abundant plasma *N*-palmitoyl-taurine and *N*-stearoyl-taurine measured a 4-fold increase in concentration in male rats after the first day of mTBI indicated that this family of lipids appears to be upregulated after head hits in multiple areas of the body. The literature on the regulation of *N*-acyl taurines is sparse, and not much is known about their signaling roles other than to activate some TRP channels (Raboune et al., 2014; Balakrishna et al., 2014; Bradshaw et al., 2013; Hanus et al., 2014; Manchanda et al., 2021); therefore, this family of endogenous lipids provides a novel direction for future study.

### Development of lipid biomarkers in plasma as disease outcome predictors

A recent study from our group (Balaji et al., 2024) has shown that the systemic injection of the endolipid, palmitoyl ethanolamine (PEA; 3, 10, or 30 mg/kg), caused an inverse dose–response for negative BOLD, suggesting a decrease in brain activity affecting the prefrontal cortex, sensorimotor cortices, basal ganglia, and thalamus. However, there was a dose-dependent increase in functional connectivity in these same brain areas. Plasma and CNS levels of PEA and over 80 endolipids were determined after treatment. While levels of PEA in the plasma and CNS were significantly higher after 30 mg/kg treatment, levels of the endocannabinoid, anandamide, and at least 20 additional endolipids were significantly lower across the CNS, with differential regulation in the plasma. Taken together, the functional connectivity and lipidomics changes provide evidence that changes in peripheral endolipids have the potential to substantially change CNS physiology.

Utilizing quantifiable, endogenous indicators of illnesses or diseases in the blood has helped healthcare providers assess potential outcomes, which can range from cancer to neurodegenerative diseases. However, with mTBI, determining these biomarkers and then linking them to future outcomes has been more difficult due, in part, to the variable nature of the condition. Previous studies have identified potential proteins, enzymes, and amino acids as candidates (Fiandaca et al., 2018; Shahim et al., 2018; Newcombe et al., 2022; Korley et al., 2022; Posti and Tenovuo, 2022), but none to date have proven to be clinically predictive. An examination of a broader range of plasma endolipids, as analyzed in this study, could provide additional avenues for evaluations of short-term effects from mTBI that may be related to long-term outcomes. Lipidomics data presented here provide a novel framework to investigate potential plasma endolipid biomarkers for mTBI that are relevant to understanding the sex differences in initial treatment protocols during clinical presentation that may contribute to negative long-term outcomes for female patients.

## Data Availability

The original contributions presented in the study are included in the article/Supplementary material, further inquiries can be directed to the corresponding author.
