# Supplementary material for: Sex differences in plasma endocannabinoids and related lipids before and after single and repeated mTBI: an exploratory study of endolipid plasma biomarkers
Source: Front Mol Neurosci. 2026 Jun 18;19:1707732. doi: 10.3389/fnmol.2026.1707732 (PMC13323484; doi:10.3389/fnmol.2026.1707732)
Supplement: Supplementary file 1 [file Data_Sheet_1.pdf]

# Oneway

## Notes

| | | |
|---|---|---|
| Output Created | | 05-MAR-2026 22:21:12 |
| Comments | | |
| Input | Data | C:\Users\hbbradsh\One Drive - Indiana University\Collaborators\Craig Ferris\Head and Shoulers_2023\260305_mTBI_plasma_NE_Head only.sav |
| | Active Dataset | DataSet1 |
| | Filter | <none> |
| | Weight | <none> |
| | Split File | <none> |
| | N of Rows in Working Data File | 40 |
| Missing Value Handling | Definition of Missing | User-defined missing values are treated as missing. |
| | Cases Used | Statistics for each analysis are based on cases with no missing data for any variable in the analysis. |
| Syntax | | ONEWAY twoAG twoLG twoOG twoPG AA OA EIC DOC LIN NAGly LinGly PalGly OlGly DocGly StrGly PEA SEA OEA AEA LEA DEA StrTaurine OlTaurine NATaurine PalTaurine |

| | | |
|---|---|---|
| | | PalLeucine LinLeucine DocLeucine StrLeucine OlLeucine PalPhenyl StePhenyl OlePhenyl LinPhenyl AraPhenyl DocPhenyl palAla steAla oleAla linAla araAla docAla palGABA steGABA oleGABA linGABA araGABA docGABA palMeth steMeth oleMeth linMeth araMeth docMeth PGEtwo PGFtwoA SixketoPGFOneA palProline steProline oleProline linProline araProline docProline palSerine steSerine oleSerine linSerine araSerine docSerine palTrypt steTrypt oleTrypt linTrypt araTrypt docTrypt palTyr steTyr oleTyr linTyr araTyr docTyr palVal steVal oleVal linVal docVal BY Treatment /ES=OVERALL /MISSING ANALYSIS /CRITERIA=CILEVEL(0.95) /POSTHOC=LSD ALPHA(0.05). |
| Resources | Processor Time | 00:00:00.02 |
| | Elapsed Time | 00:00:00.10 |

## Warnings

| |
|---|
| Post hoc tests are not performed for araAla because at least one group has fewer than two cases. |
| Post hoc tests are not performed for docAla because at least one group has fewer than two cases. |
| Post hoc tests are not performed for SixketoPGFOneA because at least one group has fewer than two cases. |
| Post hoc tests are not performed for steProline because at least one group has fewer than two cases. |
| Post hoc tests are not performed for linSerine because at least one group has fewer than two cases. |
| Post hoc tests are not performed for araSerine because at least one group has fewer than two cases. |
| Post hoc tests are not performed for docSerine because at least one group has fewer than two cases. |
| Post hoc tests are not performed for linTrypt because at least one group has fewer than two cases. |
| Post hoc tests are not performed for araTrypt because at least one group has fewer than two cases. |
| Post hoc tests are not performed for docTrypt because at least one group has fewer than two cases. |
| Post hoc tests are not performed for docTyr because at least one group has fewer than two cases. |

## ANOVA

| | | Sum of Squares | df | Mean Square | F | Sig. |
|---|---|---|---|---|---|---|
| twoAG | Between Groups | .000 | 7 | .000 | 4.019 | .003 |
| | Within Groups | .000 | 32 | .000 | | |
| | Total | .000 | 39 | | | |
| twoLG | Between Groups | .000 | 7 | .000 | 5.434 | <.001 |
| | Within Groups | .000 | 32 | .000 | | |

| | | | | | | |
|---|---|---|---|---|---|---|
| | Total | .000 | 39 | | | |
| twoOG | Between Groups | .000 | 7 | .000 | 1.126 | .372 |
| | Within Groups | .000 | 32 | .000 | | |
| | Total | .000 | 39 | | | |
| twoPG | Between Groups | .000 | 7 | .000 | 2.312 | .050 |
| | Within Groups | .000 | 32 | .000 | | |
| | Total | .000 | 39 | | | |
| AA | Between Groups | .000 | 7 | .000 | 4.495 | .001 |
| | Within Groups | .000 | 32 | .000 | | |
| | Total | .000 | 39 | | | |
| OA | Between Groups | .000 | 7 | .000 | 2.773 | .023 |
| | Within Groups | .000 | 32 | .000 | | |
| | Total | .000 | 39 | | | |
| EIC | Between Groups | .000 | 7 | .000 | 2.251 | .056 |
| | Within Groups | .000 | 32 | .000 | | |
| | Total | .000 | 39 | | | |
| DOC | Between Groups | .000 | 7 | .000 | 2.330 | .049 |
| | Within Groups | .000 | 32 | .000 | | |
| | Total | .000 | 39 | | | |
| LIN | Between Groups | .000 | 7 | .000 | 3.884 | .004 |
| | Within Groups | .000 | 32 | .000 | | |
| | Total | .000 | 39 | | | |
| NAGly | Between Groups | .000 | 7 | .000 | .872 | .541 |

| | | | | | | |
|---|---|---|---|---|---|---|
| | Within Groups | .000 | 26 | .000 | | |
| | Total | .000 | 33 | | | |
| LinGly | Between Groups | .000 | 7 | .000 | 5.410 | <.001 |
| | Within Groups | .000 | 32 | .000 | | |
| | Total | .000 | 39 | | | |
| PalGly | Between Groups | .000 | 7 | .000 | 1.989 | .088 |
| | Within Groups | .000 | 32 | .000 | | |
| | Total | .000 | 39 | | | |
| OlGly | Between Groups | .000 | 7 | .000 | 2.626 | .029 |
| | Within Groups | .000 | 32 | .000 | | |
| | Total | .000 | 39 | | | |
| DocGly | Between Groups | .000 | 7 | .000 | 1.787 | .143 |
| | Within Groups | .000 | 21 | .000 | | |
| | Total | .000 | 28 | | | |
| StrGly | Between Groups | .000 | 7 | .000 | 1.622 | .165 |
| | Within Groups | .000 | 32 | .000 | | |
| | Total | .000 | 39 | | | |
| PEA | Between Groups | .000 | 7 | .000 | .768 | .618 |
| | Within Groups | .000 | 32 | .000 | | |
| | Total | .000 | 39 | | | |
| SEA | Between Groups | .000 | 7 | .000 | 5.452 | <.001 |
| | Within Groups | .000 | 32 | .000 | | |
| | Total | .000 | 39 | | | |

| | | | | | | |
|---|---|---|---|---|---|---|
| OEA | Between Groups | .000 | 7 | .000 | .666 | .699 |
| | Within Groups | .000 | 32 | .000 | | |
| | Total | .000 | 39 | | | |
| AEA | Between Groups | .000 | 7 | .000 | 1.274 | .294 |
| | Within Groups | .000 | 32 | .000 | | |
| | Total | .000 | 39 | | | |
| LEA | Between Groups | .000 | 7 | .000 | 2.272 | .054 |
| | Within Groups | .000 | 32 | .000 | | |
| | Total | .000 | 39 | | | |
| DEA | Between Groups | .000 | 7 | .000 | 1.422 | .231 |
| | Within Groups | .000 | 32 | .000 | | |
| | Total | .000 | 39 | | | |
| StrTaurine | Between Groups | .000 | 7 | .000 | 1.837 | .121 |
| | Within Groups | .000 | 27 | .000 | | |
| | Total | .000 | 34 | | | |
| OlTaurine | Between Groups | .000 | 7 | .000 | 1.691 | .149 |
| | Within Groups | .000 | 30 | .000 | | |
| | Total | .000 | 37 | | | |
| NATaurine | Between Groups | .000 | 7 | .000 | .563 | .779 |
| | Within Groups | .000 | 27 | .000 | | |
| | Total | .000 | 34 | | | |
| PalTaurine | Between Groups | .000 | 7 | .000 | 2.656 | .028 |
| | Within Groups | .000 | 32 | .000 | | |

| | | | | | | |
|---|---|---|---|---|---|---|
| | Total | .000 | 39 | | | |
| PalLeucine | Between Groups | .000 | 7 | .000 | 1.336 | .266 |
| | Within Groups | .000 | 32 | .000 | | |
| | Total | .000 | 39 | | | |
| LinLeucine | Between Groups | .000 | 7 | .000 | 3.581 | .006 |
| | Within Groups | .000 | 32 | .000 | | |
| | Total | .000 | 39 | | | |
| DocLeucine | Between Groups | .000 | 7 | .000 | 1.667 | .153 |
| | Within Groups | .000 | 32 | .000 | | |
| | Total | .000 | 39 | | | |
| StrLeucine | Between Groups | .000 | 7 | .000 | 2.106 | .072 |
| | Within Groups | .000 | 32 | .000 | | |
| | Total | .000 | 39 | | | |
| OlLeucine | Between Groups | .000 | 7 | .000 | 1.236 | .313 |
| | Within Groups | .000 | 32 | .000 | | |
| | Total | .000 | 39 | | | |
| PalPhenyl | Between Groups | .000 | 7 | .000 | 1.551 | .186 |
| | Within Groups | .000 | 32 | .000 | | |
| | Total | .000 | 39 | | | |
| StePhenyl | Between Groups | .000 | 7 | .000 | 1.264 | .299 |
| | Within Groups | .000 | 32 | .000 | | |
| | Total | .000 | 39 | | | |
| OlePhenyl | Between Groups | .000 | 7 | .000 | 3.348 | .009 |

| | | | | | | |
|---|---|---|---|---|---|---|
| | Within Groups | .000 | 32 | .000 | | |
| | Total | .000 | 39 | | | |
| LinPhenyl | Between Groups | .000 | 7 | .000 | 5.032 | <.001 |
| | Within Groups | .000 | 32 | .000 | | |
| | Total | .000 | 39 | | | |
| AraPhenyl | Between Groups | .000 | 7 | .000 | .751 | .632 |
| | Within Groups | .000 | 32 | .000 | | |
| | Total | .000 | 39 | | | |
| DocPhenyl | Between Groups | .000 | 7 | .000 | 1.943 | .095 |
| | Within Groups | .000 | 32 | .000 | | |
| | Total | .000 | 39 | | | |
| palAla | Between Groups | .000 | 7 | .000 | 1.332 | .268 |
| | Within Groups | .000 | 32 | .000 | | |
| | Total | .000 | 39 | | | |
| steAla | Between Groups | .000 | 7 | .000 | .591 | .758 |
| | Within Groups | .000 | 31 | .000 | | |
| | Total | .000 | 38 | | | |
| oleAla | Between Groups | .000 | 7 | .000 | 1.081 | .399 |
| | Within Groups | .000 | 30 | .000 | | |
| | Total | .000 | 37 | | | |
| linAla | Between Groups | .000 | 7 | .000 | .919 | .505 |
| | Within Groups | .000 | 32 | .000 | | |
| | Total | .000 | 39 | | | |

| | | | | | | |
|---|---|---|---|---|---|---|
| araAla | Between Groups | .000 | 6 | .000 | .806 | .606 |
| | Within Groups | .000 | 5 | .000 | | |
| | Total | .000 | 11 | | | |
| docAla | Between Groups | .000 | 5 | .000 | .342 | .879 |
| | Within Groups | .000 | 14 | .000 | | |
| | Total | .000 | 19 | | | |
| palGABA | Between Groups | .000 | 7 | .000 | .820 | .578 |
| | Within Groups | .000 | 32 | .000 | | |
| | Total | .000 | 39 | | | |
| steGABA | Between Groups | .000 | 7 | .000 | .380 | .907 |
| | Within Groups | .000 | 32 | .000 | | |
| | Total | .000 | 39 | | | |
| oleGABA | Between Groups | .000 | 7 | .000 | 2.601 | .031 |
| | Within Groups | .000 | 31 | .000 | | |
| | Total | .000 | 38 | | | |
| linGABA | Between Groups | .000 | 7 | .000 | 2.800 | .022 |
| | Within Groups | .000 | 31 | .000 | | |
| | Total | .000 | 38 | | | |
| araGABA | Between Groups | .000 | 7 | .000 | .742 | .639 |
| | Within Groups | .000 | 25 | .000 | | |
| | Total | .000 | 32 | | | |
| docGABA | Between Groups | .000 | 7 | .000 | .401 | .891 |
| | Within Groups | .000 | 21 | .000 | | |

| | | | | | | |
|---|---|---|---|---|---|---|
| | Total | .000 | 28 | | | |
| palMeth | Between Groups | .000 | 7 | .000 | 1.855 | .111 |
| | Within Groups | .000 | 32 | .000 | | |
| | Total | .000 | 39 | | | |
| steMeth | Between Groups | .000 | 7 | .000 | 2.390 | .047 |
| | Within Groups | .000 | 29 | .000 | | |
| | Total | .000 | 36 | | | |
| oleMeth | Between Groups | .000 | 7 | .000 | 3.269 | .010 |
| | Within Groups | .000 | 32 | .000 | | |
| | Total | .000 | 39 | | | |
| linMeth | Between Groups | .000 | 7 | .000 | 5.231 | <.001 |
| | Within Groups | .000 | 31 | .000 | | |
| | Total | .000 | 38 | | | |
| araMeth | Between Groups | .000 | 7 | .000 | .441 | .866 |
| | Within Groups | .000 | 23 | .000 | | |
| | Total | .000 | 30 | | | |
| docMeth | Between Groups | .000 | 7 | .000 | 1.317 | .279 |
| | Within Groups | .000 | 28 | .000 | | |
| | Total | .000 | 35 | | | |
| PGEtwo | Between Groups | .000 | 7 | .000 | 2.445 | .042 |
| | Within Groups | .000 | 29 | .000 | | |
| | Total | .000 | 36 | | | |
| PGFtwoA | Between Groups | .000 | 7 | .000 | 2.254 | .062 |

| | | | | | | |
|---|---|---|---|---|---|---|
| | Within Groups | .000 | 26 | .000 | | |
| | Total | .000 | 33 | | | |
| SixketoPGFOneA | Between Groups | .000 | 3 | .000 | 12.418 | .075 |
| | Within Groups | .000 | 2 | .000 | | |
| | Total | .000 | 5 | | | |
| palProline | Between Groups | .000 | 7 | .000 | 1.325 | .271 |
| | Within Groups | .000 | 32 | .000 | | |
| | Total | .000 | 39 | | | |
| steProline | Between Groups | .000 | 7 | .000 | 1.613 | .272 |
| | Within Groups | .000 | 7 | .000 | | |
| | Total | .000 | 14 | | | |
| oleProline | Between Groups | .000 | 7 | .000 | 2.816 | .021 |
| | Within Groups | .000 | 32 | .000 | | |
| | Total | .000 | 39 | | | |
| linProline | Between Groups | .000 | 7 | .000 | 1.092 | .409 |
| | Within Groups | .000 | 18 | .000 | | |
| | Total | .000 | 25 | | | |
| araProline | Between Groups | .000 | 7 | .000 | 1.063 | .416 |
| | Within Groups | .000 | 24 | .000 | | |
| | Total | .000 | 31 | | | |
| docProline | Between Groups | .000 | 7 | .000 | .480 | .840 |
| | Within Groups | .000 | 26 | .000 | | |
| | Total | .000 | 33 | | | |

| | | | | | | |
|---|---|---|---|---|---|---|
| palSerine | Between Groups | .000 | 7 | .000 | .778 | .611 |
| | Within Groups | .000 | 29 | .000 | | |
| | Total | .000 | 36 | | | |
| steSerine | Between Groups | .000 | 7 | .000 | .486 | .837 |
| | Within Groups | .000 | 31 | .000 | | |
| | Total | .000 | 38 | | | |
| oleSerine | Between Groups | .000 | 7 | .000 | 1.887 | .118 |
| | Within Groups | .000 | 23 | .000 | | |
| | Total | .000 | 30 | | | |
| linSerine | Between Groups | .000 | 7 | .000 | 1.706 | .167 |
| | Within Groups | .000 | 19 | .000 | | |
| | Total | .000 | 26 | | | |
| araSerine | Between Groups | .000 | 6 | .000 | 1.991 | .233 |
| | Within Groups | .000 | 5 | .000 | | |
| | Total | .000 | 11 | | | |
| docSerine | Between Groups | .000 | 7 | .000 | 5.459 | .008 |
| | Within Groups | .000 | 10 | .000 | | |
| | Total | .000 | 17 | | | |
| palTrypt | Between Groups | .000 | 7 | .000 | .486 | .834 |
| | Within Groups | .000 | 21 | .000 | | |
| | Total | .000 | 28 | | | |
| steTrypt | Between Groups | .000 | 7 | .000 | .290 | .952 |
| | Within Groups | .000 | 28 | .000 | | |

| | | | | | | |
|---|---|---|---|---|---|---|
| | Total | .000 | 35 | | | |
| oleTrypt | Between Groups | .000 | 7 | .000 | 1.794 | .154 |
| | Within Groups | .000 | 17 | .000 | | |
| | Total | .000 | 24 | | | |
| linTrypt | Between Groups | .000 | 7 | .000 | .719 | .658 |
| | Within Groups | .000 | 15 | .000 | | |
| | Total | .000 | 22 | | | |
| araTrypt | Between Groups | .000 | 3 | .000 | 10.503 | .088 |
| | Within Groups | .000 | 2 | .000 | | |
| | Total | .000 | 5 | | | |
| docTrypt | Between Groups | .000 | 7 | .000 | .244 | .959 |
| | Within Groups | .000 | 7 | .000 | | |
| | Total | .000 | 14 | | | |
| palTyr | Between Groups | .000 | 7 | .000 | 1.737 | .135 |
| | Within Groups | .000 | 32 | .000 | | |
| | Total | .000 | 39 | | | |
| steTyr | Between Groups | .000 | 7 | .000 | 1.070 | .405 |
| | Within Groups | .000 | 32 | .000 | | |
| | Total | .000 | 39 | | | |
| oleTyr | Between Groups | .000 | 7 | .000 | 2.708 | .025 |
| | Within Groups | .000 | 32 | .000 | | |
| | Total | .000 | 39 | | | |
| linTyr | Between Groups | .000 | 7 | .000 | 1.973 | .090 |

| | | | | | | |
|---|---|---|---|---|---|---|
| | Within Groups | .000 | 32 | .000 | | |
| | Total | .000 | 39 | | | |
| araTyr | Between Groups | .000 | 7 | .000 | .475 | .841 |
| | Within Groups | .000 | 20 | .000 | | |
| | Total | .000 | 27 | | | |
| docTyr | Between Groups | .000 | 7 | .000 | .116 | .996 |
| | Within Groups | .000 | 18 | .000 | | |
| | Total | .000 | 25 | | | |
| palVal | Between Groups | .000 | 7 | .000 | 1.278 | .293 |
| | Within Groups | .000 | 31 | .000 | | |
| | Total | .000 | 38 | | | |
| steVal | Between Groups | .000 | 7 | .000 | 1.423 | .234 |
| | Within Groups | .000 | 29 | .000 | | |
| | Total | .000 | 36 | | | |
| oleVal | Between Groups | .000 | 7 | .000 | 1.579 | .180 |
| | Within Groups | .000 | 30 | .000 | | |
| | Total | .000 | 37 | | | |
| linVal | Between Groups | .000 | 7 | .000 | 1.584 | .181 |
| | Within Groups | .000 | 28 | .000 | | |
| | Total | .000 | 35 | | | |
| docVal | Between Groups | .000 | 7 | .000 | 1.171 | .354 |
| | Within Groups | .000 | 25 | .000 | | |
| | Total | .000 | 32 | | | |

## ANOVA Effect Sizes[a,b]

| | | Point Estimate | 95% Confidence Interval | |
|---|---|---|---|---|
| | | | Lower | Upper |
| twoAG | Eta-squared | .468 | .086 | .562 |
| | Epsilon-squared | .351 | -.114 | .466 |
| | Omega-squared Fixed-effect | .346 | -.111 | .460 |
| | Omega-squared Random-effect | .070 | -.014 | .108 |
| twoLG | Eta-squared | .543 | .174 | .628 |
| | Epsilon-squared | .443 | -.007 | .546 |
| | Omega-squared Fixed-effect | .437 | -.007 | .540 |
| | Omega-squared Random-effect | .100 | -.001 | .144 |
| twoOG | Eta-squared | .198 | .000 | .284 |
| | Epsilon-squared | .022 | -.219 | .128 |
| | Omega-squared Fixed-effect | .021 | -.212 | .125 |
| | Omega-squared Random-effect | .003 | -.026 | .020 |
| twoPG | Eta-squared | .336 | .000 | .438 |
| | Epsilon-squared | .191 | -.219 | .315 |
| | Omega-squared Fixed-effect | .187 | -.212 | .310 |
| | Omega-squared Random-effect | .032 | -.026 | .060 |
| AA | Eta-squared | .496 | .116 | .587 |
| | Epsilon-squared | .385 | -.077 | .496 |
| | Omega-squared Fixed-effect | .380 | -.075 | .490 |
| | Omega-squared Random-effect | .080 | -.010 | .121 |
| OA | Eta-squared | .378 | .004 | .479 |
| | Epsilon-squared | .241 | -.214 | .365 |

| | | | | |
|---|---|---|---|---|
| | Omega-squared Fixed-effect | .237 | -.208 | .359 |
| | Omega-squared Random-effect | .042 | -.025 | .074 |
| EIC | Eta-squared | .330 | .000 | .432 |
| | Epsilon-squared | .183 | -.219 | .308 |
| | Omega-squared Fixed-effect | .180 | -.212 | .302 |
| | Omega-squared Random-effect | .030 | -.026 | .058 |
| DOC | Eta-squared | .338 | .000 | .440 |
| | Epsilon-squared | .193 | -.219 | .317 |
| | Omega-squared Fixed-effect | .189 | -.212 | .312 |
| | Omega-squared Random-effect | .032 | -.026 | .061 |
| LIN | Eta-squared | .459 | .077 | .554 |
| | Epsilon-squared | .341 | -.125 | .457 |
| | Omega-squared Fixed-effect | .335 | -.121 | .451 |
| | Omega-squared Random-effect | .067 | -.016 | .105 |
| NAGly | Eta-squared | .190 | .000 | .268 |
| | Epsilon-squared | -.028 | -.269 | .071 |
| | Omega-squared Fixed-effect | -.027 | -.259 | .069 |
| | Omega-squared Random-effect | -.004 | -.030 | .010 |
| LinGly | Eta-squared | .542 | .172 | .627 |
| | Epsilon-squared | .442 | -.009 | .545 |
| | Omega-squared Fixed-effect | .436 | -.009 | .539 |
| | Omega-squared Random-effect | .099 | -.001 | .143 |
| PalGly | Eta-squared | .303 | .000 | .405 |
| | Epsilon-squared | .151 | -.219 | .274 |
| | Omega-squared Fixed-effect | .147 | -.212 | .269 |

| | | | | |
|---|---|---|---|---|
| | Omega-squared Random-effect | .024 | -.026 | .050 |
| OlGly | Eta-squared | .365 | .000 | .467 |
| | Epsilon-squared | .226 | -.219 | .350 |
| | Omega-squared Fixed-effect | .222 | -.212 | .344 |
| | Omega-squared Random-effect | .039 | -.026 | .070 |
| DocGly | Eta-squared | .373 | .000 | .469 |
| | Epsilon-squared | .164 | -.333 | .292 |
| | Omega-squared Fixed-effect | .160 | -.318 | .285 |
| | Omega-squared Random-effect | .026 | -.036 | .054 |
| StrGly | Eta-squared | .262 | .000 | .360 |
| | Epsilon-squared | .100 | -.219 | .220 |
| | Omega-squared Fixed-effect | .098 | -.212 | .216 |
| | Omega-squared Random-effect | .015 | -.026 | .038 |
| PEA | Eta-squared | .144 | .000 | .210 |
| | Epsilon-squared | -.043 | -.219 | .038 |
| | Omega-squared Fixed-effect | -.042 | -.212 | .037 |
| | Omega-squared Random-effect | -.006 | -.026 | .005 |
| SEA | Eta-squared | .544 | .175 | .628 |
| | Epsilon-squared | .444 | -.006 | .547 |
| | Omega-squared Fixed-effect | .438 | -.006 | .541 |
| | Omega-squared Random-effect | .100 | -.001 | .144 |
| OEA | Eta-squared | .127 | .000 | .184 |
| | Epsilon-squared | -.064 | -.219 | .006 |
| | Omega-squared Fixed-effect | -.062 | -.212 | .006 |
| | Omega-squared Random-effect | -.008 | -.026 | .001 |

| | | | | |
|---|---|---|---|---|
| AEA | Eta-squared | .218 | .000 | .310 |
| | Epsilon-squared | .047 | -.219 | .159 |
| | Omega-squared Fixed-effect | .046 | -.212 | .155 |
| | Omega-squared Random-effect | .007 | -.026 | .026 |
| LEA | Eta-squared | .332 | .000 | .434 |
| | Epsilon-squared | .186 | -.219 | .310 |
| | Omega-squared Fixed-effect | .182 | -.212 | .305 |
| | Omega-squared Random-effect | .031 | -.026 | .059 |
| DEA | Eta-squared | .237 | .000 | .332 |
| | Epsilon-squared | .070 | -.219 | .186 |
| | Omega-squared Fixed-effect | .069 | -.212 | .183 |
| | Omega-squared Random-effect | .010 | -.026 | .031 |
| StrTaurine | Eta-squared | .323 | .000 | .423 |
| | Epsilon-squared | .147 | -.259 | .273 |
| | Omega-squared Fixed-effect | .143 | -.250 | .268 |
| | Omega-squared Random-effect | .023 | -.029 | .050 |
| OlTaurine | Eta-squared | .283 | .000 | .382 |
| | Epsilon-squared | .116 | -.233 | .238 |
| | Omega-squared Fixed-effect | .113 | -.226 | .234 |
| | Omega-squared Random-effect | .018 | -.027 | .042 |
| NATaurine | Eta-squared | .127 | .000 | .174 |
| | Epsilon-squared | -.099 | -.259 | -.040 |
| | Omega-squared Fixed-effect | -.096 | -.250 | -.038 |
| | Omega-squared Random-effect | -.013 | -.029 | -.005 |
| PalTaurine | Eta-squared | .367 | .000 | .469 |
| | Epsilon-squared | .229 | -.219 | .353 |

| | | | | |
|---|---|---|---|---|
| | Omega-squared Fixed-effect | .225 | -.212 | .347 |
| | Omega-squared Random-effect | .040 | -.026 | .071 |
| PalLeucine | Eta-squared | .226 | .000 | .319 |
| | Epsilon-squared | .057 | -.219 | .171 |
| | Omega-squared Fixed-effect | .056 | -.212 | .167 |
| | Omega-squared Random-effect | .008 | -.026 | .028 |
| LinLeucine | Eta-squared | .439 | .057 | .536 |
| | Epsilon-squared | .317 | -.149 | .435 |
| | Omega-squared Fixed-effect | .311 | -.145 | .429 |
| | Omega-squared Random-effect | .061 | -.018 | .097 |
| DocLeucine | Eta-squared | .267 | .000 | .366 |
| | Epsilon-squared | .107 | -.219 | .228 |
| | Omega-squared Fixed-effect | .105 | -.212 | .223 |
| | Omega-squared Random-effect | .016 | -.026 | .039 |
| StrLeucine | Eta-squared | .315 | .000 | .417 |
| | Epsilon-squared | .166 | -.219 | .290 |
| | Omega-squared Fixed-effect | .162 | -.212 | .285 |
| | Omega-squared Random-effect | .027 | -.026 | .054 |
| OlLeucine | Eta-squared | .213 | .000 | .303 |
| | Epsilon-squared | .041 | -.219 | .151 |
| | Omega-squared Fixed-effect | .040 | -.212 | .148 |
| | Omega-squared Random-effect | .006 | -.026 | .024 |
| PalPhenyl | Eta-squared | .253 | .000 | .351 |
| | Epsilon-squared | .090 | -.219 | .209 |
| | Omega-squared Fixed-effect | .088 | -.212 | .205 |

| | | | | |
|---|---|---|---|---|
| | Omega-squared Random-effect | .014 | -.026 | .035 |
| StePhenyl | Eta-squared | .217 | .000 | .308 |
| | Epsilon-squared | .045 | -.219 | .157 |
| | Omega-squared Fixed-effect | .044 | -.212 | .153 |
| | Omega-squared Random-effect | .007 | -.026 | .025 |
| OlePhenyl | Eta-squared | .423 | .042 | .521 |
| | Epsilon-squared | .296 | -.168 | .416 |
| | Omega-squared Fixed-effect | .291 | -.163 | .410 |
| | Omega-squared Random-effect | .055 | -.020 | .090 |
| LinPhenyl | Eta-squared | .524 | .150 | .611 |
| | Epsilon-squared | .420 | -.036 | .526 |
| | Omega-squared Fixed-effect | .414 | -.035 | .520 |
| | Omega-squared Random-effect | .092 | -.005 | .134 |
| AraPhenyl | Eta-squared | .141 | .000 | .206 |
| | Epsilon-squared | -.047 | -.219 | .032 |
| | Omega-squared Fixed-effect | -.046 | -.212 | .032 |
| | Omega-squared Random-effect | -.006 | -.026 | .005 |
| DocPhenyl | Eta-squared | .298 | .000 | .400 |
| | Epsilon-squared | .145 | -.219 | .268 |
| | Omega-squared Fixed-effect | .142 | -.212 | .263 |
| | Omega-squared Random-effect | .023 | -.026 | .049 |
| palAla | Eta-squared | .226 | .000 | .319 |
| | Epsilon-squared | .056 | -.219 | .170 |
| | Omega-squared Fixed-effect | .055 | -.212 | .166 |
| | Omega-squared Random-effect | .008 | -.026 | .028 |

| | | | | |
|---|---|---|---|---|
| steAla | Eta-squared | .118 | .000 | .166 |
| | Epsilon-squared | -.082 | -.226 | -.022 |
| | Omega-squared Fixed-effect | -.079 | -.219 | -.021 |
| | Omega-squared Random-effect | -.011 | -.026 | -.003 |
| oleAla | Eta-squared | .201 | .000 | .288 |
| | Epsilon-squared | .015 | -.233 | .121 |
| | Omega-squared Fixed-effect | .015 | -.226 | .119 |
| | Omega-squared Random-effect | .002 | -.027 | .019 |
| linAla | Eta-squared | .167 | .000 | .245 |
| | Epsilon-squared | -.015 | -.219 | .079 |
| | Omega-squared Fixed-effect | -.014 | -.212 | .077 |
| | Omega-squared Random-effect | -.002 | -.026 | .012 |
| araAla | Eta-squared | .492 | .000 | .535 |
| | Epsilon-squared | -.118 | -1.200 | -.022 |
| | Omega-squared Fixed-effect | -.107 | -1.000 | -.020 |
| | Omega-squared Random-effect | -.016 | -.091 | -.003 |
| docAla | Eta-squared | .109 | .000 | .178 |
| | Epsilon-squared | -.209 | -.357 | -.115 |
| | Omega-squared Fixed-effect | -.197 | -.333 | -.109 |
| | Omega-squared Random-effect | -.034 | -.053 | -.020 |
| palGABA | Eta-squared | .152 | .000 | .223 |
| | Epsilon-squared | -.033 | -.219 | .053 |
| | Omega-squared Fixed-effect | -.033 | -.212 | .051 |
| | Omega-squared Random-effect | -.005 | -.026 | .008 |
| steGABA | Eta-squared | .077 | .000 | .086 |
| | Epsilon-squared | -.125 | -.219 | -.114 |

| | | | | |
|---|---|---|---|---|
| | Omega-squared Fixed-effect | -.122 | -.212 | -.111 |
| | Omega-squared Random-effect | -.016 | -.026 | -.014 |
| oleGABA | Eta-squared | .370 | .000 | .471 |
| | Epsilon-squared | .228 | -.226 | .352 |
| | Omega-squared Fixed-effect | .223 | -.219 | .346 |
| | Omega-squared Random-effect | .039 | -.026 | .070 |
| linGABA | Eta-squared | .387 | .005 | .488 |
| | Epsilon-squared | .249 | -.220 | .372 |
| | Omega-squared Fixed-effect | .244 | -.213 | .366 |
| | Omega-squared Random-effect | .044 | -.026 | .076 |
| araGABA | Eta-squared | .172 | .000 | .241 |
| | Epsilon-squared | -.060 | -.280 | .028 |
| | Omega-squared Fixed-effect | -.058 | -.269 | .027 |
| | Omega-squared Random-effect | -.008 | -.031 | .004 |
| docGABA | Eta-squared | .118 | .000 | .132 |
| | Epsilon-squared | -.176 | -.333 | -.157 |
| | Omega-squared Fixed-effect | -.169 | -.318 | -.151 |
| | Omega-squared Random-effect | -.021 | -.036 | -.019 |
| palMeth | Eta-squared | .289 | .000 | .389 |
| | Epsilon-squared | .133 | -.219 | .256 |
| | Omega-squared Fixed-effect | .130 | -.212 | .251 |
| | Omega-squared Random-effect | .021 | -.026 | .046 |
| steMeth | Eta-squared | .366 | .000 | .467 |
| | Epsilon-squared | .213 | -.241 | .338 |
| | Omega-squared Fixed-effect | .208 | -.233 | .332 |

| | | | | |
|---|---|---|---|---|
| | Omega-squared Random-effect | .036 | -.028 | .066 |
| oleMeth | Eta-squared | .417 | .036 | .516 |
| | Epsilon-squared | .289 | -.174 | .410 |
| | Omega-squared Fixed-effect | .284 | -.169 | .404 |
| | Omega-squared Random-effect | .054 | -.021 | .088 |
| linMeth | Eta-squared | .542 | .163 | .626 |
| | Epsilon-squared | .438 | -.026 | .542 |
| | Omega-squared Fixed-effect | .432 | -.025 | .536 |
| | Omega-squared Random-effect | .098 | -.004 | .141 |
| araMeth | Eta-squared | .118 | .000 | .143 |
| | Epsilon-squared | -.150 | -.304 | -.117 |
| | Omega-squared Fixed-effect | -.144 | -.292 | -.113 |
| | Omega-squared Random-effect | -.018 | -.033 | -.015 |
| docMeth | Eta-squared | .248 | .000 | .342 |
| | Epsilon-squared | .060 | -.250 | .177 |
| | Omega-squared Fixed-effect | .058 | -.241 | .173 |
| | Omega-squared Random-effect | .009 | -.029 | .029 |
| PGEtwo | Eta-squared | .371 | .000 | .472 |
| | Epsilon-squared | .219 | -.241 | .345 |
| | Omega-squared Fixed-effect | .215 | -.233 | .338 |
| | Omega-squared Random-effect | .038 | -.028 | .068 |
| PGFtwoA | Eta-squared | .378 | .000 | .477 |
| | Epsilon-squared | .210 | -.269 | .337 |
| | Omega-squared Fixed-effect | .205 | -.259 | .330 |
| | Omega-squared Random-effect | .036 | -.030 | .066 |

| | | | | |
|---|---|---|---|---|
| SixketoPGFOneA | Eta-squared | .949 | .000 | .959 |
| | Epsilon-squared | .873 | -1.500 | .898 |
| | Omega-squared Fixed-effect | .851 | -1.000 | .881 |
| | Omega-squared Random-effect | .656 | -.200 | .711 |
| palProline | Eta-squared | .225 | .000 | .318 |
| | Epsilon-squared | .055 | -.219 | .169 |
| | Omega-squared Fixed-effect | .054 | -.212 | .165 |
| | Omega-squared Random-effect | .008 | -.026 | .027 |
| steProline | Eta-squared | .617 | .000 | .650 |
| | Epsilon-squared | .234 | -1.000 | .301 |
| | Omega-squared Fixed-effect | .222 | -.875 | .287 |
| | Omega-squared Random-effect | .039 | -.071 | .054 |
| oleProline | Eta-squared | .381 | .007 | .482 |
| | Epsilon-squared | .246 | -.211 | .369 |
| | Omega-squared Fixed-effect | .241 | -.204 | .363 |
| | Omega-squared Random-effect | .043 | -.025 | .075 |
| linProline | Eta-squared | .298 | .000 | .384 |
| | Epsilon-squared | .025 | -.389 | .145 |
| | Omega-squared Fixed-effect | .024 | -.368 | .140 |
| | Omega-squared Random-effect | .004 | -.040 | .023 |
| araProline | Eta-squared | .237 | .000 | .324 |
| | Epsilon-squared | .014 | -.292 | .127 |
| | Omega-squared Fixed-effect | .014 | -.280 | .124 |
| | Omega-squared Random-effect | .002 | -.032 | .020 |
| docProline | Eta-squared | .114 | .000 | .148 |
| | Epsilon-squared | -.124 | -.269 | -.082 |

| | | | | |
|---|---|---|---|---|
| | Omega-squared Fixed-effect | -.120 | -.259 | -.079 |
| | Omega-squared Random-effect | -.016 | -.030 | -.011 |
| palSerine | Eta-squared | .158 | .000 | .227 |
| | Epsilon-squared | -.045 | -.241 | .041 |
| | Omega-squared Fixed-effect | -.044 | -.233 | .040 |
| | Omega-squared Random-effect | -.006 | -.028 | .006 |
| steSerine | Eta-squared | .099 | .000 | .131 |
| | Epsilon-squared | -.105 | -.226 | -.065 |
| | Omega-squared Fixed-effect | -.102 | -.219 | -.063 |
| | Omega-squared Random-effect | -.013 | -.026 | -.009 |
| oleSerine | Eta-squared | .365 | .000 | .463 |
| | Epsilon-squared | .171 | -.304 | .299 |
| | Omega-squared Fixed-effect | .167 | -.292 | .292 |
| | Omega-squared Random-effect | .028 | -.033 | .056 |
| linSerine | Eta-squared | .386 | .000 | .479 |
| | Epsilon-squared | .160 | -.368 | .287 |
| | Omega-squared Fixed-effect | .155 | -.350 | .279 |
| | Omega-squared Random-effect | .025 | -.038 | .053 |
| araSerine | Eta-squared | .705 | .000 | .735 |
| | Epsilon-squared | .351 | -1.200 | .417 |
| | Omega-squared Fixed-effect | .331 | -1.000 | .396 |
| | Omega-squared Random-effect | .076 | -.091 | .099 |
| docSerine | Eta-squared | .793 | .136 | .821 |
| | Epsilon-squared | .647 | -.469 | .696 |
| | Omega-squared Fixed-effect | .634 | -.431 | .684 |

| | | | | |
|---|---|---|---|---|
| | Omega-squared Random-effect | .199 | -.045 | .236 |
| palTrypt | Eta-squared | .139 | .000 | .175 |
| | Epsilon-squared | -.147 | -.333 | -.100 |
| | Omega-squared Fixed-effect | -.142 | -.318 | -.097 |
| | Omega-squared Random-effect | -.018 | -.036 | -.013 |
| steTrypt | Eta-squared | .068 | .000 | .046 |
| | Epsilon-squared | -.165 | -.250 | -.193 |
| | Omega-squared Fixed-effect | -.160 | -.241 | -.187 |
| | Omega-squared Random-effect | -.020 | -.029 | -.023 |
| oleTrypt | Eta-squared | .425 | .000 | .514 |
| | Epsilon-squared | .188 | -.412 | .313 |
| | Omega-squared Fixed-effect | .182 | -.389 | .305 |
| | Omega-squared Random-effect | .031 | -.042 | .059 |
| linTrypt | Eta-squared | .251 | .000 | .316 |
| | Epsilon-squared | -.098 | -.467 | -.003 |
| | Omega-squared Fixed-effect | -.093 | -.438 | -.003 |
| | Omega-squared Random-effect | -.012 | -.045 | .000 |
| araTrypt | Eta-squared | .940 | .000 | .953 |
| | Epsilon-squared | .851 | -1.500 | .881 |
| | Omega-squared Fixed-effect | .826 | -1.000 | .861 |
| | Omega-squared Random-effect | .613 | -.200 | .674 |
| docTrypt | Eta-squared | .196 | .000 | .087 |
| | Epsilon-squared | -.607 | -1.000 | -.826 |
| | Omega-squared Fixed-effect | -.545 | -.875 | -.731 |
| | Omega-squared Random-effect | -.053 | -.071 | -.064 |

| | | | | |
|---|---|---|---|---|
| palTyr | Eta-squared | .275 | .000 | .375 |
| | Epsilon-squared | .117 | -.219 | .238 |
| | Omega-squared Fixed-effect | .114 | -.212 | .234 |
| | Omega-squared Random-effect | .018 | -.026 | .042 |
| steTyr | Eta-squared | .190 | .000 | .274 |
| | Epsilon-squared | .012 | -.219 | .116 |
| | Omega-squared Fixed-effect | .012 | -.212 | .113 |
| | Omega-squared Random-effect | .002 | -.026 | .018 |
| oleTyr | Eta-squared | .372 | .000 | .473 |
| | Epsilon-squared | .235 | -.219 | .358 |
| | Omega-squared Fixed-effect | .230 | -.212 | .352 |
| | Omega-squared Random-effect | .041 | -.026 | .072 |
| linTyr | Eta-squared | .302 | .000 | .403 |
| | Epsilon-squared | .149 | -.219 | .272 |
| | Omega-squared Fixed-effect | .146 | -.212 | .267 |
| | Omega-squared Random-effect | .024 | -.026 | .050 |
| araTyr | Eta-squared | .143 | .000 | .175 |
| | Epsilon-squared | -.157 | -.350 | -.113 |
| | Omega-squared Fixed-effect | -.151 | -.333 | -.109 |
| | Omega-squared Random-effect | -.019 | -.037 | -.014 |
| docTyr | Eta-squared | .043 | .000 | .000 |
| | Epsilon-squared | -.329 | -.389 | -.389 |
| | Omega-squared Fixed-effect | -.312 | -.368 | -.368 |
| | Omega-squared Random-effect | -.035 | -.040 | -.040 |
| palVal | Eta-squared | .224 | .000 | .316 |
| | Epsilon-squared | .049 | -.226 | .162 |

| | | | | |
|---|---|---|---|---|
| | Omega-squared Fixed-effect | .048 | -.219 | .158 |
| | Omega-squared Random-effect | .007 | -.026 | .026 |
| steVal | Eta-squared | .256 | .000 | .352 |
| | Epsilon-squared | .076 | -.241 | .195 |
| | Omega-squared Fixed-effect | .074 | -.233 | .191 |
| | Omega-squared Random-effect | .011 | -.028 | .033 |
| oleVal | Eta-squared | .269 | .000 | .368 |
| | Epsilon-squared | .099 | -.233 | .220 |
| | Omega-squared Fixed-effect | .096 | -.226 | .216 |
| | Omega-squared Random-effect | .015 | -.027 | .038 |
| linVal | Eta-squared | .284 | .000 | .382 |
| | Epsilon-squared | .105 | -.250 | .228 |
| | Omega-squared Fixed-effect | .102 | -.241 | .223 |
| | Omega-squared Random-effect | .016 | -.029 | .039 |
| docVal | Eta-squared | .247 | .000 | .338 |
| | Epsilon-squared | .036 | -.280 | .153 |
| | Omega-squared Fixed-effect | .035 | -.269 | .149 |
| | Omega-squared Random-effect | .005 | -.031 | .024 |

a. Eta-squared and Epsilon-squared are estimated based on the fixed-effect model.

b. Negative but less biased estimates are retained, not rounded to zero.

## Post Hoc Tests

## Multiple Comparisons

LSD

| Dependent Variable | (I) Treatment | (J) Treatment | Mean Difference (I-J) | Std. Error | Sig. | 95% Confidence Interval Lower Bound | 95% Confidence Interval Upper Bound |
|---|---|---|---|---|---|---|---|
| twoAG | BFHDay1 | PFHDay1 | -2.66076E-012 | 1.01618E-011 | .795 | -2.3360E-011 | 1.8038E-011 |
| | | BMHDay1 | 3.74832E-011* | 1.01618E-011 | <.001 | 1.6784E-011 | 5.8182E-011 |
| | | PMHDay1 | 3.24254E-011* | 1.01618E-011 | .003 | 1.1727E-011 | 5.3124E-011 |
| | | BFHDay2 | 1.37363E-011 | 1.01618E-011 | .186 | -6.9626E-012 | 3.4435E-011 |
| | | PFHDay2 | 9.76646E-012 | 1.01618E-011 | .344 | -1.0932E-011 | 3.0465E-011 |
| | | BMHDay2 | 2.46489E-011* | 1.01618E-011 | .021 | 3.9500E-012 | 4.5348E-011 |
| | | PMHDay2 | 1.64572E-011 | 1.01618E-011 | .115 | -4.2416E-012 | 3.7156E-011 |
| | PFHDay1 | BFHDay1 | 2.66076E-012 | 1.01618E-011 | .795 | -1.8038E-011 | 2.3360E-011 |
| | | BMHDay1 | 4.01440E-011* | 1.01618E-011 | <.001 | 1.9445E-011 | 6.0843E-011 |
| | | PMHDay1 | 3.50862E-011* | 1.01618E-011 | .002 | 1.4387E-011 | 5.5785E-011 |
| | | BFHDay2 | 1.63970E-011 | 1.01618E-011 | .116 | -4.3018E-012 | 3.7096E-011 |
| | | PFHDay2 | 1.24272E-011 | 1.01618E-011 | .230 | -8.2716E-012 | 3.3126E-011 |
| | | BMHDay2 | 2.73096E-011* | 1.01618E-011 | .011 | 6.6108E-012 | 4.8008E-011 |
| | | PMHDay2 | 1.91180E-011 | 1.01618E-011 | .069 | -1.5808E-012 | 3.9817E-011 |
| | BMHDay1 | BFHDay1 | -3.74832E-011* | 1.01618E-011 | <.001 | -5.8182E-011 | -1.6784E-011 |
| | | PFHDay1 | -4.01440E-011* | 1.01618E-011 | <.001 | -6.0843E-011 | -1.9445E-011 |

| | | | | | | |
|---|---|---|---|---|---|---|
| | PMHDay1 | -5.05782E-012 | 1.01618E-011 | .622 | -2.5757E-011 | 1.5641E-011 |
| | BFHDay2 | -2.37470E-011* | 1.01618E-011 | .026 | -4.4446E-011 | -3.0481E-012 |
| | PFHDay2 | -2.77168E-011* | 1.01618E-011 | .010 | -4.8416E-011 | -7.0179E-012 |
| | BMHDay2 | -1.28344E-011 | 1.01618E-011 | .216 | -3.3533E-011 | 7.8645E-012 |
| | PMHDay2 | -2.10260E-011* | 1.01618E-011 | .047 | -4.1725E-011 | -3.2714E-013 |
| PMHDay1 | BFHDay1 | -3.24254E-011* | 1.01618E-011 | .003 | -5.3124E-011 | -1.1727E-011 |
| | PFHDay1 | -3.50862E-011* | 1.01618E-011 | .002 | -5.5785E-011 | -1.4387E-011 |
| | BMHDay1 | 5.05782E-012 | 1.01618E-011 | .622 | -1.5641E-011 | 2.5757E-011 |
| | BFHDay2 | -1.86891E-011 | 1.01618E-011 | .075 | -3.9388E-011 | 2.0097E-012 |
| | PFHDay2 | -2.26589E-011* | 1.01618E-011 | .033 | -4.3358E-011 | -1.9601E-012 |
| | BMHDay2 | -7.77654E-012 | 1.01618E-011 | .450 | -2.8475E-011 | 1.2922E-011 |
| | PMHDay2 | -1.59682E-011 | 1.01618E-011 | .126 | -3.6667E-011 | 4.7307E-012 |
| BFHDay2 | BFHDay1 | -1.37363E-011 | 1.01618E-011 | .186 | -3.4435E-011 | 6.9626E-012 |
| | PFHDay1 | -1.63970E-011 | 1.01618E-011 | .116 | -3.7096E-011 | 4.3018E-012 |
| | BMHDay1 | 2.37470E-011* | 1.01618E-011 | .026 | 3.0481E-012 | 4.4446E-011 |
| | PMHDay1 | 1.86891E-011 | 1.01618E-011 | .075 | -2.0097E-012 | 3.9388E-011 |
| | PFHDay2 | -3.96980E-012 | 1.01618E-011 | .699 | -2.4669E-011 | 1.6729E-011 |
| | BMHDay2 | 1.09126E-011 | 1.01618E-011 | .291 | -9.7862E-012 | 3.1611E-011 |
| | PMHDay2 | 2.72098E-012 | 1.01618E-011 | .791 | -1.7978E-011 | 2.3420E-011 |

| | | | | | | |
|---|---|---|---|---|---|---|
| | PFHDay2 | BFHDay1 | -9.76646E-012 | 1.01618E-011 | .344 | -3.0465E-011 | 1.0932E-011 |
| | | PFHDay1 | -1.24272E-011 | 1.01618E-011 | .230 | -3.3126E-011 | 8.2716E-012 |
| | | BMHDay1 | 2.77168E-011* | 1.01618E-011 | .010 | 7.0179E-012 | 4.8416E-011 |
| | | PMHDay1 | 2.26589E-011* | 1.01618E-011 | .033 | 1.9601E-012 | 4.3358E-011 |
| | | BFHDay2 | 3.96980E-012 | 1.01618E-011 | .699 | -1.6729E-011 | 2.4669E-011 |
| | | BMHDay2 | 1.48824E-011 | 1.01618E-011 | .153 | -5.8164E-012 | 3.5581E-011 |
| | | PMHDay2 | 6.69078E-012 | 1.01618E-011 | .515 | -1.4008E-011 | 2.7390E-011 |
| | BMHDay2 | BFHDay1 | -2.46489E-011* | 1.01618E-011 | .021 | -4.5348E-011 | -3.9500E-012 |
| | | PFHDay1 | -2.73096E-011* | 1.01618E-011 | .011 | -4.8008E-011 | -6.6108E-012 |
| | | BMHDay1 | 1.28344E-011 | 1.01618E-011 | .216 | -7.8645E-012 | 3.3533E-011 |
| | | PMHDay1 | 7.77654E-012 | 1.01618E-011 | .450 | -1.2922E-011 | 2.8475E-011 |
| | | BFHDay2 | -1.09126E-011 | 1.01618E-011 | .291 | -3.1611E-011 | 9.7862E-012 |
| | | PFHDay2 | -1.48824E-011 | 1.01618E-011 | .153 | -3.5581E-011 | 5.8164E-012 |
| | | PMHDay2 | -8.19162E-012 | 1.01618E-011 | .426 | -2.8890E-011 | 1.2507E-011 |
| | PMHDay2 | BFHDay1 | -1.64572E-011 | 1.01618E-011 | .115 | -3.7156E-011 | 4.2416E-012 |
| | | PFHDay1 | -1.91180E-011 | 1.01618E-011 | .069 | -3.9817E-011 | 1.5808E-012 |
| | | BMHDay1 | 2.10260E-011* | 1.01618E-011 | .047 | 3.2714E-013 | 4.1725E-011 |
| | | PMHDay1 | 1.59682E-011 | 1.01618E-011 | .126 | -4.7307E-012 | 3.6667E-011 |
| | | BFHDay2 | -2.72098E-012 | 1.01618E-011 | .791 | -2.3420E-011 | 1.7978E-011 |

| | | | | | | | |
|---|---|---|---|---|---|---|---|
| | | PFHDay2 | -6.69078E-012 | 1.01618E-011 | .515 | -2.7390E-011 | 1.4008E-011 |
| | | BMHDay2 | 8.19162E-012 | 1.01618E-011 | .426 | -1.2507E-011 | 2.8890E-011 |
| twoLG | BFHDay1 | PFHDay1 | 6.64344E-010 | 1.30688E-009 | .615 | -1.9977E-009 | 3.3264E-009 |
| | | BMHDay1 | 2.59840E-009 | 1.30688E-009 | .055 | -6.3635E-011 | 5.2604E-009 |
| | | PMHDay1 | 1.19998E-009 | 1.30688E-009 | .365 | -1.4621E-009 | 3.8620E-009 |
| | | BFHDay2 | 7.88696E-010 | 1.30688E-009 | .550 | -1.8733E-009 | 3.4507E-009 |
| | | PFHDay2 | 4.22784E-010 | 1.30688E-009 | .748 | -2.2392E-009 | 3.0848E-009 |
| | | BMHDay2 | -9.11642E-010 | 1.30688E-009 | .490 | -3.5737E-009 | 1.7504E-009 |
| | | PMHDay2 | -4.71352E-009* | 1.30688E-009 | .001 | -7.3755E-009 | -2.0515E-009 |
| | PFHDay1 | BFHDay1 | -6.64344E-010 | 1.30688E-009 | .615 | -3.3264E-009 | 1.9977E-009 |
| | | BMHDay1 | 1.93405E-009 | 1.30688E-009 | .149 | -7.2798E-010 | 4.5961E-009 |
| | | PMHDay1 | 5.35632E-010 | 1.30688E-009 | .685 | -2.1264E-009 | 3.1977E-009 |
| | | BFHDay2 | 1.24352E-010 | 1.30688E-009 | .925 | -2.5377E-009 | 2.7864E-009 |
| | | PFHDay2 | -2.41560E-010 | 1.30688E-009 | .855 | -2.9036E-009 | 2.4205E-009 |
| | | BMHDay2 | -1.57599E-009 | 1.30688E-009 | .237 | -4.2380E-009 | 1.0860E-009 |
| | | PMHDay2 | -5.37786E-009* | 1.30688E-009 | <.001 | -8.0399E-009 | -2.7158E-009 |
| | BMHDay1 | BFHDay1 | -2.59840E-009 | 1.30688E-009 | .055 | -5.2604E-009 | 6.3635E-011 |
| | | PFHDay1 | -1.93405E-009 | 1.30688E-009 | .149 | -4.5961E-009 | 7.2798E-010 |
| | | PMHDay1 | -1.39842E-009 | 1.30688E-009 | .293 | -4.0605E-009 | 1.2636E-009 |

| | | | | | | | |
|---|---|---|---|---|---|---|---|
| | | BFHDay2 | -1.80970E-009 | 1.30688E-009 | .176 | -4.4717E-009 | 8.5233E-010 |
| | | PFHDay2 | -2.17561E-009 | 1.30688E-009 | .106 | -4.8376E-009 | 4.8642E-010 |
| | | BMHDay2 | -3.51004E-009* | 1.30688E-009 | .011 | -6.1721E-009 | -8.4801E-010 |
| | | PMHDay2 | -7.31191E-009* | 1.30688E-009 | <.001 | -9.9739E-009 | -4.6499E-009 |
| | PMHDay1 | BFHDay1 | -1.19998E-009 | 1.30688E-009 | .365 | -3.8620E-009 | 1.4621E-009 |
| | | PFHDay1 | -5.35632E-010 | 1.30688E-009 | .685 | -3.1977E-009 | 2.1264E-009 |
| | | BMHDay1 | 1.39842E-009 | 1.30688E-009 | .293 | -1.2636E-009 | 4.0605E-009 |
| | | BFHDay2 | -4.11280E-010 | 1.30688E-009 | .755 | -3.0733E-009 | 2.2508E-009 |
| | | PFHDay2 | -7.77192E-010 | 1.30688E-009 | .556 | -3.4392E-009 | 1.8848E-009 |
| | | BMHDay2 | -2.11162E-009 | 1.30688E-009 | .116 | -4.7737E-009 | 5.5042E-010 |
| | | PMHDay2 | -5.91349E-009* | 1.30688E-009 | <.001 | -8.5755E-009 | -3.2515E-009 |
| | BFHDay2 | BFHDay1 | -7.88696E-010 | 1.30688E-009 | .550 | -3.4507E-009 | 1.8733E-009 |
| | | PFHDay1 | -1.24352E-010 | 1.30688E-009 | .925 | -2.7864E-009 | 2.5377E-009 |
| | | BMHDay1 | 1.80970E-009 | 1.30688E-009 | .176 | -8.5233E-010 | 4.4717E-009 |
| | | PMHDay1 | 4.11280E-010 | 1.30688E-009 | .755 | -2.2508E-009 | 3.0733E-009 |
| | | PFHDay2 | -3.65912E-010 | 1.30688E-009 | .781 | -3.0279E-009 | 2.2961E-009 |
| | | BMHDay2 | -1.70034E-009 | 1.30688E-009 | .203 | -4.3624E-009 | 9.6170E-010 |
| | | PMHDay2 | -5.50221E-009* | 1.30688E-009 | <.001 | -8.1642E-009 | -2.8402E-009 |
| | PFHDay2 | BFHDay1 | -4.22784E-010 | 1.30688E-009 | .748 | -3.0848E-009 | 2.2392E-009 |

| | | | | | | |
|---|---|---|---|---|---|---|
| | PFHDay1 | 2.41560E-010 | 1.30688E-009 | .855 | -2.4205E-009 | 2.9036E-009 |
| | BMHDay1 | 2.17561E-009 | 1.30688E-009 | .106 | -4.8642E-010 | 4.8376E-009 |
| | PMHDay1 | 7.77192E-010 | 1.30688E-009 | .556 | -1.8848E-009 | 3.4392E-009 |
| | BFHDay2 | 3.65912E-010 | 1.30688E-009 | .781 | -2.2961E-009 | 3.0279E-009 |
| | BMHDay2 | -1.33443E-009 | 1.30688E-009 | .315 | -3.9965E-009 | 1.3276E-009 |
| | PMHDay2 | -5.13630E-009* | 1.30688E-009 | <.001 | -7.7983E-009 | -2.4743E-009 |
| BMHDay2 | BFHDay1 | 9.11642E-010 | 1.30688E-009 | .490 | -1.7504E-009 | 3.5737E-009 |
| | PFHDay1 | 1.57599E-009 | 1.30688E-009 | .237 | -1.0860E-009 | 4.2380E-009 |
| | BMHDay1 | 3.51004E-009* | 1.30688E-009 | .011 | 8.4801E-010 | 6.1721E-009 |
| | PMHDay1 | 2.11162E-009 | 1.30688E-009 | .116 | -5.5042E-010 | 4.7737E-009 |
| | BFHDay2 | 1.70034E-009 | 1.30688E-009 | .203 | -9.6170E-010 | 4.3624E-009 |
| | PFHDay2 | 1.33443E-009 | 1.30688E-009 | .315 | -1.3276E-009 | 3.9965E-009 |
| | PMHDay2 | -3.80187E-009* | 1.30688E-009 | .007 | -6.4639E-009 | -1.1398E-009 |
| PMHDay2 | BFHDay1 | 4.71352E-009* | 1.30688E-009 | .001 | 2.0515E-009 | 7.3755E-009 |
| | PFHDay1 | 5.37786E-009* | 1.30688E-009 | <.001 | 2.7158E-009 | 8.0399E-009 |
| | BMHDay1 | 7.31191E-009* | 1.30688E-009 | <.001 | 4.6499E-009 | 9.9739E-009 |
| | PMHDay1 | 5.91349E-009* | 1.30688E-009 | <.001 | 3.2515E-009 | 8.5755E-009 |
| | BFHDay2 | 5.50221E-009* | 1.30688E-009 | <.001 | 2.8402E-009 | 8.1642E-009 |
| | PFHDay2 | 5.13630E-009* | 1.30688E-009 | <.001 | 2.4743E-009 | 7.7983E-009 |

| | | | | | | | |
|---|---|---|---|---|---|---|---|
| | | BMHDay2 | 3.80187E-009* | 1.30688E-009 | .007 | 1.1398E-009 | 6.4639E-009 |
| twoOG | BFHDay1 | PFHDay1 | 2.78106E-009 | 2.85466E-009 | .337 | -3.0337E-009 | 8.5958E-009 |
| | | BMHDay1 | 5.08520E-009 | 2.85466E-009 | .084 | -7.2956E-010 | 1.0900E-008 |
| | | PMHDay1 | 3.91551E-009 | 2.85466E-009 | .180 | -1.8992E-009 | 9.7303E-009 |
| | | BFHDay2 | 4.35370E-009 | 2.85466E-009 | .137 | -1.4611E-009 | 1.0168E-008 |
| | | PFHDay2 | 2.62471E-009 | 2.85466E-009 | .365 | -3.1900E-009 | 8.4395E-009 |
| | | BMHDay2 | 3.37468E-009 | 2.85466E-009 | .246 | -2.4401E-009 | 9.1894E-009 |
| | | PMHDay2 | -1.10574E-009 | 2.85466E-009 | .701 | -6.9205E-009 | 4.7090E-009 |
| | PFHDay1 | BFHDay1 | -2.78106E-009 | 2.85466E-009 | .337 | -8.5958E-009 | 3.0337E-009 |
| | | BMHDay1 | 2.30414E-009 | 2.85466E-009 | .426 | -3.5106E-009 | 8.1189E-009 |
| | | PMHDay1 | 1.13445E-009 | 2.85466E-009 | .694 | -4.6803E-009 | 6.9492E-009 |
| | | BFHDay2 | 1.57264E-009 | 2.85466E-009 | .586 | -4.2421E-009 | 7.3874E-009 |
| | | PFHDay2 | -1.56352E-010 | 2.85466E-009 | .957 | -5.9711E-009 | 5.6584E-009 |
| | | BMHDay2 | 5.93620E-010 | 2.85466E-009 | .837 | -5.2211E-009 | 6.4084E-009 |
| | | PMHDay2 | -3.88680E-009 | 2.85466E-009 | .183 | -9.7016E-009 | 1.9280E-009 |
| | BMHDay1 | BFHDay1 | -5.08520E-009 | 2.85466E-009 | .084 | -1.0900E-008 | 7.2956E-010 |
| | | PFHDay1 | -2.30414E-009 | 2.85466E-009 | .426 | -8.1189E-009 | 3.5106E-009 |
| | | PMHDay1 | -1.16969E-009 | 2.85466E-009 | .685 | -6.9844E-009 | 4.6451E-009 |
| | | BFHDay2 | -7.31503E-010 | 2.85466E-009 | .799 | -6.5463E-009 | 5.0833E-009 |

| | | | | | | |
|---|---|---|---|---|---|---|
| | PFHDay2 | -2.46049E-009 | 2.85466E-009 | .395 | -8.2752E-009 | 3.3543E-009 |
| | BMHDay2 | -1.71052E-009 | 2.85466E-009 | .553 | -7.5253E-009 | 4.1042E-009 |
| | PMHDay2 | -6.19094E-009* | 2.85466E-009 | .038 | -1.2006E-008 | -3.7619E-010 |
| PMHDay1 | BFHDay1 | -3.91551E-009 | 2.85466E-009 | .180 | -9.7303E-009 | 1.8992E-009 |
| | PFHDay1 | -1.13445E-009 | 2.85466E-009 | .694 | -6.9492E-009 | 4.6803E-009 |
| | BMHDay1 | 1.16969E-009 | 2.85466E-009 | .685 | -4.6451E-009 | 6.9844E-009 |
| | BFHDay2 | 4.38188E-010 | 2.85466E-009 | .879 | -5.3766E-009 | 6.2529E-009 |
| | PFHDay2 | -1.29080E-009 | 2.85466E-009 | .654 | -7.1056E-009 | 4.5240E-009 |
| | BMHDay2 | -5.40828E-010 | 2.85466E-009 | .851 | -6.3556E-009 | 5.2739E-009 |
| | PMHDay2 | -5.02125E-009 | 2.85466E-009 | .088 | -1.0836E-008 | 7.9350E-010 |
| BFHDay2 | BFHDay1 | -4.35370E-009 | 2.85466E-009 | .137 | -1.0168E-008 | 1.4611E-009 |
| | PFHDay1 | -1.57264E-009 | 2.85466E-009 | .586 | -7.3874E-009 | 4.2421E-009 |
| | BMHDay1 | 7.31503E-010 | 2.85466E-009 | .799 | -5.0833E-009 | 6.5463E-009 |
| | PMHDay1 | -4.38188E-010 | 2.85466E-009 | .879 | -6.2529E-009 | 5.3766E-009 |
| | PFHDay2 | -1.72899E-009 | 2.85466E-009 | .549 | -7.5437E-009 | 4.0858E-009 |
| | BMHDay2 | -9.79016E-010 | 2.85466E-009 | .734 | -6.7938E-009 | 4.8357E-009 |
| | PMHDay2 | -5.45944E-009 | 2.85466E-009 | .065 | -1.1274E-008 | 3.5531E-010 |
| PFHDay2 | BFHDay1 | -2.62471E-009 | 2.85466E-009 | .365 | -8.4395E-009 | 3.1900E-009 |
| | PFHDay1 | 1.56352E-010 | 2.85466E-009 | .957 | -5.6584E-009 | 5.9711E-009 |

| | | | | | | |
|---|---|---|---|---|---|---|
| | BMHDay1 | 2.46049E-009 | 2.85466E-009 | .395 | -3.3543E-009 | 8.2752E-009 |
| | PMHDay1 | 1.29080E-009 | 2.85466E-009 | .654 | -4.5240E-009 | 7.1056E-009 |
| | BFHDay2 | 1.72899E-009 | 2.85466E-009 | .549 | -4.0858E-009 | 7.5437E-009 |
| | BMHDay2 | 7.49972E-010 | 2.85466E-009 | .794 | -5.0648E-009 | 6.5647E-009 |
| | PMHDay2 | -3.73045E-009 | 2.85466E-009 | .201 | -9.5452E-009 | 2.0843E-009 |
| BMHDay2 | BFHDay1 | -3.37468E-009 | 2.85466E-009 | .246 | -9.1894E-009 | 2.4401E-009 |
| | PFHDay1 | -5.93620E-010 | 2.85466E-009 | .837 | -6.4084E-009 | 5.2211E-009 |
| | BMHDay1 | 1.71052E-009 | 2.85466E-009 | .553 | -4.1042E-009 | 7.5253E-009 |
| | PMHDay1 | 5.40828E-010 | 2.85466E-009 | .851 | -5.2739E-009 | 6.3556E-009 |
| | BFHDay2 | 9.79016E-010 | 2.85466E-009 | .734 | -4.8357E-009 | 6.7938E-009 |
| | PFHDay2 | -7.49972E-010 | 2.85466E-009 | .794 | -6.5647E-009 | 5.0648E-009 |
| | PMHDay2 | -4.48042E-009 | 2.85466E-009 | .126 | -1.0295E-008 | 1.3343E-009 |
| PMHDay2 | BFHDay1 | 1.10574E-009 | 2.85466E-009 | .701 | -4.7090E-009 | 6.9205E-009 |
| | PFHDay1 | 3.88680E-009 | 2.85466E-009 | .183 | -1.9280E-009 | 9.7016E-009 |
| | BMHDay1 | 6.19094E-009* | 2.85466E-009 | .038 | 3.7619E-010 | 1.2006E-008 |
| | PMHDay1 | 5.02125E-009 | 2.85466E-009 | .088 | -7.9350E-010 | 1.0836E-008 |
| | BFHDay2 | 5.45944E-009 | 2.85466E-009 | .065 | -3.5531E-010 | 1.1274E-008 |
| | PFHDay2 | 3.73045E-009 | 2.85466E-009 | .201 | -2.0843E-009 | 9.5452E-009 |
| | BMHDay2 | 4.48042E-009 | 2.85466E-009 | .126 | -1.3343E-009 | 1.0295E-008 |

| | | | | | | | |
|---|---|---|---|---|---|---|---|
| twoPG | BFHDay1 | PFHDay1 | -4.28141E-011* | 1.64451E-011 | .014 | -7.6312E-011 | -9.3165E-012 |
| | | BMHDay1 | 5.43520E-014 | 1.64451E-011 | .997 | -3.3443E-011 | 3.3552E-011 |
| | | PMHDay1 | 1.25036E-012 | 1.64451E-011 | .940 | -3.2247E-011 | 3.4748E-011 |
| | | BFHDay2 | -2.87620E-013 | 1.64451E-011 | .986 | -3.3785E-011 | 3.3210E-011 |
| | | PFHDay2 | 1.65123E-011 | 1.64451E-011 | .323 | -1.6985E-011 | 5.0010E-011 |
| | | BMHDay2 | 1.46598E-012 | 1.64451E-011 | .930 | -3.2032E-011 | 3.4964E-011 |
| | | PMHDay2 | 1.01330E-011 | 1.64451E-011 | .542 | -2.3365E-011 | 4.3631E-011 |
| | PFHDay1 | BFHDay1 | 4.28141E-011* | 1.64451E-011 | .014 | 9.3165E-012 | 7.6312E-011 |
| | | BMHDay1 | 4.28685E-011* | 1.64451E-011 | .014 | 9.3709E-012 | 7.6366E-011 |
| | | PMHDay1 | 4.40645E-011* | 1.64451E-011 | .012 | 1.0567E-011 | 7.7562E-011 |
| | | BFHDay2 | 4.25265E-011* | 1.64451E-011 | .014 | 9.0289E-012 | 7.6024E-011 |
| | | PFHDay2 | 5.93264E-011* | 1.64451E-011 | .001 | 2.5829E-011 | 9.2824E-011 |
| | | BMHDay2 | 4.42801E-011* | 1.64451E-011 | .011 | 1.0783E-011 | 7.7778E-011 |
| | | PMHDay2 | 5.29471E-011* | 1.64451E-011 | .003 | 1.9450E-011 | 8.6445E-011 |
| | BMHDay1 | BFHDay1 | -5.43520E-014 | 1.64451E-011 | .997 | -3.3552E-011 | 3.3443E-011 |
| | | PFHDay1 | -4.28685E-011* | 1.64451E-011 | .014 | -7.6366E-011 | -9.3709E-012 |
| | | PMHDay1 | 1.19601E-012 | 1.64451E-011 | .942 | -3.2302E-011 | 3.4694E-011 |
| | | BFHDay2 | -3.41972E-013 | 1.64451E-011 | .984 | -3.3840E-011 | 3.3156E-011 |
| | | PFHDay2 | 1.64579E-011 | 1.64451E-011 | .324 | -1.7040E-011 | 4.9955E-011 |

| | | | | | | |
|---|---|---|---|---|---|---|
| | BMHDay2 | 1.41163E-012 | 1.64451E-011 | .932 | -3.2086E-011 | 3.4909E-011 |
| | PMHDay2 | 1.00787E-011 | 1.64451E-011 | .544 | -2.3419E-011 | 4.3576E-011 |
| PMHDay1 | BFHDay1 | -1.25036E-012 | 1.64451E-011 | .940 | -3.4748E-011 | 3.2247E-011 |
| | PFHDay1 | -4.40645E-011* | 1.64451E-011 | .012 | -7.7562E-011 | -1.0567E-011 |
| | BMHDay1 | -1.19601E-012 | 1.64451E-011 | .942 | -3.4694E-011 | 3.2302E-011 |
| | BFHDay2 | -1.53798E-012 | 1.64451E-011 | .926 | -3.5036E-011 | 3.1960E-011 |
| | PFHDay2 | 1.52619E-011 | 1.64451E-011 | .360 | -1.8236E-011 | 4.8759E-011 |
| | BMHDay2 | 2.15620E-013 | 1.64451E-011 | .990 | -3.3282E-011 | 3.3713E-011 |
| | PMHDay2 | 8.88265E-012 | 1.64451E-011 | .593 | -2.4615E-011 | 4.2380E-011 |
| BFHDay2 | BFHDay1 | 2.87620E-013 | 1.64451E-011 | .986 | -3.3210E-011 | 3.3785E-011 |
| | PFHDay1 | -4.25265E-011* | 1.64451E-011 | .014 | -7.6024E-011 | -9.0289E-012 |
| | BMHDay1 | 3.41972E-013 | 1.64451E-011 | .984 | -3.3156E-011 | 3.3840E-011 |
| | PMHDay1 | 1.53798E-012 | 1.64451E-011 | .926 | -3.1960E-011 | 3.5036E-011 |
| | PFHDay2 | 1.67999E-011 | 1.64451E-011 | .315 | -1.6698E-011 | 5.0297E-011 |
| | BMHDay2 | 1.75360E-012 | 1.64451E-011 | .916 | -3.1744E-011 | 3.5251E-011 |
| | PMHDay2 | 1.04206E-011 | 1.64451E-011 | .531 | -2.3077E-011 | 4.3918E-011 |
| PFHDay2 | BFHDay1 | -1.65123E-011 | 1.64451E-011 | .323 | -5.0010E-011 | 1.6985E-011 |
| | PFHDay1 | -5.93264E-011* | 1.64451E-011 | .001 | -9.2824E-011 | -2.5829E-011 |
| | BMHDay1 | -1.64579E-011 | 1.64451E-011 | .324 | -4.9955E-011 | 1.7040E-011 |

| | | | | | | | |
|---|---|---|---|---|---|---|---|
| | | PMHDay1 | -1.52619E-011 | 1.64451E-011 | .360 | -4.8759E-011 | 1.8236E-011 |
| | | BFHDay2 | -1.67999E-011 | 1.64451E-011 | .315 | -5.0297E-011 | 1.6698E-011 |
| | | BMHDay2 | -1.50463E-011 | 1.64451E-011 | .367 | -4.8544E-011 | 1.8451E-011 |
| | | PMHDay2 | -6.37925E-012 | 1.64451E-011 | .701 | -3.9877E-011 | 2.7118E-011 |
| | BMHDay2 | BFHDay1 | -1.46598E-012 | 1.64451E-011 | .930 | -3.4964E-011 | 3.2032E-011 |
| | | PFHDay1 | -4.42801E-011* | 1.64451E-011 | .011 | -7.7778E-011 | -1.0783E-011 |
| | | BMHDay1 | -1.41163E-012 | 1.64451E-011 | .932 | -3.4909E-011 | 3.2086E-011 |
| | | PMHDay1 | -2.15620E-013 | 1.64451E-011 | .990 | -3.3713E-011 | 3.3282E-011 |
| | | BFHDay2 | -1.75360E-012 | 1.64451E-011 | .916 | -3.5251E-011 | 3.1744E-011 |
| | | PFHDay2 | 1.50463E-011 | 1.64451E-011 | .367 | -1.8451E-011 | 4.8544E-011 |
| | | PMHDay2 | 8.66703E-012 | 1.64451E-011 | .602 | -2.4831E-011 | 4.2165E-011 |
| | PMHDay2 | BFHDay1 | -1.01330E-011 | 1.64451E-011 | .542 | -4.3631E-011 | 2.3365E-011 |
| | | PFHDay1 | -5.29471E-011* | 1.64451E-011 | .003 | -8.6445E-011 | -1.9450E-011 |
| | | BMHDay1 | -1.00787E-011 | 1.64451E-011 | .544 | -4.3576E-011 | 2.3419E-011 |
| | | PMHDay1 | -8.88265E-012 | 1.64451E-011 | .593 | -4.2380E-011 | 2.4615E-011 |
| | | BFHDay2 | -1.04206E-011 | 1.64451E-011 | .531 | -4.3918E-011 | 2.3077E-011 |
| | | PFHDay2 | 6.37925E-012 | 1.64451E-011 | .701 | -2.7118E-011 | 3.9877E-011 |
| | | BMHDay2 | -8.66703E-012 | 1.64451E-011 | .602 | -4.2165E-011 | 2.4831E-011 |
| AA | BFHDay1 | PFHDay1 | 8.72616E-010 | 5.08313E-010 | .096 | -1.6278E-010 | 1.9080E-009 |

| | | | | | | |
|---|---|---|---|---|---|---|
| | BMHDay1 | 2.29990E-009* | 5.08313E-010 | <.001 | 1.2645E-009 | 3.3353E-009 |
| | PMHDay1 | 1.72213E-009* | 5.08313E-010 | .002 | 6.8672E-010 | 2.7575E-009 |
| | BFHDay2 | 1.11703E-009* | 5.08313E-010 | .035 | 8.1634E-011 | 2.1524E-009 |
| | PFHDay2 | 4.53292E-010 | 5.08313E-010 | .379 | -5.8211E-010 | 1.4887E-009 |
| | BMHDay2 | 1.84502E-009* | 5.08313E-010 | <.001 | 8.0962E-010 | 2.8804E-009 |
| | PMHDay2 | 1.44030E-009* | 5.08313E-010 | .008 | 4.0490E-010 | 2.4757E-009 |
| PFHDay1 | BFHDay1 | -8.72616E-010 | 5.08313E-010 | .096 | -1.9080E-009 | 1.6278E-010 |
| | BMHDay1 | 1.42728E-009* | 5.08313E-010 | .008 | 3.9188E-010 | 2.4627E-009 |
| | PMHDay1 | 8.49510E-010 | 5.08313E-010 | .104 | -1.8589E-010 | 1.8849E-009 |
| | BFHDay2 | 2.44418E-010 | 5.08313E-010 | .634 | -7.9098E-010 | 1.2798E-009 |
| | PFHDay2 | -4.19324E-010 | 5.08313E-010 | .416 | -1.4547E-009 | 6.1608E-010 |
| | BMHDay2 | 9.72408E-010 | 5.08313E-010 | .065 | -6.2993E-011 | 2.0078E-009 |
| | PMHDay2 | 5.67684E-010 | 5.08313E-010 | .272 | -4.6772E-010 | 1.6031E-009 |
| BMHDay1 | BFHDay1 | -2.29990E-009* | 5.08313E-010 | <.001 | -3.3353E-009 | -1.2645E-009 |
| | PFHDay1 | -1.42728E-009* | 5.08313E-010 | .008 | -2.4627E-009 | -3.9188E-010 |
| | PMHDay1 | -5.77771E-010 | 5.08313E-010 | .264 | -1.6132E-009 | 4.5763E-010 |
| | BFHDay2 | -1.18286E-009* | 5.08313E-010 | .026 | -2.2183E-009 | -1.4746E-010 |
| | PFHDay2 | -1.84660E-009* | 5.08313E-010 | <.001 | -2.8820E-009 | -8.1120E-010 |
| | BMHDay2 | -4.54873E-010 | 5.08313E-010 | .378 | -1.4903E-009 | 5.8053E-010 |

| | | | | | | |
|---|---|---|---|---|---|---|
| | PMHDay2 | -8.59597E-010 | 5.08313E-010 | .101 | -1.8950E-009 | 1.7580E-010 |
| PMHDay1 | BFHDay1 | -1.72213E-009* | 5.08313E-010 | .002 | -2.7575E-009 | -6.8672E-010 |
| | PFHDay1 | -8.49510E-010 | 5.08313E-010 | .104 | -1.8849E-009 | 1.8589E-010 |
| | BMHDay1 | 5.77771E-010 | 5.08313E-010 | .264 | -4.5763E-010 | 1.6132E-009 |
| | BFHDay2 | -6.05091E-010 | 5.08313E-010 | .243 | -1.6405E-009 | 4.3031E-010 |
| | PFHDay2 | -1.26883E-009* | 5.08313E-010 | .018 | -2.3042E-009 | -2.3343E-010 |
| | BMHDay2 | 1.22898E-010 | 5.08313E-010 | .810 | -9.1250E-010 | 1.1583E-009 |
| | PMHDay2 | -2.81825E-010 | 5.08313E-010 | .583 | -1.3172E-009 | 7.5357E-010 |
| BFHDay2 | BFHDay1 | -1.11703E-009* | 5.08313E-010 | .035 | -2.1524E-009 | -8.1634E-011 |
| | PFHDay1 | -2.44418E-010 | 5.08313E-010 | .634 | -1.2798E-009 | 7.9098E-010 |
| | BMHDay1 | 1.18286E-009* | 5.08313E-010 | .026 | 1.4746E-010 | 2.2183E-009 |
| | PMHDay1 | 6.05091E-010 | 5.08313E-010 | .243 | -4.3031E-010 | 1.6405E-009 |
| | PFHDay2 | -6.63742E-010 | 5.08313E-010 | .201 | -1.6991E-009 | 3.7166E-010 |
| | BMHDay2 | 7.27989E-010 | 5.08313E-010 | .162 | -3.0741E-010 | 1.7634E-009 |
| | PMHDay2 | 3.23266E-010 | 5.08313E-010 | .529 | -7.1213E-010 | 1.3587E-009 |
| PFHDay2 | BFHDay1 | -4.53292E-010 | 5.08313E-010 | .379 | -1.4887E-009 | 5.8211E-010 |
| | PFHDay1 | 4.19324E-010 | 5.08313E-010 | .416 | -6.1608E-010 | 1.4547E-009 |
| | BMHDay1 | 1.84660E-009* | 5.08313E-010 | <.001 | 8.1120E-010 | 2.8820E-009 |
| | PMHDay1 | 1.26883E-009* | 5.08313E-010 | .018 | 2.3343E-010 | 2.3042E-009 |

| | | | | | | | |
|---|---|---|---|---|---|---|---|
| | | BFHDay2 | 6.63742E-010 | 5.08313E-010 | .201 | -3.7166E-010 | 1.6991E-009 |
| | | BMHDay2 | 1.39173E-009* | 5.08313E-010 | .010 | 3.5633E-010 | 2.4271E-009 |
| | | PMHDay2 | 9.87008E-010 | 5.08313E-010 | .061 | -4.8392E-011 | 2.0224E-009 |
| | BMHDay2 | BFHDay1 | -1.84502E-009* | 5.08313E-010 | <.001 | -2.8804E-009 | -8.0962E-010 |
| | | PFHDay1 | -9.72408E-010 | 5.08313E-010 | .065 | -2.0078E-009 | 6.2993E-011 |
| | | BMHDay1 | 4.54873E-010 | 5.08313E-010 | .378 | -5.8053E-010 | 1.4903E-009 |
| | | PMHDay1 | -1.22898E-010 | 5.08313E-010 | .810 | -1.1583E-009 | 9.1250E-010 |
| | | BFHDay2 | -7.27989E-010 | 5.08313E-010 | .162 | -1.7634E-009 | 3.0741E-010 |
| | | PFHDay2 | -1.39173E-009* | 5.08313E-010 | .010 | -2.4271E-009 | -3.5633E-010 |
| | | PMHDay2 | -4.04723E-010 | 5.08313E-010 | .432 | -1.4401E-009 | 6.3068E-010 |
| | PMHDay2 | BFHDay1 | -1.44030E-009* | 5.08313E-010 | .008 | -2.4757E-009 | -4.0490E-010 |
| | | PFHDay1 | -5.67684E-010 | 5.08313E-010 | .272 | -1.6031E-009 | 4.6772E-010 |
| | | BMHDay1 | 8.59597E-010 | 5.08313E-010 | .101 | -1.7580E-010 | 1.8950E-009 |
| | | PMHDay1 | 2.81825E-010 | 5.08313E-010 | .583 | -7.5357E-010 | 1.3172E-009 |
| | | BFHDay2 | -3.23266E-010 | 5.08313E-010 | .529 | -1.3587E-009 | 7.1213E-010 |
| | | PFHDay2 | -9.87008E-010 | 5.08313E-010 | .061 | -2.0224E-009 | 4.8392E-011 |
| | | BMHDay2 | 4.04723E-010 | 5.08313E-010 | .432 | -6.3068E-010 | 1.4401E-009 |
| OA | BFHDay1 | PFHDay1 | 6.96386E-010 | 6.33848E-009 | .913 | -1.2215E-008 | 1.3607E-008 |
| | | BMHDay1 | 8.62108E-009 | 6.33848E-009 | .183 | -4.2900E-009 | 2.1532E-008 |

| | | | | | | |
|---|---|---|---|---|---|---|
| | PMHDay1 | -5.93544E-010 | 6.33848E-009 | .926 | -1.3505E-008 | 1.2318E-008 |
| | BFHDay2 | 4.55075E-009 | 6.33848E-009 | .478 | -8.3603E-009 | 1.7462E-008 |
| | PFHDay2 | -2.60287E-009 | 6.33848E-009 | .684 | -1.5514E-008 | 1.0308E-008 |
| | BMHDay2 | 2.09177E-009 | 6.33848E-009 | .744 | -1.0819E-008 | 1.5003E-008 |
| | PMHDay2 | -1.68818E-008* | 6.33848E-009 | .012 | -2.9793E-008 | -3.9707E-009 |
| PFHDay1 | BFHDay1 | -6.96386E-010 | 6.33848E-009 | .913 | -1.3607E-008 | 1.2215E-008 |
| | BMHDay1 | 7.92469E-009 | 6.33848E-009 | .220 | -4.9864E-009 | 2.0836E-008 |
| | PMHDay1 | -1.28993E-009 | 6.33848E-009 | .840 | -1.4201E-008 | 1.1621E-008 |
| | BFHDay2 | 3.85437E-009 | 6.33848E-009 | .547 | -9.0567E-009 | 1.6765E-008 |
| | PFHDay2 | -3.29926E-009 | 6.33848E-009 | .606 | -1.6210E-008 | 9.6118E-009 |
| | BMHDay2 | 1.39538E-009 | 6.33848E-009 | .827 | -1.1516E-008 | 1.4306E-008 |
| | PMHDay2 | -1.75782E-008* | 6.33848E-009 | .009 | -3.0489E-008 | -4.6671E-009 |
| BMHDay1 | BFHDay1 | -8.62108E-009 | 6.33848E-009 | .183 | -2.1532E-008 | 4.2900E-009 |
| | PFHDay1 | -7.92469E-009 | 6.33848E-009 | .220 | -2.0836E-008 | 4.9864E-009 |
| | PMHDay1 | -9.21462E-009 | 6.33848E-009 | .156 | -2.2126E-008 | 3.6964E-009 |
| | BFHDay2 | -4.07033E-009 | 6.33848E-009 | .525 | -1.6981E-008 | 8.8407E-009 |
| | PFHDay2 | -1.12240E-008 | 6.33848E-009 | .086 | -2.4135E-008 | 1.6871E-009 |
| | BMHDay2 | -6.52931E-009 | 6.33848E-009 | .311 | -1.9440E-008 | 6.3817E-009 |
| | PMHDay2 | -2.55029E-008* | 6.33848E-009 | <.001 | -3.8414E-008 | -1.2592E-008 |

| | | | | | | |
|---|---|---|---|---|---|---|
| PMHDay1 | BFHDay1 | 5.93544E-010 | 6.33848E-009 | .926 | -1.2318E-008 | 1.3505E-008 |
| | PFHDay1 | 1.28993E-009 | 6.33848E-009 | .840 | -1.1621E-008 | 1.4201E-008 |
| | BMHDay1 | 9.21462E-009 | 6.33848E-009 | .156 | -3.6964E-009 | 2.2126E-008 |
| | BFHDay2 | 5.14430E-009 | 6.33848E-009 | .423 | -7.7668E-009 | 1.8055E-008 |
| | PFHDay2 | -2.00933E-009 | 6.33848E-009 | .753 | -1.4920E-008 | 1.0902E-008 |
| | BMHDay2 | 2.68531E-009 | 6.33848E-009 | .675 | -1.0226E-008 | 1.5596E-008 |
| | PMHDay2 | -1.62883E-008* | 6.33848E-009 | .015 | -2.9199E-008 | -3.3772E-009 |
| BFHDay2 | BFHDay1 | -4.55075E-009 | 6.33848E-009 | .478 | -1.7462E-008 | 8.3603E-009 |
| | PFHDay1 | -3.85437E-009 | 6.33848E-009 | .547 | -1.6765E-008 | 9.0567E-009 |
| | BMHDay1 | 4.07033E-009 | 6.33848E-009 | .525 | -8.8407E-009 | 1.6981E-008 |
| | PMHDay1 | -5.14430E-009 | 6.33848E-009 | .423 | -1.8055E-008 | 7.7668E-009 |
| | PFHDay2 | -7.15363E-009 | 6.33848E-009 | .267 | -2.0065E-008 | 5.7574E-009 |
| | BMHDay2 | -2.45899E-009 | 6.33848E-009 | .701 | -1.5370E-008 | 1.0452E-008 |
| | PMHDay2 | -2.14326E-008* | 6.33848E-009 | .002 | -3.4344E-008 | -8.5215E-009 |
| PFHDay2 | BFHDay1 | 2.60287E-009 | 6.33848E-009 | .684 | -1.0308E-008 | 1.5514E-008 |
| | PFHDay1 | 3.29926E-009 | 6.33848E-009 | .606 | -9.6118E-009 | 1.6210E-008 |
| | BMHDay1 | 1.12240E-008 | 6.33848E-009 | .086 | -1.6871E-009 | 2.4135E-008 |
| | PMHDay1 | 2.00933E-009 | 6.33848E-009 | .753 | -1.0902E-008 | 1.4920E-008 |
| | BFHDay2 | 7.15363E-009 | 6.33848E-009 | .267 | -5.7574E-009 | 2.0065E-008 |

| | | | | | | | |
|---|---|---|---|---|---|---|---|
| | | BMHDay2 | 4.69464E-009 | 6.33848E-009 | .464 | -8.2164E-009 | 1.7606E-008 |
| | | PMHDay2 | -1.42789E-008* | 6.33848E-009 | .031 | -2.7190E-008 | -1.3679E-009 |
| | BMHDay2 | BFHDay1 | -2.09177E-009 | 6.33848E-009 | .744 | -1.5003E-008 | 1.0819E-008 |
| | | PFHDay1 | -1.39538E-009 | 6.33848E-009 | .827 | -1.4306E-008 | 1.1516E-008 |
| | | BMHDay1 | 6.52931E-009 | 6.33848E-009 | .311 | -6.3817E-009 | 1.9440E-008 |
| | | PMHDay1 | -2.68531E-009 | 6.33848E-009 | .675 | -1.5596E-008 | 1.0226E-008 |
| | | BFHDay2 | 2.45899E-009 | 6.33848E-009 | .701 | -1.0452E-008 | 1.5370E-008 |
| | | PFHDay2 | -4.69464E-009 | 6.33848E-009 | .464 | -1.7606E-008 | 8.2164E-009 |
| | | PMHDay2 | -1.89736E-008* | 6.33848E-009 | .005 | -3.1885E-008 | -6.0625E-009 |
| | PMHDay2 | BFHDay1 | 1.68818E-008* | 6.33848E-009 | .012 | 3.9707E-009 | 2.9793E-008 |
| | | PFHDay1 | 1.75782E-008* | 6.33848E-009 | .009 | 4.6671E-009 | 3.0489E-008 |
| | | BMHDay1 | 2.55029E-008* | 6.33848E-009 | <.001 | 1.2592E-008 | 3.8414E-008 |
| | | PMHDay1 | 1.62883E-008* | 6.33848E-009 | .015 | 3.3772E-009 | 2.9199E-008 |
| | | BFHDay2 | 2.14326E-008* | 6.33848E-009 | .002 | 8.5215E-009 | 3.4344E-008 |
| | | PFHDay2 | 1.42789E-008* | 6.33848E-009 | .031 | 1.3679E-009 | 2.7190E-008 |
| | | BMHDay2 | 1.89736E-008* | 6.33848E-009 | .005 | 6.0625E-009 | 3.1885E-008 |
| EIC | BFHDay1 | PFHDay1 | 2.38691E-012 | 2.54489E-012 | .355 | -2.7969E-012 | 7.5707E-012 |
| | | BMHDay1 | 4.42551E-012 | 2.54489E-012 | .092 | -7.5826E-013 | 9.6093E-012 |
| | | PMHDay1 | -1.36896E-012 | 2.54489E-012 | .594 | -6.5527E-012 | 3.8148E-012 |

| | | | | | | |
|---|---|---|---|---|---|---|
| | BFHDay2 | 2.41514E-012 | 2.54489E-012 | .350 | -2.7686E-012 | 7.5989E-012 |
| | PFHDay2 | 8.96720E-014 | 2.54489E-012 | .972 | -5.0941E-012 | 5.2734E-012 |
| | BMHDay2 | 2.04219E-012 | 2.54489E-012 | .428 | -3.1416E-012 | 7.2260E-012 |
| | PMHDay2 | -4.24102E-012 | 2.54489E-012 | .105 | -9.4248E-012 | 9.4275E-013 |
| PFHDay1 | BFHDay1 | -2.38691E-012 | 2.54489E-012 | .355 | -7.5707E-012 | 2.7969E-012 |
| | BMHDay1 | 2.03860E-012 | 2.54489E-012 | .429 | -3.1452E-012 | 7.2224E-012 |
| | PMHDay1 | -3.75586E-012 | 2.54489E-012 | .150 | -8.9396E-012 | 1.4279E-012 |
| | BFHDay2 | 2.82358E-014 | 2.54489E-012 | .991 | -5.1555E-012 | 5.2120E-012 |
| | PFHDay2 | -2.29723E-012 | 2.54489E-012 | .373 | -7.4810E-012 | 2.8865E-012 |
| | BMHDay2 | -3.44712E-013 | 2.54489E-012 | .893 | -5.5285E-012 | 4.8391E-012 |
| | PMHDay2 | -6.62792E-012* | 2.54489E-012 | .014 | -1.1812E-011 | -1.4442E-012 |
| BMHDay1 | BFHDay1 | -4.42551E-012 | 2.54489E-012 | .092 | -9.6093E-012 | 7.5826E-013 |
| | PFHDay1 | -2.03860E-012 | 2.54489E-012 | .429 | -7.2224E-012 | 3.1452E-012 |
| | PMHDay1 | -5.79447E-012* | 2.54489E-012 | .030 | -1.0978E-011 | -6.1070E-013 |
| | BFHDay2 | -2.01037E-012 | 2.54489E-012 | .435 | -7.1941E-012 | 3.1734E-012 |
| | PFHDay2 | -4.33584E-012 | 2.54489E-012 | .098 | -9.5196E-012 | 8.4793E-013 |
| | BMHDay2 | -2.38331E-012 | 2.54489E-012 | .356 | -7.5671E-012 | 2.8005E-012 |
| | PMHDay2 | -8.66653E-012* | 2.54489E-012 | .002 | -1.3850E-011 | -3.4828E-012 |
| PMHDay1 | BFHDay1 | 1.36896E-012 | 2.54489E-012 | .594 | -3.8148E-012 | 6.5527E-012 |

| | | | | | | |
|---|---|---|---|---|---|---|
| | PFHDay1 | 3.75586E-012 | 2.54489E-012 | .150 | -1.4279E-012 | 8.9396E-012 |
| | BMHDay1 | 5.79447E-012* | 2.54489E-012 | .030 | 6.1070E-013 | 1.0978E-011 |
| | BFHDay2 | 3.78410E-012 | 2.54489E-012 | .147 | -1.3997E-012 | 8.9679E-012 |
| | PFHDay2 | 1.45863E-012 | 2.54489E-012 | .571 | -3.7251E-012 | 6.6424E-012 |
| | BMHDay2 | 3.41115E-012 | 2.54489E-012 | .190 | -1.7726E-012 | 8.5949E-012 |
| | PMHDay2 | -2.87206E-012 | 2.54489E-012 | .267 | -8.0558E-012 | 2.3117E-012 |
| BFHDay2 | BFHDay1 | -2.41514E-012 | 2.54489E-012 | .350 | -7.5989E-012 | 2.7686E-012 |
| | PFHDay1 | -2.82358E-014 | 2.54489E-012 | .991 | -5.2120E-012 | 5.1555E-012 |
| | BMHDay1 | 2.01037E-012 | 2.54489E-012 | .435 | -3.1734E-012 | 7.1941E-012 |
| | PMHDay1 | -3.78410E-012 | 2.54489E-012 | .147 | -8.9679E-012 | 1.3997E-012 |
| | PFHDay2 | -2.32547E-012 | 2.54489E-012 | .368 | -7.5092E-012 | 2.8583E-012 |
| | BMHDay2 | -3.72948E-013 | 2.54489E-012 | .884 | -5.5567E-012 | 4.8108E-012 |
| | PMHDay2 | -6.65616E-012* | 2.54489E-012 | .013 | -1.1840E-011 | -1.4724E-012 |
| PFHDay2 | BFHDay1 | -8.96720E-014 | 2.54489E-012 | .972 | -5.2734E-012 | 5.0941E-012 |
| | PFHDay1 | 2.29723E-012 | 2.54489E-012 | .373 | -2.8865E-012 | 7.4810E-012 |
| | BMHDay1 | 4.33584E-012 | 2.54489E-012 | .098 | -8.4793E-013 | 9.5196E-012 |
| | PMHDay1 | -1.45863E-012 | 2.54489E-012 | .571 | -6.6424E-012 | 3.7251E-012 |
| | BFHDay2 | 2.32547E-012 | 2.54489E-012 | .368 | -2.8583E-012 | 7.5092E-012 |
| | BMHDay2 | 1.95252E-012 | 2.54489E-012 | .449 | -3.2312E-012 | 7.1363E-012 |

| | | | | | | | |
|---|---|---|---|---|---|---|---|
| | | PMHDay2 | -4.33069E-012 | 2.54489E-012 | .099 | -9.5145E-012 | 8.5308E-013 |
| | BMHDay2 | BFHDay1 | -2.04219E-012 | 2.54489E-012 | .428 | -7.2260E-012 | 3.1416E-012 |
| | | PFHDay1 | 3.44712E-013 | 2.54489E-012 | .893 | -4.8391E-012 | 5.5285E-012 |
| | | BMHDay1 | 2.38331E-012 | 2.54489E-012 | .356 | -2.8005E-012 | 7.5671E-012 |
| | | PMHDay1 | -3.41115E-012 | 2.54489E-012 | .190 | -8.5949E-012 | 1.7726E-012 |
| | | BFHDay2 | 3.72948E-013 | 2.54489E-012 | .884 | -4.8108E-012 | 5.5567E-012 |
| | | PFHDay2 | -1.95252E-012 | 2.54489E-012 | .449 | -7.1363E-012 | 3.2312E-012 |
| | | PMHDay2 | -6.28321E-012* | 2.54489E-012 | .019 | -1.1467E-011 | -1.0994E-012 |
| | PMHDay2 | BFHDay1 | 4.24102E-012 | 2.54489E-012 | .105 | -9.4275E-013 | 9.4248E-012 |
| | | PFHDay1 | 6.62792E-012* | 2.54489E-012 | .014 | 1.4442E-012 | 1.1812E-011 |
| | | BMHDay1 | 8.66653E-012* | 2.54489E-012 | .002 | 3.4828E-012 | 1.3850E-011 |
| | | PMHDay1 | 2.87206E-012 | 2.54489E-012 | .267 | -2.3117E-012 | 8.0558E-012 |
| | | BFHDay2 | 6.65616E-012* | 2.54489E-012 | .013 | 1.4724E-012 | 1.1840E-011 |
| | | PFHDay2 | 4.33069E-012 | 2.54489E-012 | .099 | -8.5308E-013 | 9.5145E-012 |
| | | BMHDay2 | 6.28321E-012* | 2.54489E-012 | .019 | 1.0994E-012 | 1.1467E-011 |
| DOC | BFHDay1 | PFHDay1 | 4.22567E-008* | 1.81198E-008 | .026 | 5.3478E-009 | 7.9166E-008 |
| | | BMHDay1 | 6.07581E-008* | 1.81198E-008 | .002 | 2.3849E-008 | 9.7667E-008 |
| | | PMHDay1 | 3.40346E-008 | 1.81198E-008 | .069 | -2.8743E-009 | 7.0943E-008 |
| | | BFHDay2 | 4.61954E-008* | 1.81198E-008 | .016 | 9.2865E-009 | 8.3104E-008 |

| | | | | | | |
|---|---|---|---|---|---|---|
| | PFHDay2 | 2.17919E-008 | 1.81198E-008 | .238 | -1.5117E-008 | 5.8701E-008 |
| | BMHDay2 | 5.55593E-008* | 1.81198E-008 | .004 | 1.8650E-008 | 9.2468E-008 |
| | PMHDay2 | 4.46113E-008* | 1.81198E-008 | .019 | 7.7024E-009 | 8.1520E-008 |
| PFHDay1 | BFHDay1 | -4.22567E-008* | 1.81198E-008 | .026 | -7.9166E-008 | -5.3478E-009 |
| | BMHDay1 | 1.85015E-008 | 1.81198E-008 | .315 | -1.8407E-008 | 5.5410E-008 |
| | PMHDay1 | -8.22204E-009 | 1.81198E-008 | .653 | -4.5131E-008 | 2.8687E-008 |
| | BFHDay2 | 3.93875E-009 | 1.81198E-008 | .829 | -3.2970E-008 | 4.0848E-008 |
| | PFHDay2 | -2.04648E-008 | 1.81198E-008 | .267 | -5.7374E-008 | 1.6444E-008 |
| | BMHDay2 | 1.33026E-008 | 1.81198E-008 | .468 | -2.3606E-008 | 5.0211E-008 |
| | PMHDay2 | 2.35461E-009 | 1.81198E-008 | .897 | -3.4554E-008 | 3.9263E-008 |
| BMHDay1 | BFHDay1 | -6.07581E-008* | 1.81198E-008 | .002 | -9.7667E-008 | -2.3849E-008 |
| | PFHDay1 | -1.85015E-008 | 1.81198E-008 | .315 | -5.5410E-008 | 1.8407E-008 |
| | PMHDay1 | -2.67235E-008 | 1.81198E-008 | .150 | -6.3632E-008 | 1.0185E-008 |
| | BFHDay2 | -1.45627E-008 | 1.81198E-008 | .428 | -5.1472E-008 | 2.2346E-008 |
| | PFHDay2 | -3.89663E-008* | 1.81198E-008 | .039 | -7.5875E-008 | -2.0574E-009 |
| | BMHDay2 | -5.19885E-009 | 1.81198E-008 | .776 | -4.2108E-008 | 3.1710E-008 |
| | PMHDay2 | -1.61469E-008 | 1.81198E-008 | .380 | -5.3056E-008 | 2.0762E-008 |
| PMHDay1 | BFHDay1 | -3.40346E-008 | 1.81198E-008 | .069 | -7.0943E-008 | 2.8743E-009 |
| | PFHDay1 | 8.22204E-009 | 1.81198E-008 | .653 | -2.8687E-008 | 4.5131E-008 |

| | | | | | | | |
|---|---|---|---|---|---|---|---|
| | | BMHDay1 | 2.67235E-008 | 1.81198E-008 | .150 | -1.0185E-008 | 6.3632E-008 |
| | | BFHDay2 | 1.21608E-008 | 1.81198E-008 | .507 | -2.4748E-008 | 4.9070E-008 |
| | | PFHDay2 | -1.22428E-008 | 1.81198E-008 | .504 | -4.9152E-008 | 2.4666E-008 |
| | | BMHDay2 | 2.15247E-008 | 1.81198E-008 | .244 | -1.5384E-008 | 5.8434E-008 |
| | | PMHDay2 | 1.05766E-008 | 1.81198E-008 | .564 | -2.6332E-008 | 4.7486E-008 |
| | BFHDay2 | BFHDay1 | -4.61954E-008* | 1.81198E-008 | .016 | -8.3104E-008 | -9.2865E-009 |
| | | PFHDay1 | -3.93875E-009 | 1.81198E-008 | .829 | -4.0848E-008 | 3.2970E-008 |
| | | BMHDay1 | 1.45627E-008 | 1.81198E-008 | .428 | -2.2346E-008 | 5.1472E-008 |
| | | PMHDay1 | -1.21608E-008 | 1.81198E-008 | .507 | -4.9070E-008 | 2.4748E-008 |
| | | PFHDay2 | -2.44036E-008 | 1.81198E-008 | .188 | -6.1312E-008 | 1.2505E-008 |
| | | BMHDay2 | 9.36386E-009 | 1.81198E-008 | .609 | -2.7545E-008 | 4.6273E-008 |
| | | PMHDay2 | -1.58414E-009 | 1.81198E-008 | .931 | -3.8493E-008 | 3.5325E-008 |
| | PFHDay2 | BFHDay1 | -2.17919E-008 | 1.81198E-008 | .238 | -5.8701E-008 | 1.5117E-008 |
| | | PFHDay1 | 2.04648E-008 | 1.81198E-008 | .267 | -1.6444E-008 | 5.7374E-008 |
| | | BMHDay1 | 3.89663E-008* | 1.81198E-008 | .039 | 2.0574E-009 | 7.5875E-008 |
| | | PMHDay1 | 1.22428E-008 | 1.81198E-008 | .504 | -2.4666E-008 | 4.9152E-008 |
| | | BFHDay2 | 2.44036E-008 | 1.81198E-008 | .188 | -1.2505E-008 | 6.1312E-008 |
| | | BMHDay2 | 3.37674E-008 | 1.81198E-008 | .072 | -3.1415E-009 | 7.0676E-008 |
| | | PMHDay2 | 2.28194E-008 | 1.81198E-008 | .217 | -1.4089E-008 | 5.9728E-008 |

| | | | | | | | |
|---|---|---|---|---|---|---|---|
| | BMHDay2 | BFHDay1 | -5.55593E-008* | 1.81198E-008 | .004 | -9.2468E-008 | -1.8650E-008 |
| | | PFHDay1 | -1.33026E-008 | 1.81198E-008 | .468 | -5.0211E-008 | 2.3606E-008 |
| | | BMHDay1 | 5.19885E-009 | 1.81198E-008 | .776 | -3.1710E-008 | 4.2108E-008 |
| | | PMHDay1 | -2.15247E-008 | 1.81198E-008 | .244 | -5.8434E-008 | 1.5384E-008 |
| | | BFHDay2 | -9.36386E-009 | 1.81198E-008 | .609 | -4.6273E-008 | 2.7545E-008 |
| | | PFHDay2 | -3.37674E-008 | 1.81198E-008 | .072 | -7.0676E-008 | 3.1415E-009 |
| | | PMHDay2 | -1.09480E-008 | 1.81198E-008 | .550 | -4.7857E-008 | 2.5961E-008 |
| | PMHDay2 | BFHDay1 | -4.46113E-008* | 1.81198E-008 | .019 | -8.1520E-008 | -7.7024E-009 |
| | | PFHDay1 | -2.35461E-009 | 1.81198E-008 | .897 | -3.9263E-008 | 3.4554E-008 |
| | | BMHDay1 | 1.61469E-008 | 1.81198E-008 | .380 | -2.0762E-008 | 5.3056E-008 |
| | | PMHDay1 | -1.05766E-008 | 1.81198E-008 | .564 | -4.7486E-008 | 2.6332E-008 |
| | | BFHDay2 | 1.58414E-009 | 1.81198E-008 | .931 | -3.5325E-008 | 3.8493E-008 |
| | | PFHDay2 | -2.28194E-008 | 1.81198E-008 | .217 | -5.9728E-008 | 1.4089E-008 |
| | | BMHDay2 | 1.09480E-008 | 1.81198E-008 | .550 | -2.5961E-008 | 4.7857E-008 |
| LIN | BFHDay1 | PFHDay1 | 2.19197E-007 | 1.69528E-006 | .898 | -3.2340E-006 | 3.6724E-006 |
| | | BMHDay1 | 8.59688E-007 | 1.69528E-006 | .616 | -2.5935E-006 | 4.3129E-006 |
| | | PMHDay1 | 1.44007E-007 | 1.69528E-006 | .933 | -3.3092E-006 | 3.5972E-006 |
| | | BFHDay2 | 3.94760E-007 | 1.69528E-006 | .817 | -3.0584E-006 | 3.8479E-006 |
| | | PFHDay2 | -4.33488E-007 | 1.69528E-006 | .800 | -3.8867E-006 | 3.0197E-006 |

| | | | | | | |
|---|---|---|---|---|---|---|
| | BMHDay2 | -9.05474E-007 | 1.69528E-006 | .597 | -4.3587E-006 | 2.5477E-006 |
| | PMHDay2 | -6.47243E-006* | 1.69528E-006 | <.001 | -9.9256E-006 | -3.0193E-006 |
| PFHDay1 | BFHDay1 | -2.19197E-007 | 1.69528E-006 | .898 | -3.6724E-006 | 3.2340E-006 |
| | BMHDay1 | 6.40491E-007 | 1.69528E-006 | .708 | -2.8127E-006 | 4.0937E-006 |
| | PMHDay1 | -7.51904E-008 | 1.69528E-006 | .965 | -3.5284E-006 | 3.3780E-006 |
| | BFHDay2 | 1.75562E-007 | 1.69528E-006 | .918 | -3.2776E-006 | 3.6287E-006 |
| | PFHDay2 | -6.52685E-007 | 1.69528E-006 | .703 | -4.1059E-006 | 2.8005E-006 |
| | BMHDay2 | -1.12467E-006 | 1.69528E-006 | .512 | -4.5779E-006 | 2.3285E-006 |
| | PMHDay2 | -6.69163E-006* | 1.69528E-006 | <.001 | -1.0145E-005 | -3.2385E-006 |
| BMHDay1 | BFHDay1 | -8.59688E-007 | 1.69528E-006 | .616 | -4.3129E-006 | 2.5935E-006 |
| | PFHDay1 | -6.40491E-007 | 1.69528E-006 | .708 | -4.0937E-006 | 2.8127E-006 |
| | PMHDay1 | -7.15682E-007 | 1.69528E-006 | .676 | -4.1689E-006 | 2.7375E-006 |
| | BFHDay2 | -4.64929E-007 | 1.69528E-006 | .786 | -3.9181E-006 | 2.9883E-006 |
| | PFHDay2 | -1.29318E-006 | 1.69528E-006 | .451 | -4.7464E-006 | 2.1600E-006 |
| | BMHDay2 | -1.76516E-006 | 1.69528E-006 | .306 | -5.2183E-006 | 1.6880E-006 |
| | PMHDay2 | -7.33212E-006* | 1.69528E-006 | <.001 | -1.0785E-005 | -3.8789E-006 |
| PMHDay1 | BFHDay1 | -1.44007E-007 | 1.69528E-006 | .933 | -3.5972E-006 | 3.3092E-006 |
| | PFHDay1 | 7.51904E-008 | 1.69528E-006 | .965 | -3.3780E-006 | 3.5284E-006 |
| | BMHDay1 | 7.15682E-007 | 1.69528E-006 | .676 | -2.7375E-006 | 4.1689E-006 |

| | | | | | | |
|---|---|---|---|---|---|---|
| | BFHDay2 | 2.50753E-007 | 1.69528E-006 | .883 | -3.2024E-006 | 3.7039E-006 |
| | PFHDay2 | -5.77495E-007 | 1.69528E-006 | .736 | -4.0307E-006 | 2.8757E-006 |
| | BMHDay2 | -1.04948E-006 | 1.69528E-006 | .540 | -4.5027E-006 | 2.4037E-006 |
| | PMHDay2 | -6.61644E-006* | 1.69528E-006 | <.001 | -1.0070E-005 | -3.1633E-006 |
| BFHDay2 | BFHDay1 | -3.94760E-007 | 1.69528E-006 | .817 | -3.8479E-006 | 3.0584E-006 |
| | PFHDay1 | -1.75562E-007 | 1.69528E-006 | .918 | -3.6287E-006 | 3.2776E-006 |
| | BMHDay1 | 4.64929E-007 | 1.69528E-006 | .786 | -2.9883E-006 | 3.9181E-006 |
| | PMHDay1 | -2.50753E-007 | 1.69528E-006 | .883 | -3.7039E-006 | 3.2024E-006 |
| | PFHDay2 | -8.28247E-007 | 1.69528E-006 | .628 | -4.2814E-006 | 2.6249E-006 |
| | BMHDay2 | -1.30023E-006 | 1.69528E-006 | .449 | -4.7534E-006 | 2.1529E-006 |
| | PMHDay2 | -6.86719E-006* | 1.69528E-006 | <.001 | -1.0320E-005 | -3.4140E-006 |
| PFHDay2 | BFHDay1 | 4.33488E-007 | 1.69528E-006 | .800 | -3.0197E-006 | 3.8867E-006 |
| | PFHDay1 | 6.52685E-007 | 1.69528E-006 | .703 | -2.8005E-006 | 4.1059E-006 |
| | BMHDay1 | 1.29318E-006 | 1.69528E-006 | .451 | -2.1600E-006 | 4.7464E-006 |
| | PMHDay1 | 5.77495E-007 | 1.69528E-006 | .736 | -2.8757E-006 | 4.0307E-006 |
| | BFHDay2 | 8.28247E-007 | 1.69528E-006 | .628 | -2.6249E-006 | 4.2814E-006 |
| | BMHDay2 | -4.71986E-007 | 1.69528E-006 | .782 | -3.9252E-006 | 2.9812E-006 |
| | PMHDay2 | -6.03895E-006* | 1.69528E-006 | .001 | -9.4921E-006 | -2.5858E-006 |
| BMHDay2 | BFHDay1 | 9.05474E-007 | 1.69528E-006 | .597 | -2.5477E-006 | 4.3587E-006 |

| | | | | | | | |
|---|---|---|---|---|---|---|---|
| | | PFHDay1 | 1.12467E-006 | 1.69528E-006 | .512 | -2.3285E-006 | 4.5779E-006 |
| | | BMHDay1 | 1.76516E-006 | 1.69528E-006 | .306 | -1.6880E-006 | 5.2183E-006 |
| | | PMHDay1 | 1.04948E-006 | 1.69528E-006 | .540 | -2.4037E-006 | 4.5027E-006 |
| | | BFHDay2 | 1.30023E-006 | 1.69528E-006 | .449 | -2.1529E-006 | 4.7534E-006 |
| | | PFHDay2 | 4.71986E-007 | 1.69528E-006 | .782 | -2.9812E-006 | 3.9252E-006 |
| | | PMHDay2 | -5.56696E-006* | 1.69528E-006 | .002 | -9.0201E-006 | -2.1138E-006 |
| | PMHDay2 | BFHDay1 | 6.47243E-006* | 1.69528E-006 | <.001 | 3.0193E-006 | 9.9256E-006 |
| | | PFHDay1 | 6.69163E-006* | 1.69528E-006 | <.001 | 3.2385E-006 | 1.0145E-005 |
| | | BMHDay1 | 7.33212E-006* | 1.69528E-006 | <.001 | 3.8789E-006 | 1.0785E-005 |
| | | PMHDay1 | 6.61644E-006* | 1.69528E-006 | <.001 | 3.1633E-006 | 1.0070E-005 |
| | | BFHDay2 | 6.86719E-006* | 1.69528E-006 | <.001 | 3.4140E-006 | 1.0320E-005 |
| | | PFHDay2 | 6.03895E-006* | 1.69528E-006 | .001 | 2.5858E-006 | 9.4921E-006 |
| | | BMHDay2 | 5.56696E-006* | 1.69528E-006 | .002 | 2.1138E-006 | 9.0201E-006 |
| NAGly | BFHDay1 | PFHDay1 | 3.57979E-014 | 3.65324E-014 | .336 | -3.9295E-014 | 1.1089E-013 |
| | | BMHDay1 | 6.03839E-014 | 4.55639E-014 | .197 | -3.3274E-014 | 1.5404E-013 |
| | | PMHDay1 | 7.34237E-014* | 3.44430E-014 | .043 | 2.6250E-015 | 1.4422E-013 |
| | | BFHDay2 | 3.66939E-014 | 3.65324E-014 | .324 | -3.8399E-014 | 1.1179E-013 |
| | | PFHDay2 | 2.37817E-014 | 3.44430E-014 | .496 | -4.7017E-014 | 9.4580E-014 |
| | | BMHDay2 | 4.39541E-014 | 3.65324E-014 | .240 | -3.1139E-014 | 1.1905E-013 |

| | | | | | | |
|---|---|---|---|---|---|---|
| | | PMHDay2 | 6.09762E-014 | 3.44430E-014 | .088 | -9.8225E-015 | 1.3177E-013 |
| | PFHDay1 | BFHDay1 | -3.57979E-014 | 3.65324E-014 | .336 | -1.1089E-013 | 3.9295E-014 |
| | | BMHDay1 | 2.45860E-014 | 4.71631E-014 | .607 | -7.2359E-014 | 1.2153E-013 |
| | | PMHDay1 | 3.76258E-014 | 3.65324E-014 | .313 | -3.7468E-014 | 1.1272E-013 |
| | | BFHDay2 | 8.95975E-016 | 3.85085E-014 | .982 | -7.8259E-014 | 8.0051E-014 |
| | | PFHDay2 | -1.20162E-014 | 3.65324E-014 | .745 | -8.7110E-014 | 6.3077E-014 |
| | | BMHDay2 | 8.15625E-015 | 3.85085E-014 | .834 | -7.0999E-014 | 8.7312E-014 |
| | | PMHDay2 | 2.51783E-014 | 3.65324E-014 | .497 | -4.9915E-014 | 1.0027E-013 |
| | BMHDay1 | BFHDay1 | -6.03839E-014 | 4.55639E-014 | .197 | -1.5404E-013 | 3.3274E-014 |
| | | PFHDay1 | -2.45860E-014 | 4.71631E-014 | .607 | -1.2153E-013 | 7.2359E-014 |
| | | PMHDay1 | 1.30398E-014 | 4.55639E-014 | .777 | -8.0618E-014 | 1.0670E-013 |
| | | BFHDay2 | -2.36901E-014 | 4.71631E-014 | .620 | -1.2064E-013 | 7.3255E-014 |
| | | PFHDay2 | -3.66022E-014 | 4.55639E-014 | .429 | -1.3026E-013 | 5.7056E-014 |
| | | BMHDay2 | -1.64298E-014 | 4.71631E-014 | .730 | -1.1337E-013 | 8.0515E-014 |
| | | PMHDay2 | 5.92300E-016 | 4.55639E-014 | .990 | -9.3066E-014 | 9.4250E-014 |
| | PMHDay1 | BFHDay1 | -7.34237E-014* | 3.44430E-014 | .043 | -1.4422E-013 | -2.6250E-015 |
| | | PFHDay1 | -3.76258E-014 | 3.65324E-014 | .313 | -1.1272E-013 | 3.7468E-014 |
| | | BMHDay1 | -1.30398E-014 | 4.55639E-014 | .777 | -1.0670E-013 | 8.0618E-014 |
| | | BFHDay2 | -3.67298E-014 | 3.65324E-014 | .324 | -1.1182E-013 | 3.8364E-014 |

| | | | | | | |
|---|---|---|---|---|---|---|
| | | PFHDay2 | -4.96420E-014 | 3.44430E-014 | .161 | -1.2044E-013 | 2.1157E-014 |
| | | BMHDay2 | -2.94696E-014 | 3.65324E-014 | .427 | -1.0456E-013 | 4.5624E-014 |
| | | PMHDay2 | -1.24475E-014 | 3.44430E-014 | .721 | -8.3246E-014 | 5.8351E-014 |
| | BFHDay2 | BFHDay1 | -3.66939E-014 | 3.65324E-014 | .324 | -1.1179E-013 | 3.8399E-014 |
| | | PFHDay1 | -8.95975E-016 | 3.85085E-014 | .982 | -8.0051E-014 | 7.8259E-014 |
| | | BMHDay1 | 2.36901E-014 | 4.71631E-014 | .620 | -7.3255E-014 | 1.2064E-013 |
| | | PMHDay1 | 3.67298E-014 | 3.65324E-014 | .324 | -3.8364E-014 | 1.1182E-013 |
| | | PFHDay2 | -1.29121E-014 | 3.65324E-014 | .727 | -8.8005E-014 | 6.2181E-014 |
| | | BMHDay2 | 7.26027E-015 | 3.85085E-014 | .852 | -7.1895E-014 | 8.6416E-014 |
| | | PMHDay2 | 2.42824E-014 | 3.65324E-014 | .512 | -5.0811E-014 | 9.9376E-014 |
| | PFHDay2 | BFHDay1 | -2.37817E-014 | 3.44430E-014 | .496 | -9.4580E-014 | 4.7017E-014 |
| | | PFHDay1 | 1.20162E-014 | 3.65324E-014 | .745 | -6.3077E-014 | 8.7110E-014 |
| | | BMHDay1 | 3.66022E-014 | 4.55639E-014 | .429 | -5.7056E-014 | 1.3026E-013 |
| | | PMHDay1 | 4.96420E-014 | 3.44430E-014 | .161 | -2.1157E-014 | 1.2044E-013 |
| | | BFHDay2 | 1.29121E-014 | 3.65324E-014 | .727 | -6.2181E-014 | 8.8005E-014 |
| | | BMHDay2 | 2.01724E-014 | 3.65324E-014 | .586 | -5.4921E-014 | 9.5266E-014 |
| | | PMHDay2 | 3.71945E-014 | 3.44430E-014 | .290 | -3.3604E-014 | 1.0799E-013 |
| | BMHDay2 | BFHDay1 | -4.39541E-014 | 3.65324E-014 | .240 | -1.1905E-013 | 3.1139E-014 |
| | | PFHDay1 | -8.15625E-015 | 3.85085E-014 | .834 | -8.7312E-014 | 7.0999E-014 |

| | | | | | | | |
|---|---|---|---|---|---|---|---|
| | | BMHDay1 | 1.64298E-014 | 4.71631E-014 | .730 | -8.0515E-014 | 1.1337E-013 |
| | | PMHDay1 | 2.94696E-014 | 3.65324E-014 | .427 | -4.5624E-014 | 1.0456E-013 |
| | | BFHDay2 | -7.26027E-015 | 3.85085E-014 | .852 | -8.6416E-014 | 7.1895E-014 |
| | | PFHDay2 | -2.01724E-014 | 3.65324E-014 | .586 | -9.5266E-014 | 5.4921E-014 |
| | | PMHDay2 | 1.70221E-014 | 3.65324E-014 | .645 | -5.8071E-014 | 9.2115E-014 |
| | PMHDay2 | BFHDay1 | -6.09762E-014 | 3.44430E-014 | .088 | -1.3177E-013 | 9.8225E-015 |
| | | PFHDay1 | -2.51783E-014 | 3.65324E-014 | .497 | -1.0027E-013 | 4.9915E-014 |
| | | BMHDay1 | -5.92300E-016 | 4.55639E-014 | .990 | -9.4250E-014 | 9.3066E-014 |
| | | PMHDay1 | 1.24475E-014 | 3.44430E-014 | .721 | -5.8351E-014 | 8.3246E-014 |
| | | BFHDay2 | -2.42824E-014 | 3.65324E-014 | .512 | -9.9376E-014 | 5.0811E-014 |
| | | PFHDay2 | -3.71945E-014 | 3.44430E-014 | .290 | -1.0799E-013 | 3.3604E-014 |
| | | BMHDay2 | -1.70221E-014 | 3.65324E-014 | .645 | -9.2115E-014 | 5.8071E-014 |
| LinGly | BFHDay1 | PFHDay1 | 2.29600E-014 | 1.29907E-013 | .861 | -2.4165E-013 | 2.8757E-013 |
| | | BMHDay1 | 1.86636E-013 | 1.29907E-013 | .161 | -7.7976E-014 | 4.5125E-013 |
| | | PMHDay1 | -1.54977E-013 | 1.29907E-013 | .242 | -4.1959E-013 | 1.0964E-013 |
| | | BFHDay2 | -6.83546E-015 | 1.29907E-013 | .958 | -2.7145E-013 | 2.5778E-013 |
| | | PFHDay2 | -1.05009E-013 | 1.29907E-013 | .425 | -3.6962E-013 | 1.5960E-013 |
| | | BMHDay2 | -6.65666E-014 | 1.29907E-013 | .612 | -3.3118E-013 | 1.9805E-013 |
| | | PMHDay2 | -5.48847E-013* | 1.29907E-013 | <.001 | -8.1346E-013 | -2.8424E-013 |

| | | | | | | |
|---|---|---|---|---|---|---|
| PFHDay1 | BFHDay1 | -2.29600E-014 | 1.29907E-013 | .861 | -2.8757E-013 | 2.4165E-013 |
| | BMHDay1 | 1.63676E-013 | 1.29907E-013 | .217 | -1.0094E-013 | 4.2829E-013 |
| | PMHDay1 | -1.77937E-013 | 1.29907E-013 | .180 | -4.4255E-013 | 8.6675E-014 |
| | BFHDay2 | -2.97955E-014 | 1.29907E-013 | .820 | -2.9441E-013 | 2.3482E-013 |
| | PFHDay2 | -1.27969E-013 | 1.29907E-013 | .332 | -3.9258E-013 | 1.3664E-013 |
| | BMHDay2 | -8.95266E-014 | 1.29907E-013 | .496 | -3.5414E-013 | 1.7509E-013 |
| | PMHDay2 | -5.71807E-013* | 1.29907E-013 | <.001 | -8.3642E-013 | -3.0720E-013 |
| BMHDay1 | BFHDay1 | -1.86636E-013 | 1.29907E-013 | .161 | -4.5125E-013 | 7.7976E-014 |
| | PFHDay1 | -1.63676E-013 | 1.29907E-013 | .217 | -4.2829E-013 | 1.0094E-013 |
| | PMHDay1 | -3.41613E-013* | 1.29907E-013 | .013 | -6.0623E-013 | -7.7001E-014 |
| | BFHDay2 | -1.93472E-013 | 1.29907E-013 | .146 | -4.5808E-013 | 7.1141E-014 |
| | PFHDay2 | -2.91645E-013* | 1.29907E-013 | .032 | -5.5626E-013 | -2.7033E-014 |
| | BMHDay2 | -2.53203E-013 | 1.29907E-013 | .060 | -5.1781E-013 | 1.1410E-014 |
| | PMHDay2 | -7.35484E-013* | 1.29907E-013 | <.001 | -1.0001E-012 | -4.7087E-013 |
| PMHDay1 | BFHDay1 | 1.54977E-013 | 1.29907E-013 | .242 | -1.0964E-013 | 4.1959E-013 |
| | PFHDay1 | 1.77937E-013 | 1.29907E-013 | .180 | -8.6675E-014 | 4.4255E-013 |
| | BMHDay1 | 3.41613E-013* | 1.29907E-013 | .013 | 7.7001E-014 | 6.0623E-013 |
| | BFHDay2 | 1.48141E-013 | 1.29907E-013 | .263 | -1.1647E-013 | 4.1275E-013 |
| | PFHDay2 | 4.99676E-014 | 1.29907E-013 | .703 | -2.1464E-013 | 3.1458E-013 |

| | | | | | | |
|---|---|---|---|---|---|---|
| | BMHDay2 | 8.84102E-014 | 1.29907E-013 | .501 | -1.7620E-013 | 3.5302E-013 |
| | PMHDay2 | -3.93871E-013* | 1.29907E-013 | .005 | -6.5848E-013 | -1.2926E-013 |
| BFHDay2 | BFHDay1 | 6.83546E-015 | 1.29907E-013 | .958 | -2.5778E-013 | 2.7145E-013 |
| | PFHDay1 | 2.97955E-014 | 1.29907E-013 | .820 | -2.3482E-013 | 2.9441E-013 |
| | BMHDay1 | 1.93472E-013 | 1.29907E-013 | .146 | -7.1141E-014 | 4.5808E-013 |
| | PMHDay1 | -1.48141E-013 | 1.29907E-013 | .263 | -4.1275E-013 | 1.1647E-013 |
| | PFHDay2 | -9.81737E-014 | 1.29907E-013 | .455 | -3.6279E-013 | 1.6644E-013 |
| | BMHDay2 | -5.97311E-014 | 1.29907E-013 | .649 | -3.2434E-013 | 2.0488E-013 |
| | PMHDay2 | -5.42012E-013* | 1.29907E-013 | <.001 | -8.0662E-013 | -2.7740E-013 |
| PFHDay2 | BFHDay1 | 1.05009E-013 | 1.29907E-013 | .425 | -1.5960E-013 | 3.6962E-013 |
| | PFHDay1 | 1.27969E-013 | 1.29907E-013 | .332 | -1.3664E-013 | 3.9258E-013 |
| | BMHDay1 | 2.91645E-013* | 1.29907E-013 | .032 | 2.7033E-014 | 5.5626E-013 |
| | PMHDay1 | -4.99676E-014 | 1.29907E-013 | .703 | -3.1458E-013 | 2.1464E-013 |
| | BFHDay2 | 9.81737E-014 | 1.29907E-013 | .455 | -1.6644E-013 | 3.6279E-013 |
| | BMHDay2 | 3.84426E-014 | 1.29907E-013 | .769 | -2.2617E-013 | 3.0305E-013 |
| | PMHDay2 | -4.43838E-013* | 1.29907E-013 | .002 | -7.0845E-013 | -1.7923E-013 |
| BMHDay2 | BFHDay1 | 6.65666E-014 | 1.29907E-013 | .612 | -1.9805E-013 | 3.3118E-013 |
| | PFHDay1 | 8.95266E-014 | 1.29907E-013 | .496 | -1.7509E-013 | 3.5414E-013 |
| | BMHDay1 | 2.53203E-013 | 1.29907E-013 | .060 | -1.1410E-014 | 5.1781E-013 |

| | | | | | | | |
|---|---|---|---|---|---|---|---|
| | | PMHDay1 | -8.84102E-014 | 1.29907E-013 | .501 | -3.5302E-013 | 1.7620E-013 |
| | | BFHDay2 | 5.97311E-014 | 1.29907E-013 | .649 | -2.0488E-013 | 3.2434E-013 |
| | | PFHDay2 | -3.84426E-014 | 1.29907E-013 | .769 | -3.0305E-013 | 2.2617E-013 |
| | | PMHDay2 | -4.82281E-013* | 1.29907E-013 | <.001 | -7.4689E-013 | -2.1767E-013 |
| | PMHDay2 | BFHDay1 | 5.48847E-013* | 1.29907E-013 | <.001 | 2.8424E-013 | 8.1346E-013 |
| | | PFHDay1 | 5.71807E-013* | 1.29907E-013 | <.001 | 3.0720E-013 | 8.3642E-013 |
| | | BMHDay1 | 7.35484E-013* | 1.29907E-013 | <.001 | 4.7087E-013 | 1.0001E-012 |
| | | PMHDay1 | 3.93871E-013* | 1.29907E-013 | .005 | 1.2926E-013 | 6.5848E-013 |
| | | BFHDay2 | 5.42012E-013* | 1.29907E-013 | <.001 | 2.7740E-013 | 8.0662E-013 |
| | | PFHDay2 | 4.43838E-013* | 1.29907E-013 | .002 | 1.7923E-013 | 7.0845E-013 |
| | | BMHDay2 | 4.82281E-013* | 1.29907E-013 | <.001 | 2.1767E-013 | 7.4689E-013 |
| PalGly | BFHDay1 | PFHDay1 | -1.69048E-013 | 1.26870E-013 | .192 | -4.2747E-013 | 8.9377E-014 |
| | | BMHDay1 | 1.63787E-013 | 1.26870E-013 | .206 | -9.4638E-014 | 4.2221E-013 |
| | | PMHDay1 | -8.00892E-014 | 1.26870E-013 | .532 | -3.3851E-013 | 1.7834E-013 |
| | | BFHDay2 | -2.81420E-015 | 1.26870E-013 | .982 | -2.6124E-013 | 2.5561E-013 |
| | | PFHDay2 | -9.92324E-014 | 1.26870E-013 | .440 | -3.5766E-013 | 1.5919E-013 |
| | | BMHDay2 | 3.81386E-014 | 1.26870E-013 | .766 | -2.2029E-013 | 2.9656E-013 |
| | | PMHDay2 | -2.40629E-013 | 1.26870E-013 | .067 | -4.9905E-013 | 1.7796E-014 |
| | PFHDay1 | BFHDay1 | 1.69048E-013 | 1.26870E-013 | .192 | -8.9377E-014 | 4.2747E-013 |

| | | | | | | |
|---|---|---|---|---|---|---|
| | BMHDay1 | 3.32835E-013* | 1.26870E-013 | .013 | 7.4410E-014 | 5.9126E-013 |
| | PMHDay1 | 8.89584E-014 | 1.26870E-013 | .488 | -1.6947E-013 | 3.4738E-013 |
| | BFHDay2 | 1.66233E-013 | 1.26870E-013 | .199 | -9.2191E-014 | 4.2466E-013 |
| | PFHDay2 | 6.98152E-014 | 1.26870E-013 | .586 | -1.8861E-013 | 3.2824E-013 |
| | BMHDay2 | 2.07186E-013 | 1.26870E-013 | .112 | -5.1239E-014 | 4.6561E-013 |
| | PMHDay2 | -7.15810E-014 | 1.26870E-013 | .577 | -3.3001E-013 | 1.8684E-013 |
| BMHDay1 | BFHDay1 | -1.63787E-013 | 1.26870E-013 | .206 | -4.2221E-013 | 9.4638E-014 |
| | PFHDay1 | -3.32835E-013* | 1.26870E-013 | .013 | -5.9126E-013 | -7.4410E-014 |
| | PMHDay1 | -2.43876E-013 | 1.26870E-013 | .064 | -5.0230E-013 | 1.4549E-014 |
| | BFHDay2 | -1.66601E-013 | 1.26870E-013 | .198 | -4.2503E-013 | 9.1824E-014 |
| | PFHDay2 | -2.63019E-013* | 1.26870E-013 | .046 | -5.2144E-013 | -4.5946E-015 |
| | BMHDay2 | -1.25648E-013 | 1.26870E-013 | .329 | -3.8407E-013 | 1.3278E-013 |
| | PMHDay2 | -4.04416E-013* | 1.26870E-013 | .003 | -6.6284E-013 | -1.4599E-013 |
| PMHDay1 | BFHDay1 | 8.00892E-014 | 1.26870E-013 | .532 | -1.7834E-013 | 3.3851E-013 |
| | PFHDay1 | -8.89584E-014 | 1.26870E-013 | .488 | -3.4738E-013 | 1.6947E-013 |
| | BMHDay1 | 2.43876E-013 | 1.26870E-013 | .064 | -1.4549E-014 | 5.0230E-013 |
| | BFHDay2 | 7.72750E-014 | 1.26870E-013 | .547 | -1.8115E-013 | 3.3570E-013 |
| | PFHDay2 | -1.91432E-014 | 1.26870E-013 | .881 | -2.7757E-013 | 2.3928E-013 |
| | BMHDay2 | 1.18228E-013 | 1.26870E-013 | .358 | -1.4020E-013 | 3.7665E-013 |

| | | | | | | |
|---|---|---|---|---|---|---|
| | PMHDay2 | -1.60539E-013 | 1.26870E-013 | .215 | -4.1896E-013 | 9.7885E-014 |
| BFHDay2 | BFHDay1 | 2.81420E-015 | 1.26870E-013 | .982 | -2.5561E-013 | 2.6124E-013 |
| | PFHDay1 | -1.66233E-013 | 1.26870E-013 | .199 | -4.2466E-013 | 9.2191E-014 |
| | BMHDay1 | 1.66601E-013 | 1.26870E-013 | .198 | -9.1824E-014 | 4.2503E-013 |
| | PMHDay1 | -7.72750E-014 | 1.26870E-013 | .547 | -3.3570E-013 | 1.8115E-013 |
| | PFHDay2 | -9.64182E-014 | 1.26870E-013 | .453 | -3.5484E-013 | 1.6201E-013 |
| | BMHDay2 | 4.09528E-014 | 1.26870E-013 | .749 | -2.1747E-013 | 2.9938E-013 |
| | PMHDay2 | -2.37814E-013 | 1.26870E-013 | .070 | -4.9624E-013 | 2.0610E-014 |
| PFHDay2 | BFHDay1 | 9.92324E-014 | 1.26870E-013 | .440 | -1.5919E-013 | 3.5766E-013 |
| | PFHDay1 | -6.98152E-014 | 1.26870E-013 | .586 | -3.2824E-013 | 1.8861E-013 |
| | BMHDay1 | 2.63019E-013* | 1.26870E-013 | .046 | 4.5946E-015 | 5.2144E-013 |
| | PMHDay1 | 1.91432E-014 | 1.26870E-013 | .881 | -2.3928E-013 | 2.7757E-013 |
| | BFHDay2 | 9.64182E-014 | 1.26870E-013 | .453 | -1.6201E-013 | 3.5484E-013 |
| | BMHDay2 | 1.37371E-013 | 1.26870E-013 | .287 | -1.2105E-013 | 3.9580E-013 |
| | PMHDay2 | -1.41396E-013 | 1.26870E-013 | .273 | -3.9982E-013 | 1.1703E-013 |
| BMHDay2 | BFHDay1 | -3.81386E-014 | 1.26870E-013 | .766 | -2.9656E-013 | 2.2029E-013 |
| | PFHDay1 | -2.07186E-013 | 1.26870E-013 | .112 | -4.6561E-013 | 5.1239E-014 |
| | BMHDay1 | 1.25648E-013 | 1.26870E-013 | .329 | -1.3278E-013 | 3.8407E-013 |
| | PMHDay1 | -1.18228E-013 | 1.26870E-013 | .358 | -3.7665E-013 | 1.4020E-013 |

| | | | | | | | |
|---|---|---|---|---|---|---|---|
| | | BFHDay2 | -4.09528E-014 | 1.26870E-013 | .749 | -2.9938E-013 | 2.1747E-013 |
| | | PFHDay2 | -1.37371E-013 | 1.26870E-013 | .287 | -3.9580E-013 | 1.2105E-013 |
| | | PMHDay2 | -2.78767E-013* | 1.26870E-013 | .035 | -5.3719E-013 | -2.0342E-014 |
| | PMHDay2 | BFHDay1 | 2.40629E-013 | 1.26870E-013 | .067 | -1.7796E-014 | 4.9905E-013 |
| | | PFHDay1 | 7.15810E-014 | 1.26870E-013 | .577 | -1.8684E-013 | 3.3001E-013 |
| | | BMHDay1 | 4.04416E-013* | 1.26870E-013 | .003 | 1.4599E-013 | 6.6284E-013 |
| | | PMHDay1 | 1.60539E-013 | 1.26870E-013 | .215 | -9.7885E-014 | 4.1896E-013 |
| | | BFHDay2 | 2.37814E-013 | 1.26870E-013 | .070 | -2.0610E-014 | 4.9624E-013 |
| | | PFHDay2 | 1.41396E-013 | 1.26870E-013 | .273 | -1.1703E-013 | 3.9982E-013 |
| | | BMHDay2 | 2.78767E-013* | 1.26870E-013 | .035 | 2.0342E-014 | 5.3719E-013 |
| OlGly | BFHDay1 | PFHDay1 | -1.49552E-014 | 7.38132E-014 | .841 | -1.6531E-013 | 1.3540E-013 |
| | | BMHDay1 | 5.07179E-014 | 7.38132E-014 | .497 | -9.9635E-014 | 2.0107E-013 |
| | | PMHDay1 | -1.04112E-013 | 7.38132E-014 | .168 | -2.5446E-013 | 4.6240E-014 |
| | | BFHDay2 | -1.16794E-015 | 7.38132E-014 | .987 | -1.5152E-013 | 1.4918E-013 |
| | | PFHDay2 | -7.04268E-014 | 7.38132E-014 | .347 | -2.2078E-013 | 7.9926E-014 |
| | | BMHDay2 | -3.78638E-014 | 7.38132E-014 | .611 | -1.8822E-013 | 1.1249E-013 |
| | | PMHDay2 | -2.24166E-013* | 7.38132E-014 | .005 | -3.7452E-013 | -7.3814E-014 |
| | PFHDay1 | BFHDay1 | 1.49552E-014 | 7.38132E-014 | .841 | -1.3540E-013 | 1.6531E-013 |
| | | BMHDay1 | 6.56731E-014 | 7.38132E-014 | .380 | -8.4679E-014 | 2.1603E-013 |

| | | | | | | |
|---|---|---|---|---|---|---|
| | PMHDay1 | -8.91570E-014 | 7.38132E-014 | .236 | -2.3951E-013 | 6.1196E-014 |
| | BFHDay2 | 1.37873E-014 | 7.38132E-014 | .853 | -1.3657E-013 | 1.6414E-013 |
| | PFHDay2 | -5.54716E-014 | 7.38132E-014 | .458 | -2.0582E-013 | 9.4881E-014 |
| | BMHDay2 | -2.29086E-014 | 7.38132E-014 | .758 | -1.7326E-013 | 1.2744E-013 |
| | PMHDay2 | -2.09211E-013* | 7.38132E-014 | .008 | -3.5956E-013 | -5.8859E-014 |
| BMHDay1 | BFHDay1 | -5.07179E-014 | 7.38132E-014 | .497 | -2.0107E-013 | 9.9635E-014 |
| | PFHDay1 | -6.56731E-014 | 7.38132E-014 | .380 | -2.1603E-013 | 8.4679E-014 |
| | PMHDay1 | -1.54830E-013* | 7.38132E-014 | .044 | -3.0518E-013 | -4.4775E-015 |
| | BFHDay2 | -5.18858E-014 | 7.38132E-014 | .487 | -2.0224E-013 | 9.8467E-014 |
| | PFHDay2 | -1.21145E-013 | 7.38132E-014 | .111 | -2.7150E-013 | 2.9208E-014 |
| | BMHDay2 | -8.85817E-014 | 7.38132E-014 | .239 | -2.3893E-013 | 6.1771E-014 |
| | PMHDay2 | -2.74884E-013* | 7.38132E-014 | <.001 | -4.2524E-013 | -1.2453E-013 |
| PMHDay1 | BFHDay1 | 1.04112E-013 | 7.38132E-014 | .168 | -4.6240E-014 | 2.5446E-013 |
| | PFHDay1 | 8.91570E-014 | 7.38132E-014 | .236 | -6.1196E-014 | 2.3951E-013 |
| | BMHDay1 | 1.54830E-013* | 7.38132E-014 | .044 | 4.4775E-015 | 3.0518E-013 |
| | BFHDay2 | 1.02944E-013 | 7.38132E-014 | .173 | -4.7408E-014 | 2.5330E-013 |
| | PFHDay2 | 3.36854E-014 | 7.38132E-014 | .651 | -1.1667E-013 | 1.8404E-013 |
| | BMHDay2 | 6.62484E-014 | 7.38132E-014 | .376 | -8.4104E-014 | 2.1660E-013 |
| | PMHDay2 | -1.20054E-013 | 7.38132E-014 | .114 | -2.7041E-013 | 3.0298E-014 |

| | | | | | | |
|---|---|---|---|---|---|---|
| | BFHDay2 | BFHDay1 | 1.16794E-015 | 7.38132E-014 | .987 | -1.4918E-013 | 1.5152E-013 |
| | | PFHDay1 | -1.37873E-014 | 7.38132E-014 | .853 | -1.6414E-013 | 1.3657E-013 |
| | | BMHDay1 | 5.18858E-014 | 7.38132E-014 | .487 | -9.8467E-014 | 2.0224E-013 |
| | | PMHDay1 | -1.02944E-013 | 7.38132E-014 | .173 | -2.5330E-013 | 4.7408E-014 |
| | | PFHDay2 | -6.92589E-014 | 7.38132E-014 | .355 | -2.1961E-013 | 8.1094E-014 |
| | | BMHDay2 | -3.66959E-014 | 7.38132E-014 | .622 | -1.8705E-013 | 1.1366E-013 |
| | | PMHDay2 | -2.22998E-013* | 7.38132E-014 | .005 | -3.7335E-013 | -7.2646E-014 |
| | PFHDay2 | BFHDay1 | 7.04268E-014 | 7.38132E-014 | .347 | -7.9926E-014 | 2.2078E-013 |
| | | PFHDay1 | 5.54716E-014 | 7.38132E-014 | .458 | -9.4881E-014 | 2.0582E-013 |
| | | BMHDay1 | 1.21145E-013 | 7.38132E-014 | .111 | -2.9208E-014 | 2.7150E-013 |
| | | PMHDay1 | -3.36854E-014 | 7.38132E-014 | .651 | -1.8404E-013 | 1.1667E-013 |
| | | BFHDay2 | 6.92589E-014 | 7.38132E-014 | .355 | -8.1094E-014 | 2.1961E-013 |
| | | BMHDay2 | 3.25630E-014 | 7.38132E-014 | .662 | -1.1779E-013 | 1.8292E-013 |
| | | PMHDay2 | -1.53740E-013* | 7.38132E-014 | .045 | -3.0409E-013 | -3.3870E-015 |
| | BMHDay2 | BFHDay1 | 3.78638E-014 | 7.38132E-014 | .611 | -1.1249E-013 | 1.8822E-013 |
| | | PFHDay1 | 2.29086E-014 | 7.38132E-014 | .758 | -1.2744E-013 | 1.7326E-013 |
| | | BMHDay1 | 8.85817E-014 | 7.38132E-014 | .239 | -6.1771E-014 | 2.3893E-013 |
| | | PMHDay1 | -6.62484E-014 | 7.38132E-014 | .376 | -2.1660E-013 | 8.4104E-014 |
| | | BFHDay2 | 3.66959E-014 | 7.38132E-014 | .622 | -1.1366E-013 | 1.8705E-013 |

| | | | | | | | |
|---|---|---|---|---|---|---|---|
| | | PFHDay2 | -3.25630E-014 | 7.38132E-014 | .662 | -1.8292E-013 | 1.1779E-013 |
| | | PMHDay2 | -1.86303E-013* | 7.38132E-014 | .017 | -3.3666E-013 | -3.5950E-014 |
| | PMHDay2 | BFHDay1 | 2.24166E-013* | 7.38132E-014 | .005 | 7.3814E-014 | 3.7452E-013 |
| | | PFHDay1 | 2.09211E-013* | 7.38132E-014 | .008 | 5.8859E-014 | 3.5956E-013 |
| | | BMHDay1 | 2.74884E-013* | 7.38132E-014 | <.001 | 1.2453E-013 | 4.2524E-013 |
| | | PMHDay1 | 1.20054E-013 | 7.38132E-014 | .114 | -3.0298E-014 | 2.7041E-013 |
| | | BFHDay2 | 2.22998E-013* | 7.38132E-014 | .005 | 7.2646E-014 | 3.7335E-013 |
| | | PFHDay2 | 1.53740E-013* | 7.38132E-014 | .045 | 3.3870E-015 | 3.0409E-013 |
| | | BMHDay2 | 1.86303E-013* | 7.38132E-014 | .017 | 3.5950E-014 | 3.3666E-013 |
| DocGly | BFHDay1 | PFHDay1 | -1.25978E-015* | 4.61243E-016 | .013 | -2.2190E-015 | -3.0057E-016 |
| | | BMHDay1 | 2.52441E-017 | 4.61243E-016 | .957 | -9.3396E-016 | 9.8445E-016 |
| | | PMHDay1 | 9.31104E-017 | 4.61243E-016 | .842 | -8.6610E-016 | 1.0523E-015 |
| | | BFHDay2 | -2.95329E-016 | 3.99448E-016 | .468 | -1.1260E-015 | 5.3537E-016 |
| | | PFHDay2 | -6.53106E-017 | 4.23678E-016 | .879 | -9.4640E-016 | 8.1578E-016 |
| | | BMHDay2 | -3.33031E-016 | 5.28420E-016 | .535 | -1.4319E-015 | 7.6588E-016 |
| | | PMHDay2 | 2.72994E-016 | 4.23678E-016 | .526 | -6.0809E-016 | 1.1541E-015 |
| | PFHDay1 | BFHDay1 | 1.25978E-015* | 4.61243E-016 | .013 | 3.0057E-016 | 2.2190E-015 |
| | | BMHDay1 | 1.28502E-015* | 5.15685E-016 | .021 | 2.1260E-016 | 2.3574E-015 |
| | | PMHDay1 | 1.35289E-015* | 5.15685E-016 | .016 | 2.8046E-016 | 2.4253E-015 |

| | | | | | | |
|---|---|---|---|---|---|---|
| | BFHDay2 | 9.64449E-016* | 4.61243E-016 | .049 | 5.2423E-018 | 1.9237E-015 |
| | PFHDay2 | 1.19447E-015* | 4.82379E-016 | .022 | 1.9131E-016 | 2.1976E-015 |
| | BMHDay2 | 9.26747E-016 | 5.76553E-016 | .123 | -2.7226E-016 | 2.1258E-015 |
| | PMHDay2 | 1.53277E-015* | 4.82379E-016 | .005 | 5.2961E-016 | 2.5359E-015 |
| BMHDay1 | BFHDay1 | -2.52441E-017 | 4.61243E-016 | .957 | -9.8445E-016 | 9.3396E-016 |
| | PFHDay1 | -1.28502E-015* | 5.15685E-016 | .021 | -2.3574E-015 | -2.1260E-016 |
| | PMHDay1 | 6.78663E-017 | 5.15685E-016 | .897 | -1.0046E-015 | 1.1403E-015 |
| | BFHDay2 | -3.20573E-016 | 4.61243E-016 | .495 | -1.2798E-015 | 6.3863E-016 |
| | PFHDay2 | -9.05547E-017 | 4.82379E-016 | .853 | -1.0937E-015 | 9.1261E-016 |
| | BMHDay2 | -3.58275E-016 | 5.76553E-016 | .541 | -1.5573E-015 | 8.4073E-016 |
| | PMHDay2 | 2.47750E-016 | 4.82379E-016 | .613 | -7.5541E-016 | 1.2509E-015 |
| PMHDay1 | BFHDay1 | -9.31104E-017 | 4.61243E-016 | .842 | -1.0523E-015 | 8.6610E-016 |
| | PFHDay1 | -1.35289E-015* | 5.15685E-016 | .016 | -2.4253E-015 | -2.8046E-016 |
| | BMHDay1 | -6.78663E-017 | 5.15685E-016 | .897 | -1.1403E-015 | 1.0046E-015 |
| | BFHDay2 | -3.88439E-016 | 4.61243E-016 | .409 | -1.3476E-015 | 5.7077E-016 |
| | PFHDay2 | -1.58421E-016 | 4.82379E-016 | .746 | -1.1616E-015 | 8.4474E-016 |
| | BMHDay2 | -4.26141E-016 | 5.76553E-016 | .468 | -1.6251E-015 | 7.7287E-016 |
| | PMHDay2 | 1.79884E-016 | 4.82379E-016 | .713 | -8.2328E-016 | 1.1830E-015 |
| BFHDay2 | BFHDay1 | 2.95329E-016 | 3.99448E-016 | .468 | -5.3537E-016 | 1.1260E-015 |

| | | | | | | |
|---|---|---|---|---|---|---|
| | PFHDay1 | -9.64449E-016* | 4.61243E-016 | .049 | -1.9237E-015 | -5.2423E-018 |
| | BMHDay1 | 3.20573E-016 | 4.61243E-016 | .495 | -6.3863E-016 | 1.2798E-015 |
| | PMHDay1 | 3.88439E-016 | 4.61243E-016 | .409 | -5.7077E-016 | 1.3476E-015 |
| | PFHDay2 | 2.30018E-016 | 4.23678E-016 | .593 | -6.5107E-016 | 1.1111E-015 |
| | BMHDay2 | -3.77016E-017 | 5.28420E-016 | .944 | -1.1366E-015 | 1.0612E-015 |
| | PMHDay2 | 5.68323E-016 | 4.23678E-016 | .194 | -3.1276E-016 | 1.4494E-015 |
| PFHDay2 | BFHDay1 | 6.53106E-017 | 4.23678E-016 | .879 | -8.1578E-016 | 9.4640E-016 |
| | PFHDay1 | -1.19447E-015* | 4.82379E-016 | .022 | -2.1976E-015 | -1.9131E-016 |
| | BMHDay1 | 9.05547E-017 | 4.82379E-016 | .853 | -9.1261E-016 | 1.0937E-015 |
| | PMHDay1 | 1.58421E-016 | 4.82379E-016 | .746 | -8.4474E-016 | 1.1616E-015 |
| | BFHDay2 | -2.30018E-016 | 4.23678E-016 | .593 | -1.1111E-015 | 6.5107E-016 |
| | BMHDay2 | -2.67720E-016 | 5.46967E-016 | .630 | -1.4052E-015 | 8.6976E-016 |
| | PMHDay2 | 3.38305E-016 | 4.46596E-016 | .457 | -5.9044E-016 | 1.2671E-015 |
| BMHDay2 | BFHDay1 | 3.33031E-016 | 5.28420E-016 | .535 | -7.6588E-016 | 1.4319E-015 |
| | PFHDay1 | -9.26747E-016 | 5.76553E-016 | .123 | -2.1258E-015 | 2.7226E-016 |
| | BMHDay1 | 3.58275E-016 | 5.76553E-016 | .541 | -8.4073E-016 | 1.5573E-015 |
| | PMHDay1 | 4.26141E-016 | 5.76553E-016 | .468 | -7.7287E-016 | 1.6251E-015 |
| | BFHDay2 | 3.77016E-017 | 5.28420E-016 | .944 | -1.0612E-015 | 1.1366E-015 |
| | PFHDay2 | 2.67720E-016 | 5.46967E-016 | .630 | -8.6976E-016 | 1.4052E-015 |

| | | | | | | | |
|---|---|---|---|---|---|---|---|
| | | PMHDay2 | 6.06025E-016 | 5.46967E-016 | .280 | -5.3145E-016 | 1.7435E-015 |
| | PMHDay2 | BFHDay1 | -2.72994E-016 | 4.23678E-016 | .526 | -1.1541E-015 | 6.0809E-016 |
| | | PFHDay1 | -1.53277E-015* | 4.82379E-016 | .005 | -2.5359E-015 | -5.2961E-016 |
| | | BMHDay1 | -2.47750E-016 | 4.82379E-016 | .613 | -1.2509E-015 | 7.5541E-016 |
| | | PMHDay1 | -1.79884E-016 | 4.82379E-016 | .713 | -1.1830E-015 | 8.2328E-016 |
| | | BFHDay2 | -5.68323E-016 | 4.23678E-016 | .194 | -1.4494E-015 | 3.1276E-016 |
| | | PFHDay2 | -3.38305E-016 | 4.46596E-016 | .457 | -1.2671E-015 | 5.9044E-016 |
| | | BMHDay2 | -6.06025E-016 | 5.46967E-016 | .280 | -1.7435E-015 | 5.3145E-016 |
| StrGly | BFHDay1 | PFHDay1 | 3.02712E-014 | 2.08056E-013 | .885 | -3.9352E-013 | 4.5407E-013 |
| | | BMHDay1 | 3.58647E-013 | 2.08056E-013 | .094 | -6.5148E-014 | 7.8244E-013 |
| | | PMHDay1 | -9.22766E-014 | 2.08056E-013 | .660 | -5.1607E-013 | 3.3152E-013 |
| | | BFHDay2 | -4.77492E-014 | 2.08056E-013 | .820 | -4.7154E-013 | 3.7605E-013 |
| | | PFHDay2 | 5.04080E-015 | 2.08056E-013 | .981 | -4.1875E-013 | 4.2884E-013 |
| | | BMHDay2 | 1.94437E-013 | 2.08056E-013 | .357 | -2.2936E-013 | 6.1823E-013 |
| | | PMHDay2 | -2.67730E-013 | 2.08056E-013 | .207 | -6.9153E-013 | 1.5607E-013 |
| | PFHDay1 | BFHDay1 | -3.02712E-014 | 2.08056E-013 | .885 | -4.5407E-013 | 3.9352E-013 |
| | | BMHDay1 | 3.28376E-013 | 2.08056E-013 | .124 | -9.5419E-014 | 7.5217E-013 |
| | | PMHDay1 | -1.22548E-013 | 2.08056E-013 | .560 | -5.4634E-013 | 3.0125E-013 |
| | | BFHDay2 | -7.80204E-014 | 2.08056E-013 | .710 | -5.0182E-013 | 3.4578E-013 |

| | | | | | | |
|---|---|---|---|---|---|---|
| | PFHDay2 | -2.52304E-014 | 2.08056E-013 | .904 | -4.4903E-013 | 3.9857E-013 |
| | BMHDay2 | 1.64166E-013 | 2.08056E-013 | .436 | -2.5963E-013 | 5.8796E-013 |
| | PMHDay2 | -2.98002E-013 | 2.08056E-013 | .162 | -7.2180E-013 | 1.2579E-013 |
| BMHDay1 | BFHDay1 | -3.58647E-013 | 2.08056E-013 | .094 | -7.8244E-013 | 6.5148E-014 |
| | PFHDay1 | -3.28376E-013 | 2.08056E-013 | .124 | -7.5217E-013 | 9.5419E-014 |
| | PMHDay1 | -4.50924E-013* | 2.08056E-013 | .038 | -8.7472E-013 | -2.7128E-014 |
| | BFHDay2 | -4.06396E-013 | 2.08056E-013 | .060 | -8.3019E-013 | 1.7399E-014 |
| | PFHDay2 | -3.53606E-013 | 2.08056E-013 | .099 | -7.7740E-013 | 7.0189E-014 |
| | BMHDay2 | -1.64210E-013 | 2.08056E-013 | .436 | -5.8801E-013 | 2.5959E-013 |
| | PMHDay2 | -6.26378E-013* | 2.08056E-013 | .005 | -1.0502E-012 | -2.0258E-013 |
| PMHDay1 | BFHDay1 | 9.22766E-014 | 2.08056E-013 | .660 | -3.3152E-013 | 5.1607E-013 |
| | PFHDay1 | 1.22548E-013 | 2.08056E-013 | .560 | -3.0125E-013 | 5.4634E-013 |
| | BMHDay1 | 4.50924E-013* | 2.08056E-013 | .038 | 2.7128E-014 | 8.7472E-013 |
| | BFHDay2 | 4.45274E-014 | 2.08056E-013 | .832 | -3.7927E-013 | 4.6832E-013 |
| | PFHDay2 | 9.73174E-014 | 2.08056E-013 | .643 | -3.2648E-013 | 5.2111E-013 |
| | BMHDay2 | 2.86714E-013 | 2.08056E-013 | .178 | -1.3708E-013 | 7.1051E-013 |
| | PMHDay2 | -1.75454E-013 | 2.08056E-013 | .405 | -5.9925E-013 | 2.4834E-013 |
| BFHDay2 | BFHDay1 | 4.77492E-014 | 2.08056E-013 | .820 | -3.7605E-013 | 4.7154E-013 |
| | PFHDay1 | 7.80204E-014 | 2.08056E-013 | .710 | -3.4578E-013 | 5.0182E-013 |

| | | | | | | |
|---|---|---|---|---|---|---|
| | BMHDay1 | 4.06396E-013 | 2.08056E-013 | .060 | -1.7399E-014 | 8.3019E-013 |
| | PMHDay1 | -4.45274E-014 | 2.08056E-013 | .832 | -4.6832E-013 | 3.7927E-013 |
| | PFHDay2 | 5.27900E-014 | 2.08056E-013 | .801 | -3.7101E-013 | 4.7659E-013 |
| | BMHDay2 | 2.42186E-013 | 2.08056E-013 | .253 | -1.8161E-013 | 6.6598E-013 |
| | PMHDay2 | -2.19981E-013 | 2.08056E-013 | .298 | -6.4378E-013 | 2.0381E-013 |
| PFHDay2 | BFHDay1 | -5.04080E-015 | 2.08056E-013 | .981 | -4.2884E-013 | 4.1875E-013 |
| | PFHDay1 | 2.52304E-014 | 2.08056E-013 | .904 | -3.9857E-013 | 4.4903E-013 |
| | BMHDay1 | 3.53606E-013 | 2.08056E-013 | .099 | -7.0189E-014 | 7.7740E-013 |
| | PMHDay1 | -9.73174E-014 | 2.08056E-013 | .643 | -5.2111E-013 | 3.2648E-013 |
| | BFHDay2 | -5.27900E-014 | 2.08056E-013 | .801 | -4.7659E-013 | 3.7101E-013 |
| | BMHDay2 | 1.89396E-013 | 2.08056E-013 | .369 | -2.3440E-013 | 6.1319E-013 |
| | PMHDay2 | -2.72771E-013 | 2.08056E-013 | .199 | -6.9657E-013 | 1.5102E-013 |
| BMHDay2 | BFHDay1 | -1.94437E-013 | 2.08056E-013 | .357 | -6.1823E-013 | 2.2936E-013 |
| | PFHDay1 | -1.64166E-013 | 2.08056E-013 | .436 | -5.8796E-013 | 2.5963E-013 |
| | BMHDay1 | 1.64210E-013 | 2.08056E-013 | .436 | -2.5959E-013 | 5.8801E-013 |
| | PMHDay1 | -2.86714E-013 | 2.08056E-013 | .178 | -7.1051E-013 | 1.3708E-013 |
| | BFHDay2 | -2.42186E-013 | 2.08056E-013 | .253 | -6.6598E-013 | 1.8161E-013 |
| | PFHDay2 | -1.89396E-013 | 2.08056E-013 | .369 | -6.1319E-013 | 2.3440E-013 |
| | PMHDay2 | -4.62168E-013* | 2.08056E-013 | .034 | -8.8596E-013 | -3.8372E-014 |

| | | | | | | | |
|---|---|---|---|---|---|---|---|
| | PMHDay2 | BFHDay1 | 2.67730E-013 | 2.08056E-013 | .207 | -1.5607E-013 | 6.9153E-013 |
| | | PFHDay1 | 2.98002E-013 | 2.08056E-013 | .162 | -1.2579E-013 | 7.2180E-013 |
| | | BMHDay1 | 6.26378E-013* | 2.08056E-013 | .005 | 2.0258E-013 | 1.0502E-012 |
| | | PMHDay1 | 1.75454E-013 | 2.08056E-013 | .405 | -2.4834E-013 | 5.9925E-013 |
| | | BFHDay2 | 2.19981E-013 | 2.08056E-013 | .298 | -2.0381E-013 | 6.4378E-013 |
| | | PFHDay2 | 2.72771E-013 | 2.08056E-013 | .199 | -1.5102E-013 | 6.9657E-013 |
| | | BMHDay2 | 4.62168E-013* | 2.08056E-013 | .034 | 3.8372E-014 | 8.8596E-013 |
| PEA | BFHDay1 | PFHDay1 | -4.30334E-012 | 5.82064E-012 | .465 | -1.6160E-011 | 7.5529E-012 |
| | | BMHDay1 | -1.80730E-012 | 5.82064E-012 | .758 | -1.3664E-011 | 1.0049E-011 |
| | | PMHDay1 | -5.44206E-012 | 5.82064E-012 | .357 | -1.7298E-011 | 6.4142E-012 |
| | | BFHDay2 | -6.44076E-012 | 5.82064E-012 | .277 | -1.8297E-011 | 5.4155E-012 |
| | | PFHDay2 | -8.17086E-012 | 5.82064E-012 | .170 | -2.0027E-011 | 3.6854E-012 |
| | | BMHDay2 | -1.65062E-012 | 5.82064E-012 | .779 | -1.3507E-011 | 1.0206E-011 |
| | | PMHDay2 | -1.06697E-011 | 5.82064E-012 | .076 | -2.2526E-011 | 1.1865E-012 |
| | PFHDay1 | BFHDay1 | 4.30334E-012 | 5.82064E-012 | .465 | -7.5529E-012 | 1.6160E-011 |
| | | BMHDay1 | 2.49604E-012 | 5.82064E-012 | .671 | -9.3602E-012 | 1.4352E-011 |
| | | PMHDay1 | -1.13872E-012 | 5.82064E-012 | .846 | -1.2995E-011 | 1.0718E-011 |
| | | BFHDay2 | -2.13742E-012 | 5.82064E-012 | .716 | -1.3994E-011 | 9.7188E-012 |
| | | PFHDay2 | -3.86752E-012 | 5.82064E-012 | .511 | -1.5724E-011 | 7.9887E-012 |

| | | | | | | |
|---|---|---|---|---|---|---|
| | BMHDay2 | 2.65272E-012 | 5.82064E-012 | .652 | -9.2035E-012 | 1.4509E-011 |
| | PMHDay2 | -6.36640E-012 | 5.82064E-012 | .282 | -1.8223E-011 | 5.4899E-012 |
| BMHDay1 | BFHDay1 | 1.80730E-012 | 5.82064E-012 | .758 | -1.0049E-011 | 1.3664E-011 |
| | PFHDay1 | -2.49604E-012 | 5.82064E-012 | .671 | -1.4352E-011 | 9.3602E-012 |
| | PMHDay1 | -3.63476E-012 | 5.82064E-012 | .537 | -1.5491E-011 | 8.2215E-012 |
| | BFHDay2 | -4.63346E-012 | 5.82064E-012 | .432 | -1.6490E-011 | 7.2228E-012 |
| | PFHDay2 | -6.36356E-012 | 5.82064E-012 | .282 | -1.8220E-011 | 5.4927E-012 |
| | BMHDay2 | 1.56680E-013 | 5.82064E-012 | .979 | -1.1700E-011 | 1.2013E-011 |
| | PMHDay2 | -8.86244E-012 | 5.82064E-012 | .138 | -2.0719E-011 | 2.9938E-012 |
| PMHDay1 | BFHDay1 | 5.44206E-012 | 5.82064E-012 | .357 | -6.4142E-012 | 1.7298E-011 |
| | PFHDay1 | 1.13872E-012 | 5.82064E-012 | .846 | -1.0718E-011 | 1.2995E-011 |
| | BMHDay1 | 3.63476E-012 | 5.82064E-012 | .537 | -8.2215E-012 | 1.5491E-011 |
| | BFHDay2 | -9.98700E-013 | 5.82064E-012 | .865 | -1.2855E-011 | 1.0858E-011 |
| | PFHDay2 | -2.72880E-012 | 5.82064E-012 | .642 | -1.4585E-011 | 9.1275E-012 |
| | BMHDay2 | 3.79144E-012 | 5.82064E-012 | .519 | -8.0648E-012 | 1.5648E-011 |
| | PMHDay2 | -5.22768E-012 | 5.82064E-012 | .376 | -1.7084E-011 | 6.6286E-012 |
| BFHDay2 | BFHDay1 | 6.44076E-012 | 5.82064E-012 | .277 | -5.4155E-012 | 1.8297E-011 |
| | PFHDay1 | 2.13742E-012 | 5.82064E-012 | .716 | -9.7188E-012 | 1.3994E-011 |
| | BMHDay1 | 4.63346E-012 | 5.82064E-012 | .432 | -7.2228E-012 | 1.6490E-011 |

| | | | | | | | |
|---|---|---|---|---|---|---|---|
| | | PMHDay1 | 9.98700E-013 | 5.82064E-012 | .865 | -1.0858E-011 | 1.2855E-011 |
| | | PFHDay2 | -1.73010E-012 | 5.82064E-012 | .768 | -1.3586E-011 | 1.0126E-011 |
| | | BMHDay2 | 4.79014E-012 | 5.82064E-012 | .417 | -7.0661E-012 | 1.6646E-011 |
| | | PMHDay2 | -4.22898E-012 | 5.82064E-012 | .473 | -1.6085E-011 | 7.6273E-012 |
| | PFHDay2 | BFHDay1 | 8.17086E-012 | 5.82064E-012 | .170 | -3.6854E-012 | 2.0027E-011 |
| | | PFHDay1 | 3.86752E-012 | 5.82064E-012 | .511 | -7.9887E-012 | 1.5724E-011 |
| | | BMHDay1 | 6.36356E-012 | 5.82064E-012 | .282 | -5.4927E-012 | 1.8220E-011 |
| | | PMHDay1 | 2.72880E-012 | 5.82064E-012 | .642 | -9.1275E-012 | 1.4585E-011 |
| | | BFHDay2 | 1.73010E-012 | 5.82064E-012 | .768 | -1.0126E-011 | 1.3586E-011 |
| | | BMHDay2 | 6.52024E-012 | 5.82064E-012 | .271 | -5.3360E-012 | 1.8376E-011 |
| | | PMHDay2 | -2.49888E-012 | 5.82064E-012 | .671 | -1.4355E-011 | 9.3574E-012 |
| | BMHDay2 | BFHDay1 | 1.65062E-012 | 5.82064E-012 | .779 | -1.0206E-011 | 1.3507E-011 |
| | | PFHDay1 | -2.65272E-012 | 5.82064E-012 | .652 | -1.4509E-011 | 9.2035E-012 |
| | | BMHDay1 | -1.56680E-013 | 5.82064E-012 | .979 | -1.2013E-011 | 1.1700E-011 |
| | | PMHDay1 | -3.79144E-012 | 5.82064E-012 | .519 | -1.5648E-011 | 8.0648E-012 |
| | | BFHDay2 | -4.79014E-012 | 5.82064E-012 | .417 | -1.6646E-011 | 7.0661E-012 |
| | | PFHDay2 | -6.52024E-012 | 5.82064E-012 | .271 | -1.8376E-011 | 5.3360E-012 |
| | | PMHDay2 | -9.01912E-012 | 5.82064E-012 | .131 | -2.0875E-011 | 2.8371E-012 |
| | PMHDay2 | BFHDay1 | 1.06697E-011 | 5.82064E-012 | .076 | -1.1865E-012 | 2.2526E-011 |

| | | | | | | | |
|---|---|---|---|---|---|---|---|
| | | PFHDay1 | 6.36640E-012 | 5.82064E-012 | .282 | -5.4899E-012 | 1.8223E-011 |
| | | BMHDay1 | 8.86244E-012 | 5.82064E-012 | .138 | -2.9938E-012 | 2.0719E-011 |
| | | PMHDay1 | 5.22768E-012 | 5.82064E-012 | .376 | -6.6286E-012 | 1.7084E-011 |
| | | BFHDay2 | 4.22898E-012 | 5.82064E-012 | .473 | -7.6273E-012 | 1.6085E-011 |
| | | PFHDay2 | 2.49888E-012 | 5.82064E-012 | .671 | -9.3574E-012 | 1.4355E-011 |
| | | BMHDay2 | 9.01912E-012 | 5.82064E-012 | .131 | -2.8371E-012 | 2.0875E-011 |
| SEA | BFHDay1 | PFHDay1 | 9.73844E-013 | 2.80215E-012 | .730 | -4.7339E-012 | 6.6816E-012 |
| | | BMHDay1 | -6.92144E-012* | 2.80215E-012 | .019 | -1.2629E-011 | -1.2136E-012 |
| | | PMHDay1 | -8.24772E-012* | 2.80215E-012 | .006 | -1.3956E-011 | -2.5399E-012 |
| | | BFHDay2 | 8.66406E-013 | 2.80215E-012 | .759 | -4.8414E-012 | 6.5742E-012 |
| | | PFHDay2 | -1.35594E-012 | 2.80215E-012 | .632 | -7.0637E-012 | 4.3519E-012 |
| | | BMHDay2 | -7.65270E-012* | 2.80215E-012 | .010 | -1.3360E-011 | -1.9449E-012 |
| | | PMHDay2 | -1.02071E-011* | 2.80215E-012 | <.001 | -1.5915E-011 | -4.4993E-012 |
| | PFHDay1 | BFHDay1 | -9.73844E-013 | 2.80215E-012 | .730 | -6.6816E-012 | 4.7339E-012 |
| | | BMHDay1 | -7.89528E-012* | 2.80215E-012 | .008 | -1.3603E-011 | -2.1875E-012 |
| | | PMHDay1 | -9.22156E-012* | 2.80215E-012 | .002 | -1.4929E-011 | -3.5138E-012 |
| | | BFHDay2 | -1.07438E-013 | 2.80215E-012 | .970 | -5.8152E-012 | 5.6004E-012 |
| | | PFHDay2 | -2.32978E-012 | 2.80215E-012 | .412 | -8.0376E-012 | 3.3780E-012 |
| | | BMHDay2 | -8.62654E-012* | 2.80215E-012 | .004 | -1.4334E-011 | -2.9188E-012 |

| | | | | | | |
|---|---|---|---|---|---|---|
| | PMHDay2 | -1.11809E-011* | 2.80215E-012 | <.001 | -1.6889E-011 | -5.4732E-012 |
| BMHDay1 | BFHDay1 | 6.92144E-012* | 2.80215E-012 | .019 | 1.2136E-012 | 1.2629E-011 |
| | PFHDay1 | 7.89528E-012* | 2.80215E-012 | .008 | 2.1875E-012 | 1.3603E-011 |
| | PMHDay1 | -1.32628E-012 | 2.80215E-012 | .639 | -7.0341E-012 | 4.3815E-012 |
| | BFHDay2 | 7.78785E-012* | 2.80215E-012 | .009 | 2.0801E-012 | 1.3496E-011 |
| | PFHDay2 | 5.56550E-012 | 2.80215E-012 | .056 | -1.4229E-013 | 1.1273E-011 |
| | BMHDay2 | -7.31260E-013 | 2.80215E-012 | .796 | -6.4391E-012 | 4.9765E-012 |
| | PMHDay2 | -3.28566E-012 | 2.80215E-012 | .250 | -8.9935E-012 | 2.4221E-012 |
| PMHDay1 | BFHDay1 | 8.24772E-012* | 2.80215E-012 | .006 | 2.5399E-012 | 1.3956E-011 |
| | PFHDay1 | 9.22156E-012* | 2.80215E-012 | .002 | 3.5138E-012 | 1.4929E-011 |
| | BMHDay1 | 1.32628E-012 | 2.80215E-012 | .639 | -4.3815E-012 | 7.0341E-012 |
| | BFHDay2 | 9.11413E-012* | 2.80215E-012 | .003 | 3.4063E-012 | 1.4822E-011 |
| | PFHDay2 | 6.89178E-012* | 2.80215E-012 | .020 | 1.1840E-012 | 1.2600E-011 |
| | BMHDay2 | 5.95020E-013 | 2.80215E-012 | .833 | -5.1128E-012 | 6.3028E-012 |
| | PMHDay2 | -1.95938E-012 | 2.80215E-012 | .489 | -7.6672E-012 | 3.7484E-012 |
| BFHDay2 | BFHDay1 | -8.66406E-013 | 2.80215E-012 | .759 | -6.5742E-012 | 4.8414E-012 |
| | PFHDay1 | 1.07438E-013 | 2.80215E-012 | .970 | -5.6004E-012 | 5.8152E-012 |
| | BMHDay1 | -7.78785E-012* | 2.80215E-012 | .009 | -1.3496E-011 | -2.0801E-012 |
| | PMHDay1 | -9.11413E-012* | 2.80215E-012 | .003 | -1.4822E-011 | -3.4063E-012 |

| | | | | | | | |
|---|---|---|---|---|---|---|---|
| | | PFHDay2 | -2.22235E-012 | 2.80215E-012 | .434 | -7.9301E-012 | 3.4854E-012 |
| | | BMHDay2 | -8.51911E-012* | 2.80215E-012 | .005 | -1.4227E-011 | -2.8113E-012 |
| | | PMHDay2 | -1.10735E-011* | 2.80215E-012 | <.001 | -1.6781E-011 | -5.3657E-012 |
| | PFHDay2 | BFHDay1 | 1.35594E-012 | 2.80215E-012 | .632 | -4.3519E-012 | 7.0637E-012 |
| | | PFHDay1 | 2.32978E-012 | 2.80215E-012 | .412 | -3.3780E-012 | 8.0376E-012 |
| | | BMHDay1 | -5.56550E-012 | 2.80215E-012 | .056 | -1.1273E-011 | 1.4229E-013 |
| | | PMHDay1 | -6.89178E-012* | 2.80215E-012 | .020 | -1.2600E-011 | -1.1840E-012 |
| | | BFHDay2 | 2.22235E-012 | 2.80215E-012 | .434 | -3.4854E-012 | 7.9301E-012 |
| | | BMHDay2 | -6.29676E-012* | 2.80215E-012 | .032 | -1.2005E-011 | -5.8897E-013 |
| | | PMHDay2 | -8.85116E-012* | 2.80215E-012 | .003 | -1.4559E-011 | -3.1434E-012 |
| | BMHDay2 | BFHDay1 | 7.65270E-012* | 2.80215E-012 | .010 | 1.9449E-012 | 1.3360E-011 |
| | | PFHDay1 | 8.62654E-012* | 2.80215E-012 | .004 | 2.9188E-012 | 1.4334E-011 |
| | | BMHDay1 | 7.31260E-013 | 2.80215E-012 | .796 | -4.9765E-012 | 6.4391E-012 |
| | | PMHDay1 | -5.95020E-013 | 2.80215E-012 | .833 | -6.3028E-012 | 5.1128E-012 |
| | | BFHDay2 | 8.51911E-012* | 2.80215E-012 | .005 | 2.8113E-012 | 1.4227E-011 |
| | | PFHDay2 | 6.29676E-012* | 2.80215E-012 | .032 | 5.8897E-013 | 1.2005E-011 |
| | | PMHDay2 | -2.55440E-012 | 2.80215E-012 | .369 | -8.2622E-012 | 3.1534E-012 |
| | PMHDay2 | BFHDay1 | 1.02071E-011* | 2.80215E-012 | <.001 | 4.4993E-012 | 1.5915E-011 |
| | | PFHDay1 | 1.11809E-011* | 2.80215E-012 | <.001 | 5.4732E-012 | 1.6889E-011 |

| | | | | | | | |
|---|---|---|---|---|---|---|---|
| | | BMHDay1 | 3.28566E-012 | 2.80215E-012 | .250 | -2.4221E-012 | 8.9935E-012 |
| | | PMHDay1 | 1.95938E-012 | 2.80215E-012 | .489 | -3.7484E-012 | 7.6672E-012 |
| | | BFHDay2 | 1.10735E-011* | 2.80215E-012 | <.001 | 5.3657E-012 | 1.6781E-011 |
| | | PFHDay2 | 8.85116E-012* | 2.80215E-012 | .003 | 3.1434E-012 | 1.4559E-011 |
| | | BMHDay2 | 2.55440E-012 | 2.80215E-012 | .369 | -3.1534E-012 | 8.2622E-012 |
| OEA | BFHDay1 | PFHDay1 | -1.30241E-011 | 4.91288E-011 | .793 | -1.1310E-010 | 8.7048E-011 |
| | | BMHDay1 | 6.46960E-013 | 4.91288E-011 | .990 | -9.9425E-011 | 1.0072E-010 |
| | | PMHDay1 | -1.66869E-011 | 4.91288E-011 | .736 | -1.1676E-010 | 8.3385E-011 |
| | | BFHDay2 | -2.83383E-011 | 4.91288E-011 | .568 | -1.2841E-010 | 7.1734E-011 |
| | | PFHDay2 | -5.44932E-011 | 4.91288E-011 | .276 | -1.5457E-010 | 4.5579E-011 |
| | | BMHDay2 | -2.84316E-011 | 4.91288E-011 | .567 | -1.2850E-010 | 7.1640E-011 |
| | | PMHDay2 | -8.24593E-011 | 4.91288E-011 | .103 | -1.8253E-010 | 1.7613E-011 |
| | PFHDay1 | BFHDay1 | 1.30241E-011 | 4.91288E-011 | .793 | -8.7048E-011 | 1.1310E-010 |
| | | BMHDay1 | 1.36711E-011 | 4.91288E-011 | .783 | -8.6401E-011 | 1.1374E-010 |
| | | PMHDay1 | -3.66281E-012 | 4.91288E-011 | .941 | -1.0373E-010 | 9.6409E-011 |
| | | BFHDay2 | -1.53142E-011 | 4.91288E-011 | .757 | -1.1539E-010 | 8.4758E-011 |
| | | PFHDay2 | -4.14691E-011 | 4.91288E-011 | .405 | -1.4154E-010 | 5.8603E-011 |
| | | BMHDay2 | -1.54075E-011 | 4.91288E-011 | .756 | -1.1548E-010 | 8.4664E-011 |
| | | PMHDay2 | -6.94352E-011 | 4.91288E-011 | .167 | -1.6951E-010 | 3.0637E-011 |

| | | | | | | |
|---|---|---|---|---|---|---|
| BMHDay1 | BFHDay1 | -6.46960E-013 | 4.91288E-011 | .990 | -1.0072E-010 | 9.9425E-011 |
| | PFHDay1 | -1.36711E-011 | 4.91288E-011 | .783 | -1.1374E-010 | 8.6401E-011 |
| | PMHDay1 | -1.73339E-011 | 4.91288E-011 | .727 | -1.1741E-010 | 8.2738E-011 |
| | BFHDay2 | -2.89853E-011 | 4.91288E-011 | .559 | -1.2906E-010 | 7.1087E-011 |
| | PFHDay2 | -5.51401E-011 | 4.91288E-011 | .270 | -1.5521E-010 | 4.4932E-011 |
| | BMHDay2 | -2.90786E-011 | 4.91288E-011 | .558 | -1.2915E-010 | 7.0993E-011 |
| | PMHDay2 | -8.31063E-011 | 4.91288E-011 | .100 | -1.8318E-010 | 1.6966E-011 |
| PMHDay1 | BFHDay1 | 1.66869E-011 | 4.91288E-011 | .736 | -8.3385E-011 | 1.1676E-010 |
| | PFHDay1 | 3.66281E-012 | 4.91288E-011 | .941 | -9.6409E-011 | 1.0373E-010 |
| | BMHDay1 | 1.73339E-011 | 4.91288E-011 | .727 | -8.2738E-011 | 1.1741E-010 |
| | BFHDay2 | -1.16514E-011 | 4.91288E-011 | .814 | -1.1172E-010 | 8.8421E-011 |
| | PFHDay2 | -3.78063E-011 | 4.91288E-011 | .447 | -1.3788E-010 | 6.2266E-011 |
| | BMHDay2 | -1.17447E-011 | 4.91288E-011 | .813 | -1.1182E-010 | 8.8327E-011 |
| | PMHDay2 | -6.57724E-011 | 4.91288E-011 | .190 | -1.6584E-010 | 3.4300E-011 |
| BFHDay2 | BFHDay1 | 2.83383E-011 | 4.91288E-011 | .568 | -7.1734E-011 | 1.2841E-010 |
| | PFHDay1 | 1.53142E-011 | 4.91288E-011 | .757 | -8.4758E-011 | 1.1539E-010 |
| | BMHDay1 | 2.89853E-011 | 4.91288E-011 | .559 | -7.1087E-011 | 1.2906E-010 |
| | PMHDay1 | 1.16514E-011 | 4.91288E-011 | .814 | -8.8421E-011 | 1.1172E-010 |
| | PFHDay2 | -2.61549E-011 | 4.91288E-011 | .598 | -1.2623E-010 | 7.3917E-011 |

| | | | | | | |
|---|---|---|---|---|---|---|
| | | BMHDay2 | -9.33460E-014 | 4.91288E-011 | .998 | -1.0017E-010 | 9.9979E-011 |
| | | PMHDay2 | -5.41210E-011 | 4.91288E-011 | .279 | -1.5419E-010 | 4.5951E-011 |
| | PFHDay2 | BFHDay1 | 5.44932E-011 | 4.91288E-011 | .276 | -4.5579E-011 | 1.5457E-010 |
| | | PFHDay1 | 4.14691E-011 | 4.91288E-011 | .405 | -5.8603E-011 | 1.4154E-010 |
| | | BMHDay1 | 5.51401E-011 | 4.91288E-011 | .270 | -4.4932E-011 | 1.5521E-010 |
| | | PMHDay1 | 3.78063E-011 | 4.91288E-011 | .447 | -6.2266E-011 | 1.3788E-010 |
| | | BFHDay2 | 2.61549E-011 | 4.91288E-011 | .598 | -7.3917E-011 | 1.2623E-010 |
| | | BMHDay2 | 2.60615E-011 | 4.91288E-011 | .599 | -7.4010E-011 | 1.2613E-010 |
| | | PMHDay2 | -2.79661E-011 | 4.91288E-011 | .573 | -1.2804E-010 | 7.2106E-011 |
| | BMHDay2 | BFHDay1 | 2.84316E-011 | 4.91288E-011 | .567 | -7.1640E-011 | 1.2850E-010 |
| | | PFHDay1 | 1.54075E-011 | 4.91288E-011 | .756 | -8.4664E-011 | 1.1548E-010 |
| | | BMHDay1 | 2.90786E-011 | 4.91288E-011 | .558 | -7.0993E-011 | 1.2915E-010 |
| | | PMHDay1 | 1.17447E-011 | 4.91288E-011 | .813 | -8.8327E-011 | 1.1182E-010 |
| | | BFHDay2 | 9.33460E-014 | 4.91288E-011 | .998 | -9.9979E-011 | 1.0017E-010 |
| | | PFHDay2 | -2.60615E-011 | 4.91288E-011 | .599 | -1.2613E-010 | 7.4010E-011 |
| | | PMHDay2 | -5.40277E-011 | 4.91288E-011 | .280 | -1.5410E-010 | 4.6044E-011 |
| | PMHDay2 | BFHDay1 | 8.24593E-011 | 4.91288E-011 | .103 | -1.7613E-011 | 1.8253E-010 |
| | | PFHDay1 | 6.94352E-011 | 4.91288E-011 | .167 | -3.0637E-011 | 1.6951E-010 |
| | | BMHDay1 | 8.31063E-011 | 4.91288E-011 | .100 | -1.6966E-011 | 1.8318E-010 |

| | | | | | | | |
|---|---|---|---|---|---|---|---|
| | | PMHDay1 | 6.57724E-011 | 4.91288E-011 | .190 | -3.4300E-011 | 1.6584E-010 |
| | | BFHDay2 | 5.41210E-011 | 4.91288E-011 | .279 | -4.5951E-011 | 1.5419E-010 |
| | | PFHDay2 | 2.79661E-011 | 4.91288E-011 | .573 | -7.2106E-011 | 1.2804E-010 |
| | | BMHDay2 | 5.40277E-011 | 4.91288E-011 | .280 | -4.6044E-011 | 1.5410E-010 |
| AEA | BFHDay1 | PFHDay1 | -3.82669E-014 | 7.65045E-014 | .620 | -1.9410E-013 | 1.1757E-013 |
| | | BMHDay1 | 2.55108E-014 | 7.65045E-014 | .741 | -1.3032E-013 | 1.8135E-013 |
| | | PMHDay1 | -3.21697E-014 | 7.65045E-014 | .677 | -1.8800E-013 | 1.2366E-013 |
| | | BFHDay2 | -1.24275E-013 | 7.65045E-014 | .114 | -2.8011E-013 | 3.1559E-014 |
| | | PFHDay2 | -1.24871E-013 | 7.65045E-014 | .112 | -2.8071E-013 | 3.0963E-014 |
| | | BMHDay2 | -7.14199E-014 | 7.65045E-014 | .358 | -2.2725E-013 | 8.4415E-014 |
| | | PMHDay2 | -1.34297E-013 | 7.65045E-014 | .089 | -2.9013E-013 | 2.1537E-014 |
| | PFHDay1 | BFHDay1 | 3.82669E-014 | 7.65045E-014 | .620 | -1.1757E-013 | 1.9410E-013 |
| | | BMHDay1 | 6.37777E-014 | 7.65045E-014 | .411 | -9.2057E-014 | 2.1961E-013 |
| | | PMHDay1 | 6.09716E-015 | 7.65045E-014 | .937 | -1.4974E-013 | 1.6193E-013 |
| | | BFHDay2 | -8.60084E-014 | 7.65045E-014 | .269 | -2.4184E-013 | 6.9826E-014 |
| | | PFHDay2 | -8.66046E-014 | 7.65045E-014 | .266 | -2.4244E-013 | 6.9230E-014 |
| | | BMHDay2 | -3.31531E-014 | 7.65045E-014 | .668 | -1.8899E-013 | 1.2268E-013 |
| | | PMHDay2 | -9.60302E-014 | 7.65045E-014 | .218 | -2.5186E-013 | 5.9804E-014 |
| | BMHDay1 | BFHDay1 | -2.55108E-014 | 7.65045E-014 | .741 | -1.8135E-013 | 1.3032E-013 |

| | | | | | | |
|---|---|---|---|---|---|---|
| | PFHDay1 | -6.37777E-014 | 7.65045E-014 | .411 | -2.1961E-013 | 9.2057E-014 |
| | PMHDay1 | -5.76805E-014 | 7.65045E-014 | .456 | -2.1352E-013 | 9.8154E-014 |
| | BFHDay2 | -1.49786E-013 | 7.65045E-014 | .059 | -3.0562E-013 | 6.0484E-015 |
| | PFHDay2 | -1.50382E-013 | 7.65045E-014 | .058 | -3.0622E-013 | 5.4522E-015 |
| | BMHDay2 | -9.69308E-014 | 7.65045E-014 | .214 | -2.5277E-013 | 5.8904E-014 |
| | PMHDay2 | -1.59808E-013* | 7.65045E-014 | .045 | -3.1564E-013 | -3.9734E-015 |
| PMHDay1 | BFHDay1 | 3.21697E-014 | 7.65045E-014 | .677 | -1.2366E-013 | 1.8800E-013 |
| | PFHDay1 | -6.09716E-015 | 7.65045E-014 | .937 | -1.6193E-013 | 1.4974E-013 |
| | BMHDay1 | 5.76805E-014 | 7.65045E-014 | .456 | -9.8154E-014 | 2.1352E-013 |
| | BFHDay2 | -9.21055E-014 | 7.65045E-014 | .237 | -2.4794E-013 | 6.3729E-014 |
| | PFHDay2 | -9.27018E-014 | 7.65045E-014 | .234 | -2.4854E-013 | 6.3133E-014 |
| | BMHDay2 | -3.92502E-014 | 7.65045E-014 | .611 | -1.9508E-013 | 1.1658E-013 |
| | PMHDay2 | -1.02127E-013 | 7.65045E-014 | .191 | -2.5796E-013 | 5.3707E-014 |
| BFHDay2 | BFHDay1 | 1.24275E-013 | 7.65045E-014 | .114 | -3.1559E-014 | 2.8011E-013 |
| | PFHDay1 | 8.60084E-014 | 7.65045E-014 | .269 | -6.9826E-014 | 2.4184E-013 |
| | BMHDay1 | 1.49786E-013 | 7.65045E-014 | .059 | -6.0484E-015 | 3.0562E-013 |
| | PMHDay1 | 9.21055E-014 | 7.65045E-014 | .237 | -6.3729E-014 | 2.4794E-013 |
| | PFHDay2 | -5.96240E-016 | 7.65045E-014 | .994 | -1.5643E-013 | 1.5524E-013 |
| | BMHDay2 | 5.28553E-014 | 7.65045E-014 | .495 | -1.0298E-013 | 2.0869E-013 |

| | | | | | | |
|---|---|---|---|---|---|---|
| | PMHDay2 | -1.00218E-014 | 7.65045E-014 | .897 | -1.6586E-013 | 1.4581E-013 |
| PFHDay2 | BFHDay1 | 1.24871E-013 | 7.65045E-014 | .112 | -3.0963E-014 | 2.8071E-013 |
| | PFHDay1 | 8.66046E-014 | 7.65045E-014 | .266 | -6.9230E-014 | 2.4244E-013 |
| | BMHDay1 | 1.50382E-013 | 7.65045E-014 | .058 | -5.4522E-015 | 3.0622E-013 |
| | PMHDay1 | 9.27018E-014 | 7.65045E-014 | .234 | -6.3133E-014 | 2.4854E-013 |
| | BFHDay2 | 5.96240E-016 | 7.65045E-014 | .994 | -1.5524E-013 | 1.5643E-013 |
| | BMHDay2 | 5.34516E-014 | 7.65045E-014 | .490 | -1.0238E-013 | 2.0929E-013 |
| | PMHDay2 | -9.42560E-015 | 7.65045E-014 | .903 | -1.6526E-013 | 1.4641E-013 |
| BMHDay2 | BFHDay1 | 7.14199E-014 | 7.65045E-014 | .358 | -8.4415E-014 | 2.2725E-013 |
| | PFHDay1 | 3.31531E-014 | 7.65045E-014 | .668 | -1.2268E-013 | 1.8899E-013 |
| | BMHDay1 | 9.69308E-014 | 7.65045E-014 | .214 | -5.8904E-014 | 2.5277E-013 |
| | PMHDay1 | 3.92502E-014 | 7.65045E-014 | .611 | -1.1658E-013 | 1.9508E-013 |
| | BFHDay2 | -5.28553E-014 | 7.65045E-014 | .495 | -2.0869E-013 | 1.0298E-013 |
| | PFHDay2 | -5.34516E-014 | 7.65045E-014 | .490 | -2.0929E-013 | 1.0238E-013 |
| | PMHDay2 | -6.28772E-014 | 7.65045E-014 | .417 | -2.1871E-013 | 9.2957E-014 |
| PMHDay2 | BFHDay1 | 1.34297E-013 | 7.65045E-014 | .089 | -2.1537E-014 | 2.9013E-013 |
| | PFHDay1 | 9.60302E-014 | 7.65045E-014 | .218 | -5.9804E-014 | 2.5186E-013 |
| | BMHDay1 | 1.59808E-013* | 7.65045E-014 | .045 | 3.9734E-015 | 3.1564E-013 |
| | PMHDay1 | 1.02127E-013 | 7.65045E-014 | .191 | -5.3707E-014 | 2.5796E-013 |

| | | | | | | | |
|---|---|---|---|---|---|---|---|
| | | BFHDay2 | 1.00218E-014 | 7.65045E-014 | .897 | -1.4581E-013 | 1.6586E-013 |
| | | PFHDay2 | 9.42560E-015 | 7.65045E-014 | .903 | -1.4641E-013 | 1.6526E-013 |
| | | BMHDay2 | 6.28772E-014 | 7.65045E-014 | .417 | -9.2957E-014 | 2.1871E-013 |
| LEA | BFHDay1 | PFHDay1 | -3.90070E-014 | 2.67794E-013 | .885 | -5.8449E-013 | 5.0647E-013 |
| | | BMHDay1 | 3.57293E-013 | 2.67794E-013 | .192 | -1.8819E-013 | 9.0277E-013 |
| | | PMHDay1 | -2.09327E-013 | 2.67794E-013 | .440 | -7.5481E-013 | 3.3615E-013 |
| | | BFHDay2 | -1.07466E-013 | 2.67794E-013 | .691 | -6.5295E-013 | 4.3801E-013 |
| | | PFHDay2 | -5.47717E-013* | 2.67794E-013 | .049 | -1.0932E-012 | -2.2379E-015 |
| | | BMHDay2 | -1.71044E-013 | 2.67794E-013 | .528 | -7.1652E-013 | 3.7444E-013 |
| | | PMHDay2 | -4.87783E-013 | 2.67794E-013 | .078 | -1.0333E-012 | 5.7696E-014 |
| | PFHDay1 | BFHDay1 | 3.90070E-014 | 2.67794E-013 | .885 | -5.0647E-013 | 5.8449E-013 |
| | | BMHDay1 | 3.96300E-013 | 2.67794E-013 | .149 | -1.4918E-013 | 9.4178E-013 |
| | | PMHDay1 | -1.70320E-013 | 2.67794E-013 | .529 | -7.1580E-013 | 3.7516E-013 |
| | | BFHDay2 | -6.84588E-014 | 2.67794E-013 | .800 | -6.1394E-013 | 4.7702E-013 |
| | | PFHDay2 | -5.08710E-013 | 2.67794E-013 | .067 | -1.0542E-012 | 3.6769E-014 |
| | | BMHDay2 | -1.32037E-013 | 2.67794E-013 | .625 | -6.7752E-013 | 4.1344E-013 |
| | | PMHDay2 | -4.48776E-013 | 2.67794E-013 | .104 | -9.9426E-013 | 9.6703E-014 |
| | BMHDay1 | BFHDay1 | -3.57293E-013 | 2.67794E-013 | .192 | -9.0277E-013 | 1.8819E-013 |
| | | PFHDay1 | -3.96300E-013 | 2.67794E-013 | .149 | -9.4178E-013 | 1.4918E-013 |

| | | | | | | |
|---|---|---|---|---|---|---|
| | PMHDay1 | -5.66619E-013* | 2.67794E-013 | .042 | -1.1121E-012 | -2.1140E-014 |
| | BFHDay2 | -4.64758E-013 | 2.67794E-013 | .092 | -1.0102E-012 | 8.0721E-014 |
| | PFHDay2 | -9.05010E-013* | 2.67794E-013 | .002 | -1.4505E-012 | -3.5953E-013 |
| | BMHDay2 | -5.28337E-013 | 2.67794E-013 | .057 | -1.0738E-012 | 1.7143E-014 |
| | PMHDay2 | -8.45076E-013* | 2.67794E-013 | .003 | -1.3906E-012 | -2.9960E-013 |
| PMHDay1 | BFHDay1 | 2.09327E-013 | 2.67794E-013 | .440 | -3.3615E-013 | 7.5481E-013 |
| | PFHDay1 | 1.70320E-013 | 2.67794E-013 | .529 | -3.7516E-013 | 7.1580E-013 |
| | BMHDay1 | 5.66619E-013* | 2.67794E-013 | .042 | 2.1140E-014 | 1.1121E-012 |
| | BFHDay2 | 1.01861E-013 | 2.67794E-013 | .706 | -4.4362E-013 | 6.4734E-013 |
| | PFHDay2 | -3.38391E-013 | 2.67794E-013 | .215 | -8.8387E-013 | 2.0709E-013 |
| | BMHDay2 | 3.82826E-014 | 2.67794E-013 | .887 | -5.0720E-013 | 5.8376E-013 |
| | PMHDay2 | -2.78457E-013 | 2.67794E-013 | .306 | -8.2394E-013 | 2.6702E-013 |
| BFHDay2 | BFHDay1 | 1.07466E-013 | 2.67794E-013 | .691 | -4.3801E-013 | 6.5295E-013 |
| | PFHDay1 | 6.84588E-014 | 2.67794E-013 | .800 | -4.7702E-013 | 6.1394E-013 |
| | BMHDay1 | 4.64758E-013 | 2.67794E-013 | .092 | -8.0721E-014 | 1.0102E-012 |
| | PMHDay1 | -1.01861E-013 | 2.67794E-013 | .706 | -6.4734E-013 | 4.4362E-013 |
| | PFHDay2 | -4.40252E-013 | 2.67794E-013 | .110 | -9.8573E-013 | 1.0523E-013 |
| | BMHDay2 | -6.35784E-014 | 2.67794E-013 | .814 | -6.0906E-013 | 4.8190E-013 |
| | PMHDay2 | -3.80318E-013 | 2.67794E-013 | .165 | -9.2580E-013 | 1.6516E-013 |

| | | | | | | |
|---|---|---|---|---|---|---|
| PFHDay2 | BFHDay1 | 5.47717E-013* | 2.67794E-013 | .049 | 2.2379E-015 | 1.0932E-012 |
| | PFHDay1 | 5.08710E-013 | 2.67794E-013 | .067 | -3.6769E-014 | 1.0542E-012 |
| | BMHDay1 | 9.05010E-013* | 2.67794E-013 | .002 | 3.5953E-013 | 1.4505E-012 |
| | PMHDay1 | 3.38391E-013 | 2.67794E-013 | .215 | -2.0709E-013 | 8.8387E-013 |
| | BFHDay2 | 4.40252E-013 | 2.67794E-013 | .110 | -1.0523E-013 | 9.8573E-013 |
| | BMHDay2 | 3.76673E-013 | 2.67794E-013 | .169 | -1.6881E-013 | 9.2215E-013 |
| | PMHDay2 | 5.99340E-014 | 2.67794E-013 | .824 | -4.8555E-013 | 6.0541E-013 |
| BMHDay2 | BFHDay1 | 1.71044E-013 | 2.67794E-013 | .528 | -3.7444E-013 | 7.1652E-013 |
| | PFHDay1 | 1.32037E-013 | 2.67794E-013 | .625 | -4.1344E-013 | 6.7752E-013 |
| | BMHDay1 | 5.28337E-013 | 2.67794E-013 | .057 | -1.7143E-014 | 1.0738E-012 |
| | PMHDay1 | -3.82826E-014 | 2.67794E-013 | .887 | -5.8376E-013 | 5.0720E-013 |
| | BFHDay2 | 6.35784E-014 | 2.67794E-013 | .814 | -4.8190E-013 | 6.0906E-013 |
| | PFHDay2 | -3.76673E-013 | 2.67794E-013 | .169 | -9.2215E-013 | 1.6881E-013 |
| | PMHDay2 | -3.16739E-013 | 2.67794E-013 | .246 | -8.6222E-013 | 2.2874E-013 |
| PMHDay2 | BFHDay1 | 4.87783E-013 | 2.67794E-013 | .078 | -5.7696E-014 | 1.0333E-012 |
| | PFHDay1 | 4.48776E-013 | 2.67794E-013 | .104 | -9.6703E-014 | 9.9426E-013 |
| | BMHDay1 | 8.45076E-013* | 2.67794E-013 | .003 | 2.9960E-013 | 1.3906E-012 |
| | PMHDay1 | 2.78457E-013 | 2.67794E-013 | .306 | -2.6702E-013 | 8.2394E-013 |
| | BFHDay2 | 3.80318E-013 | 2.67794E-013 | .165 | -1.6516E-013 | 9.2580E-013 |

| | | | | | | | |
|---|---|---|---|---|---|---|---|
| | | PFHDay2 | -5.99340E-014 | 2.67794E-013 | .824 | -6.0541E-013 | 4.8555E-013 |
| | | BMHDay2 | 3.16739E-013 | 2.67794E-013 | .246 | -2.2874E-013 | 8.6222E-013 |
| DEA | BFHDay1 | PFHDay1 | -3.33790E-012 | 2.13056E-012 | .127 | -7.6777E-012 | 1.0019E-012 |
| | | BMHDay1 | -8.68178E-013 | 2.13056E-012 | .686 | -5.2080E-012 | 3.4716E-012 |
| | | PMHDay1 | -2.41066E-013 | 2.13056E-012 | .911 | -4.5809E-012 | 4.0987E-012 |
| | | BFHDay2 | -9.30888E-013 | 2.13056E-012 | .665 | -5.2707E-012 | 3.4089E-012 |
| | | PFHDay2 | -5.39248E-012* | 2.13056E-012 | .016 | -9.7323E-012 | -1.0527E-012 |
| | | BMHDay2 | -1.65968E-012 | 2.13056E-012 | .442 | -5.9995E-012 | 2.6801E-012 |
| | | PMHDay2 | -1.89200E-012 | 2.13056E-012 | .381 | -6.2318E-012 | 2.4478E-012 |
| | PFHDay1 | BFHDay1 | 3.33790E-012 | 2.13056E-012 | .127 | -1.0019E-012 | 7.6777E-012 |
| | | BMHDay1 | 2.46972E-012 | 2.13056E-012 | .255 | -1.8701E-012 | 6.8095E-012 |
| | | PMHDay1 | 3.09684E-012 | 2.13056E-012 | .156 | -1.2430E-012 | 7.4366E-012 |
| | | BFHDay2 | 2.40701E-012 | 2.13056E-012 | .267 | -1.9328E-012 | 6.7468E-012 |
| | | PFHDay2 | -2.05458E-012 | 2.13056E-012 | .342 | -6.3944E-012 | 2.2852E-012 |
| | | BMHDay2 | 1.67823E-012 | 2.13056E-012 | .437 | -2.6616E-012 | 6.0180E-012 |
| | | PMHDay2 | 1.44590E-012 | 2.13056E-012 | .502 | -2.8939E-012 | 5.7857E-012 |
| | BMHDay1 | BFHDay1 | 8.68178E-013 | 2.13056E-012 | .686 | -3.4716E-012 | 5.2080E-012 |
| | | PFHDay1 | -2.46972E-012 | 2.13056E-012 | .255 | -6.8095E-012 | 1.8701E-012 |
| | | PMHDay1 | 6.27112E-013 | 2.13056E-012 | .770 | -3.7127E-012 | 4.9669E-012 |

| | | | | | | | |
|---|---|---|---|---|---|---|---|
| | | BFHDay2 | -6.27100E-014 | 2.13056E-012 | .977 | -4.4025E-012 | 4.2771E-012 |
| | | PFHDay2 | -4.52430E-012* | 2.13056E-012 | .042 | -8.8641E-012 | -1.8450E-013 |
| | | BMHDay2 | -7.91498E-013 | 2.13056E-012 | .713 | -5.1313E-012 | 3.5483E-012 |
| | | PMHDay2 | -1.02382E-012 | 2.13056E-012 | .634 | -5.3636E-012 | 3.3160E-012 |
| | PMHDay1 | BFHDay1 | 2.41066E-013 | 2.13056E-012 | .911 | -4.0987E-012 | 4.5809E-012 |
| | | PFHDay1 | -3.09684E-012 | 2.13056E-012 | .156 | -7.4366E-012 | 1.2430E-012 |
| | | BMHDay1 | -6.27112E-013 | 2.13056E-012 | .770 | -4.9669E-012 | 3.7127E-012 |
| | | BFHDay2 | -6.89822E-013 | 2.13056E-012 | .748 | -5.0296E-012 | 3.6500E-012 |
| | | PFHDay2 | -5.15141E-012* | 2.13056E-012 | .021 | -9.4912E-012 | -8.1161E-013 |
| | | BMHDay2 | -1.41861E-012 | 2.13056E-012 | .510 | -5.7584E-012 | 2.9212E-012 |
| | | PMHDay2 | -1.65093E-012 | 2.13056E-012 | .444 | -5.9907E-012 | 2.6889E-012 |
| | BFHDay2 | BFHDay1 | 9.30888E-013 | 2.13056E-012 | .665 | -3.4089E-012 | 5.2707E-012 |
| | | PFHDay1 | -2.40701E-012 | 2.13056E-012 | .267 | -6.7468E-012 | 1.9328E-012 |
| | | BMHDay1 | 6.27100E-014 | 2.13056E-012 | .977 | -4.2771E-012 | 4.4025E-012 |
| | | PMHDay1 | 6.89822E-013 | 2.13056E-012 | .748 | -3.6500E-012 | 5.0296E-012 |
| | | PFHDay2 | -4.46159E-012* | 2.13056E-012 | .044 | -8.8014E-012 | -1.2179E-013 |
| | | BMHDay2 | -7.28788E-013 | 2.13056E-012 | .735 | -5.0686E-012 | 3.6110E-012 |
| | | PMHDay2 | -9.61110E-013 | 2.13056E-012 | .655 | -5.3009E-012 | 3.3787E-012 |
| | PFHDay2 | BFHDay1 | 5.39248E-012* | 2.13056E-012 | .016 | 1.0527E-012 | 9.7323E-012 |

| | | | | | | |
|---|---|---|---|---|---|---|
| | PFHDay1 | 2.05458E-012 | 2.13056E-012 | .342 | -2.2852E-012 | 6.3944E-012 |
| | BMHDay1 | 4.52430E-012* | 2.13056E-012 | .042 | 1.8450E-013 | 8.8641E-012 |
| | PMHDay1 | 5.15141E-012* | 2.13056E-012 | .021 | 8.1161E-013 | 9.4912E-012 |
| | BFHDay2 | 4.46159E-012* | 2.13056E-012 | .044 | 1.2179E-013 | 8.8014E-012 |
| | BMHDay2 | 3.73280E-012 | 2.13056E-012 | .089 | -6.0700E-013 | 8.0726E-012 |
| | PMHDay2 | 3.50048E-012 | 2.13056E-012 | .110 | -8.3932E-013 | 7.8403E-012 |
| BMHDay2 | BFHDay1 | 1.65968E-012 | 2.13056E-012 | .442 | -2.6801E-012 | 5.9995E-012 |
| | PFHDay1 | -1.67823E-012 | 2.13056E-012 | .437 | -6.0180E-012 | 2.6616E-012 |
| | BMHDay1 | 7.91498E-013 | 2.13056E-012 | .713 | -3.5483E-012 | 5.1313E-012 |
| | PMHDay1 | 1.41861E-012 | 2.13056E-012 | .510 | -2.9212E-012 | 5.7584E-012 |
| | BFHDay2 | 7.28788E-013 | 2.13056E-012 | .735 | -3.6110E-012 | 5.0686E-012 |
| | PFHDay2 | -3.73280E-012 | 2.13056E-012 | .089 | -8.0726E-012 | 6.0700E-013 |
| | PMHDay2 | -2.32322E-013 | 2.13056E-012 | .914 | -4.5721E-012 | 4.1075E-012 |
| PMHDay2 | BFHDay1 | 1.89200E-012 | 2.13056E-012 | .381 | -2.4478E-012 | 6.2318E-012 |
| | PFHDay1 | -1.44590E-012 | 2.13056E-012 | .502 | -5.7857E-012 | 2.8939E-012 |
| | BMHDay1 | 1.02382E-012 | 2.13056E-012 | .634 | -3.3160E-012 | 5.3636E-012 |
| | PMHDay1 | 1.65093E-012 | 2.13056E-012 | .444 | -2.6889E-012 | 5.9907E-012 |
| | BFHDay2 | 9.61110E-013 | 2.13056E-012 | .655 | -3.3787E-012 | 5.3009E-012 |
| | PFHDay2 | -3.50048E-012 | 2.13056E-012 | .110 | -7.8403E-012 | 8.3932E-013 |

| | | | | | | | |
|---|---|---|---|---|---|---|---|
| | | BMHDay2 | 2.32322E-013 | 2.13056E-012 | .914 | -4.1075E-012 | 4.5721E-012 |
| StrTaurine | BFHDay1 | PFHDay1 | -6.78448E-014 | 2.30174E-013 | .770 | -5.4012E-013 | 4.0443E-013 |
| | | BMHDay1 | 1.22228E-013 | 2.30174E-013 | .600 | -3.5005E-013 | 5.9451E-013 |
| | | PMHDay1 | -4.35817E-013 | 2.30174E-013 | .069 | -9.0810E-013 | 3.6461E-014 |
| | | BFHDay2 | -2.63323E-015 | 2.97153E-013 | .993 | -6.1234E-013 | 6.0707E-013 |
| | | PFHDay2 | -1.53967E-013 | 2.30174E-013 | .509 | -6.2625E-013 | 3.1831E-013 |
| | | BMHDay2 | -5.23896E-013* | 2.42625E-013 | .040 | -1.0217E-012 | -2.6072E-014 |
| | | PMHDay2 | -2.10314E-013 | 2.30174E-013 | .369 | -6.8259E-013 | 2.6196E-013 |
| | PFHDay1 | BFHDay1 | 6.78448E-014 | 2.30174E-013 | .770 | -4.0443E-013 | 5.4012E-013 |
| | | BMHDay1 | 1.90073E-013 | 2.17010E-013 | .389 | -2.5520E-013 | 6.3534E-013 |
| | | PMHDay1 | -3.67972E-013 | 2.17010E-013 | .101 | -8.1324E-013 | 7.7295E-014 |
| | | BFHDay2 | 6.52115E-014 | 2.87077E-013 | .822 | -5.2382E-013 | 6.5425E-013 |
| | | PFHDay2 | -8.61227E-014 | 2.17010E-013 | .695 | -5.3139E-013 | 3.5915E-013 |
| | | BMHDay2 | -4.56052E-013 | 2.30174E-013 | .058 | -9.2833E-013 | 1.6226E-014 |
| | | PMHDay2 | -1.42470E-013 | 2.17010E-013 | .517 | -5.8774E-013 | 3.0280E-013 |
| | BMHDay1 | BFHDay1 | -1.22228E-013 | 2.30174E-013 | .600 | -5.9451E-013 | 3.5005E-013 |
| | | PFHDay1 | -1.90073E-013 | 2.17010E-013 | .389 | -6.3534E-013 | 2.5520E-013 |
| | | PMHDay1 | -5.58045E-013* | 2.17010E-013 | .016 | -1.0033E-012 | -1.1278E-013 |
| | | BFHDay2 | -1.24861E-013 | 2.87077E-013 | .667 | -7.1390E-013 | 4.6417E-013 |

| | | | | | | |
|---|---|---|---|---|---|---|
| | PFHDay2 | -2.76195E-013 | 2.17010E-013 | .214 | -7.2146E-013 | 1.6907E-013 |
| | BMHDay2 | -6.46124E-013* | 2.30174E-013 | .009 | -1.1184E-012 | -1.7385E-013 |
| | PMHDay2 | -3.32542E-013 | 2.17010E-013 | .137 | -7.7781E-013 | 1.1273E-013 |
| PMHDay1 | BFHDay1 | 4.35817E-013 | 2.30174E-013 | .069 | -3.6461E-014 | 9.0810E-013 |
| | PFHDay1 | 3.67972E-013 | 2.17010E-013 | .101 | -7.7295E-014 | 8.1324E-013 |
| | BMHDay1 | 5.58045E-013* | 2.17010E-013 | .016 | 1.1278E-013 | 1.0033E-012 |
| | BFHDay2 | 4.33184E-013 | 2.87077E-013 | .143 | -1.5585E-013 | 1.0222E-012 |
| | PFHDay2 | 2.81850E-013 | 2.17010E-013 | .205 | -1.6342E-013 | 7.2712E-013 |
| | BMHDay2 | -8.80792E-014 | 2.30174E-013 | .705 | -5.6036E-013 | 3.8420E-013 |
| | PMHDay2 | 2.25503E-013 | 2.17010E-013 | .308 | -2.1976E-013 | 6.7077E-013 |
| BFHDay2 | BFHDay1 | 2.63323E-015 | 2.97153E-013 | .993 | -6.0707E-013 | 6.1234E-013 |
| | PFHDay1 | -6.52115E-014 | 2.87077E-013 | .822 | -6.5425E-013 | 5.2382E-013 |
| | BMHDay1 | 1.24861E-013 | 2.87077E-013 | .667 | -4.6417E-013 | 7.1390E-013 |
| | PMHDay1 | -4.33184E-013 | 2.87077E-013 | .143 | -1.0222E-012 | 1.5585E-013 |
| | PFHDay2 | -1.51334E-013 | 2.87077E-013 | .602 | -7.4037E-013 | 4.3770E-013 |
| | BMHDay2 | -5.21263E-013 | 2.97153E-013 | .091 | -1.1310E-012 | 8.8445E-014 |
| | PMHDay2 | -2.07681E-013 | 2.87077E-013 | .476 | -7.9672E-013 | 3.8135E-013 |
| PFHDay2 | BFHDay1 | 1.53967E-013 | 2.30174E-013 | .509 | -3.1831E-013 | 6.2625E-013 |
| | PFHDay1 | 8.61227E-014 | 2.17010E-013 | .695 | -3.5915E-013 | 5.3139E-013 |

| | | | | | | |
|---|---|---|---|---|---|---|
| | | BMHDay1 | 2.76195E-013 | 2.17010E-013 | .214 | -1.6907E-013 | 7.2146E-013 |
| | | PMHDay1 | -2.81850E-013 | 2.17010E-013 | .205 | -7.2712E-013 | 1.6342E-013 |
| | | BFHDay2 | 1.51334E-013 | 2.87077E-013 | .602 | -4.3770E-013 | 7.4037E-013 |
| | | BMHDay2 | -3.69929E-013 | 2.30174E-013 | .120 | -8.4221E-013 | 1.0235E-013 |
| | | PMHDay2 | -5.63469E-014 | 2.17010E-013 | .797 | -5.0161E-013 | 3.8892E-013 |
| | BMHDay2 | BFHDay1 | 5.23896E-013* | 2.42625E-013 | .040 | 2.6072E-014 | 1.0217E-012 |
| | | PFHDay1 | 4.56052E-013 | 2.30174E-013 | .058 | -1.6226E-014 | 9.2833E-013 |
| | | BMHDay1 | 6.46124E-013* | 2.30174E-013 | .009 | 1.7385E-013 | 1.1184E-012 |
| | | PMHDay1 | 8.80792E-014 | 2.30174E-013 | .705 | -3.8420E-013 | 5.6036E-013 |
| | | BFHDay2 | 5.21263E-013 | 2.97153E-013 | .091 | -8.8445E-014 | 1.1310E-012 |
| | | PFHDay2 | 3.69929E-013 | 2.30174E-013 | .120 | -1.0235E-013 | 8.4221E-013 |
| | | PMHDay2 | 3.13582E-013 | 2.30174E-013 | .184 | -1.5870E-013 | 7.8586E-013 |
| | PMHDay2 | BFHDay1 | 2.10314E-013 | 2.30174E-013 | .369 | -2.6196E-013 | 6.8259E-013 |
| | | PFHDay1 | 1.42470E-013 | 2.17010E-013 | .517 | -3.0280E-013 | 5.8774E-013 |
| | | BMHDay1 | 3.32542E-013 | 2.17010E-013 | .137 | -1.1273E-013 | 7.7781E-013 |
| | | PMHDay1 | -2.25503E-013 | 2.17010E-013 | .308 | -6.7077E-013 | 2.1976E-013 |
| | | BFHDay2 | 2.07681E-013 | 2.87077E-013 | .476 | -3.8135E-013 | 7.9672E-013 |
| | | PFHDay2 | 5.63469E-014 | 2.17010E-013 | .797 | -3.8892E-013 | 5.0161E-013 |
| | | BMHDay2 | -3.13582E-013 | 2.30174E-013 | .184 | -7.8586E-013 | 1.5870E-013 |

| | | | | | | | |
|---|---|---|---|---|---|---|---|
| OlTaurine | BFHDay1 | PFHDay1 | -6.54400E-014 | 2.52474E-013 | .797 | -5.8106E-013 | 4.5018E-013 |
| | | BMHDay1 | 1.64370E-013 | 2.52474E-013 | .520 | -3.5125E-013 | 6.7999E-013 |
| | | PMHDay1 | -2.18281E-013 | 2.52474E-013 | .394 | -7.3390E-013 | 2.9734E-013 |
| | | BFHDay2 | -1.51263E-013 | 2.66131E-013 | .574 | -6.9477E-013 | 3.9225E-013 |
| | | PFHDay2 | -1.69237E-013 | 2.52474E-013 | .508 | -6.8486E-013 | 3.4638E-013 |
| | | BMHDay2 | -9.89693E-014 | 2.52474E-013 | .698 | -6.1459E-013 | 4.1665E-013 |
| | | PMHDay2 | -6.01435E-013* | 2.52474E-013 | .024 | -1.1171E-012 | -8.5815E-014 |
| | PFHDay1 | BFHDay1 | 6.54400E-014 | 2.52474E-013 | .797 | -4.5018E-013 | 5.8106E-013 |
| | | BMHDay1 | 2.29810E-013 | 2.38035E-013 | .342 | -2.5632E-013 | 7.1594E-013 |
| | | PMHDay1 | -1.52841E-013 | 2.38035E-013 | .526 | -6.3897E-013 | 3.3329E-013 |
| | | BFHDay2 | -8.58233E-014 | 2.52474E-013 | .736 | -6.0144E-013 | 4.2980E-013 |
| | | PFHDay2 | -1.03797E-013 | 2.38035E-013 | .666 | -5.8993E-013 | 3.8233E-013 |
| | | BMHDay2 | -3.35293E-014 | 2.38035E-013 | .889 | -5.1966E-013 | 4.5260E-013 |
| | | PMHDay2 | -5.35995E-013* | 2.38035E-013 | .032 | -1.0221E-012 | -4.9864E-014 |
| | BMHDay1 | BFHDay1 | -1.64370E-013 | 2.52474E-013 | .520 | -6.7999E-013 | 3.5125E-013 |
| | | PFHDay1 | -2.29810E-013 | 2.38035E-013 | .342 | -7.1594E-013 | 2.5632E-013 |
| | | PMHDay1 | -3.82650E-013 | 2.38035E-013 | .118 | -8.6878E-013 | 1.0348E-013 |
| | | BFHDay2 | -3.15633E-013 | 2.52474E-013 | .221 | -8.3125E-013 | 1.9999E-013 |
| | | PFHDay2 | -3.33607E-013 | 2.38035E-013 | .171 | -8.1974E-013 | 1.5252E-013 |

| | | | | | | |
|---|---|---|---|---|---|---|
| | BMHDay2 | -2.63339E-013 | 2.38035E-013 | .277 | -7.4947E-013 | 2.2279E-013 |
| | PMHDay2 | -7.65805E-013* | 2.38035E-013 | .003 | -1.2519E-012 | -2.7967E-013 |
| PMHDay1 | BFHDay1 | 2.18281E-013 | 2.52474E-013 | .394 | -2.9734E-013 | 7.3390E-013 |
| | PFHDay1 | 1.52841E-013 | 2.38035E-013 | .526 | -3.3329E-013 | 6.3897E-013 |
| | BMHDay1 | 3.82650E-013 | 2.38035E-013 | .118 | -1.0348E-013 | 8.6878E-013 |
| | BFHDay2 | 6.70172E-014 | 2.52474E-013 | .792 | -4.4860E-013 | 5.8264E-013 |
| | PFHDay2 | 4.90436E-014 | 2.38035E-013 | .838 | -4.3709E-013 | 5.3517E-013 |
| | BMHDay2 | 1.19311E-013 | 2.38035E-013 | .620 | -3.6682E-013 | 6.0544E-013 |
| | PMHDay2 | -3.83155E-013 | 2.38035E-013 | .118 | -8.6929E-013 | 1.0298E-013 |
| BFHDay2 | BFHDay1 | 1.51263E-013 | 2.66131E-013 | .574 | -3.9225E-013 | 6.9477E-013 |
| | PFHDay1 | 8.58233E-014 | 2.52474E-013 | .736 | -4.2980E-013 | 6.0144E-013 |
| | BMHDay1 | 3.15633E-013 | 2.52474E-013 | .221 | -1.9999E-013 | 8.3125E-013 |
| | PMHDay1 | -6.70172E-014 | 2.52474E-013 | .792 | -5.8264E-013 | 4.4860E-013 |
| | PFHDay2 | -1.79736E-014 | 2.52474E-013 | .944 | -5.3359E-013 | 4.9765E-013 |
| | BMHDay2 | 5.22940E-014 | 2.52474E-013 | .837 | -4.6333E-013 | 5.6791E-013 |
| | PMHDay2 | -4.50172E-013 | 2.52474E-013 | .085 | -9.6579E-013 | 6.5448E-014 |
| PFHDay2 | BFHDay1 | 1.69237E-013 | 2.52474E-013 | .508 | -3.4638E-013 | 6.8486E-013 |
| | PFHDay1 | 1.03797E-013 | 2.38035E-013 | .666 | -3.8233E-013 | 5.8993E-013 |
| | BMHDay1 | 3.33607E-013 | 2.38035E-013 | .171 | -1.5252E-013 | 8.1974E-013 |

| | | | | | | | |
|---|---|---|---|---|---|---|---|
| | | PMHDay1 | -4.90436E-014 | 2.38035E-013 | .838 | -5.3517E-013 | 4.3709E-013 |
| | | BFHDay2 | 1.79736E-014 | 2.52474E-013 | .944 | -4.9765E-013 | 5.3359E-013 |
| | | BMHDay2 | 7.02676E-014 | 2.38035E-013 | .770 | -4.1586E-013 | 5.5640E-013 |
| | | PMHDay2 | -4.32198E-013 | 2.38035E-013 | .079 | -9.1833E-013 | 5.3933E-014 |
| | BMHDay2 | BFHDay1 | 9.89693E-014 | 2.52474E-013 | .698 | -4.1665E-013 | 6.1459E-013 |
| | | PFHDay1 | 3.35293E-014 | 2.38035E-013 | .889 | -4.5260E-013 | 5.1966E-013 |
| | | BMHDay1 | 2.63339E-013 | 2.38035E-013 | .277 | -2.2279E-013 | 7.4947E-013 |
| | | PMHDay1 | -1.19311E-013 | 2.38035E-013 | .620 | -6.0544E-013 | 3.6682E-013 |
| | | BFHDay2 | -5.22940E-014 | 2.52474E-013 | .837 | -5.6791E-013 | 4.6333E-013 |
| | | PFHDay2 | -7.02676E-014 | 2.38035E-013 | .770 | -5.5640E-013 | 4.1586E-013 |
| | | PMHDay2 | -5.02466E-013* | 2.38035E-013 | .043 | -9.8860E-013 | -1.6335E-014 |
| | PMHDay2 | BFHDay1 | 6.01435E-013* | 2.52474E-013 | .024 | 8.5815E-014 | 1.1171E-012 |
| | | PFHDay1 | 5.35995E-013* | 2.38035E-013 | .032 | 4.9864E-014 | 1.0221E-012 |
| | | BMHDay1 | 7.65805E-013* | 2.38035E-013 | .003 | 2.7967E-013 | 1.2519E-012 |
| | | PMHDay1 | 3.83155E-013 | 2.38035E-013 | .118 | -1.0298E-013 | 8.6929E-013 |
| | | BFHDay2 | 4.50172E-013 | 2.52474E-013 | .085 | -6.5448E-014 | 9.6579E-013 |
| | | PFHDay2 | 4.32198E-013 | 2.38035E-013 | .079 | -5.3933E-014 | 9.1833E-013 |
| | | BMHDay2 | 5.02466E-013* | 2.38035E-013 | .043 | 1.6335E-014 | 9.8860E-013 |
| NATaurine | BFHDay1 | PFHDay1 | 9.99635E-014 | 1.45715E-013 | .499 | -1.9902E-013 | 3.9895E-013 |

| | | | | | | |
|---|---|---|---|---|---|---|
| | BMHDay1 | 8.75911E-014 | 1.53597E-013 | .573 | -2.2756E-013 | 4.0275E-013 |
| | PMHDay1 | -9.29229E-014 | 1.45715E-013 | .529 | -3.9190E-013 | 2.0606E-013 |
| | BFHDay2 | 2.40771E-014 | 1.53597E-013 | .877 | -2.9108E-013 | 3.3923E-013 |
| | PFHDay2 | -1.19701E-014 | 1.45715E-013 | .935 | -3.1095E-013 | 2.8701E-013 |
| | BMHDay2 | -1.21831E-013 | 1.53597E-013 | .435 | -4.3699E-013 | 1.9332E-013 |
| | PMHDay2 | -2.27558E-015 | 1.53597E-013 | .988 | -3.1743E-013 | 3.1288E-013 |
| PFHDay1 | BFHDay1 | -9.99635E-014 | 1.45715E-013 | .499 | -3.9895E-013 | 1.9902E-013 |
| | BMHDay1 | -1.23725E-014 | 1.45715E-013 | .933 | -3.1135E-013 | 2.8661E-013 |
| | PMHDay1 | -1.92886E-013 | 1.37381E-013 | .172 | -4.7477E-013 | 8.8996E-014 |
| | BFHDay2 | -7.58864E-014 | 1.45715E-013 | .607 | -3.7487E-013 | 2.2310E-013 |
| | PFHDay2 | -1.11934E-013 | 1.37381E-013 | .422 | -3.9382E-013 | 1.6995E-013 |
| | BMHDay2 | -2.21795E-013 | 1.45715E-013 | .140 | -5.2078E-013 | 7.7187E-014 |
| | PMHDay2 | -1.02239E-013 | 1.45715E-013 | .489 | -4.0122E-013 | 1.9674E-013 |
| BMHDay1 | BFHDay1 | -8.75911E-014 | 1.53597E-013 | .573 | -4.0275E-013 | 2.2756E-013 |
| | PFHDay1 | 1.23725E-014 | 1.45715E-013 | .933 | -2.8661E-013 | 3.1135E-013 |
| | PMHDay1 | -1.80514E-013 | 1.45715E-013 | .226 | -4.7950E-013 | 1.1847E-013 |
| | BFHDay2 | -6.35140E-014 | 1.53597E-013 | .682 | -3.7867E-013 | 2.5164E-013 |
| | PFHDay2 | -9.95612E-014 | 1.45715E-013 | .500 | -3.9854E-013 | 1.9942E-013 |
| | BMHDay2 | -2.09422E-013 | 1.53597E-013 | .184 | -5.2458E-013 | 1.0573E-013 |

| | | | | | | |
|---|---|---|---|---|---|---|
| | PMHDay2 | -8.98667E-<br>014 | 1.53597E-<br>013 | .563 | -4.0502E-<br>013 | 2.2529E-<br>013 |
| PMHDay1 | BFHDay1 | 9.29229E-<br>014 | 1.45715E-<br>013 | .529 | -2.0606E-<br>013 | 3.9190E-<br>013 |
| | PFHDay1 | 1.92886E-<br>013 | 1.37381E-<br>013 | .172 | -8.8996E-<br>014 | 4.7477E-<br>013 |
| | BMHDay1 | 1.80514E-<br>013 | 1.45715E-<br>013 | .226 | -1.1847E-<br>013 | 4.7950E-<br>013 |
| | BFHDay2 | 1.17000E-<br>013 | 1.45715E-<br>013 | .429 | -1.8198E-<br>013 | 4.1598E-<br>013 |
| | PFHDay2 | 8.09528E-<br>014 | 1.37381E-<br>013 | .561 | -2.0093E-<br>013 | 3.6284E-<br>013 |
| | BMHDay2 | -2.89083E-<br>014 | 1.45715E-<br>013 | .844 | -3.2789E-<br>013 | 2.7007E-<br>013 |
| | PMHDay2 | 9.06473E-<br>014 | 1.45715E-<br>013 | .539 | -2.0833E-<br>013 | 3.8963E-<br>013 |
| BFHDay2 | BFHDay1 | -2.40771E-<br>014 | 1.53597E-<br>013 | .877 | -3.3923E-<br>013 | 2.9108E-<br>013 |
| | PFHDay1 | 7.58864E-<br>014 | 1.45715E-<br>013 | .607 | -2.2310E-<br>013 | 3.7487E-<br>013 |
| | BMHDay1 | 6.35140E-<br>014 | 1.53597E-<br>013 | .682 | -2.5164E-<br>013 | 3.7867E-<br>013 |
| | PMHDay1 | -1.17000E-<br>013 | 1.45715E-<br>013 | .429 | -4.1598E-<br>013 | 1.8198E-<br>013 |
| | PFHDay2 | -3.60472E-<br>014 | 1.45715E-<br>013 | .806 | -3.3503E-<br>013 | 2.6293E-<br>013 |
| | BMHDay2 | -1.45908E-<br>013 | 1.53597E-<br>013 | .351 | -4.6106E-<br>013 | 1.6925E-<br>013 |
| | PMHDay2 | -2.63527E-<br>014 | 1.53597E-<br>013 | .865 | -3.4151E-<br>013 | 2.8880E-<br>013 |
| PFHDay2 | BFHDay1 | 1.19701E-<br>014 | 1.45715E-<br>013 | .935 | -2.8701E-<br>013 | 3.1095E-<br>013 |
| | PFHDay1 | 1.11934E-<br>013 | 1.37381E-<br>013 | .422 | -1.6995E-<br>013 | 3.9382E-<br>013 |
| | BMHDay1 | 9.95612E-<br>014 | 1.45715E-<br>013 | .500 | -1.9942E-<br>013 | 3.9854E-<br>013 |
| | PMHDay1 | -8.09528E-<br>014 | 1.37381E-<br>013 | .561 | -3.6284E-<br>013 | 2.0093E-<br>013 |

| | | | | | | | |
|---|---|---|---|---|---|---|---|
| | | BFHDay2 | 3.60472E-014 | 1.45715E-013 | .806 | -2.6293E-013 | 3.3503E-013 |
| | | BMHDay2 | -1.09861E-013 | 1.45715E-013 | .457 | -4.0884E-013 | 1.8912E-013 |
| | | PMHDay2 | 9.69452E-015 | 1.45715E-013 | .947 | -2.8929E-013 | 3.0868E-013 |
| | BMHDay2 | BFHDay1 | 1.21831E-013 | 1.53597E-013 | .435 | -1.9332E-013 | 4.3699E-013 |
| | | PFHDay1 | 2.21795E-013 | 1.45715E-013 | .140 | -7.7187E-014 | 5.2078E-013 |
| | | BMHDay1 | 2.09422E-013 | 1.53597E-013 | .184 | -1.0573E-013 | 5.2458E-013 |
| | | PMHDay1 | 2.89083E-014 | 1.45715E-013 | .844 | -2.7007E-013 | 3.2789E-013 |
| | | BFHDay2 | 1.45908E-013 | 1.53597E-013 | .351 | -1.6925E-013 | 4.6106E-013 |
| | | PFHDay2 | 1.09861E-013 | 1.45715E-013 | .457 | -1.8912E-013 | 4.0884E-013 |
| | | PMHDay2 | 1.19556E-013 | 1.53597E-013 | .443 | -1.9560E-013 | 4.3471E-013 |
| | PMHDay2 | BFHDay1 | 2.27558E-015 | 1.53597E-013 | .988 | -3.1288E-013 | 3.1743E-013 |
| | | PFHDay1 | 1.02239E-013 | 1.45715E-013 | .489 | -1.9674E-013 | 4.0122E-013 |
| | | BMHDay1 | 8.98667E-014 | 1.53597E-013 | .563 | -2.2529E-013 | 4.0502E-013 |
| | | PMHDay1 | -9.06473E-014 | 1.45715E-013 | .539 | -3.8963E-013 | 2.0833E-013 |
| | | BFHDay2 | 2.63527E-014 | 1.53597E-013 | .865 | -2.8880E-013 | 3.4151E-013 |
| | | PFHDay2 | -9.69452E-015 | 1.45715E-013 | .947 | -3.0868E-013 | 2.8929E-013 |
| | | BMHDay2 | -1.19556E-013 | 1.53597E-013 | .443 | -4.3471E-013 | 1.9560E-013 |
| PalTaurine | BFHDay1 | PFHDay1 | 9.25484E-013 | 6.88800E-013 | .189 | -4.7756E-013 | 2.3285E-012 |
| | | BMHDay1 | 1.46003E-012* | 6.88800E-013 | .042 | 5.6991E-014 | 2.8631E-012 |

| | | | | | | |
|---|---|---|---|---|---|---|
| | PMHDay1 | 4.25812E-014 | 6.88800E-013 | .951 | -1.3605E-012 | 1.4456E-012 |
| | BFHDay2 | 5.44738E-013 | 6.88800E-013 | .435 | -8.5830E-013 | 1.9478E-012 |
| | PFHDay2 | -7.75675E-013 | 6.88800E-013 | .268 | -2.1787E-012 | 6.2736E-013 |
| | BMHDay2 | 4.38479E-013 | 6.88800E-013 | .529 | -9.6456E-013 | 1.8415E-012 |
| | PMHDay2 | -8.52564E-013 | 6.88800E-013 | .225 | -2.2556E-012 | 5.5048E-013 |
| PFHDay1 | BFHDay1 | -9.25484E-013 | 6.88800E-013 | .189 | -2.3285E-012 | 4.7756E-013 |
| | BMHDay1 | 5.34547E-013 | 6.88800E-013 | .443 | -8.6849E-013 | 1.9376E-012 |
| | PMHDay1 | -8.82902E-013 | 6.88800E-013 | .209 | -2.2859E-012 | 5.2014E-013 |
| | BFHDay2 | -3.80746E-013 | 6.88800E-013 | .584 | -1.7838E-012 | 1.0223E-012 |
| | PFHDay2 | -1.70116E-012* | 6.88800E-013 | .019 | -3.1042E-012 | -2.9812E-013 |
| | BMHDay2 | -4.87005E-013 | 6.88800E-013 | .485 | -1.8900E-012 | 9.1603E-013 |
| | PMHDay2 | -1.77805E-012* | 6.88800E-013 | .015 | -3.1811E-012 | -3.7501E-013 |
| BMHDay1 | BFHDay1 | -1.46003E-012* | 6.88800E-013 | .042 | -2.8631E-012 | -5.6991E-014 |
| | PFHDay1 | -5.34547E-013 | 6.88800E-013 | .443 | -1.9376E-012 | 8.6849E-013 |
| | PMHDay1 | -1.41745E-012* | 6.88800E-013 | .048 | -2.8205E-012 | -1.4409E-014 |
| | BFHDay2 | -9.15293E-013 | 6.88800E-013 | .193 | -2.3183E-012 | 4.8775E-013 |
| | PFHDay2 | -2.23571E-012* | 6.88800E-013 | .003 | -3.6387E-012 | -8.3267E-013 |
| | BMHDay2 | -1.02155E-012 | 6.88800E-013 | .148 | -2.4246E-012 | 3.8149E-013 |
| | PMHDay2 | -2.31259E-012* | 6.88800E-013 | .002 | -3.7156E-012 | -9.0955E-013 |

| | | | | | | | |
|---|---|---|---|---|---|---|---|
| | PMHDay1 | BFHDay1 | -4.25812E-014 | 6.88800E-013 | .951 | -1.4456E-012 | 1.3605E-012 |
| | | PFHDay1 | 8.82902E-013 | 6.88800E-013 | .209 | -5.2014E-013 | 2.2859E-012 |
| | | BMHDay1 | 1.41745E-012* | 6.88800E-013 | .048 | 1.4409E-014 | 2.8205E-012 |
| | | BFHDay2 | 5.02157E-013 | 6.88800E-013 | .471 | -9.0088E-013 | 1.9052E-012 |
| | | PFHDay2 | -8.18257E-013 | 6.88800E-013 | .244 | -2.2213E-012 | 5.8478E-013 |
| | | BMHDay2 | 3.95898E-013 | 6.88800E-013 | .569 | -1.0071E-012 | 1.7989E-012 |
| | | PMHDay2 | -8.95145E-013 | 6.88800E-013 | .203 | -2.2982E-012 | 5.0789E-013 |
| | BFHDay2 | BFHDay1 | -5.44738E-013 | 6.88800E-013 | .435 | -1.9478E-012 | 8.5830E-013 |
| | | PFHDay1 | 3.80746E-013 | 6.88800E-013 | .584 | -1.0223E-012 | 1.7838E-012 |
| | | BMHDay1 | 9.15293E-013 | 6.88800E-013 | .193 | -4.8775E-013 | 2.3183E-012 |
| | | PMHDay1 | -5.02157E-013 | 6.88800E-013 | .471 | -1.9052E-012 | 9.0088E-013 |
| | | PFHDay2 | -1.32041E-012 | 6.88800E-013 | .064 | -2.7235E-012 | 8.2627E-014 |
| | | BMHDay2 | -1.06259E-013 | 6.88800E-013 | .878 | -1.5093E-012 | 1.2968E-012 |
| | | PMHDay2 | -1.39730E-012 | 6.88800E-013 | .051 | -2.8003E-012 | 5.7380E-015 |
| | PFHDay2 | BFHDay1 | 7.75675E-013 | 6.88800E-013 | .268 | -6.2736E-013 | 2.1787E-012 |
| | | PFHDay1 | 1.70116E-012* | 6.88800E-013 | .019 | 2.9812E-013 | 3.1042E-012 |
| | | BMHDay1 | 2.23571E-012* | 6.88800E-013 | .003 | 8.3267E-013 | 3.6387E-012 |
| | | PMHDay1 | 8.18257E-013 | 6.88800E-013 | .244 | -5.8478E-013 | 2.2213E-012 |
| | | BFHDay2 | 1.32041E-012 | 6.88800E-013 | .064 | -8.2627E-014 | 2.7235E-012 |

| | | | | | | | |
|---|---|---|---|---|---|---|---|
| | | BMHDay2 | 1.21415E-012 | 6.88800E-013 | .087 | -1.8889E-013 | 2.6172E-012 |
| | | PMHDay2 | -7.68886E-014 | 6.88800E-013 | .912 | -1.4799E-012 | 1.3262E-012 |
| | BMHDay2 | BFHDay1 | -4.38479E-013 | 6.88800E-013 | .529 | -1.8415E-012 | 9.6456E-013 |
| | | PFHDay1 | 4.87005E-013 | 6.88800E-013 | .485 | -9.1603E-013 | 1.8900E-012 |
| | | BMHDay1 | 1.02155E-012 | 6.88800E-013 | .148 | -3.8149E-013 | 2.4246E-012 |
| | | PMHDay1 | -3.95898E-013 | 6.88800E-013 | .569 | -1.7989E-012 | 1.0071E-012 |
| | | BFHDay2 | 1.06259E-013 | 6.88800E-013 | .878 | -1.2968E-012 | 1.5093E-012 |
| | | PFHDay2 | -1.21415E-012 | 6.88800E-013 | .087 | -2.6172E-012 | 1.8889E-013 |
| | | PMHDay2 | -1.29104E-012 | 6.88800E-013 | .070 | -2.6941E-012 | 1.1200E-013 |
| | PMHDay2 | BFHDay1 | 8.52564E-013 | 6.88800E-013 | .225 | -5.5048E-013 | 2.2556E-012 |
| | | PFHDay1 | 1.77805E-012* | 6.88800E-013 | .015 | 3.7501E-013 | 3.1811E-012 |
| | | BMHDay1 | 2.31259E-012* | 6.88800E-013 | .002 | 9.0955E-013 | 3.7156E-012 |
| | | PMHDay1 | 8.95145E-013 | 6.88800E-013 | .203 | -5.0789E-013 | 2.2982E-012 |
| | | BFHDay2 | 1.39730E-012 | 6.88800E-013 | .051 | -5.7380E-015 | 2.8003E-012 |
| | | PFHDay2 | 7.68886E-014 | 6.88800E-013 | .912 | -1.3262E-012 | 1.4799E-012 |
| | | BMHDay2 | 1.29104E-012 | 6.88800E-013 | .070 | -1.1200E-013 | 2.6941E-012 |
| PalLeucine | BFHDay1 | PFHDay1 | -9.29580E-014 | 2.97367E-012 | .975 | -6.1501E-012 | 5.9642E-012 |
| | | BMHDay1 | 3.58660E-012 | 2.97367E-012 | .237 | -2.4706E-012 | 9.6438E-012 |
| | | PMHDay1 | -2.78138E-012 | 2.97367E-012 | .357 | -8.8386E-012 | 3.2758E-012 |

| | | | | | | |
|---|---|---|---|---|---|---|
| | BFHDay2 | 1.81662E-012 | 2.97367E-012 | .546 | -4.2406E-012 | 7.8738E-012 |
| | PFHDay2 | -2.22497E-012 | 2.97367E-012 | .460 | -8.2821E-012 | 3.8322E-012 |
| | BMHDay2 | -7.32100E-014 | 2.97367E-012 | .981 | -6.1304E-012 | 5.9840E-012 |
| | PMHDay2 | -3.74625E-012 | 2.97367E-012 | .217 | -9.8034E-012 | 2.3109E-012 |
| PFHDay1 | BFHDay1 | 9.29580E-014 | 2.97367E-012 | .975 | -5.9642E-012 | 6.1501E-012 |
| | BMHDay1 | 3.67956E-012 | 2.97367E-012 | .225 | -2.3776E-012 | 9.7367E-012 |
| | PMHDay1 | -2.68842E-012 | 2.97367E-012 | .373 | -8.7456E-012 | 3.3688E-012 |
| | BFHDay2 | 1.90958E-012 | 2.97367E-012 | .525 | -4.1476E-012 | 7.9668E-012 |
| | PFHDay2 | -2.13202E-012 | 2.97367E-012 | .479 | -8.1892E-012 | 3.9252E-012 |
| | BMHDay2 | 1.97480E-014 | 2.97367E-012 | .995 | -6.0374E-012 | 6.0769E-012 |
| | PMHDay2 | -3.65329E-012 | 2.97367E-012 | .228 | -9.7105E-012 | 2.4039E-012 |
| BMHDay1 | BFHDay1 | -3.58660E-012 | 2.97367E-012 | .237 | -9.6438E-012 | 2.4706E-012 |
| | PFHDay1 | -3.67956E-012 | 2.97367E-012 | .225 | -9.7367E-012 | 2.3776E-012 |
| | PMHDay1 | -6.36798E-012* | 2.97367E-012 | .040 | -1.2425E-011 | -3.1080E-013 |
| | BFHDay2 | -1.76998E-012 | 2.97367E-012 | .556 | -7.8272E-012 | 4.2872E-012 |
| | PFHDay2 | -5.81157E-012 | 2.97367E-012 | .059 | -1.1869E-011 | 2.4560E-013 |
| | BMHDay2 | -3.65981E-012 | 2.97367E-012 | .227 | -9.7170E-012 | 2.3974E-012 |
| | PMHDay2 | -7.33285E-012* | 2.97367E-012 | .019 | -1.3390E-011 | -1.2757E-012 |
| PMHDay1 | BFHDay1 | 2.78138E-012 | 2.97367E-012 | .357 | -3.2758E-012 | 8.8386E-012 |

| | | | | | | |
|---|---|---|---|---|---|---|
| | PFHDay1 | 2.68842E-012 | 2.97367E-012 | .373 | -3.3688E-012 | 8.7456E-012 |
| | BMHDay1 | 6.36798E-012* | 2.97367E-012 | .040 | 3.1080E-013 | 1.2425E-011 |
| | BFHDay2 | 4.59800E-012 | 2.97367E-012 | .132 | -1.4592E-012 | 1.0655E-011 |
| | PFHDay2 | 5.56404E-013 | 2.97367E-012 | .853 | -5.5008E-012 | 6.6136E-012 |
| | BMHDay2 | 2.70817E-012 | 2.97367E-012 | .369 | -3.3490E-012 | 8.7653E-012 |
| | PMHDay2 | -9.64874E-013 | 2.97367E-012 | .748 | -7.0220E-012 | 5.0923E-012 |
| BFHDay2 | BFHDay1 | -1.81662E-012 | 2.97367E-012 | .546 | -7.8738E-012 | 4.2406E-012 |
| | PFHDay1 | -1.90958E-012 | 2.97367E-012 | .525 | -7.9668E-012 | 4.1476E-012 |
| | BMHDay1 | 1.76998E-012 | 2.97367E-012 | .556 | -4.2872E-012 | 7.8272E-012 |
| | PMHDay1 | -4.59800E-012 | 2.97367E-012 | .132 | -1.0655E-011 | 1.4592E-012 |
| | PFHDay2 | -4.04159E-012 | 2.97367E-012 | .184 | -1.0099E-011 | 2.0156E-012 |
| | BMHDay2 | -1.88983E-012 | 2.97367E-012 | .530 | -7.9470E-012 | 4.1673E-012 |
| | PMHDay2 | -5.56287E-012 | 2.97367E-012 | .071 | -1.1620E-011 | 4.9430E-013 |
| PFHDay2 | BFHDay1 | 2.22497E-012 | 2.97367E-012 | .460 | -3.8322E-012 | 8.2821E-012 |
| | PFHDay1 | 2.13202E-012 | 2.97367E-012 | .479 | -3.9252E-012 | 8.1892E-012 |
| | BMHDay1 | 5.81157E-012 | 2.97367E-012 | .059 | -2.4560E-013 | 1.1869E-011 |
| | PMHDay1 | -5.56404E-013 | 2.97367E-012 | .853 | -6.6136E-012 | 5.5008E-012 |
| | BFHDay2 | 4.04159E-012 | 2.97367E-012 | .184 | -2.0156E-012 | 1.0099E-011 |
| | BMHDay2 | 2.15176E-012 | 2.97367E-012 | .475 | -3.9054E-012 | 8.2089E-012 |

| | | | | | | | |
|---|---|---|---|---|---|---|---|
| | | PMHDay2 | -1.52128E-012 | 2.97367E-012 | .612 | -7.5785E-012 | 4.5359E-012 |
| | BMHDay2 | BFHDay1 | 7.32100E-014 | 2.97367E-012 | .981 | -5.9840E-012 | 6.1304E-012 |
| | | PFHDay1 | -1.97480E-014 | 2.97367E-012 | .995 | -6.0769E-012 | 6.0374E-012 |
| | | BMHDay1 | 3.65981E-012 | 2.97367E-012 | .227 | -2.3974E-012 | 9.7170E-012 |
| | | PMHDay1 | -2.70817E-012 | 2.97367E-012 | .369 | -8.7653E-012 | 3.3490E-012 |
| | | BFHDay2 | 1.88983E-012 | 2.97367E-012 | .530 | -4.1673E-012 | 7.9470E-012 |
| | | PFHDay2 | -2.15176E-012 | 2.97367E-012 | .475 | -8.2089E-012 | 3.9054E-012 |
| | | PMHDay2 | -3.67304E-012 | 2.97367E-012 | .226 | -9.7302E-012 | 2.3841E-012 |
| | PMHDay2 | BFHDay1 | 3.74625E-012 | 2.97367E-012 | .217 | -2.3109E-012 | 9.8034E-012 |
| | | PFHDay1 | 3.65329E-012 | 2.97367E-012 | .228 | -2.4039E-012 | 9.7105E-012 |
| | | BMHDay1 | 7.33285E-012* | 2.97367E-012 | .019 | 1.2757E-012 | 1.3390E-011 |
| | | PMHDay1 | 9.64874E-013 | 2.97367E-012 | .748 | -5.0923E-012 | 7.0220E-012 |
| | | BFHDay2 | 5.56287E-012 | 2.97367E-012 | .071 | -4.9430E-013 | 1.1620E-011 |
| | | PFHDay2 | 1.52128E-012 | 2.97367E-012 | .612 | -4.5359E-012 | 7.5785E-012 |
| | | BMHDay2 | 3.67304E-012 | 2.97367E-012 | .226 | -2.3841E-012 | 9.7302E-012 |
| LinLeucine | BFHDay1 | PFHDay1 | 7.69804E-013 | 1.55339E-012 | .624 | -2.3944E-012 | 3.9340E-012 |
| | | BMHDay1 | 2.35382E-013 | 1.55339E-012 | .881 | -2.9288E-012 | 3.3995E-012 |
| | | PMHDay1 | -8.37014E-013 | 1.55339E-012 | .594 | -4.0012E-012 | 2.3271E-012 |
| | | BFHDay2 | 4.23078E-013 | 1.55339E-012 | .787 | -2.7411E-012 | 3.5872E-012 |

| | | | | | | |
|---|---|---|---|---|---|---|
| | PFHDay2 | -4.33478E-013 | 1.55339E-012 | .782 | -3.5976E-012 | 2.7307E-012 |
| | BMHDay2 | -2.40587E-012 | 1.55339E-012 | .131 | -5.5700E-012 | 7.5829E-013 |
| | PMHDay2 | -5.49981E-012* | 1.55339E-012 | .001 | -8.6640E-012 | -2.3357E-012 |
| PFHDay1 | BFHDay1 | -7.69804E-013 | 1.55339E-012 | .624 | -3.9340E-012 | 2.3944E-012 |
| | BMHDay1 | -5.34422E-013 | 1.55339E-012 | .733 | -3.6986E-012 | 2.6297E-012 |
| | PMHDay1 | -1.60682E-012 | 1.55339E-012 | .309 | -4.7710E-012 | 1.5573E-012 |
| | BFHDay2 | -3.46726E-013 | 1.55339E-012 | .825 | -3.5109E-012 | 2.8174E-012 |
| | PFHDay2 | -1.20328E-012 | 1.55339E-012 | .444 | -4.3674E-012 | 1.9609E-012 |
| | BMHDay2 | -3.17567E-012* | 1.55339E-012 | .049 | -6.3398E-012 | -1.1516E-014 |
| | PMHDay2 | -6.26961E-012* | 1.55339E-012 | <.001 | -9.4338E-012 | -3.1055E-012 |
| BMHDay1 | BFHDay1 | -2.35382E-013 | 1.55339E-012 | .881 | -3.3995E-012 | 2.9288E-012 |
| | PFHDay1 | 5.34422E-013 | 1.55339E-012 | .733 | -2.6297E-012 | 3.6986E-012 |
| | PMHDay1 | -1.07240E-012 | 1.55339E-012 | .495 | -4.2366E-012 | 2.0918E-012 |
| | BFHDay2 | 1.87696E-013 | 1.55339E-012 | .905 | -2.9765E-012 | 3.3519E-012 |
| | PFHDay2 | -6.68860E-013 | 1.55339E-012 | .670 | -3.8330E-012 | 2.4953E-012 |
| | BMHDay2 | -2.64125E-012 | 1.55339E-012 | .099 | -5.8054E-012 | 5.2291E-013 |
| | PMHDay2 | -5.73519E-012* | 1.55339E-012 | <.001 | -8.8993E-012 | -2.5710E-012 |
| PMHDay1 | BFHDay1 | 8.37014E-013 | 1.55339E-012 | .594 | -2.3271E-012 | 4.0012E-012 |
| | PFHDay1 | 1.60682E-012 | 1.55339E-012 | .309 | -1.5573E-012 | 4.7710E-012 |

| | | | | | | |
|---|---|---|---|---|---|---|
| | BMHDay1 | 1.07240E-012 | 1.55339E-012 | .495 | -2.0918E-012 | 4.2366E-012 |
| | BFHDay2 | 1.26009E-012 | 1.55339E-012 | .423 | -1.9041E-012 | 4.4242E-012 |
| | PFHDay2 | 4.03536E-013 | 1.55339E-012 | .797 | -2.7606E-012 | 3.5677E-012 |
| | BMHDay2 | -1.56885E-012 | 1.55339E-012 | .320 | -4.7330E-012 | 1.5953E-012 |
| | PMHDay2 | -4.66279E-012* | 1.55339E-012 | .005 | -7.8269E-012 | -1.4986E-012 |
| BFHDay2 | BFHDay1 | -4.23078E-013 | 1.55339E-012 | .787 | -3.5872E-012 | 2.7411E-012 |
| | PFHDay1 | 3.46726E-013 | 1.55339E-012 | .825 | -2.8174E-012 | 3.5109E-012 |
| | BMHDay1 | -1.87696E-013 | 1.55339E-012 | .905 | -3.3519E-012 | 2.9765E-012 |
| | PMHDay1 | -1.26009E-012 | 1.55339E-012 | .423 | -4.4242E-012 | 1.9041E-012 |
| | PFHDay2 | -8.56556E-013 | 1.55339E-012 | .585 | -4.0207E-012 | 2.3076E-012 |
| | BMHDay2 | -2.82894E-012 | 1.55339E-012 | .078 | -5.9931E-012 | 3.3521E-013 |
| | PMHDay2 | -5.92288E-012* | 1.55339E-012 | <.001 | -9.0870E-012 | -2.7587E-012 |
| PFHDay2 | BFHDay1 | 4.33478E-013 | 1.55339E-012 | .782 | -2.7307E-012 | 3.5976E-012 |
| | PFHDay1 | 1.20328E-012 | 1.55339E-012 | .444 | -1.9609E-012 | 4.3674E-012 |
| | BMHDay1 | 6.68860E-013 | 1.55339E-012 | .670 | -2.4953E-012 | 3.8330E-012 |
| | PMHDay1 | -4.03536E-013 | 1.55339E-012 | .797 | -3.5677E-012 | 2.7606E-012 |
| | BFHDay2 | 8.56556E-013 | 1.55339E-012 | .585 | -2.3076E-012 | 4.0207E-012 |
| | BMHDay2 | -1.97239E-012 | 1.55339E-012 | .213 | -5.1365E-012 | 1.1918E-012 |
| | PMHDay2 | -5.06633E-012* | 1.55339E-012 | .003 | -8.2305E-012 | -1.9022E-012 |

| | | | | | | | |
|---|---|---|---|---|---|---|---|
| | BMHDay2 | BFHDay1 | 2.40587E-012 | 1.55339E-012 | .131 | -7.5829E-013 | 5.5700E-012 |
| | | PFHDay1 | 3.17567E-012* | 1.55339E-012 | .049 | 1.1516E-014 | 6.3398E-012 |
| | | BMHDay1 | 2.64125E-012 | 1.55339E-012 | .099 | -5.2291E-013 | 5.8054E-012 |
| | | PMHDay1 | 1.56885E-012 | 1.55339E-012 | .320 | -1.5953E-012 | 4.7330E-012 |
| | | BFHDay2 | 2.82894E-012 | 1.55339E-012 | .078 | -3.3521E-013 | 5.9931E-012 |
| | | PFHDay2 | 1.97239E-012 | 1.55339E-012 | .213 | -1.1918E-012 | 5.1365E-012 |
| | | PMHDay2 | -3.09394E-012 | 1.55339E-012 | .055 | -6.2581E-012 | 7.0214E-014 |
| | PMHDay2 | BFHDay1 | 5.49981E-012* | 1.55339E-012 | .001 | 2.3357E-012 | 8.6640E-012 |
| | | PFHDay1 | 6.26961E-012* | 1.55339E-012 | <.001 | 3.1055E-012 | 9.4338E-012 |
| | | BMHDay1 | 5.73519E-012* | 1.55339E-012 | <.001 | 2.5710E-012 | 8.8993E-012 |
| | | PMHDay1 | 4.66279E-012* | 1.55339E-012 | .005 | 1.4986E-012 | 7.8269E-012 |
| | | BFHDay2 | 5.92288E-012* | 1.55339E-012 | <.001 | 2.7587E-012 | 9.0870E-012 |
| | | PFHDay2 | 5.06633E-012* | 1.55339E-012 | .003 | 1.9022E-012 | 8.2305E-012 |
| | | BMHDay2 | 3.09394E-012 | 1.55339E-012 | .055 | -7.0214E-014 | 6.2581E-012 |
| DocLeucine | BFHDay1 | PFHDay1 | -1.52079E-013 | 3.56047E-013 | .672 | -8.7732E-013 | 5.7316E-013 |
| | | BMHDay1 | 4.00764E-014 | 3.56047E-013 | .911 | -6.8517E-013 | 7.6532E-013 |
| | | PMHDay1 | -8.99366E-013* | 3.56047E-013 | .017 | -1.6246E-012 | -1.7412E-013 |
| | | BFHDay2 | 5.80848E-014 | 3.56047E-013 | .871 | -6.6716E-013 | 7.8333E-013 |
| | | PFHDay2 | -1.67236E-013 | 3.56047E-013 | .642 | -8.9248E-013 | 5.5801E-013 |

| | | | | | | |
|---|---|---|---|---|---|---|
| | BMHDay2 | -7.44198E-014 | 3.56047E-013 | .836 | -7.9966E-013 | 6.5082E-013 |
| | PMHDay2 | 1.18640E-013 | 3.56047E-013 | .741 | -6.0660E-013 | 8.4388E-013 |
| PFHDay1 | BFHDay1 | 1.52079E-013 | 3.56047E-013 | .672 | -5.7316E-013 | 8.7732E-013 |
| | BMHDay1 | 1.92156E-013 | 3.56047E-013 | .593 | -5.3309E-013 | 9.1740E-013 |
| | PMHDay1 | -7.47287E-013* | 3.56047E-013 | .044 | -1.4725E-012 | -2.2043E-014 |
| | BFHDay2 | 2.10164E-013 | 3.56047E-013 | .559 | -5.1508E-013 | 9.3541E-013 |
| | PFHDay2 | -1.51568E-014 | 3.56047E-013 | .966 | -7.4040E-013 | 7.1009E-013 |
| | BMHDay2 | 7.76594E-014 | 3.56047E-013 | .829 | -6.4758E-013 | 8.0290E-013 |
| | PMHDay2 | 2.70720E-013 | 3.56047E-013 | .453 | -4.5452E-013 | 9.9596E-013 |
| BMHDay1 | BFHDay1 | -4.00764E-014 | 3.56047E-013 | .911 | -7.6532E-013 | 6.8517E-013 |
| | PFHDay1 | -1.92156E-013 | 3.56047E-013 | .593 | -9.1740E-013 | 5.3309E-013 |
| | PMHDay1 | -9.39443E-013* | 3.56047E-013 | .013 | -1.6647E-012 | -2.1420E-013 |
| | BFHDay2 | 1.80084E-014 | 3.56047E-013 | .960 | -7.0724E-013 | 7.4325E-013 |
| | PFHDay2 | -2.07312E-013 | 3.56047E-013 | .564 | -9.3256E-013 | 5.1793E-013 |
| | BMHDay2 | -1.14496E-013 | 3.56047E-013 | .750 | -8.3974E-013 | 6.1075E-013 |
| | PMHDay2 | 7.85640E-014 | 3.56047E-013 | .827 | -6.4668E-013 | 8.0381E-013 |
| PMHDay1 | BFHDay1 | 8.99366E-013* | 3.56047E-013 | .017 | 1.7412E-013 | 1.6246E-012 |
| | PFHDay1 | 7.47287E-013* | 3.56047E-013 | .044 | 2.2043E-014 | 1.4725E-012 |
| | BMHDay1 | 9.39443E-013* | 3.56047E-013 | .013 | 2.1420E-013 | 1.6647E-012 |

| | | | | | | |
|---|---|---|---|---|---|---|
| | BFHDay2 | 9.57451E-013* | 3.56047E-013 | .011 | 2.3221E-013 | 1.6827E-012 |
| | PFHDay2 | 7.32130E-013* | 3.56047E-013 | .048 | 6.8863E-015 | 1.4574E-012 |
| | BMHDay2 | 8.24946E-013* | 3.56047E-013 | .027 | 9.9703E-014 | 1.5502E-012 |
| | PMHDay2 | 1.01801E-012* | 3.56047E-013 | .007 | 2.9276E-013 | 1.7433E-012 |
| BFHDay2 | BFHDay1 | -5.80848E-014 | 3.56047E-013 | .871 | -7.8333E-013 | 6.6716E-013 |
| | PFHDay1 | -2.10164E-013 | 3.56047E-013 | .559 | -9.3541E-013 | 5.1508E-013 |
| | BMHDay1 | -1.80084E-014 | 3.56047E-013 | .960 | -7.4325E-013 | 7.0724E-013 |
| | PMHDay1 | -9.57451E-013* | 3.56047E-013 | .011 | -1.6827E-012 | -2.3221E-013 |
| | PFHDay2 | -2.25321E-013 | 3.56047E-013 | .531 | -9.5056E-013 | 4.9992E-013 |
| | BMHDay2 | -1.32505E-013 | 3.56047E-013 | .712 | -8.5775E-013 | 5.9274E-013 |
| | PMHDay2 | 6.05556E-014 | 3.56047E-013 | .866 | -6.6469E-013 | 7.8580E-013 |
| PFHDay2 | BFHDay1 | 1.67236E-013 | 3.56047E-013 | .642 | -5.5801E-013 | 8.9248E-013 |
| | PFHDay1 | 1.51568E-014 | 3.56047E-013 | .966 | -7.1009E-013 | 7.4040E-013 |
| | BMHDay1 | 2.07312E-013 | 3.56047E-013 | .564 | -5.1793E-013 | 9.3256E-013 |
| | PMHDay1 | -7.32130E-013* | 3.56047E-013 | .048 | -1.4574E-012 | -6.8863E-015 |
| | BFHDay2 | 2.25321E-013 | 3.56047E-013 | .531 | -4.9992E-013 | 9.5056E-013 |
| | BMHDay2 | 9.28162E-014 | 3.56047E-013 | .796 | -6.3243E-013 | 8.1806E-013 |
| | PMHDay2 | 2.85876E-013 | 3.56047E-013 | .428 | -4.3937E-013 | 1.0111E-012 |
| BMHDay2 | BFHDay1 | 7.44198E-014 | 3.56047E-013 | .836 | -6.5082E-013 | 7.9966E-013 |

| | | | | | | | |
|---|---|---|---|---|---|---|---|
| | | PFHDay1 | -7.76594E-014 | 3.56047E-013 | .829 | -8.0290E-013 | 6.4758E-013 |
| | | BMHDay1 | 1.14496E-013 | 3.56047E-013 | .750 | -6.1075E-013 | 8.3974E-013 |
| | | PMHDay1 | -8.24946E-013* | 3.56047E-013 | .027 | -1.5502E-012 | -9.9703E-014 |
| | | BFHDay2 | 1.32505E-013 | 3.56047E-013 | .712 | -5.9274E-013 | 8.5775E-013 |
| | | PFHDay2 | -9.28162E-014 | 3.56047E-013 | .796 | -8.1806E-013 | 6.3243E-013 |
| | | PMHDay2 | 1.93060E-013 | 3.56047E-013 | .591 | -5.3218E-013 | 9.1830E-013 |
| | PMHDay2 | BFHDay1 | -1.18640E-013 | 3.56047E-013 | .741 | -8.4388E-013 | 6.0660E-013 |
| | | PFHDay1 | -2.70720E-013 | 3.56047E-013 | .453 | -9.9596E-013 | 4.5452E-013 |
| | | BMHDay1 | -7.85640E-014 | 3.56047E-013 | .827 | -8.0381E-013 | 6.4668E-013 |
| | | PMHDay1 | -1.01801E-012* | 3.56047E-013 | .007 | -1.7433E-012 | -2.9276E-013 |
| | | BFHDay2 | -6.05556E-014 | 3.56047E-013 | .866 | -7.8580E-013 | 6.6469E-013 |
| | | PFHDay2 | -2.85876E-013 | 3.56047E-013 | .428 | -1.0111E-012 | 4.3937E-013 |
| | | BMHDay2 | -1.93060E-013 | 3.56047E-013 | .591 | -9.1830E-013 | 5.3218E-013 |
| StrLeucine | BFHDay1 | PFHDay1 | 6.23097E-013 | 7.67299E-013 | .423 | -9.3984E-013 | 2.1860E-012 |
| | | BMHDay1 | 1.26196E-012 | 7.67299E-013 | .110 | -3.0098E-013 | 2.8249E-012 |
| | | PMHDay1 | -7.83886E-013 | 7.67299E-013 | .315 | -2.3468E-012 | 7.7905E-013 |
| | | BFHDay2 | 7.97015E-013 | 7.67299E-013 | .307 | -7.6592E-013 | 2.3600E-012 |
| | | PFHDay2 | 1.34370E-013 | 7.67299E-013 | .862 | -1.4286E-012 | 1.6973E-012 |
| | | BMHDay2 | 1.26010E-013 | 7.67299E-013 | .871 | -1.4369E-012 | 1.6889E-012 |

| | | | | | | |
|---|---|---|---|---|---|---|
| | PMHDay2 | -1.10192E-012 | 7.67299E-013 | .161 | -2.6649E-012 | 4.6102E-013 |
| PFHDay1 | BFHDay1 | -6.23097E-013 | 7.67299E-013 | .423 | -2.1860E-012 | 9.3984E-013 |
| | BMHDay1 | 6.38859E-013 | 7.67299E-013 | .411 | -9.2408E-013 | 2.2018E-012 |
| | PMHDay1 | -1.40698E-012 | 7.67299E-013 | .076 | -2.9699E-012 | 1.5595E-013 |
| | BFHDay2 | 1.73919E-013 | 7.67299E-013 | .822 | -1.3890E-012 | 1.7369E-012 |
| | PFHDay2 | -4.88727E-013 | 7.67299E-013 | .529 | -2.0517E-012 | 1.0742E-012 |
| | BMHDay2 | -4.97087E-013 | 7.67299E-013 | .522 | -2.0600E-012 | 1.0659E-012 |
| | PMHDay2 | -1.72502E-012* | 7.67299E-013 | .032 | -3.2880E-012 | -1.6208E-013 |
| BMHDay1 | BFHDay1 | -1.26196E-012 | 7.67299E-013 | .110 | -2.8249E-012 | 3.0098E-013 |
| | PFHDay1 | -6.38859E-013 | 7.67299E-013 | .411 | -2.2018E-012 | 9.2408E-013 |
| | PMHDay1 | -2.04584E-012* | 7.67299E-013 | .012 | -3.6088E-012 | -4.8290E-013 |
| | BFHDay2 | -4.64941E-013 | 7.67299E-013 | .549 | -2.0279E-012 | 1.0980E-012 |
| | PFHDay2 | -1.12759E-012 | 7.67299E-013 | .151 | -2.6905E-012 | 4.3535E-013 |
| | BMHDay2 | -1.13595E-012 | 7.67299E-013 | .149 | -2.6989E-012 | 4.2699E-013 |
| | PMHDay2 | -2.36388E-012* | 7.67299E-013 | .004 | -3.9268E-012 | -8.0094E-013 |
| PMHDay1 | BFHDay1 | 7.83886E-013 | 7.67299E-013 | .315 | -7.7905E-013 | 2.3468E-012 |
| | PFHDay1 | 1.40698E-012 | 7.67299E-013 | .076 | -1.5595E-013 | 2.9699E-012 |
| | BMHDay1 | 2.04584E-012* | 7.67299E-013 | .012 | 4.8290E-013 | 3.6088E-012 |
| | BFHDay2 | 1.58090E-012* | 7.67299E-013 | .048 | 1.7964E-014 | 3.1438E-012 |

| | | | | | | |
|---|---|---|---|---|---|---|
| | PFHDay2 | 9.18256E-013 | 7.67299E-013 | .240 | -6.4468E-013 | 2.4812E-012 |
| | BMHDay2 | 9.09896E-013 | 7.67299E-013 | .244 | -6.5304E-013 | 2.4728E-012 |
| | PMHDay2 | -3.18036E-013 | 7.67299E-013 | .681 | -1.8810E-012 | 1.2449E-012 |
| BFHDay2 | BFHDay1 | -7.97015E-013 | 7.67299E-013 | .307 | -2.3600E-012 | 7.6592E-013 |
| | PFHDay1 | -1.73919E-013 | 7.67299E-013 | .822 | -1.7369E-012 | 1.3890E-012 |
| | BMHDay1 | 4.64941E-013 | 7.67299E-013 | .549 | -1.0980E-012 | 2.0279E-012 |
| | PMHDay1 | -1.58090E-012* | 7.67299E-013 | .048 | -3.1438E-012 | -1.7964E-014 |
| | PFHDay2 | -6.62646E-013 | 7.67299E-013 | .394 | -2.2256E-012 | 9.0029E-013 |
| | BMHDay2 | -6.71006E-013 | 7.67299E-013 | .388 | -2.2339E-012 | 8.9193E-013 |
| | PMHDay2 | -1.89894E-012* | 7.67299E-013 | .019 | -3.4619E-012 | -3.3600E-013 |
| PFHDay2 | BFHDay1 | -1.34370E-013 | 7.67299E-013 | .862 | -1.6973E-012 | 1.4286E-012 |
| | PFHDay1 | 4.88727E-013 | 7.67299E-013 | .529 | -1.0742E-012 | 2.0517E-012 |
| | BMHDay1 | 1.12759E-012 | 7.67299E-013 | .151 | -4.3535E-013 | 2.6905E-012 |
| | PMHDay1 | -9.18256E-013 | 7.67299E-013 | .240 | -2.4812E-012 | 6.4468E-013 |
| | BFHDay2 | 6.62646E-013 | 7.67299E-013 | .394 | -9.0029E-013 | 2.2256E-012 |
| | BMHDay2 | -8.36000E-015 | 7.67299E-013 | .991 | -1.5713E-012 | 1.5546E-012 |
| | PMHDay2 | -1.23629E-012 | 7.67299E-013 | .117 | -2.7992E-012 | 3.2665E-013 |
| BMHDay2 | BFHDay1 | -1.26010E-013 | 7.67299E-013 | .871 | -1.6889E-012 | 1.4369E-012 |
| | PFHDay1 | 4.97087E-013 | 7.67299E-013 | .522 | -1.0659E-012 | 2.0600E-012 |

| | | | | | | | |
|---|---|---|---|---|---|---|---|
| | | BMHDay1 | 1.13595E-012 | 7.67299E-013 | .149 | -4.2699E-013 | 2.6989E-012 |
| | | PMHDay1 | -9.09896E-013 | 7.67299E-013 | .244 | -2.4728E-012 | 6.5304E-013 |
| | | BFHDay2 | 6.71006E-013 | 7.67299E-013 | .388 | -8.9193E-013 | 2.2339E-012 |
| | | PFHDay2 | 8.36000E-015 | 7.67299E-013 | .991 | -1.5546E-012 | 1.5713E-012 |
| | | PMHDay2 | -1.22793E-012 | 7.67299E-013 | .119 | -2.7909E-012 | 3.3501E-013 |
| | PMHDay2 | BFHDay1 | 1.10192E-012 | 7.67299E-013 | .161 | -4.6102E-013 | 2.6649E-012 |
| | | PFHDay1 | 1.72502E-012* | 7.67299E-013 | .032 | 1.6208E-013 | 3.2880E-012 |
| | | BMHDay1 | 2.36388E-012* | 7.67299E-013 | .004 | 8.0094E-013 | 3.9268E-012 |
| | | PMHDay1 | 3.18036E-013 | 7.67299E-013 | .681 | -1.2449E-012 | 1.8810E-012 |
| | | BFHDay2 | 1.89894E-012* | 7.67299E-013 | .019 | 3.3600E-013 | 3.4619E-012 |
| | | PFHDay2 | 1.23629E-012 | 7.67299E-013 | .117 | -3.2665E-013 | 2.7992E-012 |
| | | BMHDay2 | 1.22793E-012 | 7.67299E-013 | .119 | -3.3501E-013 | 2.7909E-012 |
| OlLeucine | BFHDay1 | PFHDay1 | 1.25364E-013 | 2.74153E-012 | .964 | -5.4590E-012 | 5.7097E-012 |
| | | BMHDay1 | 1.66190E-012 | 2.74153E-012 | .549 | -3.9224E-012 | 7.2462E-012 |
| | | PMHDay1 | -3.42431E-012 | 2.74153E-012 | .221 | -9.0086E-012 | 2.1600E-012 |
| | | BFHDay2 | 1.37412E-012 | 2.74153E-012 | .620 | -4.2102E-012 | 6.9584E-012 |
| | | PFHDay2 | -1.85554E-012 | 2.74153E-012 | .503 | -7.4399E-012 | 3.7288E-012 |
| | | BMHDay2 | -5.03002E-013 | 2.74153E-012 | .856 | -6.0873E-012 | 5.0813E-012 |
| | | PMHDay2 | -4.28761E-012 | 2.74153E-012 | .128 | -9.8719E-012 | 1.2967E-012 |

| | | | | | | |
|---|---|---|---|---|---|---|
| PFHDay1 | BFHDay1 | -1.25364E-013 | 2.74153E-012 | .964 | -5.7097E-012 | 5.4590E-012 |
| | BMHDay1 | 1.53653E-012 | 2.74153E-012 | .579 | -4.0478E-012 | 7.1209E-012 |
| | PMHDay1 | -3.54967E-012 | 2.74153E-012 | .205 | -9.1340E-012 | 2.0346E-012 |
| | BFHDay2 | 1.24876E-012 | 2.74153E-012 | .652 | -4.3356E-012 | 6.8331E-012 |
| | PFHDay2 | -1.98091E-012 | 2.74153E-012 | .475 | -7.5652E-012 | 3.6034E-012 |
| | BMHDay2 | -6.28366E-013 | 2.74153E-012 | .820 | -6.2127E-012 | 4.9560E-012 |
| | PMHDay2 | -4.41298E-012 | 2.74153E-012 | .117 | -9.9973E-012 | 1.1713E-012 |
| BMHDay1 | BFHDay1 | -1.66190E-012 | 2.74153E-012 | .549 | -7.2462E-012 | 3.9224E-012 |
| | PFHDay1 | -1.53653E-012 | 2.74153E-012 | .579 | -7.1209E-012 | 4.0478E-012 |
| | PMHDay1 | -5.08621E-012 | 2.74153E-012 | .073 | -1.0671E-011 | 4.9812E-013 |
| | BFHDay2 | -2.87778E-013 | 2.74153E-012 | .917 | -5.8721E-012 | 5.2965E-012 |
| | PFHDay2 | -3.51744E-012 | 2.74153E-012 | .209 | -9.1018E-012 | 2.0669E-012 |
| | BMHDay2 | -2.16490E-012 | 2.74153E-012 | .436 | -7.7492E-012 | 3.4194E-012 |
| | PMHDay2 | -5.94951E-012* | 2.74153E-012 | .038 | -1.1534E-011 | -3.6519E-013 |
| PMHDay1 | BFHDay1 | 3.42431E-012 | 2.74153E-012 | .221 | -2.1600E-012 | 9.0086E-012 |
| | PFHDay1 | 3.54967E-012 | 2.74153E-012 | .205 | -2.0346E-012 | 9.1340E-012 |
| | BMHDay1 | 5.08621E-012 | 2.74153E-012 | .073 | -4.9812E-013 | 1.0671E-011 |
| | BFHDay2 | 4.79843E-012 | 2.74153E-012 | .090 | -7.8589E-013 | 1.0383E-011 |
| | PFHDay2 | 1.56877E-012 | 2.74153E-012 | .571 | -4.0156E-012 | 7.1531E-012 |

| | | | | | | |
|---|---|---|---|---|---|---|
| | BMHDay2 | 2.92131E-012 | 2.74153E-012 | .295 | -2.6630E-012 | 8.5056E-012 |
| | PMHDay2 | -8.63306E-013 | 2.74153E-012 | .755 | -6.4476E-012 | 4.7210E-012 |
| BFHDay2 | BFHDay1 | -1.37412E-012 | 2.74153E-012 | .620 | -6.9584E-012 | 4.2102E-012 |
| | PFHDay1 | -1.24876E-012 | 2.74153E-012 | .652 | -6.8331E-012 | 4.3356E-012 |
| | BMHDay1 | 2.87778E-013 | 2.74153E-012 | .917 | -5.2965E-012 | 5.8721E-012 |
| | PMHDay1 | -4.79843E-012 | 2.74153E-012 | .090 | -1.0383E-011 | 7.8589E-013 |
| | PFHDay2 | -3.22966E-012 | 2.74153E-012 | .247 | -8.8140E-012 | 2.3547E-012 |
| | BMHDay2 | -1.87712E-012 | 2.74153E-012 | .498 | -7.4614E-012 | 3.7072E-012 |
| | PMHDay2 | -5.66173E-012* | 2.74153E-012 | .047 | -1.1246E-011 | -7.7412E-014 |
| PFHDay2 | BFHDay1 | 1.85554E-012 | 2.74153E-012 | .503 | -3.7288E-012 | 7.4399E-012 |
| | PFHDay1 | 1.98091E-012 | 2.74153E-012 | .475 | -3.6034E-012 | 7.5652E-012 |
| | BMHDay1 | 3.51744E-012 | 2.74153E-012 | .209 | -2.0669E-012 | 9.1018E-012 |
| | PMHDay1 | -1.56877E-012 | 2.74153E-012 | .571 | -7.1531E-012 | 4.0156E-012 |
| | BFHDay2 | 3.22966E-012 | 2.74153E-012 | .247 | -2.3547E-012 | 8.8140E-012 |
| | BMHDay2 | 1.35254E-012 | 2.74153E-012 | .625 | -4.2318E-012 | 6.9369E-012 |
| | PMHDay2 | -2.43207E-012 | 2.74153E-012 | .382 | -8.0164E-012 | 3.1522E-012 |
| BMHDay2 | BFHDay1 | 5.03002E-013 | 2.74153E-012 | .856 | -5.0813E-012 | 6.0873E-012 |
| | PFHDay1 | 6.28366E-013 | 2.74153E-012 | .820 | -4.9560E-012 | 6.2127E-012 |
| | BMHDay1 | 2.16490E-012 | 2.74153E-012 | .436 | -3.4194E-012 | 7.7492E-012 |

| | | | | | | | |
|---|---|---|---|---|---|---|---|
| | | PMHDay1 | -2.92131E-012 | 2.74153E-012 | .295 | -8.5056E-012 | 2.6630E-012 |
| | | BFHDay2 | 1.87712E-012 | 2.74153E-012 | .498 | -3.7072E-012 | 7.4614E-012 |
| | | PFHDay2 | -1.35254E-012 | 2.74153E-012 | .625 | -6.9369E-012 | 4.2318E-012 |
| | | PMHDay2 | -3.78461E-012 | 2.74153E-012 | .177 | -9.3689E-012 | 1.7997E-012 |
| | PMHDay2 | BFHDay1 | 4.28761E-012 | 2.74153E-012 | .128 | -1.2967E-012 | 9.8719E-012 |
| | | PFHDay1 | 4.41298E-012 | 2.74153E-012 | .117 | -1.1713E-012 | 9.9973E-012 |
| | | BMHDay1 | 5.94951E-012* | 2.74153E-012 | .038 | 3.6519E-013 | 1.1534E-011 |
| | | PMHDay1 | 8.63306E-013 | 2.74153E-012 | .755 | -4.7210E-012 | 6.4476E-012 |
| | | BFHDay2 | 5.66173E-012* | 2.74153E-012 | .047 | 7.7412E-014 | 1.1246E-011 |
| | | PFHDay2 | 2.43207E-012 | 2.74153E-012 | .382 | -3.1522E-012 | 8.0164E-012 |
| | | BMHDay2 | 3.78461E-012 | 2.74153E-012 | .177 | -1.7997E-012 | 9.3689E-012 |
| PalPhenyl | BFHDay1 | PFHDay1 | -4.05412E-013 | 3.37030E-012 | .905 | -7.2705E-012 | 6.4597E-012 |
| | | BMHDay1 | 2.94971E-012 | 3.37030E-012 | .388 | -3.9154E-012 | 9.8148E-012 |
| | | PMHDay1 | -2.65289E-012 | 3.37030E-012 | .437 | -9.5180E-012 | 4.2122E-012 |
| | | BFHDay2 | 7.36516E-013 | 3.37030E-012 | .828 | -6.1286E-012 | 7.6016E-012 |
| | | PFHDay2 | -2.16044E-012 | 3.37030E-012 | .526 | -9.0255E-012 | 4.7046E-012 |
| | | BMHDay2 | -1.09780E-012 | 3.37030E-012 | .747 | -7.9629E-012 | 5.7673E-012 |
| | | PMHDay2 | -7.16481E-012* | 3.37030E-012 | .041 | -1.4030E-011 | -2.9974E-013 |
| | PFHDay1 | BFHDay1 | 4.05412E-013 | 3.37030E-012 | .905 | -6.4597E-012 | 7.2705E-012 |

| | | | | | | |
|---|---|---|---|---|---|---|
| | BMHDay1 | 3.35512E-012 | 3.37030E-012 | .327 | -3.5099E-012 | 1.0220E-011 |
| | PMHDay1 | -2.24748E-012 | 3.37030E-012 | .510 | -9.1125E-012 | 4.6176E-012 |
| | BFHDay2 | 1.14193E-012 | 3.37030E-012 | .737 | -5.7231E-012 | 8.0070E-012 |
| | PFHDay2 | -1.75502E-012 | 3.37030E-012 | .606 | -8.6201E-012 | 5.1100E-012 |
| | BMHDay2 | -6.92390E-013 | 3.37030E-012 | .839 | -7.5575E-012 | 6.1727E-012 |
| | PMHDay2 | -6.75940E-012 | 3.37030E-012 | .053 | -1.3624E-011 | 1.0567E-013 |
| BMHDay1 | BFHDay1 | -2.94971E-012 | 3.37030E-012 | .388 | -9.8148E-012 | 3.9154E-012 |
| | PFHDay1 | -3.35512E-012 | 3.37030E-012 | .327 | -1.0220E-011 | 3.5099E-012 |
| | PMHDay1 | -5.60260E-012 | 3.37030E-012 | .106 | -1.2468E-011 | 1.2625E-012 |
| | BFHDay2 | -2.21319E-012 | 3.37030E-012 | .516 | -9.0783E-012 | 4.6519E-012 |
| | PFHDay2 | -5.11015E-012 | 3.37030E-012 | .139 | -1.1975E-011 | 1.7549E-012 |
| | BMHDay2 | -4.04751E-012 | 3.37030E-012 | .239 | -1.0913E-011 | 2.8176E-012 |
| | PMHDay2 | -1.01145E-011* | 3.37030E-012 | .005 | -1.6980E-011 | -3.2494E-012 |
| PMHDay1 | BFHDay1 | 2.65289E-012 | 3.37030E-012 | .437 | -4.2122E-012 | 9.5180E-012 |
| | PFHDay1 | 2.24748E-012 | 3.37030E-012 | .510 | -4.6176E-012 | 9.1125E-012 |
| | BMHDay1 | 5.60260E-012 | 3.37030E-012 | .106 | -1.2625E-012 | 1.2468E-011 |
| | BFHDay2 | 3.38941E-012 | 3.37030E-012 | .322 | -3.4757E-012 | 1.0254E-011 |
| | PFHDay2 | 4.92454E-013 | 3.37030E-012 | .885 | -6.3726E-012 | 7.3575E-012 |
| | BMHDay2 | 1.55509E-012 | 3.37030E-012 | .648 | -5.3100E-012 | 8.4202E-012 |

| | | | | | | |
|---|---|---|---|---|---|---|
| | PMHDay2 | -4.51192E-012 | 3.37030E-012 | .190 | -1.1377E-011 | 2.3532E-012 |
| BFHDay2 | BFHDay1 | -7.36516E-013 | 3.37030E-012 | .828 | -7.6016E-012 | 6.1286E-012 |
| | PFHDay1 | -1.14193E-012 | 3.37030E-012 | .737 | -8.0070E-012 | 5.7231E-012 |
| | BMHDay1 | 2.21319E-012 | 3.37030E-012 | .516 | -4.6519E-012 | 9.0783E-012 |
| | PMHDay1 | -3.38941E-012 | 3.37030E-012 | .322 | -1.0254E-011 | 3.4757E-012 |
| | PFHDay2 | -2.89695E-012 | 3.37030E-012 | .396 | -9.7620E-012 | 3.9681E-012 |
| | BMHDay2 | -1.83432E-012 | 3.37030E-012 | .590 | -8.6994E-012 | 5.0308E-012 |
| | PMHDay2 | -7.90132E-012* | 3.37030E-012 | .025 | -1.4766E-011 | -1.0363E-012 |
| PFHDay2 | BFHDay1 | 2.16044E-012 | 3.37030E-012 | .526 | -4.7046E-012 | 9.0255E-012 |
| | PFHDay1 | 1.75502E-012 | 3.37030E-012 | .606 | -5.1100E-012 | 8.6201E-012 |
| | BMHDay1 | 5.11015E-012 | 3.37030E-012 | .139 | -1.7549E-012 | 1.1975E-011 |
| | PMHDay1 | -4.92454E-013 | 3.37030E-012 | .885 | -7.3575E-012 | 6.3726E-012 |
| | BFHDay2 | 2.89695E-012 | 3.37030E-012 | .396 | -3.9681E-012 | 9.7620E-012 |
| | BMHDay2 | 1.06263E-012 | 3.37030E-012 | .755 | -5.8024E-012 | 7.9277E-012 |
| | PMHDay2 | -5.00437E-012 | 3.37030E-012 | .147 | -1.1869E-011 | 1.8607E-012 |
| BMHDay2 | BFHDay1 | 1.09780E-012 | 3.37030E-012 | .747 | -5.7673E-012 | 7.9629E-012 |
| | PFHDay1 | 6.92390E-013 | 3.37030E-012 | .839 | -6.1727E-012 | 7.5575E-012 |
| | BMHDay1 | 4.04751E-012 | 3.37030E-012 | .239 | -2.8176E-012 | 1.0913E-011 |
| | PMHDay1 | -1.55509E-012 | 3.37030E-012 | .648 | -8.4202E-012 | 5.3100E-012 |

| | | | | | | | |
|---|---|---|---|---|---|---|---|
| | | BFHDay2 | 1.83432E-012 | 3.37030E-012 | .590 | -5.0308E-012 | 8.6994E-012 |
| | | PFHDay2 | -1.06263E-012 | 3.37030E-012 | .755 | -7.9277E-012 | 5.8024E-012 |
| | | PMHDay2 | -6.06701E-012 | 3.37030E-012 | .081 | -1.2932E-011 | 7.9806E-013 |
| | PMHDay2 | BFHDay1 | 7.16481E-012* | 3.37030E-012 | .041 | 2.9974E-013 | 1.4030E-011 |
| | | PFHDay1 | 6.75940E-012 | 3.37030E-012 | .053 | -1.0567E-013 | 1.3624E-011 |
| | | BMHDay1 | 1.01145E-011* | 3.37030E-012 | .005 | 3.2494E-012 | 1.6980E-011 |
| | | PMHDay1 | 4.51192E-012 | 3.37030E-012 | .190 | -2.3532E-012 | 1.1377E-011 |
| | | BFHDay2 | 7.90132E-012* | 3.37030E-012 | .025 | 1.0363E-012 | 1.4766E-011 |
| | | PFHDay2 | 5.00437E-012 | 3.37030E-012 | .147 | -1.8607E-012 | 1.1869E-011 |
| | | BMHDay2 | 6.06701E-012 | 3.37030E-012 | .081 | -7.9806E-013 | 1.2932E-011 |
| StePhenyl | BFHDay1 | PFHDay1 | 2.91662E-013 | 1.29405E-012 | .823 | -2.3442E-012 | 2.9276E-012 |
| | | BMHDay1 | 3.06060E-014 | 1.29405E-012 | .981 | -2.6053E-012 | 2.6665E-012 |
| | | PMHDay1 | -1.79961E-012 | 1.29405E-012 | .174 | -4.4355E-012 | 8.3629E-013 |
| | | BFHDay2 | -5.74465E-013 | 1.29405E-012 | .660 | -3.2104E-012 | 2.0614E-012 |
| | | PFHDay2 | -8.20120E-013 | 1.29405E-012 | .531 | -3.4560E-012 | 1.8158E-012 |
| | | BMHDay2 | -1.25855E-012 | 1.29405E-012 | .338 | -3.8944E-012 | 1.3773E-012 |
| | | PMHDay2 | -2.71101E-012* | 1.29405E-012 | .044 | -5.3469E-012 | -7.5120E-014 |
| | PFHDay1 | BFHDay1 | -2.91662E-013 | 1.29405E-012 | .823 | -2.9276E-012 | 2.3442E-012 |
| | | BMHDay1 | -2.61056E-013 | 1.29405E-012 | .841 | -2.8969E-012 | 2.3748E-012 |

| | | | | | | |
|---|---|---|---|---|---|---|
| | PMHDay1 | -2.09127E-012 | 1.29405E-012 | .116 | -4.7272E-012 | 5.4462E-013 |
| | BFHDay2 | -8.66127E-013 | 1.29405E-012 | .508 | -3.5020E-012 | 1.7698E-012 |
| | PFHDay2 | -1.11178E-012 | 1.29405E-012 | .397 | -3.7477E-012 | 1.5241E-012 |
| | BMHDay2 | -1.55021E-012 | 1.29405E-012 | .240 | -4.1861E-012 | 1.0857E-012 |
| | PMHDay2 | -3.00267E-012* | 1.29405E-012 | .027 | -5.6386E-012 | -3.6678E-013 |
| BMHDay1 | BFHDay1 | -3.06060E-014 | 1.29405E-012 | .981 | -2.6665E-012 | 2.6053E-012 |
| | PFHDay1 | 2.61056E-013 | 1.29405E-012 | .841 | -2.3748E-012 | 2.8969E-012 |
| | PMHDay1 | -1.83021E-012 | 1.29405E-012 | .167 | -4.4661E-012 | 8.0568E-013 |
| | BFHDay2 | -6.05071E-013 | 1.29405E-012 | .643 | -3.2410E-012 | 2.0308E-012 |
| | PFHDay2 | -8.50726E-013 | 1.29405E-012 | .516 | -3.4866E-012 | 1.7852E-012 |
| | BMHDay2 | -1.28916E-012 | 1.29405E-012 | .327 | -3.9250E-012 | 1.3467E-012 |
| | PMHDay2 | -2.74162E-012* | 1.29405E-012 | .042 | -5.3775E-012 | -1.0573E-013 |
| PMHDay1 | BFHDay1 | 1.79961E-012 | 1.29405E-012 | .174 | -8.3629E-013 | 4.4355E-012 |
| | PFHDay1 | 2.09127E-012 | 1.29405E-012 | .116 | -5.4462E-013 | 4.7272E-012 |
| | BMHDay1 | 1.83021E-012 | 1.29405E-012 | .167 | -8.0568E-013 | 4.4661E-012 |
| | BFHDay2 | 1.22514E-012 | 1.29405E-012 | .351 | -1.4108E-012 | 3.8610E-012 |
| | PFHDay2 | 9.79486E-013 | 1.29405E-012 | .455 | -1.6564E-012 | 3.6154E-012 |
| | BMHDay2 | 5.41054E-013 | 1.29405E-012 | .679 | -2.0948E-012 | 3.1769E-012 |
| | PMHDay2 | -9.11406E-013 | 1.29405E-012 | .486 | -3.5473E-012 | 1.7245E-012 |

| | | | | | | |
|---|---|---|---|---|---|---|
| BFHDay2 | BFHDay1 | 5.74465E-013 | 1.29405E-012 | .660 | -2.0614E-012 | 3.2104E-012 |
| | PFHDay1 | 8.66127E-013 | 1.29405E-012 | .508 | -1.7698E-012 | 3.5020E-012 |
| | BMHDay1 | 6.05071E-013 | 1.29405E-012 | .643 | -2.0308E-012 | 3.2410E-012 |
| | PMHDay1 | -1.22514E-012 | 1.29405E-012 | .351 | -3.8610E-012 | 1.4108E-012 |
| | PFHDay2 | -2.45655E-013 | 1.29405E-012 | .851 | -2.8815E-012 | 2.3902E-012 |
| | BMHDay2 | -6.84087E-013 | 1.29405E-012 | .601 | -3.3200E-012 | 1.9518E-012 |
| | PMHDay2 | -2.13655E-012 | 1.29405E-012 | .109 | -4.7724E-012 | 4.9934E-013 |
| PFHDay2 | BFHDay1 | 8.20120E-013 | 1.29405E-012 | .531 | -1.8158E-012 | 3.4560E-012 |
| | PFHDay1 | 1.11178E-012 | 1.29405E-012 | .397 | -1.5241E-012 | 3.7477E-012 |
| | BMHDay1 | 8.50726E-013 | 1.29405E-012 | .516 | -1.7852E-012 | 3.4866E-012 |
| | PMHDay1 | -9.79486E-013 | 1.29405E-012 | .455 | -3.6154E-012 | 1.6564E-012 |
| | BFHDay2 | 2.45655E-013 | 1.29405E-012 | .851 | -2.3902E-012 | 2.8815E-012 |
| | BMHDay2 | -4.38432E-013 | 1.29405E-012 | .737 | -3.0743E-012 | 2.1975E-012 |
| | PMHDay2 | -1.89089E-012 | 1.29405E-012 | .154 | -4.5268E-012 | 7.4500E-013 |
| BMHDay2 | BFHDay1 | 1.25855E-012 | 1.29405E-012 | .338 | -1.3773E-012 | 3.8944E-012 |
| | PFHDay1 | 1.55021E-012 | 1.29405E-012 | .240 | -1.0857E-012 | 4.1861E-012 |
| | BMHDay1 | 1.28916E-012 | 1.29405E-012 | .327 | -1.3467E-012 | 3.9250E-012 |
| | PMHDay1 | -5.41054E-013 | 1.29405E-012 | .679 | -3.1769E-012 | 2.0948E-012 |
| | BFHDay2 | 6.84087E-013 | 1.29405E-012 | .601 | -1.9518E-012 | 3.3200E-012 |

| | | | | | | | |
|---|---|---|---|---|---|---|---|
| | | PFHDay2 | 4.38432E-013 | 1.29405E-012 | .737 | -2.1975E-012 | 3.0743E-012 |
| | | PMHDay2 | -1.45246E-012 | 1.29405E-012 | .270 | -4.0884E-012 | 1.1834E-012 |
| | PMHDay2 | BFHDay1 | 2.71101E-012* | 1.29405E-012 | .044 | 7.5120E-014 | 5.3469E-012 |
| | | PFHDay1 | 3.00267E-012* | 1.29405E-012 | .027 | 3.6678E-013 | 5.6386E-012 |
| | | BMHDay1 | 2.74162E-012* | 1.29405E-012 | .042 | 1.0573E-013 | 5.3775E-012 |
| | | PMHDay1 | 9.11406E-013 | 1.29405E-012 | .486 | -1.7245E-012 | 3.5473E-012 |
| | | BFHDay2 | 2.13655E-012 | 1.29405E-012 | .109 | -4.9934E-013 | 4.7724E-012 |
| | | PFHDay2 | 1.89089E-012 | 1.29405E-012 | .154 | -7.4500E-013 | 4.5268E-012 |
| | | BMHDay2 | 1.45246E-012 | 1.29405E-012 | .270 | -1.1834E-012 | 4.0884E-012 |
| OlePhenyl | BFHDay1 | PFHDay1 | 2.56502E-012 | 2.71619E-012 | .352 | -2.9677E-012 | 8.0977E-012 |
| | | BMHDay1 | 3.54760E-012 | 2.71619E-012 | .201 | -1.9851E-012 | 9.0803E-012 |
| | | PMHDay1 | 5.62780E-014 | 2.71619E-012 | .984 | -5.4764E-012 | 5.5890E-012 |
| | | BFHDay2 | 1.00638E-012 | 2.71619E-012 | .713 | -4.5263E-012 | 6.5391E-012 |
| | | PFHDay2 | 5.39434E-013 | 2.71619E-012 | .844 | -4.9933E-012 | 6.0721E-012 |
| | | BMHDay2 | -1.37351E-012 | 2.71619E-012 | .617 | -6.9062E-012 | 4.1592E-012 |
| | | PMHDay2 | -8.02799E-012* | 2.71619E-012 | .006 | -1.3561E-011 | -2.4953E-012 |
| | PFHDay1 | BFHDay1 | -2.56502E-012 | 2.71619E-012 | .352 | -8.0977E-012 | 2.9677E-012 |
| | | BMHDay1 | 9.82574E-013 | 2.71619E-012 | .720 | -4.5501E-012 | 6.5153E-012 |
| | | PMHDay1 | -2.50874E-012 | 2.71619E-012 | .363 | -8.0414E-012 | 3.0240E-012 |

| | | | | | | | |
|---|---|---|---|---|---|---|---|
| | | BFHDay2 | -1.55864E-012 | 2.71619E-012 | .570 | -7.0913E-012 | 3.9741E-012 |
| | | PFHDay2 | -2.02559E-012 | 2.71619E-012 | .461 | -7.5583E-012 | 3.5071E-012 |
| | | BMHDay2 | -3.93854E-012 | 2.71619E-012 | .157 | -9.4712E-012 | 1.5942E-012 |
| | | PMHDay2 | -1.05930E-011* | 2.71619E-012 | <.001 | -1.6126E-011 | -5.0603E-012 |
| | BMHDay1 | BFHDay1 | -3.54760E-012 | 2.71619E-012 | .201 | -9.0803E-012 | 1.9851E-012 |
| | | PFHDay1 | -9.82574E-013 | 2.71619E-012 | .720 | -6.5153E-012 | 4.5501E-012 |
| | | PMHDay1 | -3.49132E-012 | 2.71619E-012 | .208 | -9.0240E-012 | 2.0414E-012 |
| | | BFHDay2 | -2.54121E-012 | 2.71619E-012 | .356 | -8.0739E-012 | 2.9915E-012 |
| | | PFHDay2 | -3.00816E-012 | 2.71619E-012 | .276 | -8.5409E-012 | 2.5245E-012 |
| | | BMHDay2 | -4.92111E-012 | 2.71619E-012 | .079 | -1.0454E-011 | 6.1159E-013 |
| | | PMHDay2 | -1.15756E-011* | 2.71619E-012 | <.001 | -1.7108E-011 | -6.0429E-012 |
| | PMHDay1 | BFHDay1 | -5.62780E-014 | 2.71619E-012 | .984 | -5.5890E-012 | 5.4764E-012 |
| | | PFHDay1 | 2.50874E-012 | 2.71619E-012 | .363 | -3.0240E-012 | 8.0414E-012 |
| | | BMHDay1 | 3.49132E-012 | 2.71619E-012 | .208 | -2.0414E-012 | 9.0240E-012 |
| | | BFHDay2 | 9.50106E-013 | 2.71619E-012 | .729 | -4.5826E-012 | 6.4828E-012 |
| | | PFHDay2 | 4.83156E-013 | 2.71619E-012 | .860 | -5.0495E-012 | 6.0159E-012 |
| | | BMHDay2 | -1.42979E-012 | 2.71619E-012 | .602 | -6.9625E-012 | 4.1029E-012 |
| | | PMHDay2 | -8.08427E-012* | 2.71619E-012 | .006 | -1.3617E-011 | -2.5516E-012 |
| | BFHDay2 | BFHDay1 | -1.00638E-012 | 2.71619E-012 | .713 | -6.5391E-012 | 4.5263E-012 |

| | | | | | | | |
|---|---|---|---|---|---|---|---|
| | | PFHDay1 | 1.55864E-012 | 2.71619E-012 | .570 | -3.9741E-012 | 7.0913E-012 |
| | | BMHDay1 | 2.54121E-012 | 2.71619E-012 | .356 | -2.9915E-012 | 8.0739E-012 |
| | | PMHDay1 | -9.50106E-013 | 2.71619E-012 | .729 | -6.4828E-012 | 4.5826E-012 |
| | | PFHDay2 | -4.66950E-013 | 2.71619E-012 | .865 | -5.9997E-012 | 5.0658E-012 |
| | | BMHDay2 | -2.37990E-012 | 2.71619E-012 | .387 | -7.9126E-012 | 3.1528E-012 |
| | | PMHDay2 | -9.03437E-012* | 2.71619E-012 | .002 | -1.4567E-011 | -3.5017E-012 |
| | PFHDay2 | BFHDay1 | -5.39434E-013 | 2.71619E-012 | .844 | -6.0721E-012 | 4.9933E-012 |
| | | PFHDay1 | 2.02559E-012 | 2.71619E-012 | .461 | -3.5071E-012 | 7.5583E-012 |
| | | BMHDay1 | 3.00816E-012 | 2.71619E-012 | .276 | -2.5245E-012 | 8.5409E-012 |
| | | PMHDay1 | -4.83156E-013 | 2.71619E-012 | .860 | -6.0159E-012 | 5.0495E-012 |
| | | BFHDay2 | 4.66950E-013 | 2.71619E-012 | .865 | -5.0658E-012 | 5.9997E-012 |
| | | BMHDay2 | -1.91295E-012 | 2.71619E-012 | .486 | -7.4457E-012 | 3.6198E-012 |
| | | PMHDay2 | -8.56742E-012* | 2.71619E-012 | .003 | -1.4100E-011 | -3.0347E-012 |
| | BMHDay2 | BFHDay1 | 1.37351E-012 | 2.71619E-012 | .617 | -4.1592E-012 | 6.9062E-012 |
| | | PFHDay1 | 3.93854E-012 | 2.71619E-012 | .157 | -1.5942E-012 | 9.4712E-012 |
| | | BMHDay1 | 4.92111E-012 | 2.71619E-012 | .079 | -6.1159E-013 | 1.0454E-011 |
| | | PMHDay1 | 1.42979E-012 | 2.71619E-012 | .602 | -4.1029E-012 | 6.9625E-012 |
| | | BFHDay2 | 2.37990E-012 | 2.71619E-012 | .387 | -3.1528E-012 | 7.9126E-012 |
| | | PFHDay2 | 1.91295E-012 | 2.71619E-012 | .486 | -3.6198E-012 | 7.4457E-012 |

| | | | | | | | |
|---|---|---|---|---|---|---|---|
| | | PMHDay2 | -6.65448E-012* | 2.71619E-012 | .020 | -1.2187E-011 | -1.1218E-012 |
| | PMHDay2 | BFHDay1 | 8.02799E-012* | 2.71619E-012 | .006 | 2.4953E-012 | 1.3561E-011 |
| | | PFHDay1 | 1.05930E-011* | 2.71619E-012 | <.001 | 5.0603E-012 | 1.6126E-011 |
| | | BMHDay1 | 1.15756E-011* | 2.71619E-012 | <.001 | 6.0429E-012 | 1.7108E-011 |
| | | PMHDay1 | 8.08427E-012* | 2.71619E-012 | .006 | 2.5516E-012 | 1.3617E-011 |
| | | BFHDay2 | 9.03437E-012* | 2.71619E-012 | .002 | 3.5017E-012 | 1.4567E-011 |
| | | PFHDay2 | 8.56742E-012* | 2.71619E-012 | .003 | 3.0347E-012 | 1.4100E-011 |
| | | BMHDay2 | 6.65448E-012* | 2.71619E-012 | .020 | 1.1218E-012 | 1.2187E-011 |
| LinPhenyl | BFHDay1 | PFHDay1 | 2.12462E-012 | 2.85424E-012 | .462 | -3.6893E-012 | 7.9385E-012 |
| | | BMHDay1 | 2.64014E-012 | 2.85424E-012 | .362 | -3.1738E-012 | 8.4540E-012 |
| | | PMHDay1 | -3.10159E-012 | 2.85424E-012 | .285 | -8.9155E-012 | 2.7123E-012 |
| | | BFHDay2 | -1.23028E-013 | 2.85424E-012 | .966 | -5.9369E-012 | 5.6909E-012 |
| | | PFHDay2 | 1.12420E-013 | 2.85424E-012 | .969 | -5.7015E-012 | 5.9263E-012 |
| | | BMHDay2 | -6.70612E-012* | 2.85424E-012 | .025 | -1.2520E-011 | -8.9221E-013 |
| | | PMHDay2 | -1.03446E-011* | 2.85424E-012 | <.001 | -1.6159E-011 | -4.5307E-012 |
| | PFHDay1 | BFHDay1 | -2.12462E-012 | 2.85424E-012 | .462 | -7.9385E-012 | 3.6893E-012 |
| | | BMHDay1 | 5.15518E-013 | 2.85424E-012 | .858 | -5.2984E-012 | 6.3294E-012 |
| | | PMHDay1 | -5.22621E-012 | 2.85424E-012 | .076 | -1.1040E-011 | 5.8769E-013 |
| | | BFHDay2 | -2.24765E-012 | 2.85424E-012 | .437 | -8.0616E-012 | 3.5663E-012 |

| | | | | | | |
|---|---|---|---|---|---|---|
| | PFHDay2 | -2.01220E-012 | 2.85424E-012 | .486 | -7.8261E-012 | 3.8017E-012 |
| | BMHDay2 | -8.83074E-012* | 2.85424E-012 | .004 | -1.4645E-011 | -3.0168E-012 |
| | PMHDay2 | -1.24692E-011* | 2.85424E-012 | <.001 | -1.8283E-011 | -6.6553E-012 |
| BMHDay1 | BFHDay1 | -2.64014E-012 | 2.85424E-012 | .362 | -8.4540E-012 | 3.1738E-012 |
| | PFHDay1 | -5.15518E-013 | 2.85424E-012 | .858 | -6.3294E-012 | 5.2984E-012 |
| | PMHDay1 | -5.74173E-012 | 2.85424E-012 | .053 | -1.1556E-011 | 7.2176E-014 |
| | BFHDay2 | -2.76317E-012 | 2.85424E-012 | .340 | -8.5771E-012 | 3.0507E-012 |
| | PFHDay2 | -2.52772E-012 | 2.85424E-012 | .382 | -8.3416E-012 | 3.2862E-012 |
| | BMHDay2 | -9.34625E-012* | 2.85424E-012 | .003 | -1.5160E-011 | -3.5323E-012 |
| | PMHDay2 | -1.29848E-011* | 2.85424E-012 | <.001 | -1.8799E-011 | -7.1709E-012 |
| PMHDay1 | BFHDay1 | 3.10159E-012 | 2.85424E-012 | .285 | -2.7123E-012 | 8.9155E-012 |
| | PFHDay1 | 5.22621E-012 | 2.85424E-012 | .076 | -5.8769E-013 | 1.1040E-011 |
| | BMHDay1 | 5.74173E-012 | 2.85424E-012 | .053 | -7.2176E-014 | 1.1556E-011 |
| | BFHDay2 | 2.97856E-012 | 2.85424E-012 | .305 | -2.8353E-012 | 8.7925E-012 |
| | PFHDay2 | 3.21401E-012 | 2.85424E-012 | .269 | -2.5999E-012 | 9.0279E-012 |
| | BMHDay2 | -3.60452E-012 | 2.85424E-012 | .216 | -9.4184E-012 | 2.2094E-012 |
| | PMHDay2 | -7.24303E-012* | 2.85424E-012 | .016 | -1.3057E-011 | -1.4291E-012 |
| BFHDay2 | BFHDay1 | 1.23028E-013 | 2.85424E-012 | .966 | -5.6909E-012 | 5.9369E-012 |
| | PFHDay1 | 2.24765E-012 | 2.85424E-012 | .437 | -3.5663E-012 | 8.0616E-012 |

| | | | | | | |
|---|---|---|---|---|---|---|
| | BMHDay1 | 2.76317E-012 | 2.85424E-012 | .340 | -3.0507E-012 | 8.5771E-012 |
| | PMHDay1 | -2.97856E-012 | 2.85424E-012 | .305 | -8.7925E-012 | 2.8353E-012 |
| | PFHDay2 | 2.35448E-013 | 2.85424E-012 | .935 | -5.5785E-012 | 6.0494E-012 |
| | BMHDay2 | -6.58309E-012* | 2.85424E-012 | .028 | -1.2397E-011 | -7.6918E-013 |
| | PMHDay2 | -1.02216E-011* | 2.85424E-012 | .001 | -1.6035E-011 | -4.4077E-012 |
| PFHDay2 | BFHDay1 | -1.12420E-013 | 2.85424E-012 | .969 | -5.9263E-012 | 5.7015E-012 |
| | PFHDay1 | 2.01220E-012 | 2.85424E-012 | .486 | -3.8017E-012 | 7.8261E-012 |
| | BMHDay1 | 2.52772E-012 | 2.85424E-012 | .382 | -3.2862E-012 | 8.3416E-012 |
| | PMHDay1 | -3.21401E-012 | 2.85424E-012 | .269 | -9.0279E-012 | 2.5999E-012 |
| | BFHDay2 | -2.35448E-013 | 2.85424E-012 | .935 | -6.0494E-012 | 5.5785E-012 |
| | BMHDay2 | -6.81854E-012* | 2.85424E-012 | .023 | -1.2632E-011 | -1.0046E-012 |
| | PMHDay2 | -1.04570E-011* | 2.85424E-012 | <.001 | -1.6271E-011 | -4.6431E-012 |
| BMHDay2 | BFHDay1 | 6.70612E-012* | 2.85424E-012 | .025 | 8.9221E-013 | 1.2520E-011 |
| | PFHDay1 | 8.83074E-012* | 2.85424E-012 | .004 | 3.0168E-012 | 1.4645E-011 |
| | BMHDay1 | 9.34625E-012* | 2.85424E-012 | .003 | 3.5323E-012 | 1.5160E-011 |
| | PMHDay1 | 3.60452E-012 | 2.85424E-012 | .216 | -2.2094E-012 | 9.4184E-012 |
| | BFHDay2 | 6.58309E-012* | 2.85424E-012 | .028 | 7.6918E-013 | 1.2397E-011 |
| | PFHDay2 | 6.81854E-012* | 2.85424E-012 | .023 | 1.0046E-012 | 1.2632E-011 |
| | PMHDay2 | -3.63851E-012 | 2.85424E-012 | .212 | -9.4524E-012 | 2.1754E-012 |

| | | | | | | | |
|---|---|---|---|---|---|---|---|
| | PMHDay2 | BFHDay1 | 1.03446E-011* | 2.85424E-012 | <.001 | 4.5307E-012 | 1.6159E-011 |
| | | PFHDay1 | 1.24692E-011* | 2.85424E-012 | <.001 | 6.6553E-012 | 1.8283E-011 |
| | | BMHDay1 | 1.29848E-011* | 2.85424E-012 | <.001 | 7.1709E-012 | 1.8799E-011 |
| | | PMHDay1 | 7.24303E-012* | 2.85424E-012 | .016 | 1.4291E-012 | 1.3057E-011 |
| | | BFHDay2 | 1.02216E-011* | 2.85424E-012 | .001 | 4.4077E-012 | 1.6035E-011 |
| | | PFHDay2 | 1.04570E-011* | 2.85424E-012 | <.001 | 4.6431E-012 | 1.6271E-011 |
| | | BMHDay2 | 3.63851E-012 | 2.85424E-012 | .212 | -2.1754E-012 | 9.4524E-012 |
| AraPhenyl | BFHDay1 | PFHDay1 | 1.32116E-012 | 8.16007E-013 | .115 | -3.4099E-013 | 2.9833E-012 |
| | | BMHDay1 | 1.35903E-012 | 8.16007E-013 | .106 | -3.0312E-013 | 3.0212E-012 |
| | | PMHDay1 | 1.20499E-012 | 8.16007E-013 | .150 | -4.5717E-013 | 2.8671E-012 |
| | | BFHDay2 | 1.37361E-012 | 8.16007E-013 | .102 | -2.8855E-013 | 3.0358E-012 |
| | | PFHDay2 | 1.08130E-012 | 8.16007E-013 | .195 | -5.8085E-013 | 2.7434E-012 |
| | | BMHDay2 | 7.40055E-013 | 8.16007E-013 | .371 | -9.2210E-013 | 2.4022E-012 |
| | | PMHDay2 | 4.64046E-013 | 8.16007E-013 | .574 | -1.1981E-012 | 2.1262E-012 |
| | PFHDay1 | BFHDay1 | -1.32116E-012 | 8.16007E-013 | .115 | -2.9833E-012 | 3.4099E-013 |
| | | BMHDay1 | 3.78686E-014 | 8.16007E-013 | .963 | -1.6243E-012 | 1.7000E-012 |
| | | PMHDay1 | -1.16178E-013 | 8.16007E-013 | .888 | -1.7783E-012 | 1.5460E-012 |
| | | BFHDay2 | 5.24426E-014 | 8.16007E-013 | .949 | -1.6097E-012 | 1.7146E-012 |
| | | PFHDay2 | -2.39866E-013 | 8.16007E-013 | .771 | -1.9020E-012 | 1.4223E-012 |

| | | | | | | |
|---|---|---|---|---|---|---|
| | BMHDay2 | -5.81108E-013 | 8.16007E-013 | .482 | -2.2433E-012 | 1.0810E-012 |
| | PMHDay2 | -8.57117E-013 | 8.16007E-013 | .301 | -2.5193E-012 | 8.0503E-013 |
| BMHDay1 | BFHDay1 | -1.35903E-012 | 8.16007E-013 | .106 | -3.0212E-012 | 3.0312E-013 |
| | PFHDay1 | -3.78686E-014 | 8.16007E-013 | .963 | -1.7000E-012 | 1.6243E-012 |
| | PMHDay1 | -1.54046E-013 | 8.16007E-013 | .851 | -1.8162E-012 | 1.5081E-012 |
| | BFHDay2 | 1.45740E-014 | 8.16007E-013 | .986 | -1.6476E-012 | 1.6767E-012 |
| | PFHDay2 | -2.77734E-013 | 8.16007E-013 | .736 | -1.9399E-012 | 1.3844E-012 |
| | BMHDay2 | -6.18977E-013 | 8.16007E-013 | .454 | -2.2811E-012 | 1.0432E-012 |
| | PMHDay2 | -8.94986E-013 | 8.16007E-013 | .281 | -2.5571E-012 | 7.6717E-013 |
| PMHDay1 | BFHDay1 | -1.20499E-012 | 8.16007E-013 | .150 | -2.8671E-012 | 4.5717E-013 |
| | PFHDay1 | 1.16178E-013 | 8.16007E-013 | .888 | -1.5460E-012 | 1.7783E-012 |
| | BMHDay1 | 1.54046E-013 | 8.16007E-013 | .851 | -1.5081E-012 | 1.8162E-012 |
| | BFHDay2 | 1.68620E-013 | 8.16007E-013 | .838 | -1.4935E-012 | 1.8308E-012 |
| | PFHDay2 | -1.23688E-013 | 8.16007E-013 | .880 | -1.7858E-012 | 1.5385E-012 |
| | BMHDay2 | -4.64930E-013 | 8.16007E-013 | .573 | -2.1271E-012 | 1.1972E-012 |
| | PMHDay2 | -7.40940E-013 | 8.16007E-013 | .371 | -2.4031E-012 | 9.2121E-013 |
| BFHDay2 | BFHDay1 | -1.37361E-012 | 8.16007E-013 | .102 | -3.0358E-012 | 2.8855E-013 |
| | PFHDay1 | -5.24426E-014 | 8.16007E-013 | .949 | -1.7146E-012 | 1.6097E-012 |
| | BMHDay1 | -1.45740E-014 | 8.16007E-013 | .986 | -1.6767E-012 | 1.6476E-012 |

| | | | | | | |
|---|---|---|---|---|---|---|
| | PMHDay1 | -1.68620E-013 | 8.16007E-013 | .838 | -1.8308E-012 | 1.4935E-012 |
| | PFHDay2 | -2.92308E-013 | 8.16007E-013 | .723 | -1.9545E-012 | 1.3698E-012 |
| | BMHDay2 | -6.33551E-013 | 8.16007E-013 | .443 | -2.2957E-012 | 1.0286E-012 |
| | PMHDay2 | -9.09560E-013 | 8.16007E-013 | .273 | -2.5717E-012 | 7.5259E-013 |
| PFHDay2 | BFHDay1 | -1.08130E-012 | 8.16007E-013 | .195 | -2.7434E-012 | 5.8085E-013 |
| | PFHDay1 | 2.39866E-013 | 8.16007E-013 | .771 | -1.4223E-012 | 1.9020E-012 |
| | BMHDay1 | 2.77734E-013 | 8.16007E-013 | .736 | -1.3844E-012 | 1.9399E-012 |
| | PMHDay1 | 1.23688E-013 | 8.16007E-013 | .880 | -1.5385E-012 | 1.7858E-012 |
| | BFHDay2 | 2.92308E-013 | 8.16007E-013 | .723 | -1.3698E-012 | 1.9545E-012 |
| | BMHDay2 | -3.41242E-013 | 8.16007E-013 | .679 | -2.0034E-012 | 1.3209E-012 |
| | PMHDay2 | -6.17252E-013 | 8.16007E-013 | .455 | -2.2794E-012 | 1.0449E-012 |
| BMHDay2 | BFHDay1 | -7.40055E-013 | 8.16007E-013 | .371 | -2.4022E-012 | 9.2210E-013 |
| | PFHDay1 | 5.81108E-013 | 8.16007E-013 | .482 | -1.0810E-012 | 2.2433E-012 |
| | BMHDay1 | 6.18977E-013 | 8.16007E-013 | .454 | -1.0432E-012 | 2.2811E-012 |
| | PMHDay1 | 4.64930E-013 | 8.16007E-013 | .573 | -1.1972E-012 | 2.1271E-012 |
| | BFHDay2 | 6.33551E-013 | 8.16007E-013 | .443 | -1.0286E-012 | 2.2957E-012 |
| | PFHDay2 | 3.41242E-013 | 8.16007E-013 | .679 | -1.3209E-012 | 2.0034E-012 |
| | PMHDay2 | -2.76009E-013 | 8.16007E-013 | .737 | -1.9382E-012 | 1.3861E-012 |
| PMHDay2 | BFHDay1 | -4.64046E-013 | 8.16007E-013 | .574 | -2.1262E-012 | 1.1981E-012 |

| | | | | | | | |
|---|---|---|---|---|---|---|---|
| | | PFHDay1 | 8.57117E-013 | 8.16007E-013 | .301 | -8.0503E-013 | 2.5193E-012 |
| | | BMHDay1 | 8.94986E-013 | 8.16007E-013 | .281 | -7.6717E-013 | 2.5571E-012 |
| | | PMHDay1 | 7.40940E-013 | 8.16007E-013 | .371 | -9.2121E-013 | 2.4031E-012 |
| | | BFHDay2 | 9.09560E-013 | 8.16007E-013 | .273 | -7.5259E-013 | 2.5717E-012 |
| | | PFHDay2 | 6.17252E-013 | 8.16007E-013 | .455 | -1.0449E-012 | 2.2794E-012 |
| | | BMHDay2 | 2.76009E-013 | 8.16007E-013 | .737 | -1.3861E-012 | 1.9382E-012 |
| DocPhenyl | BFHDay1 | PFHDay1 | 1.61569E-012 | 9.92140E-013 | .113 | -4.0523E-013 | 3.6366E-012 |
| | | BMHDay1 | 2.52942E-012* | 9.92140E-013 | .016 | 5.0849E-013 | 4.5503E-012 |
| | | PMHDay1 | -2.55370E-013 | 9.92140E-013 | .799 | -2.2763E-012 | 1.7656E-012 |
| | | BFHDay2 | 1.18567E-012 | 9.92140E-013 | .241 | -8.3525E-013 | 3.2066E-012 |
| | | PFHDay2 | 1.61948E-012 | 9.92140E-013 | .112 | -4.0145E-013 | 3.6404E-012 |
| | | BMHDay2 | 2.02294E-012* | 9.92140E-013 | .050 | 2.0179E-015 | 4.0439E-012 |
| | | PMHDay2 | 1.88383E-012 | 9.92140E-013 | .067 | -1.3709E-013 | 3.9048E-012 |
| | PFHDay1 | BFHDay1 | -1.61569E-012 | 9.92140E-013 | .113 | -3.6366E-012 | 4.0523E-013 |
| | | BMHDay1 | 9.13728E-013 | 9.92140E-013 | .364 | -1.1072E-012 | 2.9347E-012 |
| | | PMHDay1 | -1.87106E-012 | 9.92140E-013 | .068 | -3.8920E-012 | 1.4986E-013 |
| | | BFHDay2 | -4.30020E-013 | 9.92140E-013 | .668 | -2.4509E-012 | 1.5909E-012 |
| | | PFHDay2 | 3.78800E-015 | 9.92140E-013 | .997 | -2.0171E-012 | 2.0247E-012 |
| | | BMHDay2 | 4.07252E-013 | 9.92140E-013 | .684 | -1.6137E-012 | 2.4282E-012 |

| | | | | | | |
|---|---|---|---|---|---|---|
| | | PMHDay2 | 2.68142E-013 | 9.92140E-013 | .789 | -1.7528E-012 | 2.2891E-012 |
| | BMHDay1 | BFHDay1 | -2.52942E-012* | 9.92140E-013 | .016 | -4.5503E-012 | -5.0849E-013 |
| | | PFHDay1 | -9.13728E-013 | 9.92140E-013 | .364 | -2.9347E-012 | 1.1072E-012 |
| | | PMHDay1 | -2.78479E-012* | 9.92140E-013 | .008 | -4.8057E-012 | -7.6386E-013 |
| | | BFHDay2 | -1.34375E-012 | 9.92140E-013 | .185 | -3.3647E-012 | 6.7717E-013 |
| | | PFHDay2 | -9.09940E-013 | 9.92140E-013 | .366 | -2.9309E-012 | 1.1110E-012 |
| | | BMHDay2 | -5.06476E-013 | 9.92140E-013 | .613 | -2.5274E-012 | 1.5144E-012 |
| | | PMHDay2 | -6.45586E-013 | 9.92140E-013 | .520 | -2.6665E-012 | 1.3753E-012 |
| | PMHDay1 | BFHDay1 | 2.55370E-013 | 9.92140E-013 | .799 | -1.7656E-012 | 2.2763E-012 |
| | | PFHDay1 | 1.87106E-012 | 9.92140E-013 | .068 | -1.4986E-013 | 3.8920E-012 |
| | | BMHDay1 | 2.78479E-012* | 9.92140E-013 | .008 | 7.6386E-013 | 4.8057E-012 |
| | | BFHDay2 | 1.44104E-012 | 9.92140E-013 | .156 | -5.7988E-013 | 3.4620E-012 |
| | | PFHDay2 | 1.87485E-012 | 9.92140E-013 | .068 | -1.4608E-013 | 3.8958E-012 |
| | | BMHDay2 | 2.27831E-012* | 9.92140E-013 | .028 | 2.5739E-013 | 4.2992E-012 |
| | | PMHDay2 | 2.13920E-012* | 9.92140E-013 | .039 | 1.1828E-013 | 4.1601E-012 |
| | BFHDay2 | BFHDay1 | -1.18567E-012 | 9.92140E-013 | .241 | -3.2066E-012 | 8.3525E-013 |
| | | PFHDay1 | 4.30020E-013 | 9.92140E-013 | .668 | -1.5909E-012 | 2.4509E-012 |
| | | BMHDay1 | 1.34375E-012 | 9.92140E-013 | .185 | -6.7717E-013 | 3.3647E-012 |
| | | PMHDay1 | -1.44104E-012 | 9.92140E-013 | .156 | -3.4620E-012 | 5.7988E-013 |

| | | | | | | |
|---|---|---|---|---|---|---|
| | PFHDay2 | 4.33808E-013 | 9.92140E-013 | .665 | -1.5871E-012 | 2.4547E-012 |
| | BMHDay2 | 8.37272E-013 | 9.92140E-013 | .405 | -1.1836E-012 | 2.8582E-012 |
| | PMHDay2 | 6.98162E-013 | 9.92140E-013 | .487 | -1.3228E-012 | 2.7191E-012 |
| PFHDay2 | BFHDay1 | -1.61948E-012 | 9.92140E-013 | .112 | -3.6404E-012 | 4.0145E-013 |
| | PFHDay1 | -3.78800E-015 | 9.92140E-013 | .997 | -2.0247E-012 | 2.0171E-012 |
| | BMHDay1 | 9.09940E-013 | 9.92140E-013 | .366 | -1.1110E-012 | 2.9309E-012 |
| | PMHDay1 | -1.87485E-012 | 9.92140E-013 | .068 | -3.8958E-012 | 1.4608E-013 |
| | BFHDay2 | -4.33808E-013 | 9.92140E-013 | .665 | -2.4547E-012 | 1.5871E-012 |
| | BMHDay2 | 4.03464E-013 | 9.92140E-013 | .687 | -1.6175E-012 | 2.4244E-012 |
| | PMHDay2 | 2.64354E-013 | 9.92140E-013 | .792 | -1.7566E-012 | 2.2853E-012 |
| BMHDay2 | BFHDay1 | -2.02294E-012* | 9.92140E-013 | .050 | -4.0439E-012 | -2.0179E-015 |
| | PFHDay1 | -4.07252E-013 | 9.92140E-013 | .684 | -2.4282E-012 | 1.6137E-012 |
| | BMHDay1 | 5.06476E-013 | 9.92140E-013 | .613 | -1.5144E-012 | 2.5274E-012 |
| | PMHDay1 | -2.27831E-012* | 9.92140E-013 | .028 | -4.2992E-012 | -2.5739E-013 |
| | BFHDay2 | -8.37272E-013 | 9.92140E-013 | .405 | -2.8582E-012 | 1.1836E-012 |
| | PFHDay2 | -4.03464E-013 | 9.92140E-013 | .687 | -2.4244E-012 | 1.6175E-012 |
| | PMHDay2 | -1.39110E-013 | 9.92140E-013 | .889 | -2.1600E-012 | 1.8818E-012 |
| PMHDay2 | BFHDay1 | -1.88383E-012 | 9.92140E-013 | .067 | -3.9048E-012 | 1.3709E-013 |
| | PFHDay1 | -2.68142E-013 | 9.92140E-013 | .789 | -2.2891E-012 | 1.7528E-012 |

| | | | | | | | |
|---|---|---|---|---|---|---|---|
| | | BMHDay1 | 6.45586E-013 | 9.92140E-013 | .520 | -1.3753E-012 | 2.6665E-012 |
| | | PMHDay1 | -2.13920E-012* | 9.92140E-013 | .039 | -4.1601E-012 | -1.1828E-013 |
| | | BFHDay2 | -6.98162E-013 | 9.92140E-013 | .487 | -2.7191E-012 | 1.3228E-012 |
| | | PFHDay2 | -2.64354E-013 | 9.92140E-013 | .792 | -2.2853E-012 | 1.7566E-012 |
| | | BMHDay2 | 1.39110E-013 | 9.92140E-013 | .889 | -1.8818E-012 | 2.1600E-012 |
| palAla | BFHDay1 | PFHDay1 | 4.32233E-012 | 3.48917E-012 | .224 | -2.7849E-012 | 1.1430E-011 |
| | | BMHDay1 | 7.70205E-012* | 3.48917E-012 | .035 | 5.9484E-013 | 1.4809E-011 |
| | | PMHDay1 | 6.83760E-012 | 3.48917E-012 | .059 | -2.6961E-013 | 1.3945E-011 |
| | | BFHDay2 | 1.35286E-012 | 3.48917E-012 | .701 | -5.7544E-012 | 8.4601E-012 |
| | | PFHDay2 | 6.70573E-012 | 3.48917E-012 | .064 | -4.0148E-013 | 1.3813E-011 |
| | | BMHDay2 | 2.17336E-012 | 3.48917E-012 | .538 | -4.9339E-012 | 9.2806E-012 |
| | | PMHDay2 | 2.88645E-012 | 3.48917E-012 | .414 | -4.2208E-012 | 9.9937E-012 |
| | PFHDay1 | BFHDay1 | -4.32233E-012 | 3.48917E-012 | .224 | -1.1430E-011 | 2.7849E-012 |
| | | BMHDay1 | 3.37973E-012 | 3.48917E-012 | .340 | -3.7275E-012 | 1.0487E-011 |
| | | PMHDay1 | 2.51527E-012 | 3.48917E-012 | .476 | -4.5919E-012 | 9.6225E-012 |
| | | BFHDay2 | -2.96947E-012 | 3.48917E-012 | .401 | -1.0077E-011 | 4.1377E-012 |
| | | PFHDay2 | 2.38341E-012 | 3.48917E-012 | .499 | -4.7238E-012 | 9.4906E-012 |
| | | BMHDay2 | -2.14897E-012 | 3.48917E-012 | .542 | -9.2562E-012 | 4.9582E-012 |
| | | PMHDay2 | -1.43588E-012 | 3.48917E-012 | .683 | -8.5431E-012 | 5.6713E-012 |

| | | | | | | |
|---|---|---|---|---|---|---|
| | BMHDay1 | BFHDay1 | -7.70205E-012* | 3.48917E-012 | .035 | -1.4809E-011 | -5.9484E-013 |
| | | PFHDay1 | -3.37973E-012 | 3.48917E-012 | .340 | -1.0487E-011 | 3.7275E-012 |
| | | PMHDay1 | -8.64452E-013 | 3.48917E-012 | .806 | -7.9717E-012 | 6.2428E-012 |
| | | BFHDay2 | -6.34919E-012 | 3.48917E-012 | .078 | -1.3456E-011 | 7.5802E-013 |
| | | PFHDay2 | -9.96318E-013 | 3.48917E-012 | .777 | -8.1035E-012 | 6.1109E-012 |
| | | BMHDay2 | -5.52869E-012 | 3.48917E-012 | .123 | -1.2636E-011 | 1.5785E-012 |
| | | PMHDay2 | -4.81561E-012 | 3.48917E-012 | .177 | -1.1923E-011 | 2.2916E-012 |
| | PMHDay1 | BFHDay1 | -6.83760E-012 | 3.48917E-012 | .059 | -1.3945E-011 | 2.6961E-013 |
| | | PFHDay1 | -2.51527E-012 | 3.48917E-012 | .476 | -9.6225E-012 | 4.5919E-012 |
| | | BMHDay1 | 8.64452E-013 | 3.48917E-012 | .806 | -6.2428E-012 | 7.9717E-012 |
| | | BFHDay2 | -5.48474E-012 | 3.48917E-012 | .126 | -1.2592E-011 | 1.6225E-012 |
| | | PFHDay2 | -1.31866E-013 | 3.48917E-012 | .970 | -7.2391E-012 | 6.9753E-012 |
| | | BMHDay2 | -4.66424E-012 | 3.48917E-012 | .191 | -1.1771E-011 | 2.4430E-012 |
| | | PMHDay2 | -3.95115E-012 | 3.48917E-012 | .266 | -1.1058E-011 | 3.1561E-012 |
| | BFHDay2 | BFHDay1 | -1.35286E-012 | 3.48917E-012 | .701 | -8.4601E-012 | 5.7544E-012 |
| | | PFHDay1 | 2.96947E-012 | 3.48917E-012 | .401 | -4.1377E-012 | 1.0077E-011 |
| | | BMHDay1 | 6.34919E-012 | 3.48917E-012 | .078 | -7.5802E-013 | 1.3456E-011 |
| | | PMHDay1 | 5.48474E-012 | 3.48917E-012 | .126 | -1.6225E-012 | 1.2592E-011 |
| | | PFHDay2 | 5.35287E-012 | 3.48917E-012 | .135 | -1.7543E-012 | 1.2460E-011 |

| | | | | | | |
|---|---|---|---|---|---|---|
| | BMHDay2 | 8.20500E-013 | 3.48917E-012 | .816 | -6.2867E-012 | 7.9277E-012 |
| | PMHDay2 | 1.53359E-012 | 3.48917E-012 | .663 | -5.5736E-012 | 8.6408E-012 |
| PFHDay2 | BFHDay1 | -6.70573E-012 | 3.48917E-012 | .064 | -1.3813E-011 | 4.0148E-013 |
| | PFHDay1 | -2.38341E-012 | 3.48917E-012 | .499 | -9.4906E-012 | 4.7238E-012 |
| | BMHDay1 | 9.96318E-013 | 3.48917E-012 | .777 | -6.1109E-012 | 8.1035E-012 |
| | PMHDay1 | 1.31866E-013 | 3.48917E-012 | .970 | -6.9753E-012 | 7.2391E-012 |
| | BFHDay2 | -5.35287E-012 | 3.48917E-012 | .135 | -1.2460E-011 | 1.7543E-012 |
| | BMHDay2 | -4.53237E-012 | 3.48917E-012 | .203 | -1.1640E-011 | 2.5748E-012 |
| | PMHDay2 | -3.81929E-012 | 3.48917E-012 | .282 | -1.0926E-011 | 3.2879E-012 |
| BMHDay2 | BFHDay1 | -2.17336E-012 | 3.48917E-012 | .538 | -9.2806E-012 | 4.9339E-012 |
| | PFHDay1 | 2.14897E-012 | 3.48917E-012 | .542 | -4.9582E-012 | 9.2562E-012 |
| | BMHDay1 | 5.52869E-012 | 3.48917E-012 | .123 | -1.5785E-012 | 1.2636E-011 |
| | PMHDay1 | 4.66424E-012 | 3.48917E-012 | .191 | -2.4430E-012 | 1.1771E-011 |
| | BFHDay2 | -8.20500E-013 | 3.48917E-012 | .816 | -7.9277E-012 | 6.2867E-012 |
| | PFHDay2 | 4.53237E-012 | 3.48917E-012 | .203 | -2.5748E-012 | 1.1640E-011 |
| | PMHDay2 | 7.13086E-013 | 3.48917E-012 | .839 | -6.3941E-012 | 7.8203E-012 |
| PMHDay2 | BFHDay1 | -2.88645E-012 | 3.48917E-012 | .414 | -9.9937E-012 | 4.2208E-012 |
| | PFHDay1 | 1.43588E-012 | 3.48917E-012 | .683 | -5.6713E-012 | 8.5431E-012 |
| | BMHDay1 | 4.81561E-012 | 3.48917E-012 | .177 | -2.2916E-012 | 1.1923E-011 |

| | | | | | | | |
|---|---|---|---|---|---|---|---|
| | | PMHDay1 | 3.95115E-012 | 3.48917E-012 | .266 | -3.1561E-012 | 1.1058E-011 |
| | | BFHDay2 | -1.53359E-012 | 3.48917E-012 | .663 | -8.6408E-012 | 5.5736E-012 |
| | | PFHDay2 | 3.81929E-012 | 3.48917E-012 | .282 | -3.2879E-012 | 1.0926E-011 |
| | | BMHDay2 | -7.13086E-013 | 3.48917E-012 | .839 | -7.8203E-012 | 6.3941E-012 |
| steAla | BFHDay1 | PFHDay1 | 9.71912E-013 | 1.96960E-012 | .625 | -3.0451E-012 | 4.9889E-012 |
| | | BMHDay1 | -6.59350E-013 | 1.96960E-012 | .740 | -4.6764E-012 | 3.3577E-012 |
| | | PMHDay1 | -1.05495E-012 | 1.96960E-012 | .596 | -5.0720E-012 | 2.9621E-012 |
| | | BFHDay2 | 4.92668E-013 | 2.08908E-012 | .815 | -3.7680E-012 | 4.7534E-012 |
| | | PFHDay2 | -3.85852E-013 | 1.96960E-012 | .846 | -4.4029E-012 | 3.6312E-012 |
| | | BMHDay2 | 2.06349E-012 | 1.96960E-012 | .303 | -1.9535E-012 | 6.0805E-012 |
| | | PMHDay2 | 1.36263E-012 | 1.96960E-012 | .494 | -2.6544E-012 | 5.3797E-012 |
| | PFHDay1 | BFHDay1 | -9.71912E-013 | 1.96960E-012 | .625 | -4.9889E-012 | 3.0451E-012 |
| | | BMHDay1 | -1.63126E-012 | 1.96960E-012 | .414 | -5.6483E-012 | 2.3858E-012 |
| | | PMHDay1 | -2.02686E-012 | 1.96960E-012 | .311 | -6.0439E-012 | 1.9902E-012 |
| | | BFHDay2 | -4.79244E-013 | 2.08908E-012 | .820 | -4.7400E-012 | 3.7815E-012 |
| | | PFHDay2 | -1.35776E-012 | 1.96960E-012 | .496 | -5.3748E-012 | 2.6593E-012 |
| | | BMHDay2 | 1.09158E-012 | 1.96960E-012 | .583 | -2.9255E-012 | 5.1086E-012 |
| | | PMHDay2 | 3.90722E-013 | 1.96960E-012 | .844 | -3.6263E-012 | 4.4078E-012 |
| | BMHDay1 | BFHDay1 | 6.59350E-013 | 1.96960E-012 | .740 | -3.3577E-012 | 4.6764E-012 |

| | | | | | | |
|---|---|---|---|---|---|---|
| | PFHDay1 | 1.63126E-012 | 1.96960E-012 | .414 | -2.3858E-012 | 5.6483E-012 |
| | PMHDay1 | -3.95600E-013 | 1.96960E-012 | .842 | -4.4126E-012 | 3.6214E-012 |
| | BFHDay2 | 1.15202E-012 | 2.08908E-012 | .585 | -3.1087E-012 | 5.4127E-012 |
| | PFHDay2 | 2.73498E-013 | 1.96960E-012 | .890 | -3.7435E-012 | 4.2905E-012 |
| | BMHDay2 | 2.72284E-012 | 1.96960E-012 | .177 | -1.2942E-012 | 6.7399E-012 |
| | PMHDay2 | 2.02198E-012 | 1.96960E-012 | .313 | -1.9951E-012 | 6.0390E-012 |
| PMHDay1 | BFHDay1 | 1.05495E-012 | 1.96960E-012 | .596 | -2.9621E-012 | 5.0720E-012 |
| | PFHDay1 | 2.02686E-012 | 1.96960E-012 | .311 | -1.9902E-012 | 6.0439E-012 |
| | BMHDay1 | 3.95600E-013 | 1.96960E-012 | .842 | -3.6214E-012 | 4.4126E-012 |
| | BFHDay2 | 1.54762E-012 | 2.08908E-012 | .464 | -2.7131E-012 | 5.8083E-012 |
| | PFHDay2 | 6.69098E-013 | 1.96960E-012 | .736 | -3.3479E-012 | 4.6861E-012 |
| | BMHDay2 | 3.11844E-012 | 1.96960E-012 | .124 | -8.9860E-013 | 7.1355E-012 |
| | PMHDay2 | 2.41758E-012 | 1.96960E-012 | .229 | -1.5995E-012 | 6.4346E-012 |
| BFHDay2 | BFHDay1 | -4.92668E-013 | 2.08908E-012 | .815 | -4.7534E-012 | 3.7680E-012 |
| | PFHDay1 | 4.79244E-013 | 2.08908E-012 | .820 | -3.7815E-012 | 4.7400E-012 |
| | BMHDay1 | -1.15202E-012 | 2.08908E-012 | .585 | -5.4127E-012 | 3.1087E-012 |
| | PMHDay1 | -1.54762E-012 | 2.08908E-012 | .464 | -5.8083E-012 | 2.7131E-012 |
| | PFHDay2 | -8.78520E-013 | 2.08908E-012 | .677 | -5.1392E-012 | 3.3822E-012 |
| | BMHDay2 | 1.57082E-012 | 2.08908E-012 | .458 | -2.6899E-012 | 5.8315E-012 |

| | | | | | | |
|---|---|---|---|---|---|---|
| | PMHDay2 | 8.69966E-013 | 2.08908E-012 | .680 | -3.3907E-012 | 5.1307E-012 |
| PFHDay2 | BFHDay1 | 3.85852E-013 | 1.96960E-012 | .846 | -3.6312E-012 | 4.4029E-012 |
| | PFHDay1 | 1.35776E-012 | 1.96960E-012 | .496 | -2.6593E-012 | 5.3748E-012 |
| | BMHDay1 | -2.73498E-013 | 1.96960E-012 | .890 | -4.2905E-012 | 3.7435E-012 |
| | PMHDay1 | -6.69098E-013 | 1.96960E-012 | .736 | -4.6861E-012 | 3.3479E-012 |
| | BFHDay2 | 8.78520E-013 | 2.08908E-012 | .677 | -3.3822E-012 | 5.1392E-012 |
| | BMHDay2 | 2.44934E-012 | 1.96960E-012 | .223 | -1.5677E-012 | 6.4664E-012 |
| | PMHDay2 | 1.74849E-012 | 1.96960E-012 | .382 | -2.2685E-012 | 5.7655E-012 |
| BMHDay2 | BFHDay1 | -2.06349E-012 | 1.96960E-012 | .303 | -6.0805E-012 | 1.9535E-012 |
| | PFHDay1 | -1.09158E-012 | 1.96960E-012 | .583 | -5.1086E-012 | 2.9255E-012 |
| | BMHDay1 | -2.72284E-012 | 1.96960E-012 | .177 | -6.7399E-012 | 1.2942E-012 |
| | PMHDay1 | -3.11844E-012 | 1.96960E-012 | .124 | -7.1355E-012 | 8.9860E-013 |
| | BFHDay2 | -1.57082E-012 | 2.08908E-012 | .458 | -5.8315E-012 | 2.6899E-012 |
| | PFHDay2 | -2.44934E-012 | 1.96960E-012 | .223 | -6.4664E-012 | 1.5677E-012 |
| | PMHDay2 | -7.00854E-013 | 1.96960E-012 | .724 | -4.7179E-012 | 3.3162E-012 |
| PMHDay2 | BFHDay1 | -1.36263E-012 | 1.96960E-012 | .494 | -5.3797E-012 | 2.6544E-012 |
| | PFHDay1 | -3.90722E-013 | 1.96960E-012 | .844 | -4.4078E-012 | 3.6263E-012 |
| | BMHDay1 | -2.02198E-012 | 1.96960E-012 | .313 | -6.0390E-012 | 1.9951E-012 |
| | PMHDay1 | -2.41758E-012 | 1.96960E-012 | .229 | -6.4346E-012 | 1.5995E-012 |

| | | | | | | | |
|---|---|---|---|---|---|---|---|
| | | BFHDay2 | -8.69966E-013 | 2.08908E-012 | .680 | -5.1307E-012 | 3.3907E-012 |
| | | PFHDay2 | -1.74849E-012 | 1.96960E-012 | .382 | -5.7655E-012 | 2.2685E-012 |
| | | BMHDay2 | 7.00854E-013 | 1.96960E-012 | .724 | -3.3162E-012 | 4.7179E-012 |
| oleAla | BFHDay1 | PFHDay1 | 1.65079E-012 | 4.48786E-012 | .716 | -7.5146E-012 | 1.0816E-011 |
| | | BMHDay1 | 8.83907E-012 | 4.48786E-012 | .058 | -3.2638E-013 | 1.8005E-011 |
| | | PMHDay1 | 6.00724E-013 | 4.48786E-012 | .894 | -8.5647E-012 | 9.7662E-012 |
| | | BFHDay2 | 6.26568E-012 | 4.48786E-012 | .173 | -2.8998E-012 | 1.5431E-011 |
| | | PFHDay2 | 6.02133E-014 | 5.18214E-012 | .991 | -1.0523E-011 | 1.0644E-011 |
| | | BMHDay2 | 5.84517E-012 | 4.48786E-012 | .203 | -3.3203E-012 | 1.5011E-011 |
| | | PMHDay2 | 1.54335E-012 | 4.48786E-012 | .733 | -7.6221E-012 | 1.0709E-011 |
| | PFHDay1 | BFHDay1 | -1.65079E-012 | 4.48786E-012 | .716 | -1.0816E-011 | 7.5146E-012 |
| | | BMHDay1 | 7.18827E-012 | 4.48786E-012 | .120 | -1.9772E-012 | 1.6354E-011 |
| | | PMHDay1 | -1.05007E-012 | 4.48786E-012 | .817 | -1.0216E-011 | 8.1154E-012 |
| | | BFHDay2 | 4.61489E-012 | 4.48786E-012 | .312 | -4.5505E-012 | 1.3780E-011 |
| | | PFHDay2 | -1.59058E-012 | 5.18214E-012 | .761 | -1.2174E-011 | 8.9928E-012 |
| | | BMHDay2 | 4.19438E-012 | 4.48786E-012 | .357 | -4.9711E-012 | 1.3360E-011 |
| | | PMHDay2 | -1.07446E-013 | 4.48786E-012 | .981 | -9.2729E-012 | 9.0580E-012 |
| | BMHDay1 | BFHDay1 | -8.83907E-012 | 4.48786E-012 | .058 | -1.8005E-011 | 3.2638E-013 |
| | | PFHDay1 | -7.18827E-012 | 4.48786E-012 | .120 | -1.6354E-011 | 1.9772E-012 |

| | | | | | | |
|---|---|---|---|---|---|---|
| | PMHDay1 | -8.23834E-012 | 4.48786E-012 | .076 | -1.7404E-011 | 9.2710E-013 |
| | BFHDay2 | -2.57338E-012 | 4.48786E-012 | .571 | -1.1739E-011 | 6.5921E-012 |
| | PFHDay2 | -8.77885E-012 | 5.18214E-012 | .101 | -1.9362E-011 | 1.8045E-012 |
| | BMHDay2 | -2.99390E-012 | 4.48786E-012 | .510 | -1.2159E-011 | 6.1715E-012 |
| | PMHDay2 | -7.29572E-012 | 4.48786E-012 | .114 | -1.6461E-011 | 1.8697E-012 |
| PMHDay1 | BFHDay1 | -6.00724E-013 | 4.48786E-012 | .894 | -9.7662E-012 | 8.5647E-012 |
| | PFHDay1 | 1.05007E-012 | 4.48786E-012 | .817 | -8.1154E-012 | 1.0216E-011 |
| | BMHDay1 | 8.23834E-012 | 4.48786E-012 | .076 | -9.2710E-013 | 1.7404E-011 |
| | BFHDay2 | 5.66496E-012 | 4.48786E-012 | .217 | -3.5005E-012 | 1.4830E-011 |
| | PFHDay2 | -5.40511E-013 | 5.18214E-012 | .918 | -1.1124E-011 | 1.0043E-011 |
| | BMHDay2 | 5.24444E-012 | 4.48786E-012 | .252 | -3.9210E-012 | 1.4410E-011 |
| | PMHDay2 | 9.42622E-013 | 4.48786E-012 | .835 | -8.2228E-012 | 1.0108E-011 |
| BFHDay2 | BFHDay1 | -6.26568E-012 | 4.48786E-012 | .173 | -1.5431E-011 | 2.8998E-012 |
| | PFHDay1 | -4.61489E-012 | 4.48786E-012 | .312 | -1.3780E-011 | 4.5505E-012 |
| | BMHDay1 | 2.57338E-012 | 4.48786E-012 | .571 | -6.5921E-012 | 1.1739E-011 |
| | PMHDay1 | -5.66496E-012 | 4.48786E-012 | .217 | -1.4830E-011 | 3.5005E-012 |
| | PFHDay2 | -6.20547E-012 | 5.18214E-012 | .240 | -1.6789E-011 | 4.3779E-012 |
| | BMHDay2 | -4.20516E-013 | 4.48786E-012 | .926 | -9.5860E-012 | 8.7449E-012 |
| | PMHDay2 | -4.72234E-012 | 4.48786E-012 | .301 | -1.3888E-011 | 4.4431E-012 |

| | | | | | | |
|---|---|---|---|---|---|---|
| PFHDay2 | BFHDay1 | -6.02133E-014 | 5.18214E-012 | .991 | -1.0644E-011 | 1.0523E-011 |
| | PFHDay1 | 1.59058E-012 | 5.18214E-012 | .761 | -8.9928E-012 | 1.2174E-011 |
| | BMHDay1 | 8.77885E-012 | 5.18214E-012 | .101 | -1.8045E-012 | 1.9362E-011 |
| | PMHDay1 | 5.40511E-013 | 5.18214E-012 | .918 | -1.0043E-011 | 1.1124E-011 |
| | BFHDay2 | 6.20547E-012 | 5.18214E-012 | .240 | -4.3779E-012 | 1.6789E-011 |
| | BMHDay2 | 5.78495E-012 | 5.18214E-012 | .273 | -4.7984E-012 | 1.6368E-011 |
| | PMHDay2 | 1.48313E-012 | 5.18214E-012 | .777 | -9.1002E-012 | 1.2066E-011 |
| BMHDay2 | BFHDay1 | -5.84517E-012 | 4.48786E-012 | .203 | -1.5011E-011 | 3.3203E-012 |
| | PFHDay1 | -4.19438E-012 | 4.48786E-012 | .357 | -1.3360E-011 | 4.9711E-012 |
| | BMHDay1 | 2.99390E-012 | 4.48786E-012 | .510 | -6.1715E-012 | 1.2159E-011 |
| | PMHDay1 | -5.24444E-012 | 4.48786E-012 | .252 | -1.4410E-011 | 3.9210E-012 |
| | BFHDay2 | 4.20516E-013 | 4.48786E-012 | .926 | -8.7449E-012 | 9.5860E-012 |
| | PFHDay2 | -5.78495E-012 | 5.18214E-012 | .273 | -1.6368E-011 | 4.7984E-012 |
| | PMHDay2 | -4.30182E-012 | 4.48786E-012 | .345 | -1.3467E-011 | 4.8636E-012 |
| PMHDay2 | BFHDay1 | -1.54335E-012 | 4.48786E-012 | .733 | -1.0709E-011 | 7.6221E-012 |
| | PFHDay1 | 1.07446E-013 | 4.48786E-012 | .981 | -9.0580E-012 | 9.2729E-012 |
| | BMHDay1 | 7.29572E-012 | 4.48786E-012 | .114 | -1.8697E-012 | 1.6461E-011 |
| | PMHDay1 | -9.42622E-013 | 4.48786E-012 | .835 | -1.0108E-011 | 8.2228E-012 |
| | BFHDay2 | 4.72234E-012 | 4.48786E-012 | .301 | -4.4431E-012 | 1.3888E-011 |

| | | | | | | | |
|---|---|---|---|---|---|---|---|
| | | PFHDay2 | -1.48313E-012 | 5.18214E-012 | .777 | -1.2066E-011 | 9.1002E-012 |
| | | BMHDay2 | 4.30182E-012 | 4.48786E-012 | .345 | -4.8636E-012 | 1.3467E-011 |
| linAla | BFHDay1 | PFHDay1 | 7.36068E-012 | 4.16566E-012 | .087 | -1.1245E-012 | 1.5846E-011 |
| | | BMHDay1 | 8.85762E-012* | 4.16566E-012 | .041 | 3.7246E-013 | 1.7343E-011 |
| | | PMHDay1 | 8.99291E-012* | 4.16566E-012 | .038 | 5.0774E-013 | 1.7478E-011 |
| | | BFHDay2 | 6.63491E-012 | 4.16566E-012 | .121 | -1.8503E-012 | 1.5120E-011 |
| | | PFHDay2 | 6.55189E-012 | 4.16566E-012 | .126 | -1.9333E-012 | 1.5037E-011 |
| | | BMHDay2 | 6.22954E-012 | 4.16566E-012 | .145 | -2.2556E-012 | 1.4715E-011 |
| | | PMHDay2 | 7.48962E-012 | 4.16566E-012 | .082 | -9.9555E-013 | 1.5975E-011 |
| | PFHDay1 | BFHDay1 | -7.36068E-012 | 4.16566E-012 | .087 | -1.5846E-011 | 1.1245E-012 |
| | | BMHDay1 | 1.49695E-012 | 4.16566E-012 | .722 | -6.9882E-012 | 9.9821E-012 |
| | | PMHDay1 | 1.63224E-012 | 4.16566E-012 | .698 | -6.8529E-012 | 1.0117E-011 |
| | | BFHDay2 | -7.25766E-013 | 4.16566E-012 | .863 | -9.2109E-012 | 7.7594E-012 |
| | | PFHDay2 | -8.08788E-013 | 4.16566E-012 | .847 | -9.2940E-012 | 7.6764E-012 |
| | | BMHDay2 | -1.13114E-012 | 4.16566E-012 | .788 | -9.6163E-012 | 7.3540E-012 |
| | | PMHDay2 | 1.28940E-013 | 4.16566E-012 | .975 | -8.3562E-012 | 8.6141E-012 |
| | BMHDay1 | BFHDay1 | -8.85762E-012* | 4.16566E-012 | .041 | -1.7343E-011 | -3.7246E-013 |
| | | PFHDay1 | -1.49695E-012 | 4.16566E-012 | .722 | -9.9821E-012 | 6.9882E-012 |
| | | PMHDay1 | 1.35287E-013 | 4.16566E-012 | .974 | -8.3499E-012 | 8.6205E-012 |

| | | | | | | |
|---|---|---|---|---|---|---|
| | BFHDay2 | -2.22271E-012 | 4.16566E-012 | .597 | -1.0708E-011 | 6.2625E-012 |
| | PFHDay2 | -2.30574E-012 | 4.16566E-012 | .584 | -1.0791E-011 | 6.1794E-012 |
| | BMHDay2 | -2.62809E-012 | 4.16566E-012 | .533 | -1.1113E-011 | 5.8571E-012 |
| | PMHDay2 | -1.36801E-012 | 4.16566E-012 | .745 | -9.8532E-012 | 7.1172E-012 |
| PMHDay1 | BFHDay1 | -8.99291E-012* | 4.16566E-012 | .038 | -1.7478E-011 | -5.0774E-013 |
| | PFHDay1 | -1.63224E-012 | 4.16566E-012 | .698 | -1.0117E-011 | 6.8529E-012 |
| | BMHDay1 | -1.35287E-013 | 4.16566E-012 | .974 | -8.6205E-012 | 8.3499E-012 |
| | BFHDay2 | -2.35800E-012 | 4.16566E-012 | .575 | -1.0843E-011 | 6.1272E-012 |
| | PFHDay2 | -2.44102E-012 | 4.16566E-012 | .562 | -1.0926E-011 | 6.0441E-012 |
| | BMHDay2 | -2.76337E-012 | 4.16566E-012 | .512 | -1.1249E-011 | 5.7218E-012 |
| | PMHDay2 | -1.50330E-012 | 4.16566E-012 | .721 | -9.9885E-012 | 6.9819E-012 |
| BFHDay2 | BFHDay1 | -6.63491E-012 | 4.16566E-012 | .121 | -1.5120E-011 | 1.8503E-012 |
| | PFHDay1 | 7.25766E-013 | 4.16566E-012 | .863 | -7.7594E-012 | 9.2109E-012 |
| | BMHDay1 | 2.22271E-012 | 4.16566E-012 | .597 | -6.2625E-012 | 1.0708E-011 |
| | PMHDay1 | 2.35800E-012 | 4.16566E-012 | .575 | -6.1272E-012 | 1.0843E-011 |
| | PFHDay2 | -8.30220E-014 | 4.16566E-012 | .984 | -8.5682E-012 | 8.4021E-012 |
| | BMHDay2 | -4.05372E-013 | 4.16566E-012 | .923 | -8.8905E-012 | 8.0798E-012 |
| | PMHDay2 | 8.54706E-013 | 4.16566E-012 | .839 | -7.6305E-012 | 9.3399E-012 |
| PFHDay2 | BFHDay1 | -6.55189E-012 | 4.16566E-012 | .126 | -1.5037E-011 | 1.9333E-012 |

| | | | | | | |
|---|---|---|---|---|---|---|
| | PFHDay1 | 8.08788E-013 | 4.16566E-012 | .847 | -7.6764E-012 | 9.2940E-012 |
| | BMHDay1 | 2.30574E-012 | 4.16566E-012 | .584 | -6.1794E-012 | 1.0791E-011 |
| | PMHDay1 | 2.44102E-012 | 4.16566E-012 | .562 | -6.0441E-012 | 1.0926E-011 |
| | BFHDay2 | 8.30220E-014 | 4.16566E-012 | .984 | -8.4021E-012 | 8.5682E-012 |
| | BMHDay2 | -3.22350E-013 | 4.16566E-012 | .939 | -8.8075E-012 | 8.1628E-012 |
| | PMHDay2 | 9.37728E-013 | 4.16566E-012 | .823 | -7.5474E-012 | 9.4229E-012 |
| BMHDay2 | BFHDay1 | -6.22954E-012 | 4.16566E-012 | .145 | -1.4715E-011 | 2.2556E-012 |
| | PFHDay1 | 1.13114E-012 | 4.16566E-012 | .788 | -7.3540E-012 | 9.6163E-012 |
| | BMHDay1 | 2.62809E-012 | 4.16566E-012 | .533 | -5.8571E-012 | 1.1113E-011 |
| | PMHDay1 | 2.76337E-012 | 4.16566E-012 | .512 | -5.7218E-012 | 1.1249E-011 |
| | BFHDay2 | 4.05372E-013 | 4.16566E-012 | .923 | -8.0798E-012 | 8.8905E-012 |
| | PFHDay2 | 3.22350E-013 | 4.16566E-012 | .939 | -8.1628E-012 | 8.8075E-012 |
| | PMHDay2 | 1.26008E-012 | 4.16566E-012 | .764 | -7.2251E-012 | 9.7452E-012 |
| PMHDay2 | BFHDay1 | -7.48962E-012 | 4.16566E-012 | .082 | -1.5975E-011 | 9.9555E-013 |
| | PFHDay1 | -1.28940E-013 | 4.16566E-012 | .975 | -8.6141E-012 | 8.3562E-012 |
| | BMHDay1 | 1.36801E-012 | 4.16566E-012 | .745 | -7.1172E-012 | 9.8532E-012 |
| | PMHDay1 | 1.50330E-012 | 4.16566E-012 | .721 | -6.9819E-012 | 9.9885E-012 |
| | BFHDay2 | -8.54706E-013 | 4.16566E-012 | .839 | -9.3399E-012 | 7.6305E-012 |
| | PFHDay2 | -9.37728E-013 | 4.16566E-012 | .823 | -9.4229E-012 | 7.5474E-012 |

| | | | | | | | |
|---|---|---|---|---|---|---|---|
| | | BMHDay2 | -1.26008E-012 | 4.16566E-012 | .764 | -9.7452E-012 | 7.2251E-012 |
| palGABA | BFHDay1 | PFHDay1 | -1.13882E-013 | 1.10684E-013 | .311 | -3.3934E-013 | 1.1157E-013 |
| | | BMHDay1 | 1.24843E-013 | 1.10684E-013 | .268 | -1.0061E-013 | 3.5030E-013 |
| | | PMHDay1 | 6.58082E-014 | 1.10684E-013 | .556 | -1.5965E-013 | 2.9126E-013 |
| | | BFHDay2 | 2.09514E-014 | 1.10684E-013 | .851 | -2.0450E-013 | 2.4641E-013 |
| | | PFHDay2 | 7.54027E-014 | 1.10684E-013 | .501 | -1.5005E-013 | 3.0086E-013 |
| | | BMHDay2 | -6.98816E-015 | 1.10684E-013 | .950 | -2.3244E-013 | 2.1847E-013 |
| | | PMHDay2 | 3.11424E-014 | 1.10684E-013 | .780 | -1.9431E-013 | 2.5660E-013 |
| | PFHDay1 | BFHDay1 | 1.13882E-013 | 1.10684E-013 | .311 | -1.1157E-013 | 3.3934E-013 |
| | | BMHDay1 | 2.38725E-013* | 1.10684E-013 | .039 | 1.3270E-014 | 4.6418E-013 |
| | | PMHDay1 | 1.79690E-013 | 1.10684E-013 | .114 | -4.5765E-014 | 4.0515E-013 |
| | | BFHDay2 | 1.34834E-013 | 1.10684E-013 | .232 | -9.0622E-014 | 3.6029E-013 |
| | | PFHDay2 | 1.89285E-013 | 1.10684E-013 | .097 | -3.6171E-014 | 4.1474E-013 |
| | | BMHDay2 | 1.06894E-013 | 1.10684E-013 | .341 | -1.1856E-013 | 3.3235E-013 |
| | | PMHDay2 | 1.45025E-013 | 1.10684E-013 | .199 | -8.0431E-014 | 3.7048E-013 |
| | BMHDay1 | BFHDay1 | -1.24843E-013 | 1.10684E-013 | .268 | -3.5030E-013 | 1.0061E-013 |
| | | PFHDay1 | -2.38725E-013* | 1.10684E-013 | .039 | -4.6418E-013 | -1.3270E-014 |
| | | PMHDay1 | -5.90350E-014 | 1.10684E-013 | .597 | -2.8449E-013 | 1.6642E-013 |
| | | BFHDay2 | -1.03892E-013 | 1.10684E-013 | .355 | -3.2935E-013 | 1.2156E-013 |

| | | | | | | |
|---|---|---|---|---|---|---|
| | PFHDay2 | -4.94405E-014 | 1.10684E-013 | .658 | -2.7490E-013 | 1.7601E-013 |
| | BMHDay2 | -1.31831E-013 | 1.10684E-013 | .242 | -3.5729E-013 | 9.3624E-014 |
| | PMHDay2 | -9.37008E-014 | 1.10684E-013 | .404 | -3.1916E-013 | 1.3175E-013 |
| PMHDay1 | BFHDay1 | -6.58082E-014 | 1.10684E-013 | .556 | -2.9126E-013 | 1.5965E-013 |
| | PFHDay1 | -1.79690E-013 | 1.10684E-013 | .114 | -4.0515E-013 | 4.5765E-014 |
| | BMHDay1 | 5.90350E-014 | 1.10684E-013 | .597 | -1.6642E-013 | 2.8449E-013 |
| | BFHDay2 | -4.48568E-014 | 1.10684E-013 | .688 | -2.7031E-013 | 1.8060E-013 |
| | PFHDay2 | 9.59444E-015 | 1.10684E-013 | .931 | -2.1586E-013 | 2.3505E-013 |
| | BMHDay2 | -7.27964E-014 | 1.10684E-013 | .515 | -2.9825E-013 | 1.5266E-013 |
| | PMHDay2 | -3.46658E-014 | 1.10684E-013 | .756 | -2.6012E-013 | 1.9079E-013 |
| BFHDay2 | BFHDay1 | -2.09514E-014 | 1.10684E-013 | .851 | -2.4641E-013 | 2.0450E-013 |
| | PFHDay1 | -1.34834E-013 | 1.10684E-013 | .232 | -3.6029E-013 | 9.0622E-014 |
| | BMHDay1 | 1.03892E-013 | 1.10684E-013 | .355 | -1.2156E-013 | 3.2935E-013 |
| | PMHDay1 | 4.48568E-014 | 1.10684E-013 | .688 | -1.8060E-013 | 2.7031E-013 |
| | PFHDay2 | 5.44512E-014 | 1.10684E-013 | .626 | -1.7100E-013 | 2.7991E-013 |
| | BMHDay2 | -2.79396E-014 | 1.10684E-013 | .802 | -2.5340E-013 | 1.9752E-013 |
| | PMHDay2 | 1.01910E-014 | 1.10684E-013 | .927 | -2.1526E-013 | 2.3565E-013 |
| PFHDay2 | BFHDay1 | -7.54027E-014 | 1.10684E-013 | .501 | -3.0086E-013 | 1.5005E-013 |
| | PFHDay1 | -1.89285E-013 | 1.10684E-013 | .097 | -4.1474E-013 | 3.6171E-014 |

| | | | | | | |
|---|---|---|---|---|---|---|
| | BMHDay1 | 4.94405E-014 | 1.10684E-013 | .658 | -1.7601E-013 | 2.7490E-013 |
| | PMHDay1 | -9.59444E-015 | 1.10684E-013 | .931 | -2.3505E-013 | 2.1586E-013 |
| | BFHDay2 | -5.44512E-014 | 1.10684E-013 | .626 | -2.7991E-013 | 1.7100E-013 |
| | BMHDay2 | -8.23908E-014 | 1.10684E-013 | .462 | -3.0785E-013 | 1.4306E-013 |
| | PMHDay2 | -4.42603E-014 | 1.10684E-013 | .692 | -2.6972E-013 | 1.8120E-013 |
| BMHDay2 | BFHDay1 | 6.98816E-015 | 1.10684E-013 | .950 | -2.1847E-013 | 2.3244E-013 |
| | PFHDay1 | -1.06894E-013 | 1.10684E-013 | .341 | -3.3235E-013 | 1.1856E-013 |
| | BMHDay1 | 1.31831E-013 | 1.10684E-013 | .242 | -9.3624E-014 | 3.5729E-013 |
| | PMHDay1 | 7.27964E-014 | 1.10684E-013 | .515 | -1.5266E-013 | 2.9825E-013 |
| | BFHDay2 | 2.79396E-014 | 1.10684E-013 | .802 | -1.9752E-013 | 2.5340E-013 |
| | PFHDay2 | 8.23908E-014 | 1.10684E-013 | .462 | -1.4306E-013 | 3.0785E-013 |
| | PMHDay2 | 3.81306E-014 | 1.10684E-013 | .733 | -1.8732E-013 | 2.6359E-013 |
| PMHDay2 | BFHDay1 | -3.11424E-014 | 1.10684E-013 | .780 | -2.5660E-013 | 1.9431E-013 |
| | PFHDay1 | -1.45025E-013 | 1.10684E-013 | .199 | -3.7048E-013 | 8.0431E-014 |
| | BMHDay1 | 9.37008E-014 | 1.10684E-013 | .404 | -1.3175E-013 | 3.1916E-013 |
| | PMHDay1 | 3.46658E-014 | 1.10684E-013 | .756 | -1.9079E-013 | 2.6012E-013 |
| | BFHDay2 | -1.01910E-014 | 1.10684E-013 | .927 | -2.3565E-013 | 2.1526E-013 |
| | PFHDay2 | 4.42603E-014 | 1.10684E-013 | .692 | -1.8120E-013 | 2.6972E-013 |
| | BMHDay2 | -3.81306E-014 | 1.10684E-013 | .733 | -2.6359E-013 | 1.8732E-013 |

| | | | | | | | |
|---|---|---|---|---|---|---|---|
| steGABA | BFHDay1 | PFHDay1 | 4.26474E-014 | 4.44183E-014 | .344 | -4.7830E-014 | 1.3312E-013 |
| | | BMHDay1 | 4.87588E-014 | 4.44183E-014 | .281 | -4.1718E-014 | 1.3924E-013 |
| | | PMHDay1 | 4.38491E-014 | 4.44183E-014 | .331 | -4.6628E-014 | 1.3433E-013 |
| | | BFHDay2 | 2.72378E-014 | 4.44183E-014 | .544 | -6.3239E-014 | 1.1771E-013 |
| | | PFHDay2 | 2.66477E-014 | 4.44183E-014 | .553 | -6.3829E-014 | 1.1712E-013 |
| | | BMHDay2 | -3.64320E-016 | 4.44183E-014 | .994 | -9.0841E-014 | 9.0113E-014 |
| | | PMHDay2 | 1.41881E-014 | 4.44183E-014 | .751 | -7.6289E-014 | 1.0467E-013 |
| | PFHDay1 | BFHDay1 | -4.26474E-014 | 4.44183E-014 | .344 | -1.3312E-013 | 4.7830E-014 |
| | | BMHDay1 | 6.11132E-015 | 4.44183E-014 | .891 | -8.4366E-014 | 9.6588E-014 |
| | | PMHDay1 | 1.20170E-015 | 4.44183E-014 | .979 | -8.9275E-014 | 9.1679E-014 |
| | | BFHDay2 | -1.54096E-014 | 4.44183E-014 | .731 | -1.0589E-013 | 7.5068E-014 |
| | | PFHDay2 | -1.59997E-014 | 4.44183E-014 | .721 | -1.0648E-013 | 7.4477E-014 |
| | | BMHDay2 | -4.30118E-014 | 4.44183E-014 | .340 | -1.3349E-013 | 4.7465E-014 |
| | | PMHDay2 | -2.84593E-014 | 4.44183E-014 | .526 | -1.1894E-013 | 6.2018E-014 |
| | BMHDay1 | BFHDay1 | -4.87588E-014 | 4.44183E-014 | .281 | -1.3924E-013 | 4.1718E-014 |
| | | PFHDay1 | -6.11132E-015 | 4.44183E-014 | .891 | -9.6588E-014 | 8.4366E-014 |
| | | PMHDay1 | -4.90962E-015 | 4.44183E-014 | .913 | -9.5387E-014 | 8.5567E-014 |
| | | BFHDay2 | -2.15209E-014 | 4.44183E-014 | .631 | -1.1200E-013 | 6.8956E-014 |
| | | PFHDay2 | -2.21111E-014 | 4.44183E-014 | .622 | -1.1259E-013 | 6.8366E-014 |

| | | | | | | |
|---|---|---|---|---|---|---|
| | | BMHDay2 | -4.91231E-014 | 4.44183E-014 | .277 | -1.3960E-013 | 4.1354E-014 |
| | | PMHDay2 | -3.45706E-014 | 4.44183E-014 | .442 | -1.2505E-013 | 5.5906E-014 |
| | PMHDay1 | BFHDay1 | -4.38491E-014 | 4.44183E-014 | .331 | -1.3433E-013 | 4.6628E-014 |
| | | PFHDay1 | -1.20170E-015 | 4.44183E-014 | .979 | -9.1679E-014 | 8.9275E-014 |
| | | BMHDay1 | 4.90962E-015 | 4.44183E-014 | .913 | -8.5567E-014 | 9.5387E-014 |
| | | BFHDay2 | -1.66113E-014 | 4.44183E-014 | .711 | -1.0709E-013 | 7.3866E-014 |
| | | PFHDay2 | -1.72014E-014 | 4.44183E-014 | .701 | -1.0768E-013 | 7.3276E-014 |
| | | BMHDay2 | -4.42135E-014 | 4.44183E-014 | .327 | -1.3469E-013 | 4.6264E-014 |
| | | PMHDay2 | -2.96610E-014 | 4.44183E-014 | .509 | -1.2014E-013 | 6.0816E-014 |
| | BFHDay2 | BFHDay1 | -2.72378E-014 | 4.44183E-014 | .544 | -1.1771E-013 | 6.3239E-014 |
| | | PFHDay1 | 1.54096E-014 | 4.44183E-014 | .731 | -7.5068E-014 | 1.0589E-013 |
| | | BMHDay1 | 2.15209E-014 | 4.44183E-014 | .631 | -6.8956E-014 | 1.1200E-013 |
| | | PMHDay1 | 1.66113E-014 | 4.44183E-014 | .711 | -7.3866E-014 | 1.0709E-013 |
| | | PFHDay2 | -5.90140E-016 | 4.44183E-014 | .989 | -9.1067E-014 | 8.9887E-014 |
| | | BMHDay2 | -2.76022E-014 | 4.44183E-014 | .539 | -1.1808E-013 | 6.2875E-014 |
| | | PMHDay2 | -1.30497E-014 | 4.44183E-014 | .771 | -1.0353E-013 | 7.7427E-014 |
| | PFHDay2 | BFHDay1 | -2.66477E-014 | 4.44183E-014 | .553 | -1.1712E-013 | 6.3829E-014 |
| | | PFHDay1 | 1.59997E-014 | 4.44183E-014 | .721 | -7.4477E-014 | 1.0648E-013 |
| | | BMHDay1 | 2.21111E-014 | 4.44183E-014 | .622 | -6.8366E-014 | 1.1259E-013 |

| | | | | | | | |
|---|---|---|---|---|---|---|---|
| | | PMHDay1 | 1.72014E-014 | 4.44183E-014 | .701 | -7.3276E-014 | 1.0768E-013 |
| | | BFHDay2 | 5.90140E-016 | 4.44183E-014 | .989 | -8.9887E-014 | 9.1067E-014 |
| | | BMHDay2 | -2.70120E-014 | 4.44183E-014 | .547 | -1.1749E-013 | 6.3465E-014 |
| | | PMHDay2 | -1.24596E-014 | 4.44183E-014 | .781 | -1.0294E-013 | 7.8018E-014 |
| | BMHDay2 | BFHDay1 | 3.64320E-016 | 4.44183E-014 | .994 | -9.0113E-014 | 9.0841E-014 |
| | | PFHDay1 | 4.30118E-014 | 4.44183E-014 | .340 | -4.7465E-014 | 1.3349E-013 |
| | | BMHDay1 | 4.91231E-014 | 4.44183E-014 | .277 | -4.1354E-014 | 1.3960E-013 |
| | | PMHDay1 | 4.42135E-014 | 4.44183E-014 | .327 | -4.6264E-014 | 1.3469E-013 |
| | | BFHDay2 | 2.76022E-014 | 4.44183E-014 | .539 | -6.2875E-014 | 1.1808E-013 |
| | | PFHDay2 | 2.70120E-014 | 4.44183E-014 | .547 | -6.3465E-014 | 1.1749E-013 |
| | | PMHDay2 | 1.45525E-014 | 4.44183E-014 | .745 | -7.5925E-014 | 1.0503E-013 |
| | PMHDay2 | BFHDay1 | -1.41881E-014 | 4.44183E-014 | .751 | -1.0467E-013 | 7.6289E-014 |
| | | PFHDay1 | 2.84593E-014 | 4.44183E-014 | .526 | -6.2018E-014 | 1.1894E-013 |
| | | BMHDay1 | 3.45706E-014 | 4.44183E-014 | .442 | -5.5906E-014 | 1.2505E-013 |
| | | PMHDay1 | 2.96610E-014 | 4.44183E-014 | .509 | -6.0816E-014 | 1.2014E-013 |
| | | BFHDay2 | 1.30497E-014 | 4.44183E-014 | .771 | -7.7427E-014 | 1.0353E-013 |
| | | PFHDay2 | 1.24596E-014 | 4.44183E-014 | .781 | -7.8018E-014 | 1.0294E-013 |
| | | BMHDay2 | -1.45525E-014 | 4.44183E-014 | .745 | -1.0503E-013 | 7.5925E-014 |
| oleGABA | BFHDay1 | PFHDay1 | -3.11558E-014 | 3.13807E-014 | .328 | -9.5157E-014 | 3.2846E-014 |

| | | | | | | |
|---|---|---|---|---|---|---|
| | | BMHDay1 | 3.37012E-015 | 3.13807E-014 | .915 | -6.0631E-014 | 6.7372E-014 |
| | | PMHDay1 | -4.73272E-014 | 3.32843E-014 | .165 | -1.1521E-013 | 2.0557E-014 |
| | | BFHDay2 | -1.70222E-014 | 3.13807E-014 | .591 | -8.1024E-014 | 4.6979E-014 |
| | | PFHDay2 | -8.11352E-015 | 3.13807E-014 | .798 | -7.2115E-014 | 5.5888E-014 |
| | | BMHDay2 | 1.36568E-015 | 3.13807E-014 | .966 | -6.2636E-014 | 6.5367E-014 |
| | | PMHDay2 | -1.02813E-013* | 3.13807E-014 | .003 | -1.6681E-013 | -3.8812E-014 |
| | PFHDay1 | BFHDay1 | 3.11558E-014 | 3.13807E-014 | .328 | -3.2846E-014 | 9.5157E-014 |
| | | BMHDay1 | 3.45259E-014 | 3.13807E-014 | .280 | -2.9476E-014 | 9.8527E-014 |
| | | PMHDay1 | -1.61714E-014 | 3.32843E-014 | .630 | -8.4055E-014 | 5.1712E-014 |
| | | BFHDay2 | 1.41337E-014 | 3.13807E-014 | .656 | -4.9868E-014 | 7.8135E-014 |
| | | PFHDay2 | 2.30423E-014 | 3.13807E-014 | .468 | -4.0959E-014 | 8.7044E-014 |
| | | BMHDay2 | 3.25215E-014 | 3.13807E-014 | .308 | -3.1480E-014 | 9.6523E-014 |
| | | PMHDay2 | -7.16575E-014* | 3.13807E-014 | .029 | -1.3566E-013 | -7.6561E-015 |
| | BMHDay1 | BFHDay1 | -3.37012E-015 | 3.13807E-014 | .915 | -6.7372E-014 | 6.0631E-014 |
| | | PFHDay1 | -3.45259E-014 | 3.13807E-014 | .280 | -9.8527E-014 | 2.9476E-014 |
| | | PMHDay1 | -5.06973E-014 | 3.32843E-014 | .138 | -1.1858E-013 | 1.7186E-014 |
| | | BFHDay2 | -2.03923E-014 | 3.13807E-014 | .521 | -8.4394E-014 | 4.3609E-014 |
| | | PFHDay2 | -1.14836E-014 | 3.13807E-014 | .717 | -7.5485E-014 | 5.2518E-014 |
| | | BMHDay2 | -2.00444E-015 | 3.13807E-014 | .949 | -6.6006E-014 | 6.1997E-014 |

| | | | | | | |
|---|---|---|---|---|---|---|
| | | PMHDay2 | -1.06183E-013* | 3.13807E-014 | .002 | -1.7018E-013 | -4.2182E-014 |
| | PMHDay1 | BFHDay1 | 4.73272E-014 | 3.32843E-014 | .165 | -2.0557E-014 | 1.1521E-013 |
| | | PFHDay1 | 1.61714E-014 | 3.32843E-014 | .630 | -5.1712E-014 | 8.4055E-014 |
| | | BMHDay1 | 5.06973E-014 | 3.32843E-014 | .138 | -1.7186E-014 | 1.1858E-013 |
| | | BFHDay2 | 3.03050E-014 | 3.32843E-014 | .370 | -3.7579E-014 | 9.8189E-014 |
| | | PFHDay2 | 3.92137E-014 | 3.32843E-014 | .248 | -2.8670E-014 | 1.0710E-013 |
| | | BMHDay2 | 4.86929E-014 | 3.32843E-014 | .154 | -1.9191E-014 | 1.1658E-013 |
| | | PMHDay2 | -5.54862E-014 | 3.32843E-014 | .106 | -1.2337E-013 | 1.2398E-014 |
| | BFHDay2 | BFHDay1 | 1.70222E-014 | 3.13807E-014 | .591 | -4.6979E-014 | 8.1024E-014 |
| | | PFHDay1 | -1.41337E-014 | 3.13807E-014 | .656 | -7.8135E-014 | 4.9868E-014 |
| | | BMHDay1 | 2.03923E-014 | 3.13807E-014 | .521 | -4.3609E-014 | 8.4394E-014 |
| | | PMHDay1 | -3.03050E-014 | 3.32843E-014 | .370 | -9.8189E-014 | 3.7579E-014 |
| | | PFHDay2 | 8.90864E-015 | 3.13807E-014 | .778 | -5.5093E-014 | 7.2910E-014 |
| | | BMHDay2 | 1.83878E-014 | 3.13807E-014 | .562 | -4.5614E-014 | 8.2389E-014 |
| | | PMHDay2 | -8.57912E-014* | 3.13807E-014 | .010 | -1.4979E-013 | -2.1790E-014 |
| | PFHDay2 | BFHDay1 | 8.11352E-015 | 3.13807E-014 | .798 | -5.5888E-014 | 7.2115E-014 |
| | | PFHDay1 | -2.30423E-014 | 3.13807E-014 | .468 | -8.7044E-014 | 4.0959E-014 |
| | | BMHDay1 | 1.14836E-014 | 3.13807E-014 | .717 | -5.2518E-014 | 7.5485E-014 |
| | | PMHDay1 | -3.92137E-014 | 3.32843E-014 | .248 | -1.0710E-013 | 2.8670E-014 |

| | | | | | | | |
|---|---|---|---|---|---|---|---|
| | | BFHDay2 | -8.90864E-015 | 3.13807E-014 | .778 | -7.2910E-014 | 5.5093E-014 |
| | | BMHDay2 | 9.47920E-015 | 3.13807E-014 | .765 | -5.4522E-014 | 7.3481E-014 |
| | | PMHDay2 | -9.46998E-014* | 3.13807E-014 | .005 | -1.5870E-013 | -3.0698E-014 |
| | BMHDay2 | BFHDay1 | -1.36568E-015 | 3.13807E-014 | .966 | -6.5367E-014 | 6.2636E-014 |
| | | PFHDay1 | -3.25215E-014 | 3.13807E-014 | .308 | -9.6523E-014 | 3.1480E-014 |
| | | BMHDay1 | 2.00444E-015 | 3.13807E-014 | .949 | -6.1997E-014 | 6.6006E-014 |
| | | PMHDay1 | -4.86929E-014 | 3.32843E-014 | .154 | -1.1658E-013 | 1.9191E-014 |
| | | BFHDay2 | -1.83878E-014 | 3.13807E-014 | .562 | -8.2389E-014 | 4.5614E-014 |
| | | PFHDay2 | -9.47920E-015 | 3.13807E-014 | .765 | -7.3481E-014 | 5.4522E-014 |
| | | PMHDay2 | -1.04179E-013* | 3.13807E-014 | .002 | -1.6818E-013 | -4.0178E-014 |
| | PMHDay2 | BFHDay1 | 1.02813E-013* | 3.13807E-014 | .003 | 3.8812E-014 | 1.6681E-013 |
| | | PFHDay1 | 7.16575E-014* | 3.13807E-014 | .029 | 7.6561E-015 | 1.3566E-013 |
| | | BMHDay1 | 1.06183E-013* | 3.13807E-014 | .002 | 4.2182E-014 | 1.7018E-013 |
| | | PMHDay1 | 5.54862E-014 | 3.32843E-014 | .106 | -1.2398E-014 | 1.2337E-013 |
| | | BFHDay2 | 8.57912E-014* | 3.13807E-014 | .010 | 2.1790E-014 | 1.4979E-013 |
| | | PFHDay2 | 9.46998E-014* | 3.13807E-014 | .005 | 3.0698E-014 | 1.5870E-013 |
| | | BMHDay2 | 1.04179E-013* | 3.13807E-014 | .002 | 4.0178E-014 | 1.6818E-013 |
| linGABA | BFHDay1 | PFHDay1 | 1.77730E-012 | 1.81883E-011 | .923 | -3.5318E-011 | 3.8873E-011 |
| | | BMHDay1 | -1.37854E-012 | 1.81883E-011 | .940 | -3.8474E-011 | 3.5717E-011 |

| | | | | | | |
|---|---|---|---|---|---|---|
| | PMHDay1 | 1.36050E-011 | 1.81883E-011 | .460 | -2.3490E-011 | 5.0700E-011 |
| | BFHDay2 | 1.46308E-011 | 1.92916E-011 | .454 | -2.4715E-011 | 5.3976E-011 |
| | PFHDay2 | -1.66166E-011 | 1.81883E-011 | .368 | -5.3712E-011 | 2.0479E-011 |
| | BMHDay2 | 8.41998E-012 | 1.81883E-011 | .647 | -2.8675E-011 | 4.5515E-011 |
| | PMHDay2 | -5.18064E-011* | 1.81883E-011 | .008 | -8.8902E-011 | -1.4711E-011 |
| PFHDay1 | BFHDay1 | -1.77730E-012 | 1.81883E-011 | .923 | -3.8873E-011 | 3.5318E-011 |
| | BMHDay1 | -3.15584E-012 | 1.81883E-011 | .863 | -4.0251E-011 | 3.3940E-011 |
| | PMHDay1 | 1.18277E-011 | 1.81883E-011 | .520 | -2.5268E-011 | 4.8923E-011 |
| | BFHDay2 | 1.28535E-011 | 1.92916E-011 | .510 | -2.6492E-011 | 5.2199E-011 |
| | PFHDay2 | -1.83939E-011 | 1.81883E-011 | .320 | -5.5489E-011 | 1.8701E-011 |
| | BMHDay2 | 6.64268E-012 | 1.81883E-011 | .717 | -3.0453E-011 | 4.3738E-011 |
| | PMHDay2 | -5.35837E-011* | 1.81883E-011 | .006 | -9.0679E-011 | -1.6488E-011 |
| BMHDay1 | BFHDay1 | 1.37854E-012 | 1.81883E-011 | .940 | -3.5717E-011 | 3.8474E-011 |
| | PFHDay1 | 3.15584E-012 | 1.81883E-011 | .863 | -3.3940E-011 | 4.0251E-011 |
| | PMHDay1 | 1.49836E-011 | 1.81883E-011 | .416 | -2.2112E-011 | 5.2079E-011 |
| | BFHDay2 | 1.60094E-011 | 1.92916E-011 | .413 | -2.3336E-011 | 5.5355E-011 |
| | PFHDay2 | -1.52380E-011 | 1.81883E-011 | .409 | -5.2333E-011 | 2.1857E-011 |
| | BMHDay2 | 9.79852E-012 | 1.81883E-011 | .594 | -2.7297E-011 | 4.6894E-011 |
| | PMHDay2 | -5.04278E-011* | 1.81883E-011 | .009 | -8.7523E-011 | -1.3332E-011 |

| | | | | | | | |
|---|---|---|---|---|---|---|---|
| | PMHDay1 | BFHDay1 | -1.36050E-011 | 1.81883E-011 | .460 | -5.0700E-011 | 2.3490E-011 |
| | | PFHDay1 | -1.18277E-011 | 1.81883E-011 | .520 | -4.8923E-011 | 2.5268E-011 |
| | | BMHDay1 | -1.49836E-011 | 1.81883E-011 | .416 | -5.2079E-011 | 2.2112E-011 |
| | | BFHDay2 | 1.02581E-012 | 1.92916E-011 | .958 | -3.8320E-011 | 4.0371E-011 |
| | | PFHDay2 | -3.02216E-011 | 1.81883E-011 | .107 | -6.7317E-011 | 6.8738E-012 |
| | | BMHDay2 | -5.18504E-012 | 1.81883E-011 | .777 | -4.2280E-011 | 3.1910E-011 |
| | | PMHDay2 | -6.54114E-011* | 1.81883E-011 | .001 | -1.0251E-010 | -2.8316E-011 |
| | BFHDay2 | BFHDay1 | -1.46308E-011 | 1.92916E-011 | .454 | -5.3976E-011 | 2.4715E-011 |
| | | PFHDay1 | -1.28535E-011 | 1.92916E-011 | .510 | -5.2199E-011 | 2.6492E-011 |
| | | BMHDay1 | -1.60094E-011 | 1.92916E-011 | .413 | -5.5355E-011 | 2.3336E-011 |
| | | PMHDay1 | -1.02581E-012 | 1.92916E-011 | .958 | -4.0371E-011 | 3.8320E-011 |
| | | PFHDay2 | -3.12474E-011 | 1.92916E-011 | .115 | -7.0593E-011 | 8.0981E-012 |
| | | BMHDay2 | -6.21085E-012 | 1.92916E-011 | .750 | -4.5556E-011 | 3.3135E-011 |
| | | PMHDay2 | -6.64372E-011* | 1.92916E-011 | .002 | -1.0578E-010 | -2.7092E-011 |
| | PFHDay2 | BFHDay1 | 1.66166E-011 | 1.81883E-011 | .368 | -2.0479E-011 | 5.3712E-011 |
| | | PFHDay1 | 1.83939E-011 | 1.81883E-011 | .320 | -1.8701E-011 | 5.5489E-011 |
| | | BMHDay1 | 1.52380E-011 | 1.81883E-011 | .409 | -2.1857E-011 | 5.2333E-011 |
| | | PMHDay1 | 3.02216E-011 | 1.81883E-011 | .107 | -6.8738E-012 | 6.7317E-011 |
| | | BFHDay2 | 3.12474E-011 | 1.92916E-011 | .115 | -8.0981E-012 | 7.0593E-011 |

| | | | | | | | |
|---|---|---|---|---|---|---|---|
| | | BMHDay2 | 2.50366E-011 | 1.81883E-011 | .179 | -1.2059E-011 | 6.2132E-011 |
| | | PMHDay2 | -3.51898E-011 | 1.81883E-011 | .062 | -7.2285E-011 | 1.9056E-012 |
| | BMHDay2 | BFHDay1 | -8.41998E-012 | 1.81883E-011 | .647 | -4.5515E-011 | 2.8675E-011 |
| | | PFHDay1 | -6.64268E-012 | 1.81883E-011 | .717 | -4.3738E-011 | 3.0453E-011 |
| | | BMHDay1 | -9.79852E-012 | 1.81883E-011 | .594 | -4.6894E-011 | 2.7297E-011 |
| | | PMHDay1 | 5.18504E-012 | 1.81883E-011 | .777 | -3.1910E-011 | 4.2280E-011 |
| | | BFHDay2 | 6.21085E-012 | 1.92916E-011 | .750 | -3.3135E-011 | 4.5556E-011 |
| | | PFHDay2 | -2.50366E-011 | 1.81883E-011 | .179 | -6.2132E-011 | 1.2059E-011 |
| | | PMHDay2 | -6.02264E-011* | 1.81883E-011 | .002 | -9.7322E-011 | -2.3131E-011 |
| | PMHDay2 | BFHDay1 | 5.18064E-011* | 1.81883E-011 | .008 | 1.4711E-011 | 8.8902E-011 |
| | | PFHDay1 | 5.35837E-011* | 1.81883E-011 | .006 | 1.6488E-011 | 9.0679E-011 |
| | | BMHDay1 | 5.04278E-011* | 1.81883E-011 | .009 | 1.3332E-011 | 8.7523E-011 |
| | | PMHDay1 | 6.54114E-011* | 1.81883E-011 | .001 | 2.8316E-011 | 1.0251E-010 |
| | | BFHDay2 | 6.64372E-011* | 1.92916E-011 | .002 | 2.7092E-011 | 1.0578E-010 |
| | | PFHDay2 | 3.51898E-011 | 1.81883E-011 | .062 | -1.9056E-012 | 7.2285E-011 |
| | | BMHDay2 | 6.02264E-011* | 1.81883E-011 | .002 | 2.3131E-011 | 9.7322E-011 |
| araGABA | BFHDay1 | PFHDay1 | -3.50214E-015 | 3.60719E-014 | .923 | -7.7794E-014 | 7.0789E-014 |
| | | BMHDay1 | 1.03726E-014 | 3.85625E-014 | .790 | -6.9048E-014 | 8.9794E-014 |
| | | PMHDay1 | -1.84510E-014 | 3.44914E-014 | .597 | -8.9487E-014 | 5.2585E-014 |

| | | | | | | |
|---|---|---|---|---|---|---|
| | BFHDay2 | 6.53363E-015 | 3.60719E-014 | .858 | -6.7758E-014 | 8.0825E-014 |
| | PFHDay2 | -4.08821E-014 | 3.44914E-014 | .247 | -1.1192E-013 | 3.0154E-014 |
| | BMHDay2 | 4.87277E-015 | 3.44914E-014 | .889 | -6.6164E-014 | 7.5909E-014 |
| | PMHDay2 | -3.55137E-014 | 3.60719E-014 | .334 | -1.0981E-013 | 3.8778E-014 |
| PFHDay1 | BFHDay1 | 3.50214E-015 | 3.60719E-014 | .923 | -7.0789E-014 | 7.7794E-014 |
| | BMHDay1 | 1.38748E-014 | 3.60719E-014 | .704 | -6.0417E-014 | 8.8166E-014 |
| | PMHDay1 | -1.49489E-014 | 3.16823E-014 | .641 | -8.0200E-014 | 5.0302E-014 |
| | BFHDay2 | 1.00358E-014 | 3.33961E-014 | .766 | -5.8745E-014 | 7.8816E-014 |
| | PFHDay2 | -3.73800E-014 | 3.16823E-014 | .249 | -1.0263E-013 | 2.7871E-014 |
| | BMHDay2 | 8.37492E-015 | 3.16823E-014 | .794 | -5.6876E-014 | 7.3626E-014 |
| | PMHDay2 | -3.20115E-014 | 3.33961E-014 | .347 | -1.0079E-013 | 3.6769E-014 |
| BMHDay1 | BFHDay1 | -1.03726E-014 | 3.85625E-014 | .790 | -8.9794E-014 | 6.9048E-014 |
| | PFHDay1 | -1.38748E-014 | 3.60719E-014 | .704 | -8.8166E-014 | 6.0417E-014 |
| | PMHDay1 | -2.88236E-014 | 3.44914E-014 | .411 | -9.9860E-014 | 4.2213E-014 |
| | BFHDay2 | -3.83901E-015 | 3.60719E-014 | .916 | -7.8131E-014 | 7.0453E-014 |
| | PFHDay2 | -5.12547E-014 | 3.44914E-014 | .150 | -1.2229E-013 | 1.9782E-014 |
| | BMHDay2 | -5.49986E-015 | 3.44914E-014 | .875 | -7.6536E-014 | 6.5536E-014 |
| | PMHDay2 | -4.58863E-014 | 3.60719E-014 | .215 | -1.2018E-013 | 2.8405E-014 |
| PMHDay1 | BFHDay1 | 1.84510E-014 | 3.44914E-014 | .597 | -5.2585E-014 | 8.9487E-014 |

| | | | | | | |
|---|---|---|---|---|---|---|
| | PFHDay1 | 1.49489E-014 | 3.16823E-014 | .641 | -5.0302E-014 | 8.0200E-014 |
| | BMHDay1 | 2.88236E-014 | 3.44914E-014 | .411 | -4.2213E-014 | 9.9860E-014 |
| | BFHDay2 | 2.49846E-014 | 3.16823E-014 | .438 | -4.0266E-014 | 9.0236E-014 |
| | PFHDay2 | -2.24311E-014 | 2.98704E-014 | .460 | -8.3950E-014 | 3.9088E-014 |
| | BMHDay2 | 2.33238E-014 | 2.98704E-014 | .442 | -3.8195E-014 | 8.4843E-014 |
| | PMHDay2 | -1.70627E-014 | 3.16823E-014 | .595 | -8.2314E-014 | 4.8188E-014 |
| BFHDay2 | BFHDay1 | -6.53363E-015 | 3.60719E-014 | .858 | -8.0825E-014 | 6.7758E-014 |
| | PFHDay1 | -1.00358E-014 | 3.33961E-014 | .766 | -7.8816E-014 | 5.8745E-014 |
| | BMHDay1 | 3.83901E-015 | 3.60719E-014 | .916 | -7.0453E-014 | 7.8131E-014 |
| | PMHDay1 | -2.49846E-014 | 3.16823E-014 | .438 | -9.0236E-014 | 4.0266E-014 |
| | PFHDay2 | -4.74157E-014 | 3.16823E-014 | .147 | -1.1267E-013 | 1.7835E-014 |
| | BMHDay2 | -1.66085E-015 | 3.16823E-014 | .959 | -6.6912E-014 | 6.3590E-014 |
| | PMHDay2 | -4.20473E-014 | 3.33961E-014 | .220 | -1.1083E-013 | 2.6733E-014 |
| PFHDay2 | BFHDay1 | 4.08821E-014 | 3.44914E-014 | .247 | -3.0154E-014 | 1.1192E-013 |
| | PFHDay1 | 3.73800E-014 | 3.16823E-014 | .249 | -2.7871E-014 | 1.0263E-013 |
| | BMHDay1 | 5.12547E-014 | 3.44914E-014 | .150 | -1.9782E-014 | 1.2229E-013 |
| | PMHDay1 | 2.24311E-014 | 2.98704E-014 | .460 | -3.9088E-014 | 8.3950E-014 |
| | BFHDay2 | 4.74157E-014 | 3.16823E-014 | .147 | -1.7835E-014 | 1.1267E-013 |
| | BMHDay2 | 4.57549E-014 | 2.98704E-014 | .138 | -1.5764E-014 | 1.0727E-013 |

| | | | | | | | |
|---|---|---|---|---|---|---|---|
| | | PMHDay2 | 5.36844E-015 | 3.16823E-014 | .867 | -5.9883E-014 | 7.0619E-014 |
| | BMHDay2 | BFHDay1 | -4.87277E-015 | 3.44914E-014 | .889 | -7.5909E-014 | 6.6164E-014 |
| | | PFHDay1 | -8.37492E-015 | 3.16823E-014 | .794 | -7.3626E-014 | 5.6876E-014 |
| | | BMHDay1 | 5.49986E-015 | 3.44914E-014 | .875 | -6.5536E-014 | 7.6536E-014 |
| | | PMHDay1 | -2.33238E-014 | 2.98704E-014 | .442 | -8.4843E-014 | 3.8195E-014 |
| | | BFHDay2 | 1.66085E-015 | 3.16823E-014 | .959 | -6.3590E-014 | 6.6912E-014 |
| | | PFHDay2 | -4.57549E-014 | 2.98704E-014 | .138 | -1.0727E-013 | 1.5764E-014 |
| | | PMHDay2 | -4.03864E-014 | 3.16823E-014 | .214 | -1.0564E-013 | 2.4865E-014 |
| | PMHDay2 | BFHDay1 | 3.55137E-014 | 3.60719E-014 | .334 | -3.8778E-014 | 1.0981E-013 |
| | | PFHDay1 | 3.20115E-014 | 3.33961E-014 | .347 | -3.6769E-014 | 1.0079E-013 |
| | | BMHDay1 | 4.58863E-014 | 3.60719E-014 | .215 | -2.8405E-014 | 1.2018E-013 |
| | | PMHDay1 | 1.70627E-014 | 3.16823E-014 | .595 | -4.8188E-014 | 8.2314E-014 |
| | | BFHDay2 | 4.20473E-014 | 3.33961E-014 | .220 | -2.6733E-014 | 1.1083E-013 |
| | | PFHDay2 | -5.36844E-015 | 3.16823E-014 | .867 | -7.0619E-014 | 5.9883E-014 |
| | | BMHDay2 | 4.03864E-014 | 3.16823E-014 | .214 | -2.4865E-014 | 1.0564E-013 |
| docGABA | BFHDay1 | PFHDay1 | 9.99880E-016 | 3.96876E-014 | .980 | -8.1535E-014 | 8.3535E-014 |
| | | BMHDay1 | -5.19031E-015 | 4.58273E-014 | .911 | -1.0049E-013 | 9.0113E-014 |
| | | PMHDay1 | -9.05315E-015 | 4.58273E-014 | .845 | -1.0436E-013 | 8.6250E-014 |
| | | BFHDay2 | -2.39849E-014 | 3.96876E-014 | .552 | -1.0652E-013 | 5.8550E-014 |

| | | | | | | |
|---|---|---|---|---|---|---|
| | PFHDay2 | -4.12147E-014 | 5.25017E-014 | .441 | -1.5040E-013 | 6.7969E-014 |
| | BMHDay2 | -2.99588E-014 | 4.58273E-014 | .520 | -1.2526E-013 | 6.5344E-014 |
| | PMHDay2 | 3.42958E-014 | 4.58273E-014 | .463 | -6.1007E-014 | 1.2960E-013 |
| PFHDay1 | BFHDay1 | -9.99880E-016 | 3.96876E-014 | .980 | -8.3535E-014 | 8.1535E-014 |
| | BMHDay1 | -6.19019E-015 | 4.58273E-014 | .894 | -1.0149E-013 | 8.9113E-014 |
| | PMHDay1 | -1.00530E-014 | 4.58273E-014 | .828 | -1.0536E-013 | 8.5250E-014 |
| | BFHDay2 | -2.49848E-014 | 3.96876E-014 | .536 | -1.0752E-013 | 5.7550E-014 |
| | PFHDay2 | -4.22146E-014 | 5.25017E-014 | .430 | -1.5140E-013 | 6.6969E-014 |
| | BMHDay2 | -3.09587E-014 | 4.58273E-014 | .507 | -1.2626E-013 | 6.4344E-014 |
| | PMHDay2 | 3.32959E-014 | 4.58273E-014 | .476 | -6.2007E-014 | 1.2860E-013 |
| BMHDay1 | BFHDay1 | 5.19031E-015 | 4.58273E-014 | .911 | -9.0113E-014 | 1.0049E-013 |
| | PFHDay1 | 6.19019E-015 | 4.58273E-014 | .894 | -8.9113E-014 | 1.0149E-013 |
| | PMHDay1 | -3.86283E-015 | 5.12365E-014 | .941 | -1.1041E-013 | 1.0269E-013 |
| | BFHDay2 | -1.87946E-014 | 4.58273E-014 | .686 | -1.1410E-013 | 7.6508E-014 |
| | PFHDay2 | -3.60244E-014 | 5.72841E-014 | .536 | -1.5515E-013 | 8.3104E-014 |
| | BMHDay2 | -2.47685E-014 | 5.12365E-014 | .634 | -1.3132E-013 | 8.1784E-014 |
| | PMHDay2 | 3.94861E-014 | 5.12365E-014 | .449 | -6.7066E-014 | 1.4604E-013 |
| PMHDay1 | BFHDay1 | 9.05315E-015 | 4.58273E-014 | .845 | -8.6250E-014 | 1.0436E-013 |
| | PFHDay1 | 1.00530E-014 | 4.58273E-014 | .828 | -8.5250E-014 | 1.0536E-013 |

| | | | | | | |
|---|---|---|---|---|---|---|
| | BMHDay1 | 3.86283E-015 | 5.12365E-014 | .941 | -1.0269E-013 | 1.1041E-013 |
| | BFHDay2 | -1.49318E-014 | 4.58273E-014 | .748 | -1.1023E-013 | 8.0371E-014 |
| | PFHDay2 | -3.21616E-014 | 5.72841E-014 | .580 | -1.5129E-013 | 8.6967E-014 |
| | BMHDay2 | -2.09057E-014 | 5.12365E-014 | .687 | -1.2746E-013 | 8.5646E-014 |
| | PMHDay2 | 4.33490E-014 | 5.12365E-014 | .407 | -6.3203E-014 | 1.4990E-013 |
| BFHDay2 | BFHDay1 | 2.39849E-014 | 3.96876E-014 | .552 | -5.8550E-014 | 1.0652E-013 |
| | PFHDay1 | 2.49848E-014 | 3.96876E-014 | .536 | -5.7550E-014 | 1.0752E-013 |
| | BMHDay1 | 1.87946E-014 | 4.58273E-014 | .686 | -7.6508E-014 | 1.1410E-013 |
| | PMHDay1 | 1.49318E-014 | 4.58273E-014 | .748 | -8.0371E-014 | 1.1023E-013 |
| | PFHDay2 | -1.72298E-014 | 5.25017E-014 | .746 | -1.2641E-013 | 9.1954E-014 |
| | BMHDay2 | -5.97391E-015 | 4.58273E-014 | .898 | -1.0128E-013 | 8.9329E-014 |
| | PMHDay2 | 5.82807E-014 | 4.58273E-014 | .217 | -3.7022E-014 | 1.5358E-013 |
| PFHDay2 | BFHDay1 | 4.12147E-014 | 5.25017E-014 | .441 | -6.7969E-014 | 1.5040E-013 |
| | PFHDay1 | 4.22146E-014 | 5.25017E-014 | .430 | -6.6969E-014 | 1.5140E-013 |
| | BMHDay1 | 3.60244E-014 | 5.72841E-014 | .536 | -8.3104E-014 | 1.5515E-013 |
| | PMHDay1 | 3.21616E-014 | 5.72841E-014 | .580 | -8.6967E-014 | 1.5129E-013 |
| | BFHDay2 | 1.72298E-014 | 5.25017E-014 | .746 | -9.1954E-014 | 1.2641E-013 |
| | BMHDay2 | 1.12559E-014 | 5.72841E-014 | .846 | -1.0787E-013 | 1.3038E-013 |
| | PMHDay2 | 7.55106E-014 | 5.72841E-014 | .202 | -4.3618E-014 | 1.9464E-013 |

| | | | | | | | |
|---|---|---|---|---|---|---|---|
| | BMHDay2 | BFHDay1 | 2.99588E-014 | 4.58273E-014 | .520 | -6.5344E-014 | 1.2526E-013 |
| | | PFHDay1 | 3.09587E-014 | 4.58273E-014 | .507 | -6.4344E-014 | 1.2626E-013 |
| | | BMHDay1 | 2.47685E-014 | 5.12365E-014 | .634 | -8.1784E-014 | 1.3132E-013 |
| | | PMHDay1 | 2.09057E-014 | 5.12365E-014 | .687 | -8.5646E-014 | 1.2746E-013 |
| | | BFHDay2 | 5.97391E-015 | 4.58273E-014 | .898 | -8.9329E-014 | 1.0128E-013 |
| | | PFHDay2 | -1.12559E-014 | 5.72841E-014 | .846 | -1.3038E-013 | 1.0787E-013 |
| | | PMHDay2 | 6.42546E-014 | 5.12365E-014 | .224 | -4.2297E-014 | 1.7081E-013 |
| | PMHDay2 | BFHDay1 | -3.42958E-014 | 4.58273E-014 | .463 | -1.2960E-013 | 6.1007E-014 |
| | | PFHDay1 | -3.32959E-014 | 4.58273E-014 | .476 | -1.2860E-013 | 6.2007E-014 |
| | | BMHDay1 | -3.94861E-014 | 5.12365E-014 | .449 | -1.4604E-013 | 6.7066E-014 |
| | | PMHDay1 | -4.33490E-014 | 5.12365E-014 | .407 | -1.4990E-013 | 6.3203E-014 |
| | | BFHDay2 | -5.82807E-014 | 4.58273E-014 | .217 | -1.5358E-013 | 3.7022E-014 |
| | | PFHDay2 | -7.55106E-014 | 5.72841E-014 | .202 | -1.9464E-013 | 4.3618E-014 |
| | | BMHDay2 | -6.42546E-014 | 5.12365E-014 | .224 | -1.7081E-013 | 4.2297E-014 |
| palMeth | BFHDay1 | PFHDay1 | -3.25620E-014 | 2.47357E-012 | .990 | -5.0711E-012 | 5.0059E-012 |
| | | BMHDay1 | 3.91082E-013 | 2.47357E-012 | .875 | -4.6474E-012 | 5.4296E-012 |
| | | PMHDay1 | -3.28709E-012 | 2.47357E-012 | .193 | -8.3256E-012 | 1.7514E-012 |
| | | BFHDay2 | 1.52378E-012 | 2.47357E-012 | .542 | -3.5147E-012 | 6.5623E-012 |
| | | PFHDay2 | -9.86338E-013 | 2.47357E-012 | .693 | -6.0248E-012 | 4.0522E-012 |

| | | | | | | |
|---|---|---|---|---|---|---|
| | BMHDay2 | -3.96483E-012 | 2.47357E-012 | .119 | -9.0033E-012 | 1.0737E-012 |
| | PMHDay2 | -5.12000E-012* | 2.47357E-012 | .047 | -1.0158E-011 | -8.1499E-014 |
| PFHDay1 | BFHDay1 | 3.25620E-014 | 2.47357E-012 | .990 | -5.0059E-012 | 5.0711E-012 |
| | BMHDay1 | 4.23644E-013 | 2.47357E-012 | .865 | -4.6149E-012 | 5.4621E-012 |
| | PMHDay1 | -3.25453E-012 | 2.47357E-012 | .198 | -8.2930E-012 | 1.7840E-012 |
| | BFHDay2 | 1.55634E-012 | 2.47357E-012 | .534 | -3.4822E-012 | 6.5948E-012 |
| | PFHDay2 | -9.53776E-013 | 2.47357E-012 | .702 | -5.9923E-012 | 4.0847E-012 |
| | BMHDay2 | -3.93226E-012 | 2.47357E-012 | .122 | -8.9708E-012 | 1.1062E-012 |
| | PMHDay2 | -5.08744E-012* | 2.47357E-012 | .048 | -1.0126E-011 | -4.8937E-014 |
| BMHDay1 | BFHDay1 | -3.91082E-013 | 2.47357E-012 | .875 | -5.4296E-012 | 4.6474E-012 |
| | PFHDay1 | -4.23644E-013 | 2.47357E-012 | .865 | -5.4621E-012 | 4.6149E-012 |
| | PMHDay1 | -3.67817E-012 | 2.47357E-012 | .147 | -8.7167E-012 | 1.3603E-012 |
| | BFHDay2 | 1.13270E-012 | 2.47357E-012 | .650 | -3.9058E-012 | 6.1712E-012 |
| | PFHDay2 | -1.37742E-012 | 2.47357E-012 | .582 | -6.4159E-012 | 3.6611E-012 |
| | BMHDay2 | -4.35591E-012 | 2.47357E-012 | .088 | -9.3944E-012 | 6.8259E-013 |
| | PMHDay2 | -5.51108E-012* | 2.47357E-012 | .033 | -1.0550E-011 | -4.7258E-013 |
| PMHDay1 | BFHDay1 | 3.28709E-012 | 2.47357E-012 | .193 | -1.7514E-012 | 8.3256E-012 |
| | PFHDay1 | 3.25453E-012 | 2.47357E-012 | .198 | -1.7840E-012 | 8.2930E-012 |
| | BMHDay1 | 3.67817E-012 | 2.47357E-012 | .147 | -1.3603E-012 | 8.7167E-012 |

| | | | | | | |
|---|---|---|---|---|---|---|
| | | BFHDay2 | 4.81087E-012 | 2.47357E-012 | .061 | -2.2763E-013 | 9.8494E-012 |
| | | PFHDay2 | 2.30075E-012 | 2.47357E-012 | .359 | -2.7377E-012 | 7.3393E-012 |
| | | BMHDay2 | -6.77734E-013 | 2.47357E-012 | .786 | -5.7162E-012 | 4.3608E-012 |
| | | PMHDay2 | -1.83291E-012 | 2.47357E-012 | .464 | -6.8714E-012 | 3.2056E-012 |
| | BFHDay2 | BFHDay1 | -1.52378E-012 | 2.47357E-012 | .542 | -6.5623E-012 | 3.5147E-012 |
| | | PFHDay1 | -1.55634E-012 | 2.47357E-012 | .534 | -6.5948E-012 | 3.4822E-012 |
| | | BMHDay1 | -1.13270E-012 | 2.47357E-012 | .650 | -6.1712E-012 | 3.9058E-012 |
| | | PMHDay1 | -4.81087E-012 | 2.47357E-012 | .061 | -9.8494E-012 | 2.2763E-013 |
| | | PFHDay2 | -2.51012E-012 | 2.47357E-012 | .318 | -7.5486E-012 | 2.5284E-012 |
| | | BMHDay2 | -5.48860E-012* | 2.47357E-012 | .034 | -1.0527E-011 | -4.5011E-013 |
| | | PMHDay2 | -6.64378E-012* | 2.47357E-012 | .011 | -1.1682E-011 | -1.6053E-012 |
| | PFHDay2 | BFHDay1 | 9.86338E-013 | 2.47357E-012 | .693 | -4.0522E-012 | 6.0248E-012 |
| | | PFHDay1 | 9.53776E-013 | 2.47357E-012 | .702 | -4.0847E-012 | 5.9923E-012 |
| | | BMHDay1 | 1.37742E-012 | 2.47357E-012 | .582 | -3.6611E-012 | 6.4159E-012 |
| | | PMHDay1 | -2.30075E-012 | 2.47357E-012 | .359 | -7.3393E-012 | 2.7377E-012 |
| | | BFHDay2 | 2.51012E-012 | 2.47357E-012 | .318 | -2.5284E-012 | 7.5486E-012 |
| | | BMHDay2 | -2.97849E-012 | 2.47357E-012 | .237 | -8.0170E-012 | 2.0600E-012 |
| | | PMHDay2 | -4.13366E-012 | 2.47357E-012 | .104 | -9.1722E-012 | 9.0484E-013 |
| | BMHDay2 | BFHDay1 | 3.96483E-012 | 2.47357E-012 | .119 | -1.0737E-012 | 9.0033E-012 |

| | | | | | | | |
|---|---|---|---|---|---|---|---|
| | | PFHDay1 | 3.93226E-012 | 2.47357E-012 | .122 | -1.1062E-012 | 8.9708E-012 |
| | | BMHDay1 | 4.35591E-012 | 2.47357E-012 | .088 | -6.8259E-013 | 9.3944E-012 |
| | | PMHDay1 | 6.77734E-013 | 2.47357E-012 | .786 | -4.3608E-012 | 5.7162E-012 |
| | | BFHDay2 | 5.48860E-012* | 2.47357E-012 | .034 | 4.5011E-013 | 1.0527E-011 |
| | | PFHDay2 | 2.97849E-012 | 2.47357E-012 | .237 | -2.0600E-012 | 8.0170E-012 |
| | | PMHDay2 | -1.15517E-012 | 2.47357E-012 | .644 | -6.1937E-012 | 3.8833E-012 |
| | PMHDay2 | BFHDay1 | 5.12000E-012* | 2.47357E-012 | .047 | 8.1499E-014 | 1.0158E-011 |
| | | PFHDay1 | 5.08744E-012* | 2.47357E-012 | .048 | 4.8937E-014 | 1.0126E-011 |
| | | BMHDay1 | 5.51108E-012* | 2.47357E-012 | .033 | 4.7258E-013 | 1.0550E-011 |
| | | PMHDay1 | 1.83291E-012 | 2.47357E-012 | .464 | -3.2056E-012 | 6.8714E-012 |
| | | BFHDay2 | 6.64378E-012* | 2.47357E-012 | .011 | 1.6053E-012 | 1.1682E-011 |
| | | PFHDay2 | 4.13366E-012 | 2.47357E-012 | .104 | -9.0484E-013 | 9.1722E-012 |
| | | BMHDay2 | 1.15517E-012 | 2.47357E-012 | .644 | -3.8833E-012 | 6.1937E-012 |
| steMeth | BFHDay1 | PFHDay1 | 2.84581E-013 | 5.65722E-013 | .619 | -8.7245E-013 | 1.4416E-012 |
| | | BMHDay1 | 4.47085E-013 | 5.65722E-013 | .436 | -7.0995E-013 | 1.6041E-012 |
| | | PMHDay1 | -1.26168E-012* | 5.65722E-013 | .034 | -2.4187E-012 | -1.0464E-013 |
| | | BFHDay2 | 3.15276E-013 | 5.96324E-013 | .601 | -9.0434E-013 | 1.5349E-012 |
| | | PFHDay2 | -5.27198E-013 | 5.96324E-013 | .384 | -1.7468E-012 | 6.9242E-013 |
| | | BMHDay2 | -2.20826E-013 | 5.65722E-013 | .699 | -1.3779E-012 | 9.3621E-013 |

| | | | | | | |
|---|---|---|---|---|---|---|
| | PMHDay2 | -7.21097E-013 | 5.65722E-013 | .213 | -1.8781E-012 | 4.3594E-013 |
| PFHDay1 | BFHDay1 | -2.84581E-013 | 5.65722E-013 | .619 | -1.4416E-012 | 8.7245E-013 |
| | BMHDay1 | 1.62504E-013 | 5.33368E-013 | .763 | -9.2836E-013 | 1.2534E-012 |
| | PMHDay1 | -1.54626E-012* | 5.33368E-013 | .007 | -2.6371E-012 | -4.5540E-013 |
| | BFHDay2 | 3.06949E-014 | 5.65722E-013 | .957 | -1.1263E-012 | 1.1877E-012 |
| | PFHDay2 | -8.11779E-013 | 5.65722E-013 | .162 | -1.9688E-012 | 3.4525E-013 |
| | BMHDay2 | -5.05406E-013 | 5.33368E-013 | .351 | -1.5963E-012 | 5.8545E-013 |
| | PMHDay2 | -1.00568E-012 | 5.33368E-013 | .069 | -2.0965E-012 | 8.5183E-014 |
| BMHDay1 | BFHDay1 | -4.47085E-013 | 5.65722E-013 | .436 | -1.6041E-012 | 7.0995E-013 |
| | PFHDay1 | -1.62504E-013 | 5.33368E-013 | .763 | -1.2534E-012 | 9.2836E-013 |
| | PMHDay1 | -1.70876E-012* | 5.33368E-013 | .003 | -2.7996E-012 | -6.1790E-013 |
| | BFHDay2 | -1.31809E-013 | 5.65722E-013 | .817 | -1.2888E-012 | 1.0252E-012 |
| | PFHDay2 | -9.74283E-013 | 5.65722E-013 | .096 | -2.1313E-012 | 1.8275E-013 |
| | BMHDay2 | -6.67911E-013 | 5.33368E-013 | .220 | -1.7588E-012 | 4.2295E-013 |
| | PMHDay2 | -1.16818E-012* | 5.33368E-013 | .037 | -2.2590E-012 | -7.7321E-014 |
| PMHDay1 | BFHDay1 | 1.26168E-012* | 5.65722E-013 | .034 | 1.0464E-013 | 2.4187E-012 |
| | PFHDay1 | 1.54626E-012* | 5.33368E-013 | .007 | 4.5540E-013 | 2.6371E-012 |
| | BMHDay1 | 1.70876E-012* | 5.33368E-013 | .003 | 6.1790E-013 | 2.7996E-012 |
| | BFHDay2 | 1.57695E-012* | 5.65722E-013 | .009 | 4.1992E-013 | 2.7340E-012 |

| | | | | | | |
|---|---|---|---|---|---|---|
| | PFHDay2 | 7.34478E-013 | 5.65722E-013 | .204 | -4.2255E-013 | 1.8915E-012 |
| | BMHDay2 | 1.04085E-012 | 5.33368E-013 | .061 | -5.0009E-014 | 2.1317E-012 |
| | PMHDay2 | 5.40580E-013 | 5.33368E-013 | .319 | -5.5028E-013 | 1.6314E-012 |
| BFHDay2 | BFHDay1 | -3.15276E-013 | 5.96324E-013 | .601 | -1.5349E-012 | 9.0434E-013 |
| | PFHDay1 | -3.06949E-014 | 5.65722E-013 | .957 | -1.1877E-012 | 1.1263E-012 |
| | BMHDay1 | 1.31809E-013 | 5.65722E-013 | .817 | -1.0252E-012 | 1.2888E-012 |
| | PMHDay1 | -1.57695E-012* | 5.65722E-013 | .009 | -2.7340E-012 | -4.1992E-013 |
| | PFHDay2 | -8.42474E-013 | 5.96324E-013 | .168 | -2.0621E-012 | 3.7715E-013 |
| | BMHDay2 | -5.36101E-013 | 5.65722E-013 | .351 | -1.6931E-012 | 6.2093E-013 |
| | PMHDay2 | -1.03637E-012 | 5.65722E-013 | .077 | -2.1934E-012 | 1.2066E-013 |
| PFHDay2 | BFHDay1 | 5.27198E-013 | 5.96324E-013 | .384 | -6.9242E-013 | 1.7468E-012 |
| | PFHDay1 | 8.11779E-013 | 5.65722E-013 | .162 | -3.4525E-013 | 1.9688E-012 |
| | BMHDay1 | 9.74283E-013 | 5.65722E-013 | .096 | -1.8275E-013 | 2.1313E-012 |
| | PMHDay1 | -7.34478E-013 | 5.65722E-013 | .204 | -1.8915E-012 | 4.2255E-013 |
| | BFHDay2 | 8.42474E-013 | 5.96324E-013 | .168 | -3.7715E-013 | 2.0621E-012 |
| | BMHDay2 | 3.06373E-013 | 5.65722E-013 | .592 | -8.5066E-013 | 1.4634E-012 |
| | PMHDay2 | -1.93898E-013 | 5.65722E-013 | .734 | -1.3509E-012 | 9.6313E-013 |
| BMHDay2 | BFHDay1 | 2.20826E-013 | 5.65722E-013 | .699 | -9.3621E-013 | 1.3779E-012 |
| | PFHDay1 | 5.05406E-013 | 5.33368E-013 | .351 | -5.8545E-013 | 1.5963E-012 |

| | | | | | | | |
|---|---|---|---|---|---|---|---|
| | | BMHDay1 | 6.67911E-013 | 5.33368E-013 | .220 | -4.2295E-013 | 1.7588E-012 |
| | | PMHDay1 | -1.04085E-012 | 5.33368E-013 | .061 | -2.1317E-012 | 5.0009E-014 |
| | | BFHDay2 | 5.36101E-013 | 5.65722E-013 | .351 | -6.2093E-013 | 1.6931E-012 |
| | | PFHDay2 | -3.06373E-013 | 5.65722E-013 | .592 | -1.4634E-012 | 8.5066E-013 |
| | | PMHDay2 | -5.00271E-013 | 5.33368E-013 | .356 | -1.5911E-012 | 5.9059E-013 |
| | PMHDay2 | BFHDay1 | 7.21097E-013 | 5.65722E-013 | .213 | -4.3594E-013 | 1.8781E-012 |
| | | PFHDay1 | 1.00568E-012 | 5.33368E-013 | .069 | -8.5183E-014 | 2.0965E-012 |
| | | BMHDay1 | 1.16818E-012* | 5.33368E-013 | .037 | 7.7321E-014 | 2.2590E-012 |
| | | PMHDay1 | -5.40580E-013 | 5.33368E-013 | .319 | -1.6314E-012 | 5.5028E-013 |
| | | BFHDay2 | 1.03637E-012 | 5.65722E-013 | .077 | -1.2066E-013 | 2.1934E-012 |
| | | PFHDay2 | 1.93898E-013 | 5.65722E-013 | .734 | -9.6313E-013 | 1.3509E-012 |
| | | BMHDay2 | 5.00271E-013 | 5.33368E-013 | .356 | -5.9059E-013 | 1.5911E-012 |
| oleMeth | BFHDay1 | PFHDay1 | 2.03725E-012 | 2.83622E-012 | .478 | -3.7399E-012 | 7.8144E-012 |
| | | BMHDay1 | 2.14522E-012 | 2.83622E-012 | .455 | -3.6320E-012 | 7.9224E-012 |
| | | PMHDay1 | -2.25629E-012 | 2.83622E-012 | .432 | -8.0335E-012 | 3.5209E-012 |
| | | BFHDay2 | 2.46255E-012 | 2.83622E-012 | .392 | -3.3146E-012 | 8.2397E-012 |
| | | PFHDay2 | 1.70317E-012 | 2.83622E-012 | .552 | -4.0740E-012 | 7.4804E-012 |
| | | BMHDay2 | -8.59226E-013 | 2.83622E-012 | .764 | -6.6364E-012 | 4.9180E-012 |
| | | PMHDay2 | -8.34833E-012* | 2.83622E-012 | .006 | -1.4126E-011 | -2.5711E-012 |

| | | | | | | |
|---|---|---|---|---|---|---|
| | PFHDay1 | BFHDay1 | -2.03725E-012 | 2.83622E-012 | .478 | -7.8144E-012 | 3.7399E-012 |
| | | BMHDay1 | 1.07976E-013 | 2.83622E-012 | .970 | -5.6692E-012 | 5.8852E-012 |
| | | PMHDay1 | -4.29354E-012 | 2.83622E-012 | .140 | -1.0071E-011 | 1.4836E-012 |
| | | BFHDay2 | 4.25306E-013 | 2.83622E-012 | .882 | -5.3519E-012 | 6.2025E-012 |
| | | PFHDay2 | -3.34080E-013 | 2.83622E-012 | .907 | -6.1113E-012 | 5.4431E-012 |
| | | BMHDay2 | -2.89647E-012 | 2.83622E-012 | .315 | -8.6737E-012 | 2.8807E-012 |
| | | PMHDay2 | -1.03856E-011* | 2.83622E-012 | <.001 | -1.6163E-011 | -4.6084E-012 |
| | BMHDay1 | BFHDay1 | -2.14522E-012 | 2.83622E-012 | .455 | -7.9224E-012 | 3.6320E-012 |
| | | PFHDay1 | -1.07976E-013 | 2.83622E-012 | .970 | -5.8852E-012 | 5.6692E-012 |
| | | PMHDay1 | -4.40152E-012 | 2.83622E-012 | .131 | -1.0179E-011 | 1.3757E-012 |
| | | BFHDay2 | 3.17330E-013 | 2.83622E-012 | .912 | -5.4599E-012 | 6.0945E-012 |
| | | PFHDay2 | -4.42056E-013 | 2.83622E-012 | .877 | -6.2192E-012 | 5.3351E-012 |
| | | BMHDay2 | -3.00445E-012 | 2.83622E-012 | .297 | -8.7816E-012 | 2.7727E-012 |
| | | PMHDay2 | -1.04936E-011* | 2.83622E-012 | <.001 | -1.6271E-011 | -4.7164E-012 |
| | PMHDay1 | BFHDay1 | 2.25629E-012 | 2.83622E-012 | .432 | -3.5209E-012 | 8.0335E-012 |
| | | PFHDay1 | 4.29354E-012 | 2.83622E-012 | .140 | -1.4836E-012 | 1.0071E-011 |
| | | BMHDay1 | 4.40152E-012 | 2.83622E-012 | .131 | -1.3757E-012 | 1.0179E-011 |
| | | BFHDay2 | 4.71885E-012 | 2.83622E-012 | .106 | -1.0583E-012 | 1.0496E-011 |
| | | PFHDay2 | 3.95946E-012 | 2.83622E-012 | .172 | -1.8177E-012 | 9.7367E-012 |

| | | | | | | |
|---|---|---|---|---|---|---|
| | BMHDay2 | 1.39707E-012 | 2.83622E-012 | .626 | -4.3801E-012 | 7.1743E-012 |
| | PMHDay2 | -6.09204E-012* | 2.83622E-012 | .039 | -1.1869E-011 | -3.1484E-013 |
| BFHDay2 | BFHDay1 | -2.46255E-012 | 2.83622E-012 | .392 | -8.2397E-012 | 3.3146E-012 |
| | PFHDay1 | -4.25306E-013 | 2.83622E-012 | .882 | -6.2025E-012 | 5.3519E-012 |
| | BMHDay1 | -3.17330E-013 | 2.83622E-012 | .912 | -6.0945E-012 | 5.4599E-012 |
| | PMHDay1 | -4.71885E-012 | 2.83622E-012 | .106 | -1.0496E-011 | 1.0583E-012 |
| | PFHDay2 | -7.59386E-013 | 2.83622E-012 | .791 | -6.5366E-012 | 5.0178E-012 |
| | BMHDay2 | -3.32178E-012 | 2.83622E-012 | .250 | -9.0990E-012 | 2.4554E-012 |
| | PMHDay2 | -1.08109E-011* | 2.83622E-012 | <.001 | -1.6588E-011 | -5.0337E-012 |
| PFHDay2 | BFHDay1 | -1.70317E-012 | 2.83622E-012 | .552 | -7.4804E-012 | 4.0740E-012 |
| | PFHDay1 | 3.34080E-013 | 2.83622E-012 | .907 | -5.4431E-012 | 6.1113E-012 |
| | BMHDay1 | 4.42056E-013 | 2.83622E-012 | .877 | -5.3351E-012 | 6.2192E-012 |
| | PMHDay1 | -3.95946E-012 | 2.83622E-012 | .172 | -9.7367E-012 | 1.8177E-012 |
| | BFHDay2 | 7.59386E-013 | 2.83622E-012 | .791 | -5.0178E-012 | 6.5366E-012 |
| | BMHDay2 | -2.56239E-012 | 2.83622E-012 | .373 | -8.3396E-012 | 3.2148E-012 |
| | PMHDay2 | -1.00515E-011* | 2.83622E-012 | .001 | -1.5829E-011 | -4.2743E-012 |
| BMHDay2 | BFHDay1 | 8.59226E-013 | 2.83622E-012 | .764 | -4.9180E-012 | 6.6364E-012 |
| | PFHDay1 | 2.89647E-012 | 2.83622E-012 | .315 | -2.8807E-012 | 8.6737E-012 |
| | BMHDay1 | 3.00445E-012 | 2.83622E-012 | .297 | -2.7727E-012 | 8.7816E-012 |

| | | | | | | | |
|---|---|---|---|---|---|---|---|
| | | PMHDay1 | -1.39707E-012 | 2.83622E-012 | .626 | -7.1743E-012 | 4.3801E-012 |
| | | BFHDay2 | 3.32178E-012 | 2.83622E-012 | .250 | -2.4554E-012 | 9.0990E-012 |
| | | PFHDay2 | 2.56239E-012 | 2.83622E-012 | .373 | -3.2148E-012 | 8.3396E-012 |
| | | PMHDay2 | -7.48910E-012* | 2.83622E-012 | .013 | -1.3266E-011 | -1.7119E-012 |
| | PMHDay2 | BFHDay1 | 8.34833E-012* | 2.83622E-012 | .006 | 2.5711E-012 | 1.4126E-011 |
| | | PFHDay1 | 1.03856E-011* | 2.83622E-012 | <.001 | 4.6084E-012 | 1.6163E-011 |
| | | BMHDay1 | 1.04936E-011* | 2.83622E-012 | <.001 | 4.7164E-012 | 1.6271E-011 |
| | | PMHDay1 | 6.09204E-012* | 2.83622E-012 | .039 | 3.1484E-013 | 1.1869E-011 |
| | | BFHDay2 | 1.08109E-011* | 2.83622E-012 | <.001 | 5.0337E-012 | 1.6588E-011 |
| | | PFHDay2 | 1.00515E-011* | 2.83622E-012 | .001 | 4.2743E-012 | 1.5829E-011 |
| | | BMHDay2 | 7.48910E-012* | 2.83622E-012 | .013 | 1.7119E-012 | 1.3266E-011 |
| linMeth | BFHDay1 | PFHDay1 | 1.01475E-012 | 3.37451E-012 | .766 | -5.8676E-012 | 7.8971E-012 |
| | | BMHDay1 | 2.59902E-012 | 3.37451E-012 | .447 | -4.2833E-012 | 9.4814E-012 |
| | | PMHDay1 | -3.24091E-012 | 3.37451E-012 | .344 | -1.0123E-011 | 3.6415E-012 |
| | | BFHDay2 | 1.08242E-012 | 3.37451E-012 | .751 | -5.7999E-012 | 7.9648E-012 |
| | | PFHDay2 | 1.07127E-012 | 3.37451E-012 | .753 | -5.8111E-012 | 7.9536E-012 |
| | | BMHDay2 | -4.64597E-012 | 3.37451E-012 | .178 | -1.1528E-011 | 2.2364E-012 |
| | | PMHDay2 | -1.31538E-011* | 3.37451E-012 | <.001 | -2.0036E-011 | -6.2714E-012 |
| | PFHDay1 | BFHDay1 | -1.01475E-012 | 3.37451E-012 | .766 | -7.8971E-012 | 5.8676E-012 |

| | | | | | | |
|---|---|---|---|---|---|---|
| | BMHDay1 | 1.58427E-012 | 3.18152E-012 | .622 | -4.9045E-012 | 8.0730E-012 |
| | PMHDay1 | -4.25566E-012 | 3.18152E-012 | .191 | -1.0744E-011 | 2.2331E-012 |
| | BFHDay2 | 6.76700E-014 | 3.18152E-012 | .983 | -6.4211E-012 | 6.5564E-012 |
| | PFHDay2 | 5.65180E-014 | 3.18152E-012 | .986 | -6.4322E-012 | 6.5453E-012 |
| | BMHDay2 | -5.66072E-012 | 3.18152E-012 | .085 | -1.2149E-011 | 8.2804E-013 |
| | PMHDay2 | -1.41685E-011* | 3.18152E-012 | <.001 | -2.0657E-011 | -7.6798E-012 |
| BMHDay1 | BFHDay1 | -2.59902E-012 | 3.37451E-012 | .447 | -9.4814E-012 | 4.2833E-012 |
| | PFHDay1 | -1.58427E-012 | 3.18152E-012 | .622 | -8.0730E-012 | 4.9045E-012 |
| | PMHDay1 | -5.83993E-012 | 3.18152E-012 | .076 | -1.2329E-011 | 6.4883E-013 |
| | BFHDay2 | -1.51660E-012 | 3.18152E-012 | .637 | -8.0054E-012 | 4.9722E-012 |
| | PFHDay2 | -1.52775E-012 | 3.18152E-012 | .634 | -8.0165E-012 | 4.9610E-012 |
| | BMHDay2 | -7.24499E-012* | 3.18152E-012 | .030 | -1.3734E-011 | -7.5623E-013 |
| | PMHDay2 | -1.57528E-011* | 3.18152E-012 | <.001 | -2.2242E-011 | -9.2640E-012 |
| PMHDay1 | BFHDay1 | 3.24091E-012 | 3.37451E-012 | .344 | -3.6415E-012 | 1.0123E-011 |
| | PFHDay1 | 4.25566E-012 | 3.18152E-012 | .191 | -2.2331E-012 | 1.0744E-011 |
| | BMHDay1 | 5.83993E-012 | 3.18152E-012 | .076 | -6.4883E-013 | 1.2329E-011 |
| | BFHDay2 | 4.32333E-012 | 3.18152E-012 | .184 | -2.1654E-012 | 1.0812E-011 |
| | PFHDay2 | 4.31218E-012 | 3.18152E-012 | .185 | -2.1766E-012 | 1.0801E-011 |
| | BMHDay2 | -1.40506E-012 | 3.18152E-012 | .662 | -7.8938E-012 | 5.0837E-012 |

| | | | | | | |
|---|---|---|---|---|---|---|
| | PMHDay2 | -9.91287E-012* | 3.18152E-012 | .004 | -1.6402E-011 | -3.4241E-012 |
| BFHDay2 | BFHDay1 | -1.08242E-012 | 3.37451E-012 | .751 | -7.9648E-012 | 5.7999E-012 |
| | PFHDay1 | -6.76700E-014 | 3.18152E-012 | .983 | -6.5564E-012 | 6.4211E-012 |
| | BMHDay1 | 1.51660E-012 | 3.18152E-012 | .637 | -4.9722E-012 | 8.0054E-012 |
| | PMHDay1 | -4.32333E-012 | 3.18152E-012 | .184 | -1.0812E-011 | 2.1654E-012 |
| | PFHDay2 | -1.11520E-014 | 3.18152E-012 | .997 | -6.4999E-012 | 6.4776E-012 |
| | BMHDay2 | -5.72839E-012 | 3.18152E-012 | .082 | -1.2217E-011 | 7.6037E-013 |
| | PMHDay2 | -1.42362E-011* | 3.18152E-012 | <.001 | -2.0725E-011 | -7.7474E-012 |
| PFHDay2 | BFHDay1 | -1.07127E-012 | 3.37451E-012 | .753 | -7.9536E-012 | 5.8111E-012 |
| | PFHDay1 | -5.65180E-014 | 3.18152E-012 | .986 | -6.5453E-012 | 6.4322E-012 |
| | BMHDay1 | 1.52775E-012 | 3.18152E-012 | .634 | -4.9610E-012 | 8.0165E-012 |
| | PMHDay1 | -4.31218E-012 | 3.18152E-012 | .185 | -1.0801E-011 | 2.1766E-012 |
| | BFHDay2 | 1.11520E-014 | 3.18152E-012 | .997 | -6.4776E-012 | 6.4999E-012 |
| | BMHDay2 | -5.71724E-012 | 3.18152E-012 | .082 | -1.2206E-011 | 7.7152E-013 |
| | PMHDay2 | -1.42250E-011* | 3.18152E-012 | <.001 | -2.0714E-011 | -7.7363E-012 |
| BMHDay2 | BFHDay1 | 4.64597E-012 | 3.37451E-012 | .178 | -2.2364E-012 | 1.1528E-011 |
| | PFHDay1 | 5.66072E-012 | 3.18152E-012 | .085 | -8.2804E-013 | 1.2149E-011 |
| | BMHDay1 | 7.24499E-012* | 3.18152E-012 | .030 | 7.5623E-013 | 1.3734E-011 |
| | PMHDay1 | 1.40506E-012 | 3.18152E-012 | .662 | -5.0837E-012 | 7.8938E-012 |

| | | | | | | | |
|---|---|---|---|---|---|---|---|
| | | BFHDay2 | 5.72839E-012 | 3.18152E-012 | .082 | -7.6037E-013 | 1.2217E-011 |
| | | PFHDay2 | 5.71724E-012 | 3.18152E-012 | .082 | -7.7152E-013 | 1.2206E-011 |
| | | PMHDay2 | -8.50781E-012* | 3.18152E-012 | .012 | -1.4997E-011 | -2.0190E-012 |
| | PMHDay2 | BFHDay1 | 1.31538E-011* | 3.37451E-012 | <.001 | 6.2714E-012 | 2.0036E-011 |
| | | PFHDay1 | 1.41685E-011* | 3.18152E-012 | <.001 | 7.6798E-012 | 2.0657E-011 |
| | | BMHDay1 | 1.57528E-011* | 3.18152E-012 | <.001 | 9.2640E-012 | 2.2242E-011 |
| | | PMHDay1 | 9.91287E-012* | 3.18152E-012 | .004 | 3.4241E-012 | 1.6402E-011 |
| | | BFHDay2 | 1.42362E-011* | 3.18152E-012 | <.001 | 7.7474E-012 | 2.0725E-011 |
| | | PFHDay2 | 1.42250E-011* | 3.18152E-012 | <.001 | 7.7363E-012 | 2.0714E-011 |
| | | BMHDay2 | 8.50781E-012* | 3.18152E-012 | .012 | 2.0190E-012 | 1.4997E-011 |
| araMeth | BFHDay1 | PFHDay1 | -1.90152E-013 | 6.67985E-013 | .778 | -1.5720E-012 | 1.1917E-012 |
| | | BMHDay1 | 9.96542E-015 | 6.67985E-013 | .988 | -1.3719E-012 | 1.3918E-012 |
| | | PMHDay1 | -1.81351E-014 | 6.67985E-013 | .979 | -1.4000E-012 | 1.3637E-012 |
| | | BFHDay2 | -3.54987E-013 | 6.67985E-013 | .600 | -1.7368E-012 | 1.0268E-012 |
| | | PFHDay2 | -7.77097E-013 | 6.67985E-013 | .257 | -2.1589E-012 | 6.0473E-013 |
| | | BMHDay2 | 1.26044E-013 | 6.38715E-013 | .845 | -1.1952E-012 | 1.4473E-012 |
| | | PMHDay2 | -1.94598E-013 | 7.14106E-013 | .788 | -1.6718E-012 | 1.2826E-012 |
| | PFHDay1 | BFHDay1 | 1.90152E-013 | 6.67985E-013 | .778 | -1.1917E-012 | 1.5720E-012 |
| | | BMHDay1 | 2.00117E-013 | 6.18434E-013 | .749 | -1.0792E-012 | 1.4794E-012 |

| | | | | | | |
|---|---|---|---|---|---|---|
| | PMHDay1 | 1.72017E-013 | 6.18434E-013 | .783 | -1.1073E-012 | 1.4513E-012 |
| | BFHDay2 | -1.64835E-013 | 6.18434E-013 | .792 | -1.4442E-012 | 1.1145E-012 |
| | PFHDay2 | -5.86945E-013 | 6.18434E-013 | .352 | -1.8663E-012 | 6.9238E-013 |
| | BMHDay2 | 3.16196E-013 | 5.86698E-013 | .595 | -8.9748E-013 | 1.5299E-012 |
| | PMHDay2 | -4.44592E-015 | 6.67985E-013 | .995 | -1.3863E-012 | 1.3774E-012 |
| BMHDay1 | BFHDay1 | -9.96542E-015 | 6.67985E-013 | .988 | -1.3918E-012 | 1.3719E-012 |
| | PFHDay1 | -2.00117E-013 | 6.18434E-013 | .749 | -1.4794E-012 | 1.0792E-012 |
| | PMHDay1 | -2.81005E-014 | 6.18434E-013 | .964 | -1.3074E-012 | 1.2512E-012 |
| | BFHDay2 | -3.64952E-013 | 6.18434E-013 | .561 | -1.6443E-012 | 9.1438E-013 |
| | PFHDay2 | -7.87062E-013 | 6.18434E-013 | .216 | -2.0664E-012 | 4.9227E-013 |
| | BMHDay2 | 1.16079E-013 | 5.86698E-013 | .845 | -1.0976E-012 | 1.3298E-012 |
| | PMHDay2 | -2.04563E-013 | 6.67985E-013 | .762 | -1.5864E-012 | 1.1773E-012 |
| PMHDay1 | BFHDay1 | 1.81351E-014 | 6.67985E-013 | .979 | -1.3637E-012 | 1.4000E-012 |
| | PFHDay1 | -1.72017E-013 | 6.18434E-013 | .783 | -1.4513E-012 | 1.1073E-012 |
| | BMHDay1 | 2.81005E-014 | 6.18434E-013 | .964 | -1.2512E-012 | 1.3074E-012 |
| | BFHDay2 | -3.36852E-013 | 6.18434E-013 | .591 | -1.6162E-012 | 9.4248E-013 |
| | PFHDay2 | -7.58962E-013 | 6.18434E-013 | .232 | -2.0383E-012 | 5.2037E-013 |
| | BMHDay2 | 1.44179E-013 | 5.86698E-013 | .808 | -1.0695E-012 | 1.3579E-012 |
| | PMHDay2 | -1.76463E-013 | 6.67985E-013 | .794 | -1.5583E-012 | 1.2054E-012 |

| | | | | | | |
|---|---|---|---|---|---|---|
| BFHDay2 | BFHDay1 | 3.54987E-013 | 6.67985E-013 | .600 | -1.0268E-012 | 1.7368E-012 |
| | PFHDay1 | 1.64835E-013 | 6.18434E-013 | .792 | -1.1145E-012 | 1.4442E-012 |
| | BMHDay1 | 3.64952E-013 | 6.18434E-013 | .561 | -9.1438E-013 | 1.6443E-012 |
| | PMHDay1 | 3.36852E-013 | 6.18434E-013 | .591 | -9.4248E-013 | 1.6162E-012 |
| | PFHDay2 | -4.22110E-013 | 6.18434E-013 | .502 | -1.7014E-012 | 8.5722E-013 |
| | BMHDay2 | 4.81031E-013 | 5.86698E-013 | .421 | -7.3265E-013 | 1.6947E-012 |
| | PMHDay2 | 1.60389E-013 | 6.67985E-013 | .812 | -1.2214E-012 | 1.5422E-012 |
| PFHDay2 | BFHDay1 | 7.77097E-013 | 6.67985E-013 | .257 | -6.0473E-013 | 2.1589E-012 |
| | PFHDay1 | 5.86945E-013 | 6.18434E-013 | .352 | -6.9238E-013 | 1.8663E-012 |
| | BMHDay1 | 7.87062E-013 | 6.18434E-013 | .216 | -4.9227E-013 | 2.0664E-012 |
| | PMHDay1 | 7.58962E-013 | 6.18434E-013 | .232 | -5.2037E-013 | 2.0383E-012 |
| | BFHDay2 | 4.22110E-013 | 6.18434E-013 | .502 | -8.5722E-013 | 1.7014E-012 |
| | BMHDay2 | 9.03141E-013 | 5.86698E-013 | .137 | -3.1054E-013 | 2.1168E-012 |
| | PMHDay2 | 5.82499E-013 | 6.67985E-013 | .392 | -7.9933E-013 | 1.9643E-012 |
| BMHDay2 | BFHDay1 | -1.26044E-013 | 6.38715E-013 | .845 | -1.4473E-012 | 1.1952E-012 |
| | PFHDay1 | -3.16196E-013 | 5.86698E-013 | .595 | -1.5299E-012 | 8.9748E-013 |
| | BMHDay1 | -1.16079E-013 | 5.86698E-013 | .845 | -1.3298E-012 | 1.0976E-012 |
| | PMHDay1 | -1.44179E-013 | 5.86698E-013 | .808 | -1.3579E-012 | 1.0695E-012 |
| | BFHDay2 | -4.81031E-013 | 5.86698E-013 | .421 | -1.6947E-012 | 7.3265E-013 |

| | | | | | | | |
|---|---|---|---|---|---|---|---|
| | | PFHDay2 | -9.03141E-013 | 5.86698E-013 | .137 | -2.1168E-012 | 3.1054E-013 |
| | | PMHDay2 | -3.20642E-013 | 6.38715E-013 | .620 | -1.6419E-012 | 1.0006E-012 |
| | PMHDay2 | BFHDay1 | 1.94598E-013 | 7.14106E-013 | .788 | -1.2826E-012 | 1.6718E-012 |
| | | PFHDay1 | 4.44592E-015 | 6.67985E-013 | .995 | -1.3774E-012 | 1.3863E-012 |
| | | BMHDay1 | 2.04563E-013 | 6.67985E-013 | .762 | -1.1773E-012 | 1.5864E-012 |
| | | PMHDay1 | 1.76463E-013 | 6.67985E-013 | .794 | -1.2054E-012 | 1.5583E-012 |
| | | BFHDay2 | -1.60389E-013 | 6.67985E-013 | .812 | -1.5422E-012 | 1.2214E-012 |
| | | PFHDay2 | -5.82499E-013 | 6.67985E-013 | .392 | -1.9643E-012 | 7.9933E-013 |
| | | BMHDay2 | 3.20642E-013 | 6.38715E-013 | .620 | -1.0006E-012 | 1.6419E-012 |
| docMeth | BFHDay1 | PFHDay1 | 3.51666E-013 | 3.59073E-013 | .336 | -3.8386E-013 | 1.0872E-012 |
| | | BMHDay1 | 7.17354E-013 | 3.59073E-013 | .056 | -1.8173E-014 | 1.4529E-012 |
| | | PMHDay1 | 4.05005E-013 | 3.59073E-013 | .269 | -3.3052E-013 | 1.1405E-012 |
| | | BFHDay2 | 9.05079E-013* | 3.40646E-013 | .013 | 2.0730E-013 | 1.6029E-012 |
| | | PFHDay2 | 6.27496E-013 | 3.40646E-013 | .076 | -7.0286E-014 | 1.3253E-012 |
| | | BMHDay2 | 5.28210E-013 | 3.40646E-013 | .132 | -1.6957E-013 | 1.2260E-012 |
| | | PMHDay2 | 3.21708E-013 | 3.40646E-013 | .353 | -3.7607E-013 | 1.0195E-012 |
| | PFHDay1 | BFHDay1 | -3.51666E-013 | 3.59073E-013 | .336 | -1.0872E-012 | 3.8386E-013 |
| | | BMHDay1 | 3.65688E-013 | 3.59073E-013 | .317 | -3.6984E-013 | 1.1012E-012 |
| | | PMHDay1 | 5.33385E-014 | 3.59073E-013 | .883 | -6.8219E-013 | 7.8887E-013 |

| | | | | | | |
|---|---|---|---|---|---|---|
| | BFHDay2 | 5.53413E-013 | 3.40646E-013 | .115 | -1.4437E-013 | 1.2512E-012 |
| | PFHDay2 | 2.75830E-013 | 3.40646E-013 | .425 | -4.2195E-013 | 9.7361E-013 |
| | BMHDay2 | 1.76544E-013 | 3.40646E-013 | .608 | -5.2124E-013 | 8.7433E-013 |
| | PMHDay2 | -2.99583E-014 | 3.40646E-013 | .931 | -7.2774E-013 | 6.6782E-013 |
| BMHDay1 | BFHDay1 | -7.17354E-013 | 3.59073E-013 | .056 | -1.4529E-012 | 1.8173E-014 |
| | PFHDay1 | -3.65688E-013 | 3.59073E-013 | .317 | -1.1012E-012 | 3.6984E-013 |
| | PMHDay1 | -3.12350E-013 | 3.59073E-013 | .392 | -1.0479E-012 | 4.2318E-013 |
| | BFHDay2 | 1.87725E-013 | 3.40646E-013 | .586 | -5.1006E-013 | 8.8551E-013 |
| | PFHDay2 | -8.98585E-014 | 3.40646E-013 | .794 | -7.8764E-013 | 6.0792E-013 |
| | BMHDay2 | -1.89144E-013 | 3.40646E-013 | .583 | -8.8693E-013 | 5.0864E-013 |
| | PMHDay2 | -3.95646E-013 | 3.40646E-013 | .255 | -1.0934E-012 | 3.0214E-013 |
| PMHDay1 | BFHDay1 | -4.05005E-013 | 3.59073E-013 | .269 | -1.1405E-012 | 3.3052E-013 |
| | PFHDay1 | -5.33385E-014 | 3.59073E-013 | .883 | -7.8887E-013 | 6.8219E-013 |
| | BMHDay1 | 3.12350E-013 | 3.59073E-013 | .392 | -4.2318E-013 | 1.0479E-012 |
| | BFHDay2 | 5.00075E-013 | 3.40646E-013 | .153 | -1.9771E-013 | 1.1979E-012 |
| | PFHDay2 | 2.22491E-013 | 3.40646E-013 | .519 | -4.7529E-013 | 9.2027E-013 |
| | BMHDay2 | 1.23206E-013 | 3.40646E-013 | .720 | -5.7458E-013 | 8.2099E-013 |
| | PMHDay2 | -8.32968E-014 | 3.40646E-013 | .809 | -7.8108E-013 | 6.1449E-013 |
| BFHDay2 | BFHDay1 | -9.05079E-013* | 3.40646E-013 | .013 | -1.6029E-012 | -2.0730E-013 |

| | | | | | | |
|---|---|---|---|---|---|---|
| | | PFHDay1 | -5.53413E-013 | 3.40646E-013 | .115 | -1.2512E-012 | 1.4437E-013 |
| | | BMHDay1 | -1.87725E-013 | 3.40646E-013 | .586 | -8.8551E-013 | 5.1006E-013 |
| | | PMHDay1 | -5.00075E-013 | 3.40646E-013 | .153 | -1.1979E-012 | 1.9771E-013 |
| | | PFHDay2 | -2.77584E-013 | 3.21164E-013 | .395 | -9.3546E-013 | 3.8029E-013 |
| | | BMHDay2 | -3.76869E-013 | 3.21164E-013 | .251 | -1.0347E-012 | 2.8101E-013 |
| | | PMHDay2 | -5.83371E-013 | 3.21164E-013 | .080 | -1.2412E-012 | 7.4504E-014 |
| | PFHDay2 | BFHDay1 | -6.27496E-013 | 3.40646E-013 | .076 | -1.3253E-012 | 7.0286E-014 |
| | | PFHDay1 | -2.75830E-013 | 3.40646E-013 | .425 | -9.7361E-013 | 4.2195E-013 |
| | | BMHDay1 | 8.98585E-014 | 3.40646E-013 | .794 | -6.0792E-013 | 7.8764E-013 |
| | | PMHDay1 | -2.22491E-013 | 3.40646E-013 | .519 | -9.2027E-013 | 4.7529E-013 |
| | | BFHDay2 | 2.77584E-013 | 3.21164E-013 | .395 | -3.8029E-013 | 9.3546E-013 |
| | | BMHDay2 | -9.92854E-014 | 3.21164E-013 | .760 | -7.5716E-013 | 5.5859E-013 |
| | | PMHDay2 | -3.05788E-013 | 3.21164E-013 | .349 | -9.6366E-013 | 3.5209E-013 |
| | BMHDay2 | BFHDay1 | -5.28210E-013 | 3.40646E-013 | .132 | -1.2260E-012 | 1.6957E-013 |
| | | PFHDay1 | -1.76544E-013 | 3.40646E-013 | .608 | -8.7433E-013 | 5.2124E-013 |
| | | BMHDay1 | 1.89144E-013 | 3.40646E-013 | .583 | -5.0864E-013 | 8.8693E-013 |
| | | PMHDay1 | -1.23206E-013 | 3.40646E-013 | .720 | -8.2099E-013 | 5.7458E-013 |
| | | BFHDay2 | 3.76869E-013 | 3.21164E-013 | .251 | -2.8101E-013 | 1.0347E-012 |
| | | PFHDay2 | 9.92854E-014 | 3.21164E-013 | .760 | -5.5859E-013 | 7.5716E-013 |

| | | | | | | | |
|---|---|---|---|---|---|---|---|
| | | PMHDay2 | -2.06502E-013 | 3.21164E-013 | .525 | -8.6438E-013 | 4.5137E-013 |
| | PMHDay2 | BFHDay1 | -3.21708E-013 | 3.40646E-013 | .353 | -1.0195E-012 | 3.7607E-013 |
| | | PFHDay1 | 2.99583E-014 | 3.40646E-013 | .931 | -6.6782E-013 | 7.2774E-013 |
| | | BMHDay1 | 3.95646E-013 | 3.40646E-013 | .255 | -3.0214E-013 | 1.0934E-012 |
| | | PMHDay1 | 8.32968E-014 | 3.40646E-013 | .809 | -6.1449E-013 | 7.8108E-013 |
| | | BFHDay2 | 5.83371E-013 | 3.21164E-013 | .080 | -7.4504E-014 | 1.2412E-012 |
| | | PFHDay2 | 3.05788E-013 | 3.21164E-013 | .349 | -3.5209E-013 | 9.6366E-013 |
| | | BMHDay2 | 2.06502E-013 | 3.21164E-013 | .525 | -4.5137E-013 | 8.6438E-013 |
| PGEtwo | BFHDay1 | PFHDay1 | -4.80288E-012 | 5.93230E-011 | .936 | -1.2613E-010 | 1.1653E-010 |
| | | BMHDay1 | 1.74825E-010* | 7.84770E-011 | .034 | 1.4321E-011 | 3.3533E-010 |
| | | PMHDay1 | 1.68983E-010* | 5.93230E-011 | .008 | 4.7654E-011 | 2.9031E-010 |
| | | BFHDay2 | 3.71612E-011 | 5.93230E-011 | .536 | -8.4168E-011 | 1.5849E-010 |
| | | PFHDay2 | 4.21185E-011 | 5.93230E-011 | .483 | -7.9211E-011 | 1.6345E-010 |
| | | BMHDay2 | 1.01750E-010 | 5.93230E-011 | .097 | -1.9579E-011 | 2.2308E-010 |
| | | PMHDay2 | 1.21066E-010 | 5.93230E-011 | .050 | -2.6322E-013 | 2.4240E-010 |
| | PFHDay1 | BFHDay1 | 4.80288E-012 | 5.93230E-011 | .936 | -1.1653E-010 | 1.2613E-010 |
| | | BMHDay1 | 1.79628E-010* | 7.84770E-011 | .030 | 1.9124E-011 | 3.4013E-010 |
| | | PMHDay1 | 1.73786E-010* | 5.93230E-011 | .007 | 5.2457E-011 | 2.9512E-010 |
| | | BFHDay2 | 4.19641E-011 | 5.93230E-011 | .485 | -7.9365E-011 | 1.6329E-010 |

| | | | | | | |
|---|---|---|---|---|---|---|
| | PFHDay2 | 4.69214E-011 | 5.93230E-011 | .435 | -7.4408E-011 | 1.6825E-010 |
| | BMHDay2 | 1.06553E-010 | 5.93230E-011 | .083 | -1.4776E-011 | 2.2788E-010 |
| | PMHDay2 | 1.25869E-010* | 5.93230E-011 | .043 | 4.5397E-012 | 2.4720E-010 |
| BMHDay1 | BFHDay1 | -1.74825E-010* | 7.84770E-011 | .034 | -3.3533E-010 | -1.4321E-011 |
| | PFHDay1 | -1.79628E-010* | 7.84770E-011 | .030 | -3.4013E-010 | -1.9124E-011 |
| | PMHDay1 | -5.84138E-012 | 7.84770E-011 | .941 | -1.6634E-010 | 1.5466E-010 |
| | BFHDay2 | -1.37664E-010 | 7.84770E-011 | .090 | -2.9817E-010 | 2.2840E-011 |
| | PFHDay2 | -1.32706E-010 | 7.84770E-011 | .102 | -2.9321E-010 | 2.7797E-011 |
| | BMHDay2 | -7.30746E-011 | 7.84770E-011 | .359 | -2.3358E-010 | 8.7429E-011 |
| | PMHDay2 | -5.37589E-011 | 7.84770E-011 | .499 | -2.1426E-010 | 1.0674E-010 |
| PMHDay1 | BFHDay1 | -1.68983E-010* | 5.93230E-011 | .008 | -2.9031E-010 | -4.7654E-011 |
| | PFHDay1 | -1.73786E-010* | 5.93230E-011 | .007 | -2.9512E-010 | -5.2457E-011 |
| | BMHDay1 | 5.84138E-012 | 7.84770E-011 | .941 | -1.5466E-010 | 1.6634E-010 |
| | BFHDay2 | -1.31822E-010* | 5.93230E-011 | .034 | -2.5315E-010 | -1.0493E-011 |
| | PFHDay2 | -1.26865E-010* | 5.93230E-011 | .041 | -2.4819E-010 | -5.5358E-012 |
| | BMHDay2 | -6.72332E-011 | 5.93230E-011 | .266 | -1.8856E-010 | 5.4096E-011 |
| | PMHDay2 | -4.79175E-011 | 5.93230E-011 | .426 | -1.6925E-010 | 7.3412E-011 |
| BFHDay2 | BFHDay1 | -3.71612E-011 | 5.93230E-011 | .536 | -1.5849E-010 | 8.4168E-011 |
| | PFHDay1 | -4.19641E-011 | 5.93230E-011 | .485 | -1.6329E-010 | 7.9365E-011 |

| | | | | | | |
|---|---|---|---|---|---|---|
| | BMHDay1 | 1.37664E-010 | 7.84770E-011 | .090 | -2.2840E-011 | 2.9817E-010 |
| | PMHDay1 | 1.31822E-010* | 5.93230E-011 | .034 | 1.0493E-011 | 2.5315E-010 |
| | PFHDay2 | 4.95732E-012 | 5.93230E-011 | .934 | -1.1637E-010 | 1.2629E-010 |
| | BMHDay2 | 6.45890E-011 | 5.93230E-011 | .285 | -5.6740E-011 | 1.8592E-010 |
| | PMHDay2 | 8.39048E-011 | 5.93230E-011 | .168 | -3.7424E-011 | 2.0523E-010 |
| PFHDay2 | BFHDay1 | -4.21185E-011 | 5.93230E-011 | .483 | -1.6345E-010 | 7.9211E-011 |
| | PFHDay1 | -4.69214E-011 | 5.93230E-011 | .435 | -1.6825E-010 | 7.4408E-011 |
| | BMHDay1 | 1.32706E-010 | 7.84770E-011 | .102 | -2.7797E-011 | 2.9321E-010 |
| | PMHDay1 | 1.26865E-010* | 5.93230E-011 | .041 | 5.5358E-012 | 2.4819E-010 |
| | BFHDay2 | -4.95732E-012 | 5.93230E-011 | .934 | -1.2629E-010 | 1.1637E-010 |
| | BMHDay2 | 5.96317E-011 | 5.93230E-011 | .323 | -6.1697E-011 | 1.8096E-010 |
| | PMHDay2 | 7.89474E-011 | 5.93230E-011 | .194 | -4.2382E-011 | 2.0028E-010 |
| BMHDay2 | BFHDay1 | -1.01750E-010 | 5.93230E-011 | .097 | -2.2308E-010 | 1.9579E-011 |
| | PFHDay1 | -1.06553E-010 | 5.93230E-011 | .083 | -2.2788E-010 | 1.4776E-011 |
| | BMHDay1 | 7.30746E-011 | 7.84770E-011 | .359 | -8.7429E-011 | 2.3358E-010 |
| | PMHDay1 | 6.72332E-011 | 5.93230E-011 | .266 | -5.4096E-011 | 1.8856E-010 |
| | BFHDay2 | -6.45890E-011 | 5.93230E-011 | .285 | -1.8592E-010 | 5.6740E-011 |
| | PFHDay2 | -5.96317E-011 | 5.93230E-011 | .323 | -1.8096E-010 | 6.1697E-011 |
| | PMHDay2 | 1.93158E-011 | 5.93230E-011 | .747 | -1.0201E-010 | 1.4064E-010 |

| | | | | | | | |
|---|---|---|---|---|---|---|---|
| | PMHDay2 | BFHDay1 | -1.21066E-010 | 5.93230E-011 | .050 | -2.4240E-010 | 2.6322E-013 |
| | | PFHDay1 | -1.25869E-010* | 5.93230E-011 | .043 | -2.4720E-010 | -4.5397E-012 |
| | | BMHDay1 | 5.37589E-011 | 7.84770E-011 | .499 | -1.0674E-010 | 2.1426E-010 |
| | | PMHDay1 | 4.79175E-011 | 5.93230E-011 | .426 | -7.3412E-011 | 1.6925E-010 |
| | | BFHDay2 | -8.39048E-011 | 5.93230E-011 | .168 | -2.0523E-010 | 3.7424E-011 |
| | | PFHDay2 | -7.89474E-011 | 5.93230E-011 | .194 | -2.0028E-010 | 4.2382E-011 |
| | | BMHDay2 | -1.93158E-011 | 5.93230E-011 | .747 | -1.4064E-010 | 1.0201E-010 |
| PGFtwoA | BFHDay1 | PFHDay1 | 7.11052E-011 | 5.16064E-011 | .180 | -3.4973E-011 | 1.7718E-010 |
| | | BMHDay1 | 1.30913E-010 | 6.82689E-011 | .066 | -9.4153E-012 | 2.7124E-010 |
| | | PMHDay1 | 1.84368E-010* | 5.47369E-011 | .002 | 7.1855E-011 | 2.9688E-010 |
| | | BFHDay2 | 9.88194E-011 | 5.16064E-011 | .067 | -7.2591E-012 | 2.0490E-010 |
| | | PFHDay2 | 7.74042E-011 | 5.16064E-011 | .146 | -2.8674E-011 | 1.8348E-010 |
| | | BMHDay2 | 6.93980E-011 | 5.47369E-011 | .216 | -4.3115E-011 | 1.8191E-010 |
| | | PMHDay2 | 1.62838E-010* | 5.47369E-011 | .006 | 5.0325E-011 | 2.7535E-010 |
| | PFHDay1 | BFHDay1 | -7.11052E-011 | 5.16064E-011 | .180 | -1.7718E-010 | 3.4973E-011 |
| | | BMHDay1 | 5.98081E-011 | 6.82689E-011 | .389 | -8.0521E-011 | 2.0014E-010 |
| | | PMHDay1 | 1.13263E-010* | 5.47369E-011 | .049 | 7.4988E-013 | 2.2578E-010 |
| | | BFHDay2 | 2.77142E-011 | 5.16064E-011 | .596 | -7.8364E-011 | 1.3379E-010 |
| | | PFHDay2 | 6.29900E-012 | 5.16064E-011 | .904 | -9.9779E-011 | 1.1238E-010 |

| | | | | | | |
|---|---|---|---|---|---|---|
| | BMHDay2 | -1.70715E-012 | 5.47369E-011 | .975 | -1.1422E-010 | 1.1081E-010 |
| | PMHDay2 | 9.17326E-011 | 5.47369E-011 | .106 | -2.0781E-011 | 2.0425E-010 |
| BMHDay1 | BFHDay1 | -1.30913E-010 | 6.82689E-011 | .066 | -2.7124E-010 | 9.4153E-012 |
| | PFHDay1 | -5.98081E-011 | 6.82689E-011 | .389 | -2.0014E-010 | 8.0521E-011 |
| | PMHDay1 | 5.34550E-011 | 7.06650E-011 | .456 | -9.1799E-011 | 1.9871E-010 |
| | BFHDay2 | -3.20939E-011 | 6.82689E-011 | .642 | -1.7242E-010 | 1.0823E-010 |
| | PFHDay2 | -5.35091E-011 | 6.82689E-011 | .440 | -1.9384E-010 | 8.6820E-011 |
| | BMHDay2 | -6.15152E-011 | 7.06650E-011 | .392 | -2.0677E-010 | 8.3739E-011 |
| | PMHDay2 | 3.19245E-011 | 7.06650E-011 | .655 | -1.1333E-010 | 1.7718E-010 |
| PMHDay1 | BFHDay1 | -1.84368E-010* | 5.47369E-011 | .002 | -2.9688E-010 | -7.1855E-011 |
| | PFHDay1 | -1.13263E-010* | 5.47369E-011 | .049 | -2.2578E-010 | -7.4988E-013 |
| | BMHDay1 | -5.34550E-011 | 7.06650E-011 | .456 | -1.9871E-010 | 9.1799E-011 |
| | BFHDay2 | -8.55489E-011 | 5.47369E-011 | .130 | -1.9806E-010 | 2.6964E-011 |
| | PFHDay2 | -1.06964E-010 | 5.47369E-011 | .062 | -2.1948E-010 | 5.5491E-012 |
| | BMHDay2 | -1.14970E-010 | 5.76977E-011 | .057 | -2.3357E-010 | 3.6291E-012 |
| | PMHDay2 | -2.15305E-011 | 5.76977E-011 | .712 | -1.4013E-010 | 9.7069E-011 |
| BFHDay2 | BFHDay1 | -9.88194E-011 | 5.16064E-011 | .067 | -2.0490E-010 | 7.2591E-012 |
| | PFHDay1 | -2.77142E-011 | 5.16064E-011 | .596 | -1.3379E-010 | 7.8364E-011 |
| | BMHDay1 | 3.20939E-011 | 6.82689E-011 | .642 | -1.0823E-010 | 1.7242E-010 |

| | | | | | | |
|---|---|---|---|---|---|---|
| | PMHDay1 | 8.55489E-011 | 5.47369E-011 | .130 | -2.6964E-011 | 1.9806E-010 |
| | PFHDay2 | -2.14152E-011 | 5.16064E-011 | .682 | -1.2749E-010 | 8.4663E-011 |
| | BMHDay2 | -2.94213E-011 | 5.47369E-011 | .595 | -1.4193E-010 | 8.3092E-011 |
| | PMHDay2 | 6.40184E-011 | 5.47369E-011 | .253 | -4.8495E-011 | 1.7653E-010 |
| PFHDay2 | BFHDay1 | -7.74042E-011 | 5.16064E-011 | .146 | -1.8348E-010 | 2.8674E-011 |
| | PFHDay1 | -6.29900E-012 | 5.16064E-011 | .904 | -1.1238E-010 | 9.9779E-011 |
| | BMHDay1 | 5.35091E-011 | 6.82689E-011 | .440 | -8.6820E-011 | 1.9384E-010 |
| | PMHDay1 | 1.06964E-010 | 5.47369E-011 | .062 | -5.5491E-012 | 2.1948E-010 |
| | BFHDay2 | 2.14152E-011 | 5.16064E-011 | .682 | -8.4663E-011 | 1.2749E-010 |
| | BMHDay2 | -8.00615E-012 | 5.47369E-011 | .885 | -1.2052E-010 | 1.0451E-010 |
| | PMHDay2 | 8.54336E-011 | 5.47369E-011 | .131 | -2.7080E-011 | 1.9795E-010 |
| BMHDay2 | BFHDay1 | -6.93980E-011 | 5.47369E-011 | .216 | -1.8191E-010 | 4.3115E-011 |
| | PFHDay1 | 1.70715E-012 | 5.47369E-011 | .975 | -1.1081E-010 | 1.1422E-010 |
| | BMHDay1 | 6.15152E-011 | 7.06650E-011 | .392 | -8.3739E-011 | 2.0677E-010 |
| | PMHDay1 | 1.14970E-010 | 5.76977E-011 | .057 | -3.6291E-012 | 2.3357E-010 |
| | BFHDay2 | 2.94213E-011 | 5.47369E-011 | .595 | -8.3092E-011 | 1.4193E-010 |
| | PFHDay2 | 8.00615E-012 | 5.47369E-011 | .885 | -1.0451E-010 | 1.2052E-010 |
| | PMHDay2 | 9.34397E-011 | 5.76977E-011 | .117 | -2.5160E-011 | 2.1204E-010 |
| PMHDay2 | BFHDay1 | -1.62838E-010* | 5.47369E-011 | .006 | -2.7535E-010 | -5.0325E-011 |

| | | | | | | | |
|---|---|---|---|---|---|---|---|
| | | PFHDay1 | -9.17326E-011 | 5.47369E-011 | .106 | -2.0425E-010 | 2.0781E-011 |
| | | BMHDay1 | -3.19245E-011 | 7.06650E-011 | .655 | -1.7718E-010 | 1.1333E-010 |
| | | PMHDay1 | 2.15305E-011 | 5.76977E-011 | .712 | -9.7069E-011 | 1.4013E-010 |
| | | BFHDay2 | -6.40184E-011 | 5.47369E-011 | .253 | -1.7653E-010 | 4.8495E-011 |
| | | PFHDay2 | -8.54336E-011 | 5.47369E-011 | .131 | -1.9795E-010 | 2.7080E-011 |
| | | BMHDay2 | -9.34397E-011 | 5.76977E-011 | .117 | -2.1204E-010 | 2.5160E-011 |
| palProline | BFHDay1 | PFHDay1 | -4.39791E-012 | 4.26149E-012 | .310 | -1.3078E-011 | 4.2825E-012 |
| | | BMHDay1 | -9.32232E-013 | 4.26149E-012 | .828 | -9.6126E-012 | 7.7481E-012 |
| | | PMHDay1 | -7.39375E-012 | 4.26149E-012 | .092 | -1.6074E-011 | 1.2866E-012 |
| | | BFHDay2 | 2.77741E-012 | 4.26149E-012 | .519 | -5.9030E-012 | 1.1458E-011 |
| | | PFHDay2 | -7.04245E-012 | 4.26149E-012 | .108 | -1.5723E-011 | 1.6379E-012 |
| | | BMHDay2 | -3.17877E-012 | 4.26149E-012 | .461 | -1.1859E-011 | 5.5016E-012 |
| | | PMHDay2 | -2.64518E-012 | 4.26149E-012 | .539 | -1.1326E-011 | 6.0352E-012 |
| | PFHDay1 | BFHDay1 | 4.39791E-012 | 4.26149E-012 | .310 | -4.2825E-012 | 1.3078E-011 |
| | | BMHDay1 | 3.46568E-012 | 4.26149E-012 | .422 | -5.2147E-012 | 1.2146E-011 |
| | | PMHDay1 | -2.99584E-012 | 4.26149E-012 | .487 | -1.1676E-011 | 5.6845E-012 |
| | | BFHDay2 | 7.17532E-012 | 4.26149E-012 | .102 | -1.5051E-012 | 1.5856E-011 |
| | | PFHDay2 | -2.64454E-012 | 4.26149E-012 | .539 | -1.1325E-011 | 6.0358E-012 |
| | | BMHDay2 | 1.21914E-012 | 4.26149E-012 | .777 | -7.4612E-012 | 9.8995E-012 |

| | | | | | | |
|---|---|---|---|---|---|---|
| | PMHDay2 | 1.75273E-012 | 4.26149E-012 | .684 | -6.9276E-012 | 1.0433E-011 |
| BMHDay1 | BFHDay1 | 9.32232E-013 | 4.26149E-012 | .828 | -7.7481E-012 | 9.6126E-012 |
| | PFHDay1 | -3.46568E-012 | 4.26149E-012 | .422 | -1.2146E-011 | 5.2147E-012 |
| | PMHDay1 | -6.46152E-012 | 4.26149E-012 | .139 | -1.5142E-011 | 2.2189E-012 |
| | BFHDay2 | 3.70964E-012 | 4.26149E-012 | .391 | -4.9707E-012 | 1.2390E-011 |
| | PFHDay2 | -6.11022E-012 | 4.26149E-012 | .161 | -1.4791E-011 | 2.5702E-012 |
| | BMHDay2 | -2.24654E-012 | 4.26149E-012 | .602 | -1.0927E-011 | 6.4338E-012 |
| | PMHDay2 | -1.71295E-012 | 4.26149E-012 | .690 | -1.0393E-011 | 6.9674E-012 |
| PMHDay1 | BFHDay1 | 7.39375E-012 | 4.26149E-012 | .092 | -1.2866E-012 | 1.6074E-011 |
| | PFHDay1 | 2.99584E-012 | 4.26149E-012 | .487 | -5.6845E-012 | 1.1676E-011 |
| | BMHDay1 | 6.46152E-012 | 4.26149E-012 | .139 | -2.2189E-012 | 1.5142E-011 |
| | BFHDay2 | 1.01712E-011* | 4.26149E-012 | .023 | 1.4908E-012 | 1.8852E-011 |
| | PFHDay2 | 3.51300E-013 | 4.26149E-012 | .935 | -8.3291E-012 | 9.0317E-012 |
| | BMHDay2 | 4.21498E-012 | 4.26149E-012 | .330 | -4.4654E-012 | 1.2895E-011 |
| | PMHDay2 | 4.74857E-012 | 4.26149E-012 | .273 | -3.9318E-012 | 1.3429E-011 |
| BFHDay2 | BFHDay1 | -2.77741E-012 | 4.26149E-012 | .519 | -1.1458E-011 | 5.9030E-012 |
| | PFHDay1 | -7.17532E-012 | 4.26149E-012 | .102 | -1.5856E-011 | 1.5051E-012 |
| | BMHDay1 | -3.70964E-012 | 4.26149E-012 | .391 | -1.2390E-011 | 4.9707E-012 |
| | PMHDay1 | -1.01712E-011* | 4.26149E-012 | .023 | -1.8852E-011 | -1.4908E-012 |

| | | | | | | | |
|---|---|---|---|---|---|---|---|
| | | PFHDay2 | -9.81986E-012* | 4.26149E-012 | .028 | -1.8500E-011 | -1.1395E-012 |
| | | BMHDay2 | -5.95618E-012 | 4.26149E-012 | .172 | -1.4637E-011 | 2.7242E-012 |
| | | PMHDay2 | -5.42259E-012 | 4.26149E-012 | .212 | -1.4103E-011 | 3.2578E-012 |
| | PFHDay2 | BFHDay1 | 7.04245E-012 | 4.26149E-012 | .108 | -1.6379E-012 | 1.5723E-011 |
| | | PFHDay1 | 2.64454E-012 | 4.26149E-012 | .539 | -6.0358E-012 | 1.1325E-011 |
| | | BMHDay1 | 6.11022E-012 | 4.26149E-012 | .161 | -2.5702E-012 | 1.4791E-011 |
| | | PMHDay1 | -3.51300E-013 | 4.26149E-012 | .935 | -9.0317E-012 | 8.3291E-012 |
| | | BFHDay2 | 9.81986E-012* | 4.26149E-012 | .028 | 1.1395E-012 | 1.8500E-011 |
| | | BMHDay2 | 3.86368E-012 | 4.26149E-012 | .371 | -4.8167E-012 | 1.2544E-011 |
| | | PMHDay2 | 4.39727E-012 | 4.26149E-012 | .310 | -4.2831E-012 | 1.3078E-011 |
| | BMHDay2 | BFHDay1 | 3.17877E-012 | 4.26149E-012 | .461 | -5.5016E-012 | 1.1859E-011 |
| | | PFHDay1 | -1.21914E-012 | 4.26149E-012 | .777 | -9.8995E-012 | 7.4612E-012 |
| | | BMHDay1 | 2.24654E-012 | 4.26149E-012 | .602 | -6.4338E-012 | 1.0927E-011 |
| | | PMHDay1 | -4.21498E-012 | 4.26149E-012 | .330 | -1.2895E-011 | 4.4654E-012 |
| | | BFHDay2 | 5.95618E-012 | 4.26149E-012 | .172 | -2.7242E-012 | 1.4637E-011 |
| | | PFHDay2 | -3.86368E-012 | 4.26149E-012 | .371 | -1.2544E-011 | 4.8167E-012 |
| | | PMHDay2 | 5.33592E-013 | 4.26149E-012 | .901 | -8.1468E-012 | 9.2140E-012 |
| | PMHDay2 | BFHDay1 | 2.64518E-012 | 4.26149E-012 | .539 | -6.0352E-012 | 1.1326E-011 |
| | | PFHDay1 | -1.75273E-012 | 4.26149E-012 | .684 | -1.0433E-011 | 6.9276E-012 |

| | | | | | | | |
|---|---|---|---|---|---|---|---|
| | | BMHDay1 | 1.71295E-012 | 4.26149E-012 | .690 | -6.9674E-012 | 1.0393E-011 |
| | | PMHDay1 | -4.74857E-012 | 4.26149E-012 | .273 | -1.3429E-011 | 3.9318E-012 |
| | | BFHDay2 | 5.42259E-012 | 4.26149E-012 | .212 | -3.2578E-012 | 1.4103E-011 |
| | | PFHDay2 | -4.39727E-012 | 4.26149E-012 | .310 | -1.3078E-011 | 4.2831E-012 |
| | | BMHDay2 | -5.33592E-013 | 4.26149E-012 | .901 | -9.2140E-012 | 8.1468E-012 |
| oleProline | BFHDay1 | PFHDay1 | -1.05712E-012 | 6.35384E-013 | .106 | -2.3514E-012 | 2.3712E-013 |
| | | BMHDay1 | -1.13980E-012 | 6.35384E-013 | .082 | -2.4340E-012 | 1.5443E-013 |
| | | PMHDay1 | -2.53262E-012* | 6.35384E-013 | <.001 | -3.8269E-012 | -1.2384E-012 |
| | | BFHDay2 | -5.02524E-013 | 6.35384E-013 | .435 | -1.7968E-012 | 7.9171E-013 |
| | | PFHDay2 | -8.05792E-013 | 6.35384E-013 | .214 | -2.1000E-012 | 4.8844E-013 |
| | | BMHDay2 | -1.60326E-012* | 6.35384E-013 | .017 | -2.8975E-012 | -3.0903E-013 |
| | | PMHDay2 | -9.53768E-013 | 6.35384E-013 | .143 | -2.2480E-012 | 3.4047E-013 |
| | PFHDay1 | BFHDay1 | 1.05712E-012 | 6.35384E-013 | .106 | -2.3712E-013 | 2.3514E-012 |
| | | BMHDay1 | -8.26840E-014 | 6.35384E-013 | .897 | -1.3769E-012 | 1.2115E-012 |
| | | PMHDay1 | -1.47550E-012* | 6.35384E-013 | .027 | -2.7697E-012 | -1.8127E-013 |
| | | BFHDay2 | 5.54594E-013 | 6.35384E-013 | .389 | -7.3964E-013 | 1.8488E-012 |
| | | PFHDay2 | 2.51326E-013 | 6.35384E-013 | .695 | -1.0429E-012 | 1.5456E-012 |
| | | BMHDay2 | -5.46144E-013 | 6.35384E-013 | .396 | -1.8404E-012 | 7.4809E-013 |
| | | PMHDay2 | 1.03350E-013 | 6.35384E-013 | .872 | -1.1909E-012 | 1.3976E-012 |

| | | | | | | |
|---|---|---|---|---|---|---|
| | BMHDay1 | BFHDay1 | 1.13980E-012 | 6.35384E-013 | .082 | -1.5443E-013 | 2.4340E-012 |
| | | PFHDay1 | 8.26840E-014 | 6.35384E-013 | .897 | -1.2115E-012 | 1.3769E-012 |
| | | PMHDay1 | -1.39282E-012* | 6.35384E-013 | .036 | -2.6871E-012 | -9.8584E-014 |
| | | BFHDay2 | 6.37278E-013 | 6.35384E-013 | .323 | -6.5696E-013 | 1.9315E-012 |
| | | PFHDay2 | 3.34010E-013 | 6.35384E-013 | .603 | -9.6022E-013 | 1.6282E-012 |
| | | BMHDay2 | -4.63460E-013 | 6.35384E-013 | .471 | -1.7577E-012 | 8.3077E-013 |
| | | PMHDay2 | 1.86034E-013 | 6.35384E-013 | .772 | -1.1082E-012 | 1.4803E-012 |
| | PMHDay1 | BFHDay1 | 2.53262E-012* | 6.35384E-013 | <.001 | 1.2384E-012 | 3.8269E-012 |
| | | PFHDay1 | 1.47550E-012* | 6.35384E-013 | .027 | 1.8127E-013 | 2.7697E-012 |
| | | BMHDay1 | 1.39282E-012* | 6.35384E-013 | .036 | 9.8584E-014 | 2.6871E-012 |
| | | BFHDay2 | 2.03010E-012* | 6.35384E-013 | .003 | 7.3586E-013 | 3.3243E-012 |
| | | PFHDay2 | 1.72683E-012* | 6.35384E-013 | .011 | 4.3259E-013 | 3.0211E-012 |
| | | BMHDay2 | 9.29358E-013 | 6.35384E-013 | .153 | -3.6488E-013 | 2.2236E-012 |
| | | PMHDay2 | 1.57885E-012* | 6.35384E-013 | .018 | 2.8462E-013 | 2.8731E-012 |
| | BFHDay2 | BFHDay1 | 5.02524E-013 | 6.35384E-013 | .435 | -7.9171E-013 | 1.7968E-012 |
| | | PFHDay1 | -5.54594E-013 | 6.35384E-013 | .389 | -1.8488E-012 | 7.3964E-013 |
| | | BMHDay1 | -6.37278E-013 | 6.35384E-013 | .323 | -1.9315E-012 | 6.5696E-013 |
| | | PMHDay1 | -2.03010E-012* | 6.35384E-013 | .003 | -3.3243E-012 | -7.3586E-013 |
| | | PFHDay2 | -3.03268E-013 | 6.35384E-013 | .636 | -1.5975E-012 | 9.9097E-013 |

| | | | | | | | |
|---|---|---|---|---|---|---|---|
| | | BMHDay2 | -1.10074E-012 | 6.35384E-013 | .093 | -2.3950E-012 | 1.9350E-013 |
| | | PMHDay2 | -4.51244E-013 | 6.35384E-013 | .483 | -1.7455E-012 | 8.4299E-013 |
| | PFHDay2 | BFHDay1 | 8.05792E-013 | 6.35384E-013 | .214 | -4.8844E-013 | 2.1000E-012 |
| | | PFHDay1 | -2.51326E-013 | 6.35384E-013 | .695 | -1.5456E-012 | 1.0429E-012 |
| | | BMHDay1 | -3.34010E-013 | 6.35384E-013 | .603 | -1.6282E-012 | 9.6022E-013 |
| | | PMHDay1 | -1.72683E-012* | 6.35384E-013 | .011 | -3.0211E-012 | -4.3259E-013 |
| | | BFHDay2 | 3.03268E-013 | 6.35384E-013 | .636 | -9.9097E-013 | 1.5975E-012 |
| | | BMHDay2 | -7.97470E-013 | 6.35384E-013 | .219 | -2.0917E-012 | 4.9676E-013 |
| | | PMHDay2 | -1.47976E-013 | 6.35384E-013 | .817 | -1.4422E-012 | 1.1463E-012 |
| | BMHDay2 | BFHDay1 | 1.60326E-012* | 6.35384E-013 | .017 | 3.0903E-013 | 2.8975E-012 |
| | | PFHDay1 | 5.46144E-013 | 6.35384E-013 | .396 | -7.4809E-013 | 1.8404E-012 |
| | | BMHDay1 | 4.63460E-013 | 6.35384E-013 | .471 | -8.3077E-013 | 1.7577E-012 |
| | | PMHDay1 | -9.29358E-013 | 6.35384E-013 | .153 | -2.2236E-012 | 3.6488E-013 |
| | | BFHDay2 | 1.10074E-012 | 6.35384E-013 | .093 | -1.9350E-013 | 2.3950E-012 |
| | | PFHDay2 | 7.97470E-013 | 6.35384E-013 | .219 | -4.9676E-013 | 2.0917E-012 |
| | | PMHDay2 | 6.49494E-013 | 6.35384E-013 | .314 | -6.4474E-013 | 1.9437E-012 |
| | PMHDay2 | BFHDay1 | 9.53768E-013 | 6.35384E-013 | .143 | -3.4047E-013 | 2.2480E-012 |
| | | PFHDay1 | -1.03350E-013 | 6.35384E-013 | .872 | -1.3976E-012 | 1.1909E-012 |
| | | BMHDay1 | -1.86034E-013 | 6.35384E-013 | .772 | -1.4803E-012 | 1.1082E-012 |

| | | | | | | | |
|---|---|---|---|---|---|---|---|
| | | PMHDay1 | -1.57885E-012* | 6.35384E-013 | .018 | -2.8731E-012 | -2.8462E-013 |
| | | BFHDay2 | 4.51244E-013 | 6.35384E-013 | .483 | -8.4299E-013 | 1.7455E-012 |
| | | PFHDay2 | 1.47976E-013 | 6.35384E-013 | .817 | -1.1463E-012 | 1.4422E-012 |
| | | BMHDay2 | -6.49494E-013 | 6.35384E-013 | .314 | -1.9437E-012 | 6.4474E-013 |
| linProline | BFHDay1 | PFHDay1 | 9.36628E-013 | 4.78615E-013 | .066 | -6.8905E-014 | 1.9422E-012 |
| | | BMHDay1 | 8.56240E-013 | 4.28086E-013 | .061 | -4.3136E-014 | 1.7556E-012 |
| | | PMHDay1 | 4.36285E-013 | 4.00438E-013 | .290 | -4.0500E-013 | 1.2776E-012 |
| | | BFHDay2 | 5.67853E-013 | 4.28086E-013 | .201 | -3.3152E-013 | 1.4672E-012 |
| | | PFHDay2 | 8.09647E-013 | 4.28086E-013 | .075 | -8.9729E-014 | 1.7090E-012 |
| | | BMHDay2 | 2.86961E-013 | 4.00438E-013 | .483 | -5.5433E-013 | 1.1283E-012 |
| | | PMHDay2 | 6.92772E-013 | 4.00438E-013 | .101 | -1.4852E-013 | 1.5341E-012 |
| | PFHDay1 | BFHDay1 | -9.36628E-013 | 4.78615E-013 | .066 | -1.9422E-012 | 6.8905E-014 |
| | | BMHDay1 | -8.03887E-014 | 4.78615E-013 | .868 | -1.0859E-012 | 9.2514E-013 |
| | | PMHDay1 | -5.00343E-013 | 4.54054E-013 | .285 | -1.4543E-012 | 4.5359E-013 |
| | | BFHDay2 | -3.68775E-013 | 4.78615E-013 | .451 | -1.3743E-012 | 6.3676E-013 |
| | | PFHDay2 | -1.26982E-013 | 4.78615E-013 | .794 | -1.1325E-012 | 8.7855E-013 |
| | | BMHDay2 | -6.49668E-013 | 4.54054E-013 | .170 | -1.6036E-012 | 3.0426E-013 |
| | | PMHDay2 | -2.43856E-013 | 4.54054E-013 | .598 | -1.1978E-012 | 7.1008E-013 |
| | BMHDay1 | BFHDay1 | -8.56240E-013 | 4.28086E-013 | .061 | -1.7556E-012 | 4.3136E-014 |

| | | | | | | |
|---|---|---|---|---|---|---|
| | PFHDay1 | 8.03887E-014 | 4.78615E-013 | .868 | -9.2514E-013 | 1.0859E-012 |
| | PMHDay1 | -4.19954E-013 | 4.00438E-013 | .308 | -1.2612E-012 | 4.2133E-013 |
| | BFHDay2 | -2.88386E-013 | 4.28086E-013 | .509 | -1.1878E-012 | 6.1099E-013 |
| | PFHDay2 | -4.65930E-014 | 4.28086E-013 | .915 | -9.4597E-013 | 8.5278E-013 |
| | BMHDay2 | -5.69279E-013 | 4.00438E-013 | .172 | -1.4106E-012 | 2.7201E-013 |
| | PMHDay2 | -1.63467E-013 | 4.00438E-013 | .688 | -1.0048E-012 | 6.7782E-013 |
| PMHDay1 | BFHDay1 | -4.36285E-013 | 4.00438E-013 | .290 | -1.2776E-012 | 4.0500E-013 |
| | PFHDay1 | 5.00343E-013 | 4.54054E-013 | .285 | -4.5359E-013 | 1.4543E-012 |
| | BMHDay1 | 4.19954E-013 | 4.00438E-013 | .308 | -4.2133E-013 | 1.2612E-012 |
| | BFHDay2 | 1.31568E-013 | 4.00438E-013 | .746 | -7.0972E-013 | 9.7286E-013 |
| | PFHDay2 | 3.73361E-013 | 4.00438E-013 | .363 | -4.6793E-013 | 1.2147E-012 |
| | BMHDay2 | -1.49325E-013 | 3.70734E-013 | .692 | -9.2821E-013 | 6.2956E-013 |
| | PMHDay2 | 2.56487E-013 | 3.70734E-013 | .498 | -5.2240E-013 | 1.0354E-012 |
| BFHDay2 | BFHDay1 | -5.67853E-013 | 4.28086E-013 | .201 | -1.4672E-012 | 3.3152E-013 |
| | PFHDay1 | 3.68775E-013 | 4.78615E-013 | .451 | -6.3676E-013 | 1.3743E-012 |
| | BMHDay1 | 2.88386E-013 | 4.28086E-013 | .509 | -6.1099E-013 | 1.1878E-012 |
| | PMHDay1 | -1.31568E-013 | 4.00438E-013 | .746 | -9.7286E-013 | 7.0972E-013 |
| | PFHDay2 | 2.41793E-013 | 4.28086E-013 | .579 | -6.5758E-013 | 1.1412E-012 |
| | BMHDay2 | -2.80893E-013 | 4.00438E-013 | .492 | -1.1222E-012 | 5.6040E-013 |

| | | | | | | |
|---|---|---|---|---|---|---|
| | PMHDay2 | 1.24919E-013 | 4.00438E-013 | .759 | -7.1637E-013 | 9.6621E-013 |
| PFHDay2 | BFHDay1 | -8.09647E-013 | 4.28086E-013 | .075 | -1.7090E-012 | 8.9729E-014 |
| | PFHDay1 | 1.26982E-013 | 4.78615E-013 | .794 | -8.7855E-013 | 1.1325E-012 |
| | BMHDay1 | 4.65930E-014 | 4.28086E-013 | .915 | -8.5278E-013 | 9.4597E-013 |
| | PMHDay1 | -3.73361E-013 | 4.00438E-013 | .363 | -1.2147E-012 | 4.6793E-013 |
| | BFHDay2 | -2.41793E-013 | 4.28086E-013 | .579 | -1.1412E-012 | 6.5758E-013 |
| | BMHDay2 | -5.22686E-013 | 4.00438E-013 | .208 | -1.3640E-012 | 3.1860E-013 |
| | PMHDay2 | -1.16874E-013 | 4.00438E-013 | .774 | -9.5816E-013 | 7.2441E-013 |
| BMHDay2 | BFHDay1 | -2.86961E-013 | 4.00438E-013 | .483 | -1.1283E-012 | 5.5433E-013 |
| | PFHDay1 | 6.49668E-013 | 4.54054E-013 | .170 | -3.0426E-013 | 1.6036E-012 |
| | BMHDay1 | 5.69279E-013 | 4.00438E-013 | .172 | -2.7201E-013 | 1.4106E-012 |
| | PMHDay1 | 1.49325E-013 | 3.70734E-013 | .692 | -6.2956E-013 | 9.2821E-013 |
| | BFHDay2 | 2.80893E-013 | 4.00438E-013 | .492 | -5.6040E-013 | 1.1222E-012 |
| | PFHDay2 | 5.22686E-013 | 4.00438E-013 | .208 | -3.1860E-013 | 1.3640E-012 |
| | PMHDay2 | 4.05812E-013 | 3.70734E-013 | .288 | -3.7307E-013 | 1.1847E-012 |
| PMHDay2 | BFHDay1 | -6.92772E-013 | 4.00438E-013 | .101 | -1.5341E-012 | 1.4852E-013 |
| | PFHDay1 | 2.43856E-013 | 4.54054E-013 | .598 | -7.1008E-013 | 1.1978E-012 |
| | BMHDay1 | 1.63467E-013 | 4.00438E-013 | .688 | -6.7782E-013 | 1.0048E-012 |
| | PMHDay1 | -2.56487E-013 | 3.70734E-013 | .498 | -1.0354E-012 | 5.2240E-013 |

| | | | | | | | |
|---|---|---|---|---|---|---|---|
| | | BFHDay2 | -1.24919E-013 | 4.00438E-013 | .759 | -9.6621E-013 | 7.1637E-013 |
| | | PFHDay2 | 1.16874E-013 | 4.00438E-013 | .774 | -7.2441E-013 | 9.5816E-013 |
| | | BMHDay2 | -4.05812E-013 | 3.70734E-013 | .288 | -1.1847E-012 | 3.7307E-013 |
| araProline | BFHDay1 | PFHDay1 | 5.72825E-014 | 4.25144E-013 | .894 | -8.2017E-013 | 9.3474E-013 |
| | | BMHDay1 | -8.71549E-014 | 3.29315E-013 | .794 | -7.6683E-013 | 5.9252E-013 |
| | | PMHDay1 | 1.56598E-014 | 3.47129E-013 | .964 | -7.0078E-013 | 7.3210E-013 |
| | | BFHDay2 | 3.24288E-013 | 3.29315E-013 | .335 | -3.5539E-013 | 1.0040E-012 |
| | | PFHDay2 | 1.66892E-013 | 3.47129E-013 | .635 | -5.4955E-013 | 8.8333E-013 |
| | | BMHDay2 | -4.64197E-013 | 3.29315E-013 | .171 | -1.1439E-012 | 2.1548E-013 |
| | | PMHDay2 | -1.26302E-013 | 3.74942E-013 | .739 | -9.0014E-013 | 6.4754E-013 |
| | PFHDay1 | BFHDay1 | -5.72825E-014 | 4.25144E-013 | .894 | -9.3474E-013 | 8.2017E-013 |
| | | BMHDay1 | -1.44437E-013 | 4.10728E-013 | .728 | -9.9214E-013 | 7.0326E-013 |
| | | PMHDay1 | -4.16227E-014 | 4.25144E-013 | .923 | -9.1908E-013 | 8.3583E-013 |
| | | BFHDay2 | 2.67005E-013 | 4.10728E-013 | .522 | -5.8070E-013 | 1.1147E-012 |
| | | PFHDay2 | 1.09610E-013 | 4.25144E-013 | .799 | -7.6784E-013 | 9.8706E-013 |
| | | BMHDay2 | -5.21480E-013 | 4.10728E-013 | .216 | -1.3692E-012 | 3.2622E-013 |
| | | PMHDay2 | -1.83584E-013 | 4.48141E-013 | .686 | -1.1085E-012 | 7.4133E-013 |
| | BMHDay1 | BFHDay1 | 8.71549E-014 | 3.29315E-013 | .794 | -5.9252E-013 | 7.6683E-013 |
| | | PFHDay1 | 1.44437E-013 | 4.10728E-013 | .728 | -7.0326E-013 | 9.9214E-013 |

| | | | | | | |
|---|---|---|---|---|---|---|
| | PMHDay1 | 1.02815E-013 | 3.29315E-013 | .758 | -5.7686E-013 | 7.8249E-013 |
| | BFHDay2 | 4.11443E-013 | 3.10481E-013 | .198 | -2.2936E-013 | 1.0522E-012 |
| | PFHDay2 | 2.54047E-013 | 3.29315E-013 | .448 | -4.2563E-013 | 9.3372E-013 |
| | BMHDay2 | -3.77043E-013 | 3.10481E-013 | .236 | -1.0178E-012 | 2.6376E-013 |
| | PMHDay2 | -3.91469E-014 | 3.58513E-013 | .914 | -7.7908E-013 | 7.0079E-013 |
| PMHDay1 | BFHDay1 | -1.56598E-014 | 3.47129E-013 | .964 | -7.3210E-013 | 7.0078E-013 |
| | PFHDay1 | 4.16227E-014 | 4.25144E-013 | .923 | -8.3583E-013 | 9.1908E-013 |
| | BMHDay1 | -1.02815E-013 | 3.29315E-013 | .758 | -7.8249E-013 | 5.7686E-013 |
| | BFHDay2 | 3.08628E-013 | 3.29315E-013 | .358 | -3.7105E-013 | 9.8830E-013 |
| | PFHDay2 | 1.51232E-013 | 3.47129E-013 | .667 | -5.6521E-013 | 8.6767E-013 |
| | BMHDay2 | -4.79857E-013 | 3.29315E-013 | .158 | -1.1595E-012 | 1.9982E-013 |
| | PMHDay2 | -1.41962E-013 | 3.74942E-013 | .708 | -9.1580E-013 | 6.3188E-013 |
| BFHDay2 | BFHDay1 | -3.24288E-013 | 3.29315E-013 | .335 | -1.0040E-012 | 3.5539E-013 |
| | PFHDay1 | -2.67005E-013 | 4.10728E-013 | .522 | -1.1147E-012 | 5.8070E-013 |
| | BMHDay1 | -4.11443E-013 | 3.10481E-013 | .198 | -1.0522E-012 | 2.2936E-013 |
| | PMHDay1 | -3.08628E-013 | 3.29315E-013 | .358 | -9.8830E-013 | 3.7105E-013 |
| | PFHDay2 | -1.57396E-013 | 3.29315E-013 | .637 | -8.3707E-013 | 5.2228E-013 |
| | BMHDay2 | -7.88485E-013* | 3.10481E-013 | .018 | -1.4293E-012 | -1.4768E-013 |
| | PMHDay2 | -4.50590E-013 | 3.58513E-013 | .221 | -1.1905E-012 | 2.8934E-013 |

| | | | | | | |
|---|---|---|---|---|---|---|
| PFHDay2 | BFHDay1 | -1.66892E-013 | 3.47129E-013 | .635 | -8.8333E-013 | 5.4955E-013 |
| | PFHDay1 | -1.09610E-013 | 4.25144E-013 | .799 | -9.8706E-013 | 7.6784E-013 |
| | BMHDay1 | -2.54047E-013 | 3.29315E-013 | .448 | -9.3372E-013 | 4.2563E-013 |
| | PMHDay1 | -1.51232E-013 | 3.47129E-013 | .667 | -8.6767E-013 | 5.6521E-013 |
| | BFHDay2 | 1.57396E-013 | 3.29315E-013 | .637 | -5.2228E-013 | 8.3707E-013 |
| | BMHDay2 | -6.31090E-013 | 3.29315E-013 | .067 | -1.3108E-012 | 4.8583E-014 |
| | PMHDay2 | -2.93194E-013 | 3.74942E-013 | .442 | -1.0670E-012 | 4.8065E-013 |
| BMHDay2 | BFHDay1 | 4.64197E-013 | 3.29315E-013 | .171 | -2.1548E-013 | 1.1439E-012 |
| | PFHDay1 | 5.21480E-013 | 4.10728E-013 | .216 | -3.2622E-013 | 1.3692E-012 |
| | BMHDay1 | 3.77043E-013 | 3.10481E-013 | .236 | -2.6376E-013 | 1.0178E-012 |
| | PMHDay1 | 4.79857E-013 | 3.29315E-013 | .158 | -1.9982E-013 | 1.1595E-012 |
| | BFHDay2 | 7.88485E-013* | 3.10481E-013 | .018 | 1.4768E-013 | 1.4293E-012 |
| | PFHDay2 | 6.31090E-013 | 3.29315E-013 | .067 | -4.8583E-014 | 1.3108E-012 |
| | PMHDay2 | 3.37896E-013 | 3.58513E-013 | .355 | -4.0204E-013 | 1.0778E-012 |
| PMHDay2 | BFHDay1 | 1.26302E-013 | 3.74942E-013 | .739 | -6.4754E-013 | 9.0014E-013 |
| | PFHDay1 | 1.83584E-013 | 4.48141E-013 | .686 | -7.4133E-013 | 1.1085E-012 |
| | BMHDay1 | 3.91469E-014 | 3.58513E-013 | .914 | -7.0079E-013 | 7.7908E-013 |
| | PMHDay1 | 1.41962E-013 | 3.74942E-013 | .708 | -6.3188E-013 | 9.1580E-013 |
| | BFHDay2 | 4.50590E-013 | 3.58513E-013 | .221 | -2.8934E-013 | 1.1905E-012 |

| | | | | | | | |
|---|---|---|---|---|---|---|---|
| | | PFHDay2 | 2.93194E-013 | 3.74942E-013 | .442 | -4.8065E-013 | 1.0670E-012 |
| | | BMHDay2 | -3.37896E-013 | 3.58513E-013 | .355 | -1.0778E-012 | 4.0204E-013 |
| docProline | BFHDay1 | PFHDay1 | -5.21736E-010 | 1.03886E-009 | .620 | -2.6571E-009 | 1.6137E-009 |
| | | BMHDay1 | -1.22833E-009 | 1.08647E-009 | .269 | -3.4616E-009 | 1.0049E-009 |
| | | PMHDay1 | -4.08676E-010 | 1.08647E-009 | .710 | -2.6419E-009 | 1.8246E-009 |
| | | BFHDay2 | 2.18160E-010 | 1.03886E-009 | .835 | -1.9173E-009 | 2.3536E-009 |
| | | PFHDay2 | 2.07516E-010 | 1.08647E-009 | .850 | -2.0258E-009 | 2.4408E-009 |
| | | BMHDay2 | 5.10350E-012 | 1.08647E-009 | .996 | -2.2282E-009 | 2.2384E-009 |
| | | PMHDay2 | -8.00106E-011 | 1.03886E-009 | .939 | -2.2154E-009 | 2.0554E-009 |
| | PFHDay1 | BFHDay1 | 5.21736E-010 | 1.03886E-009 | .620 | -1.6137E-009 | 2.6571E-009 |
| | | BMHDay1 | -7.06595E-010 | 9.54256E-010 | .466 | -2.6681E-009 | 1.2549E-009 |
| | | PMHDay1 | 1.13060E-010 | 9.54256E-010 | .907 | -1.8484E-009 | 2.0746E-009 |
| | | BFHDay2 | 7.39896E-010 | 8.99681E-010 | .418 | -1.1094E-009 | 2.5892E-009 |
| | | PFHDay2 | 7.29252E-010 | 9.54256E-010 | .452 | -1.2322E-009 | 2.6908E-009 |
| | | BMHDay2 | 5.26839E-010 | 9.54256E-010 | .586 | -1.4347E-009 | 2.4883E-009 |
| | | PMHDay2 | 4.41725E-010 | 8.99681E-010 | .628 | -1.4076E-009 | 2.2910E-009 |
| | BMHDay1 | BFHDay1 | 1.22833E-009 | 1.08647E-009 | .269 | -1.0049E-009 | 3.4616E-009 |
| | | PFHDay1 | 7.06595E-010 | 9.54256E-010 | .466 | -1.2549E-009 | 2.6681E-009 |
| | | PMHDay1 | 8.19654E-010 | 1.00587E-009 | .423 | -1.2479E-009 | 2.8873E-009 |

| | | | | | | |
|---|---|---|---|---|---|---|
| | BFHDay2 | 1.44649E-009 | 9.54256E-010 | .142 | -5.1501E-010 | 3.4080E-009 |
| | PFHDay2 | 1.43585E-009 | 1.00587E-009 | .165 | -6.3176E-010 | 3.5035E-009 |
| | BMHDay2 | 1.23343E-009 | 1.00587E-009 | .231 | -8.3417E-010 | 3.3010E-009 |
| | PMHDay2 | 1.14832E-009 | 9.54256E-010 | .240 | -8.1318E-010 | 3.1098E-009 |
| PMHDay1 | BFHDay1 | 4.08676E-010 | 1.08647E-009 | .710 | -1.8246E-009 | 2.6419E-009 |
| | PFHDay1 | -1.13060E-010 | 9.54256E-010 | .907 | -2.0746E-009 | 1.8484E-009 |
| | BMHDay1 | -8.19654E-010 | 1.00587E-009 | .423 | -2.8873E-009 | 1.2479E-009 |
| | BFHDay2 | 6.26836E-010 | 9.54256E-010 | .517 | -1.3347E-009 | 2.5883E-009 |
| | PFHDay2 | 6.16192E-010 | 1.00587E-009 | .545 | -1.4514E-009 | 2.6838E-009 |
| | BMHDay2 | 4.13779E-010 | 1.00587E-009 | .684 | -1.6538E-009 | 2.4814E-009 |
| | PMHDay2 | 3.28665E-010 | 9.54256E-010 | .733 | -1.6328E-009 | 2.2902E-009 |
| BFHDay2 | BFHDay1 | -2.18160E-010 | 1.03886E-009 | .835 | -2.3536E-009 | 1.9173E-009 |
| | PFHDay1 | -7.39896E-010 | 8.99681E-010 | .418 | -2.5892E-009 | 1.1094E-009 |
| | BMHDay1 | -1.44649E-009 | 9.54256E-010 | .142 | -3.4080E-009 | 5.1501E-010 |
| | PMHDay1 | -6.26836E-010 | 9.54256E-010 | .517 | -2.5883E-009 | 1.3347E-009 |
| | PFHDay2 | -1.06441E-011 | 9.54256E-010 | .991 | -1.9721E-009 | 1.9509E-009 |
| | BMHDay2 | -2.13057E-010 | 9.54256E-010 | .825 | -2.1746E-009 | 1.7484E-009 |
| | PMHDay2 | -2.98171E-010 | 8.99681E-010 | .743 | -2.1475E-009 | 1.5512E-009 |
| PFHDay2 | BFHDay1 | -2.07516E-010 | 1.08647E-009 | .850 | -2.4408E-009 | 2.0258E-009 |

| | | | | | | |
|---|---|---|---|---|---|---|
| | PFHDay1 | -7.29252E-010 | 9.54256E-010 | .452 | -2.6908E-009 | 1.2322E-009 |
| | BMHDay1 | -1.43585E-009 | 1.00587E-009 | .165 | -3.5035E-009 | 6.3176E-010 |
| | PMHDay1 | -6.16192E-010 | 1.00587E-009 | .545 | -2.6838E-009 | 1.4514E-009 |
| | BFHDay2 | 1.06441E-011 | 9.54256E-010 | .991 | -1.9509E-009 | 1.9721E-009 |
| | BMHDay2 | -2.02413E-010 | 1.00587E-009 | .842 | -2.2700E-009 | 1.8652E-009 |
| | PMHDay2 | -2.87527E-010 | 9.54256E-010 | .766 | -2.2490E-009 | 1.6740E-009 |
| BMHDay2 | BFHDay1 | -5.10350E-012 | 1.08647E-009 | .996 | -2.2384E-009 | 2.2282E-009 |
| | PFHDay1 | -5.26839E-010 | 9.54256E-010 | .586 | -2.4883E-009 | 1.4347E-009 |
| | BMHDay1 | -1.23343E-009 | 1.00587E-009 | .231 | -3.3010E-009 | 8.3417E-010 |
| | PMHDay1 | -4.13779E-010 | 1.00587E-009 | .684 | -2.4814E-009 | 1.6538E-009 |
| | BFHDay2 | 2.13057E-010 | 9.54256E-010 | .825 | -1.7484E-009 | 2.1746E-009 |
| | PFHDay2 | 2.02413E-010 | 1.00587E-009 | .842 | -1.8652E-009 | 2.2700E-009 |
| | PMHDay2 | -8.51141E-011 | 9.54256E-010 | .930 | -2.0466E-009 | 1.8764E-009 |
| PMHDay2 | BFHDay1 | 8.00106E-011 | 1.03886E-009 | .939 | -2.0554E-009 | 2.2154E-009 |
| | PFHDay1 | -4.41725E-010 | 8.99681E-010 | .628 | -2.2910E-009 | 1.4076E-009 |
| | BMHDay1 | -1.14832E-009 | 9.54256E-010 | .240 | -3.1098E-009 | 8.1318E-010 |
| | PMHDay1 | -3.28665E-010 | 9.54256E-010 | .733 | -2.2902E-009 | 1.6328E-009 |
| | BFHDay2 | 2.98171E-010 | 8.99681E-010 | .743 | -1.5512E-009 | 2.1475E-009 |
| | PFHDay2 | 2.87527E-010 | 9.54256E-010 | .766 | -1.6740E-009 | 2.2490E-009 |

| | | | | | | | |
|---|---|---|---|---|---|---|---|
| | | BMHDay2 | 8.51141E-011 | 9.54256E-010 | .930 | -1.8764E-009 | 2.0466E-009 |
| palSerine | BFHDay1 | PFHDay1 | -2.97012E-012 | 1.85883E-012 | .121 | -6.7719E-012 | 8.3162E-013 |
| | | BMHDay1 | 4.21036E-013 | 1.85883E-012 | .822 | -3.3807E-012 | 4.2228E-012 |
| | | PMHDay1 | -4.54317E-013 | 1.75252E-012 | .797 | -4.0386E-012 | 3.1300E-012 |
| | | BFHDay2 | 4.96662E-013 | 1.75252E-012 | .779 | -3.0877E-012 | 4.0810E-012 |
| | | PFHDay2 | 3.22200E-013 | 1.85883E-012 | .864 | -3.4795E-012 | 4.1239E-012 |
| | | BMHDay2 | -1.50197E-012 | 1.75252E-012 | .398 | -5.0863E-012 | 2.0823E-012 |
| | | PMHDay2 | -6.35536E-013 | 1.75252E-012 | .720 | -4.2198E-012 | 2.9488E-012 |
| | PFHDay1 | BFHDay1 | 2.97012E-012 | 1.85883E-012 | .121 | -8.3162E-013 | 6.7719E-012 |
| | | BMHDay1 | 3.39116E-012 | 1.95938E-012 | .094 | -6.1623E-013 | 7.3985E-012 |
| | | PMHDay1 | 2.51580E-012 | 1.85883E-012 | .186 | -1.2859E-012 | 6.3175E-012 |
| | | BFHDay2 | 3.46678E-012 | 1.85883E-012 | .072 | -3.3496E-013 | 7.2685E-012 |
| | | PFHDay2 | 3.29232E-012 | 1.95938E-012 | .104 | -7.1506E-013 | 7.2997E-012 |
| | | BMHDay2 | 1.46815E-012 | 1.85883E-012 | .436 | -2.3336E-012 | 5.2699E-012 |
| | | PMHDay2 | 2.33458E-012 | 1.85883E-012 | .219 | -1.4672E-012 | 6.1363E-012 |
| | BMHDay1 | BFHDay1 | -4.21036E-013 | 1.85883E-012 | .822 | -4.2228E-012 | 3.3807E-012 |
| | | PFHDay1 | -3.39116E-012 | 1.95938E-012 | .094 | -7.3985E-012 | 6.1623E-013 |
| | | PMHDay1 | -8.75354E-013 | 1.85883E-012 | .641 | -4.6771E-012 | 2.9264E-012 |
| | | BFHDay2 | 7.56258E-014 | 1.85883E-012 | .968 | -3.7261E-012 | 3.8774E-012 |

| | | | | | | |
|---|---|---|---|---|---|---|
| | PFHDay2 | -9.88367E-014 | 1.95938E-012 | .960 | -4.1062E-012 | 3.9085E-012 |
| | BMHDay2 | -1.92301E-012 | 1.85883E-012 | .309 | -5.7247E-012 | 1.8787E-012 |
| | PMHDay2 | -1.05657E-012 | 1.85883E-012 | .574 | -4.8583E-012 | 2.7452E-012 |
| PMHDay1 | BFHDay1 | 4.54317E-013 | 1.75252E-012 | .797 | -3.1300E-012 | 4.0386E-012 |
| | PFHDay1 | -2.51580E-012 | 1.85883E-012 | .186 | -6.3175E-012 | 1.2859E-012 |
| | BMHDay1 | 8.75354E-013 | 1.85883E-012 | .641 | -2.9264E-012 | 4.6771E-012 |
| | BFHDay2 | 9.50979E-013 | 1.75252E-012 | .592 | -2.6333E-012 | 4.5353E-012 |
| | PFHDay2 | 7.76517E-013 | 1.85883E-012 | .679 | -3.0252E-012 | 4.5783E-012 |
| | BMHDay2 | -1.04765E-012 | 1.75252E-012 | .555 | -4.6320E-012 | 2.5367E-012 |
| | PMHDay2 | -1.81219E-013 | 1.75252E-012 | .918 | -3.7655E-012 | 3.4031E-012 |
| BFHDay2 | BFHDay1 | -4.96662E-013 | 1.75252E-012 | .779 | -4.0810E-012 | 3.0877E-012 |
| | PFHDay1 | -3.46678E-012 | 1.85883E-012 | .072 | -7.2685E-012 | 3.3496E-013 |
| | BMHDay1 | -7.56258E-014 | 1.85883E-012 | .968 | -3.8774E-012 | 3.7261E-012 |
| | PMHDay1 | -9.50979E-013 | 1.75252E-012 | .592 | -4.5353E-012 | 2.6333E-012 |
| | PFHDay2 | -1.74463E-013 | 1.85883E-012 | .926 | -3.9762E-012 | 3.6273E-012 |
| | BMHDay2 | -1.99863E-012 | 1.75252E-012 | .263 | -5.5829E-012 | 1.5857E-012 |
| | PMHDay2 | -1.13220E-012 | 1.75252E-012 | .523 | -4.7165E-012 | 2.4521E-012 |
| PFHDay2 | BFHDay1 | -3.22200E-013 | 1.85883E-012 | .864 | -4.1239E-012 | 3.4795E-012 |
| | PFHDay1 | -3.29232E-012 | 1.95938E-012 | .104 | -7.2997E-012 | 7.1506E-013 |

| | | | | | | |
|---|---|---|---|---|---|---|
| | BMHDay1 | 9.88367E-014 | 1.95938E-012 | .960 | -3.9085E-012 | 4.1062E-012 |
| | PMHDay1 | -7.76517E-013 | 1.85883E-012 | .679 | -4.5783E-012 | 3.0252E-012 |
| | BFHDay2 | 1.74463E-013 | 1.85883E-012 | .926 | -3.6273E-012 | 3.9762E-012 |
| | BMHDay2 | -1.82417E-012 | 1.85883E-012 | .335 | -5.6259E-012 | 1.9776E-012 |
| | PMHDay2 | -9.57735E-013 | 1.85883E-012 | .610 | -4.7595E-012 | 2.8440E-012 |
| BMHDay2 | BFHDay1 | 1.50197E-012 | 1.75252E-012 | .398 | -2.0823E-012 | 5.0863E-012 |
| | PFHDay1 | -1.46815E-012 | 1.85883E-012 | .436 | -5.2699E-012 | 2.3336E-012 |
| | BMHDay1 | 1.92301E-012 | 1.85883E-012 | .309 | -1.8787E-012 | 5.7247E-012 |
| | PMHDay1 | 1.04765E-012 | 1.75252E-012 | .555 | -2.5367E-012 | 4.6320E-012 |
| | BFHDay2 | 1.99863E-012 | 1.75252E-012 | .263 | -1.5857E-012 | 5.5829E-012 |
| | PFHDay2 | 1.82417E-012 | 1.85883E-012 | .335 | -1.9776E-012 | 5.6259E-012 |
| | PMHDay2 | 8.66435E-013 | 1.75252E-012 | .625 | -2.7179E-012 | 4.4507E-012 |
| PMHDay2 | BFHDay1 | 6.35536E-013 | 1.75252E-012 | .720 | -2.9488E-012 | 4.2198E-012 |
| | PFHDay1 | -2.33458E-012 | 1.85883E-012 | .219 | -6.1363E-012 | 1.4672E-012 |
| | BMHDay1 | 1.05657E-012 | 1.85883E-012 | .574 | -2.7452E-012 | 4.8583E-012 |
| | PMHDay1 | 1.81219E-013 | 1.75252E-012 | .918 | -3.4031E-012 | 3.7655E-012 |
| | BFHDay2 | 1.13220E-012 | 1.75252E-012 | .523 | -2.4521E-012 | 4.7165E-012 |
| | PFHDay2 | 9.57735E-013 | 1.85883E-012 | .610 | -2.8440E-012 | 4.7595E-012 |
| | BMHDay2 | -8.66435E-013 | 1.75252E-012 | .625 | -4.4507E-012 | 2.7179E-012 |

| | | | | | | | |
|---|---|---|---|---|---|---|---|
| steSerine | BFHDay1 | PFHDay1 | 2.97026E-012 | 1.07795E-011 | .785 | -1.9015E-011 | 2.4955E-011 |
| | | BMHDay1 | -7.15314E-013 | 1.14334E-011 | .951 | -2.4034E-011 | 2.2603E-011 |
| | | PMHDay1 | -6.52349E-012 | 1.07795E-011 | .549 | -2.8508E-011 | 1.5461E-011 |
| | | BFHDay2 | 6.19177E-012 | 1.07795E-011 | .570 | -1.5793E-011 | 2.8177E-011 |
| | | PFHDay2 | -6.00950E-012 | 1.07795E-011 | .581 | -2.7994E-011 | 1.5975E-011 |
| | | BMHDay2 | 6.73610E-012 | 1.07795E-011 | .537 | -1.5249E-011 | 2.8721E-011 |
| | | PMHDay2 | 5.99123E-012 | 1.07795E-011 | .582 | -1.5994E-011 | 2.7976E-011 |
| | PFHDay1 | BFHDay1 | -2.97026E-012 | 1.07795E-011 | .785 | -2.4955E-011 | 1.9015E-011 |
| | | BMHDay1 | -3.68557E-012 | 1.14334E-011 | .749 | -2.7004E-011 | 1.9633E-011 |
| | | PMHDay1 | -9.49375E-012 | 1.07795E-011 | .385 | -3.1479E-011 | 1.2491E-011 |
| | | BFHDay2 | 3.22152E-012 | 1.07795E-011 | .767 | -1.8763E-011 | 2.5206E-011 |
| | | PFHDay2 | -8.97975E-012 | 1.07795E-011 | .411 | -3.0965E-011 | 1.3005E-011 |
| | | BMHDay2 | 3.76585E-012 | 1.07795E-011 | .729 | -1.8219E-011 | 2.5751E-011 |
| | | PMHDay2 | 3.02097E-012 | 1.07795E-011 | .781 | -1.8964E-011 | 2.5006E-011 |
| | BMHDay1 | BFHDay1 | 7.15314E-013 | 1.14334E-011 | .951 | -2.2603E-011 | 2.4034E-011 |
| | | PFHDay1 | 3.68557E-012 | 1.14334E-011 | .749 | -1.9633E-011 | 2.7004E-011 |
| | | PMHDay1 | -5.80818E-012 | 1.14334E-011 | .615 | -2.9127E-011 | 1.7510E-011 |
| | | BFHDay2 | 6.90709E-012 | 1.14334E-011 | .550 | -1.6411E-011 | 3.0226E-011 |
| | | PFHDay2 | -5.29418E-012 | 1.14334E-011 | .647 | -2.8613E-011 | 1.8024E-011 |

| | | | | | | |
|---|---|---|---|---|---|---|
| | BMHDay2 | 7.45142E-012 | 1.14334E-011 | .519 | -1.5867E-011 | 3.0770E-011 |
| | PMHDay2 | 6.70654E-012 | 1.14334E-011 | .562 | -1.6612E-011 | 3.0025E-011 |
| PMHDay1 | BFHDay1 | 6.52349E-012 | 1.07795E-011 | .549 | -1.5461E-011 | 2.8508E-011 |
| | PFHDay1 | 9.49375E-012 | 1.07795E-011 | .385 | -1.2491E-011 | 3.1479E-011 |
| | BMHDay1 | 5.80818E-012 | 1.14334E-011 | .615 | -1.7510E-011 | 2.9127E-011 |
| | BFHDay2 | 1.27153E-011 | 1.07795E-011 | .247 | -9.2696E-012 | 3.4700E-011 |
| | PFHDay2 | 5.13993E-013 | 1.07795E-011 | .962 | -2.1471E-011 | 2.2499E-011 |
| | BMHDay2 | 1.32596E-011 | 1.07795E-011 | .228 | -8.7253E-012 | 3.5244E-011 |
| | PMHDay2 | 1.25147E-011 | 1.07795E-011 | .255 | -9.4702E-012 | 3.4500E-011 |
| BFHDay2 | BFHDay1 | -6.19177E-012 | 1.07795E-011 | .570 | -2.8177E-011 | 1.5793E-011 |
| | PFHDay1 | -3.22152E-012 | 1.07795E-011 | .767 | -2.5206E-011 | 1.8763E-011 |
| | BMHDay1 | -6.90709E-012 | 1.14334E-011 | .550 | -3.0226E-011 | 1.6411E-011 |
| | PMHDay1 | -1.27153E-011 | 1.07795E-011 | .247 | -3.4700E-011 | 9.2696E-012 |
| | PFHDay2 | -1.22013E-011 | 1.07795E-011 | .266 | -3.4186E-011 | 9.7836E-012 |
| | BMHDay2 | 5.44332E-013 | 1.07795E-011 | .960 | -2.1441E-011 | 2.2529E-011 |
| | PMHDay2 | -2.00545E-013 | 1.07795E-011 | .985 | -2.2185E-011 | 2.1784E-011 |
| PFHDay2 | BFHDay1 | 6.00950E-012 | 1.07795E-011 | .581 | -1.5975E-011 | 2.7994E-011 |
| | PFHDay1 | 8.97975E-012 | 1.07795E-011 | .411 | -1.3005E-011 | 3.0965E-011 |
| | BMHDay1 | 5.29418E-012 | 1.14334E-011 | .647 | -1.8024E-011 | 2.8613E-011 |

| | | | | | | | |
|---|---|---|---|---|---|---|---|
| | | PMHDay1 | -5.13993E-013 | 1.07795E-011 | .962 | -2.2499E-011 | 2.1471E-011 |
| | | BFHDay2 | 1.22013E-011 | 1.07795E-011 | .266 | -9.7836E-012 | 3.4186E-011 |
| | | BMHDay2 | 1.27456E-011 | 1.07795E-011 | .246 | -9.2393E-012 | 3.4730E-011 |
| | | PMHDay2 | 1.20007E-011 | 1.07795E-011 | .274 | -9.9841E-012 | 3.3986E-011 |
| | BMHDay2 | BFHDay1 | -6.73610E-012 | 1.07795E-011 | .537 | -2.8721E-011 | 1.5249E-011 |
| | | PFHDay1 | -3.76585E-012 | 1.07795E-011 | .729 | -2.5751E-011 | 1.8219E-011 |
| | | BMHDay1 | -7.45142E-012 | 1.14334E-011 | .519 | -3.0770E-011 | 1.5867E-011 |
| | | PMHDay1 | -1.32596E-011 | 1.07795E-011 | .228 | -3.5244E-011 | 8.7253E-012 |
| | | BFHDay2 | -5.44332E-013 | 1.07795E-011 | .960 | -2.2529E-011 | 2.1441E-011 |
| | | PFHDay2 | -1.27456E-011 | 1.07795E-011 | .246 | -3.4730E-011 | 9.2393E-012 |
| | | PMHDay2 | -7.44878E-013 | 1.07795E-011 | .945 | -2.2730E-011 | 2.1240E-011 |
| | PMHDay2 | BFHDay1 | -5.99123E-012 | 1.07795E-011 | .582 | -2.7976E-011 | 1.5994E-011 |
| | | PFHDay1 | -3.02097E-012 | 1.07795E-011 | .781 | -2.5006E-011 | 1.8964E-011 |
| | | BMHDay1 | -6.70654E-012 | 1.14334E-011 | .562 | -3.0025E-011 | 1.6612E-011 |
| | | PMHDay1 | -1.25147E-011 | 1.07795E-011 | .255 | -3.4500E-011 | 9.4702E-012 |
| | | BFHDay2 | 2.00545E-013 | 1.07795E-011 | .985 | -2.1784E-011 | 2.2185E-011 |
| | | PFHDay2 | -1.20007E-011 | 1.07795E-011 | .274 | -3.3986E-011 | 9.9841E-012 |
| | | BMHDay2 | 7.44878E-013 | 1.07795E-011 | .945 | -2.1240E-011 | 2.2730E-011 |
| oleSerine | BFHDay1 | PFHDay1 | -1.21971E-013 | 1.07684E-012 | .911 | -2.3496E-012 | 2.1056E-012 |

| | | | | | | |
|---|---|---|---|---|---|---|
| | BMHDay1 | 1.44353E-013 | 1.13508E-012 | .900 | -2.2037E-012 | 2.4925E-012 |
| | PMHDay1 | -1.11832E-012 | 1.07684E-012 | .310 | -3.3459E-012 | 1.1093E-012 |
| | BFHDay2 | 2.37100E-013 | 1.39019E-012 | .866 | -2.6387E-012 | 3.1129E-012 |
| | PFHDay2 | -3.10859E-013 | 1.13508E-012 | .787 | -2.6590E-012 | 2.0372E-012 |
| | BMHDay2 | -3.23809E-012* | 1.22603E-012 | .015 | -5.7743E-012 | -7.0185E-013 |
| | PMHDay2 | -1.82715E-012 | 1.13508E-012 | .121 | -4.1753E-012 | 5.2095E-013 |
| PFHDay1 | BFHDay1 | 1.21971E-013 | 1.07684E-012 | .911 | -2.1056E-012 | 2.3496E-012 |
| | BMHDay1 | 2.66325E-013 | 1.07684E-012 | .807 | -1.9613E-012 | 2.4939E-012 |
| | PMHDay1 | -9.96351E-013 | 1.01525E-012 | .337 | -3.0966E-012 | 1.1039E-012 |
| | BFHDay2 | 3.59071E-013 | 1.34305E-012 | .792 | -2.4192E-012 | 3.1374E-012 |
| | PFHDay2 | -1.88888E-013 | 1.07684E-012 | .862 | -2.4165E-012 | 2.0387E-012 |
| | BMHDay2 | -3.11612E-012* | 1.17231E-012 | .014 | -5.5412E-012 | -6.9101E-013 |
| | PMHDay2 | -1.70518E-012 | 1.07684E-012 | .127 | -3.9328E-012 | 5.2242E-013 |
| BMHDay1 | BFHDay1 | -1.44353E-013 | 1.13508E-012 | .900 | -2.4925E-012 | 2.2037E-012 |
| | PFHDay1 | -2.66325E-013 | 1.07684E-012 | .807 | -2.4939E-012 | 1.9613E-012 |
| | PMHDay1 | -1.26268E-012 | 1.07684E-012 | .253 | -3.4903E-012 | 9.6493E-013 |
| | BFHDay2 | 9.27469E-014 | 1.39019E-012 | .947 | -2.7831E-012 | 2.9686E-012 |
| | PFHDay2 | -4.55212E-013 | 1.13508E-012 | .692 | -2.8033E-012 | 1.8929E-012 |
| | BMHDay2 | -3.38244E-012* | 1.22603E-012 | .011 | -5.9187E-012 | -8.4620E-013 |

| | | | | | | | |
|---|---|---|---|---|---|---|---|
| | | PMHDay2 | -1.97151E-012 | 1.13508E-012 | .096 | -4.3196E-012 | 3.7660E-013 |
| | PMHDay1 | BFHDay1 | 1.11832E-012 | 1.07684E-012 | .310 | -1.1093E-012 | 3.3459E-012 |
| | | PFHDay1 | 9.96351E-013 | 1.01525E-012 | .337 | -1.1039E-012 | 3.0966E-012 |
| | | BMHDay1 | 1.26268E-012 | 1.07684E-012 | .253 | -9.6493E-013 | 3.4903E-012 |
| | | BFHDay2 | 1.35542E-012 | 1.34305E-012 | .323 | -1.4229E-012 | 4.1337E-012 |
| | | PFHDay2 | 8.07463E-013 | 1.07684E-012 | .461 | -1.4201E-012 | 3.0351E-012 |
| | | BMHDay2 | -2.11977E-012 | 1.17231E-012 | .084 | -4.5449E-012 | 3.0534E-013 |
| | | PMHDay2 | -7.08831E-013 | 1.07684E-012 | .517 | -2.9364E-012 | 1.5188E-012 |
| | BFHDay2 | BFHDay1 | -2.37100E-013 | 1.39019E-012 | .866 | -3.1129E-012 | 2.6387E-012 |
| | | PFHDay1 | -3.59071E-013 | 1.34305E-012 | .792 | -3.1374E-012 | 2.4192E-012 |
| | | BMHDay1 | -9.27469E-014 | 1.39019E-012 | .947 | -2.9686E-012 | 2.7831E-012 |
| | | PMHDay1 | -1.35542E-012 | 1.34305E-012 | .323 | -4.1337E-012 | 1.4229E-012 |
| | | PFHDay2 | -5.47959E-013 | 1.39019E-012 | .697 | -3.4238E-012 | 2.3279E-012 |
| | | BMHDay2 | -3.47519E-012* | 1.46539E-012 | .026 | -6.5066E-012 | -4.4380E-013 |
| | | PMHDay2 | -2.06425E-012 | 1.39019E-012 | .151 | -4.9401E-012 | 8.1157E-013 |
| | PFHDay2 | BFHDay1 | 3.10859E-013 | 1.13508E-012 | .787 | -2.0372E-012 | 2.6590E-012 |
| | | PFHDay1 | 1.88888E-013 | 1.07684E-012 | .862 | -2.0387E-012 | 2.4165E-012 |
| | | BMHDay1 | 4.55212E-013 | 1.13508E-012 | .692 | -1.8929E-012 | 2.8033E-012 |
| | | PMHDay1 | -8.07463E-013 | 1.07684E-012 | .461 | -3.0351E-012 | 1.4201E-012 |

| | | | | | | | |
|---|---|---|---|---|---|---|---|
| | | BFHDay2 | 5.47959E-013 | 1.39019E-012 | .697 | -2.3279E-012 | 3.4238E-012 |
| | | BMHDay2 | -2.92723E-012* | 1.22603E-012 | .026 | -5.4635E-012 | -3.9099E-013 |
| | | PMHDay2 | -1.51629E-012 | 1.13508E-012 | .195 | -3.8644E-012 | 8.3181E-013 |
| | BMHDay2 | BFHDay1 | 3.23809E-012* | 1.22603E-012 | .015 | 7.0185E-013 | 5.7743E-012 |
| | | PFHDay1 | 3.11612E-012* | 1.17231E-012 | .014 | 6.9101E-013 | 5.5412E-012 |
| | | BMHDay1 | 3.38244E-012* | 1.22603E-012 | .011 | 8.4620E-013 | 5.9187E-012 |
| | | PMHDay1 | 2.11977E-012 | 1.17231E-012 | .084 | -3.0534E-013 | 4.5449E-012 |
| | | BFHDay2 | 3.47519E-012* | 1.46539E-012 | .026 | 4.4380E-013 | 6.5066E-012 |
| | | PFHDay2 | 2.92723E-012* | 1.22603E-012 | .026 | 3.9099E-013 | 5.4635E-012 |
| | | PMHDay2 | 1.41094E-012 | 1.22603E-012 | .262 | -1.1253E-012 | 3.9472E-012 |
| | PMHDay2 | BFHDay1 | 1.82715E-012 | 1.13508E-012 | .121 | -5.2095E-013 | 4.1753E-012 |
| | | PFHDay1 | 1.70518E-012 | 1.07684E-012 | .127 | -5.2242E-013 | 3.9328E-012 |
| | | BMHDay1 | 1.97151E-012 | 1.13508E-012 | .096 | -3.7660E-013 | 4.3196E-012 |
| | | PMHDay1 | 7.08831E-013 | 1.07684E-012 | .517 | -1.5188E-012 | 2.9364E-012 |
| | | BFHDay2 | 2.06425E-012 | 1.39019E-012 | .151 | -8.1157E-013 | 4.9401E-012 |
| | | PFHDay2 | 1.51629E-012 | 1.13508E-012 | .195 | -8.3181E-013 | 3.8644E-012 |
| | | BMHDay2 | -1.41094E-012 | 1.22603E-012 | .262 | -3.9472E-012 | 1.1253E-012 |
| palTrypt | BFHDay1 | PFHDay1 | 9.27960E-014 | 1.35945E-012 | .946 | -2.7343E-012 | 2.9199E-012 |
| | | BMHDay1 | 5.80180E-013 | 1.42175E-012 | .687 | -2.3765E-012 | 3.5369E-012 |

| | | | | | | |
|---|---|---|---|---|---|---|
| | PMHDay1 | -2.10140E-013 | 1.42175E-012 | .884 | -3.1668E-012 | 2.7465E-012 |
| | BFHDay2 | 2.94537E-013 | 1.51991E-012 | .848 | -2.8663E-012 | 3.4554E-012 |
| | PFHDay2 | 3.70108E-013 | 1.42175E-012 | .797 | -2.5866E-012 | 3.3268E-012 |
| | BMHDay2 | 5.12425E-013 | 1.69931E-012 | .766 | -3.0215E-012 | 4.0463E-012 |
| | PMHDay2 | -1.49233E-012 | 1.42175E-012 | .306 | -4.4490E-012 | 1.4644E-012 |
| PFHDay1 | BFHDay1 | -9.27960E-014 | 1.35945E-012 | .946 | -2.9199E-012 | 2.7343E-012 |
| | BMHDay1 | 4.87384E-013 | 1.24873E-012 | .700 | -2.1095E-012 | 3.0843E-012 |
| | PMHDay1 | -3.02936E-013 | 1.24873E-012 | .811 | -2.8998E-012 | 2.2939E-012 |
| | BFHDay2 | 2.01741E-013 | 1.35945E-012 | .883 | -2.6254E-012 | 3.0289E-012 |
| | PFHDay2 | 2.77312E-013 | 1.24873E-012 | .826 | -2.3196E-012 | 2.8742E-012 |
| | BMHDay2 | 4.19629E-013 | 1.55745E-012 | .790 | -2.8193E-012 | 3.6585E-012 |
| | PMHDay2 | -1.58512E-012 | 1.24873E-012 | .218 | -4.1820E-012 | 1.0118E-012 |
| BMHDay1 | BFHDay1 | -5.80180E-013 | 1.42175E-012 | .687 | -3.5369E-012 | 2.3765E-012 |
| | PFHDay1 | -4.87384E-013 | 1.24873E-012 | .700 | -3.0843E-012 | 2.1095E-012 |
| | PMHDay1 | -7.90320E-013 | 1.31628E-012 | .555 | -3.5277E-012 | 1.9470E-012 |
| | BFHDay2 | -2.85643E-013 | 1.42175E-012 | .843 | -3.2423E-012 | 2.6710E-012 |
| | PFHDay2 | -2.10073E-013 | 1.31628E-012 | .875 | -2.9474E-012 | 2.5273E-012 |
| | BMHDay2 | -6.77550E-014 | 1.61211E-012 | .967 | -3.4203E-012 | 3.2848E-012 |
| | PMHDay2 | -2.07251E-012 | 1.31628E-012 | .130 | -4.8099E-012 | 6.6485E-013 |

| | | | | | | |
|---|---|---|---|---|---|---|
| PMHDay1 | BFHDay1 | 2.10140E-013 | 1.42175E-012 | .884 | -2.7465E-012 | 3.1668E-012 |
| | PFHDay1 | 3.02936E-013 | 1.24873E-012 | .811 | -2.2939E-012 | 2.8998E-012 |
| | BMHDay1 | 7.90320E-013 | 1.31628E-012 | .555 | -1.9470E-012 | 3.5277E-012 |
| | BFHDay2 | 5.04677E-013 | 1.42175E-012 | .726 | -2.4520E-012 | 3.4614E-012 |
| | PFHDay2 | 5.80248E-013 | 1.31628E-012 | .664 | -2.1571E-012 | 3.3176E-012 |
| | BMHDay2 | 7.22565E-013 | 1.61211E-012 | .659 | -2.6300E-012 | 4.0751E-012 |
| | PMHDay2 | -1.28219E-012 | 1.31628E-012 | .341 | -4.0195E-012 | 1.4552E-012 |
| BFHDay2 | BFHDay1 | -2.94537E-013 | 1.51991E-012 | .848 | -3.4554E-012 | 2.8663E-012 |
| | PFHDay1 | -2.01741E-013 | 1.35945E-012 | .883 | -3.0289E-012 | 2.6254E-012 |
| | BMHDay1 | 2.85643E-013 | 1.42175E-012 | .843 | -2.6710E-012 | 3.2423E-012 |
| | PMHDay1 | -5.04677E-013 | 1.42175E-012 | .726 | -3.4614E-012 | 2.4520E-012 |
| | PFHDay2 | 7.55708E-014 | 1.42175E-012 | .958 | -2.8811E-012 | 3.0323E-012 |
| | BMHDay2 | 2.17888E-013 | 1.69931E-012 | .899 | -3.3160E-012 | 3.7518E-012 |
| | PMHDay2 | -1.78686E-012 | 1.42175E-012 | .223 | -4.7435E-012 | 1.1698E-012 |
| PFHDay2 | BFHDay1 | -3.70108E-013 | 1.42175E-012 | .797 | -3.3268E-012 | 2.5866E-012 |
| | PFHDay1 | -2.77312E-013 | 1.24873E-012 | .826 | -2.8742E-012 | 2.3196E-012 |
| | BMHDay1 | 2.10073E-013 | 1.31628E-012 | .875 | -2.5273E-012 | 2.9474E-012 |
| | PMHDay1 | -5.80248E-013 | 1.31628E-012 | .664 | -3.3176E-012 | 2.1571E-012 |
| | BFHDay2 | -7.55708E-014 | 1.42175E-012 | .958 | -3.0323E-012 | 2.8811E-012 |

| | | | | | | | |
|---|---|---|---|---|---|---|---|
| | | BMHDay2 | 1.42317E-013 | 1.61211E-012 | .930 | -3.2102E-012 | 3.4949E-012 |
| | | PMHDay2 | -1.86244E-012 | 1.31628E-012 | .172 | -4.5998E-012 | 8.7492E-013 |
| | BMHDay2 | BFHDay1 | -5.12425E-013 | 1.69931E-012 | .766 | -4.0463E-012 | 3.0215E-012 |
| | | PFHDay1 | -4.19629E-013 | 1.55745E-012 | .790 | -3.6585E-012 | 2.8193E-012 |
| | | BMHDay1 | 6.77550E-014 | 1.61211E-012 | .967 | -3.2848E-012 | 3.4203E-012 |
| | | PMHDay1 | -7.22565E-013 | 1.61211E-012 | .659 | -4.0751E-012 | 2.6300E-012 |
| | | BFHDay2 | -2.17888E-013 | 1.69931E-012 | .899 | -3.7518E-012 | 3.3160E-012 |
| | | PFHDay2 | -1.42317E-013 | 1.61211E-012 | .930 | -3.4949E-012 | 3.2102E-012 |
| | | PMHDay2 | -2.00475E-012 | 1.61211E-012 | .227 | -5.3573E-012 | 1.3478E-012 |
| | PMHDay2 | BFHDay1 | 1.49233E-012 | 1.42175E-012 | .306 | -1.4644E-012 | 4.4490E-012 |
| | | PFHDay1 | 1.58512E-012 | 1.24873E-012 | .218 | -1.0118E-012 | 4.1820E-012 |
| | | BMHDay1 | 2.07251E-012 | 1.31628E-012 | .130 | -6.6485E-013 | 4.8099E-012 |
| | | PMHDay1 | 1.28219E-012 | 1.31628E-012 | .341 | -1.4552E-012 | 4.0195E-012 |
| | | BFHDay2 | 1.78686E-012 | 1.42175E-012 | .223 | -1.1698E-012 | 4.7435E-012 |
| | | PFHDay2 | 1.86244E-012 | 1.31628E-012 | .172 | -8.7492E-013 | 4.5998E-012 |
| | | BMHDay2 | 2.00475E-012 | 1.61211E-012 | .227 | -1.3478E-012 | 5.3573E-012 |
| steTrypt | BFHDay1 | PFHDay1 | 2.86395E-012 | 2.77469E-012 | .311 | -2.8197E-012 | 8.5476E-012 |
| | | BMHDay1 | 2.33384E-012 | 2.77469E-012 | .407 | -3.3499E-012 | 8.0175E-012 |
| | | PMHDay1 | 1.34788E-012 | 2.77469E-012 | .631 | -4.3358E-012 | 7.0316E-012 |

| | | | | | | |
|---|---|---|---|---|---|---|
| | BFHDay2 | 2.45797E-012 | 2.61600E-012 | .355 | -2.9007E-012 | 7.8166E-012 |
| | PFHDay2 | 2.15300E-012 | 2.77469E-012 | .444 | -3.5307E-012 | 7.8367E-012 |
| | BMHDay2 | 2.09817E-012 | 2.61600E-012 | .429 | -3.2605E-012 | 7.4568E-012 |
| | PMHDay2 | 3.30698E-012 | 2.61600E-012 | .217 | -2.0517E-012 | 8.6656E-012 |
| PFHDay1 | BFHDay1 | -2.86395E-012 | 2.77469E-012 | .311 | -8.5476E-012 | 2.8197E-012 |
| | BMHDay1 | -5.30112E-013 | 2.92478E-012 | .857 | -6.5212E-012 | 5.4610E-012 |
| | PMHDay1 | -1.51607E-012 | 2.92478E-012 | .608 | -7.5072E-012 | 4.4751E-012 |
| | BFHDay2 | -4.05982E-013 | 2.77469E-012 | .885 | -6.0897E-012 | 5.2777E-012 |
| | PFHDay2 | -7.10950E-013 | 2.92478E-012 | .810 | -6.7021E-012 | 5.2802E-012 |
| | BMHDay2 | -7.65778E-013 | 2.77469E-012 | .785 | -6.4495E-012 | 4.9179E-012 |
| | PMHDay2 | 4.43034E-013 | 2.77469E-012 | .874 | -5.2407E-012 | 6.1267E-012 |
| BMHDay1 | BFHDay1 | -2.33384E-012 | 2.77469E-012 | .407 | -8.0175E-012 | 3.3499E-012 |
| | PFHDay1 | 5.30112E-013 | 2.92478E-012 | .857 | -5.4610E-012 | 6.5212E-012 |
| | PMHDay1 | -9.85960E-013 | 2.92478E-012 | .739 | -6.9771E-012 | 5.0052E-012 |
| | BFHDay2 | 1.24130E-013 | 2.77469E-012 | .965 | -5.5596E-012 | 5.8078E-012 |
| | PFHDay2 | -1.80838E-013 | 2.92478E-012 | .951 | -6.1720E-012 | 5.8103E-012 |
| | BMHDay2 | -2.35666E-013 | 2.77469E-012 | .933 | -5.9194E-012 | 5.4480E-012 |
| | PMHDay2 | 9.73146E-013 | 2.77469E-012 | .728 | -4.7105E-012 | 6.6568E-012 |
| PMHDay1 | BFHDay1 | -1.34788E-012 | 2.77469E-012 | .631 | -7.0316E-012 | 4.3358E-012 |

| | | | | | | |
|---|---|---|---|---|---|---|
| | PFHDay1 | 1.51607E-012 | 2.92478E-012 | .608 | -4.4751E-012 | 7.5072E-012 |
| | BMHDay1 | 9.85960E-013 | 2.92478E-012 | .739 | -5.0052E-012 | 6.9771E-012 |
| | BFHDay2 | 1.11009E-012 | 2.77469E-012 | .692 | -4.5736E-012 | 6.7938E-012 |
| | PFHDay2 | 8.05123E-013 | 2.92478E-012 | .785 | -5.1860E-012 | 6.7963E-012 |
| | BMHDay2 | 7.50294E-013 | 2.77469E-012 | .789 | -4.9334E-012 | 6.4340E-012 |
| | PMHDay2 | 1.95911E-012 | 2.77469E-012 | .486 | -3.7246E-012 | 7.6428E-012 |
| BFHDay2 | BFHDay1 | -2.45797E-012 | 2.61600E-012 | .355 | -7.8166E-012 | 2.9007E-012 |
| | PFHDay1 | 4.05982E-013 | 2.77469E-012 | .885 | -5.2777E-012 | 6.0897E-012 |
| | BMHDay1 | -1.24130E-013 | 2.77469E-012 | .965 | -5.8078E-012 | 5.5596E-012 |
| | PMHDay1 | -1.11009E-012 | 2.77469E-012 | .692 | -6.7938E-012 | 4.5736E-012 |
| | PFHDay2 | -3.04967E-013 | 2.77469E-012 | .913 | -5.9887E-012 | 5.3787E-012 |
| | BMHDay2 | -3.59796E-013 | 2.61600E-012 | .892 | -5.7184E-012 | 4.9988E-012 |
| | PMHDay2 | 8.49016E-013 | 2.61600E-012 | .748 | -4.5096E-012 | 6.2077E-012 |
| PFHDay2 | BFHDay1 | -2.15300E-012 | 2.77469E-012 | .444 | -7.8367E-012 | 3.5307E-012 |
| | PFHDay1 | 7.10950E-013 | 2.92478E-012 | .810 | -5.2802E-012 | 6.7021E-012 |
| | BMHDay1 | 1.80838E-013 | 2.92478E-012 | .951 | -5.8103E-012 | 6.1720E-012 |
| | PMHDay1 | -8.05123E-013 | 2.92478E-012 | .785 | -6.7963E-012 | 5.1860E-012 |
| | BFHDay2 | 3.04967E-013 | 2.77469E-012 | .913 | -5.3787E-012 | 5.9887E-012 |
| | BMHDay2 | -5.48285E-014 | 2.77469E-012 | .984 | -5.7385E-012 | 5.6289E-012 |

| | | | | | | | |
|---|---|---|---|---|---|---|---|
| | | PMHDay2 | 1.15398E-012 | 2.77469E-012 | .681 | -4.5297E-012 | 6.8377E-012 |
| | BMHDay2 | BFHDay1 | -2.09817E-012 | 2.61600E-012 | .429 | -7.4568E-012 | 3.2605E-012 |
| | | PFHDay1 | 7.65778E-013 | 2.77469E-012 | .785 | -4.9179E-012 | 6.4495E-012 |
| | | BMHDay1 | 2.35666E-013 | 2.77469E-012 | .933 | -5.4480E-012 | 5.9194E-012 |
| | | PMHDay1 | -7.50294E-013 | 2.77469E-012 | .789 | -6.4340E-012 | 4.9334E-012 |
| | | BFHDay2 | 3.59796E-013 | 2.61600E-012 | .892 | -4.9988E-012 | 5.7184E-012 |
| | | PFHDay2 | 5.48285E-014 | 2.77469E-012 | .984 | -5.6289E-012 | 5.7385E-012 |
| | | PMHDay2 | 1.20881E-012 | 2.61600E-012 | .648 | -4.1498E-012 | 6.5674E-012 |
| | PMHDay2 | BFHDay1 | -3.30698E-012 | 2.61600E-012 | .217 | -8.6656E-012 | 2.0517E-012 |
| | | PFHDay1 | -4.43034E-013 | 2.77469E-012 | .874 | -6.1267E-012 | 5.2407E-012 |
| | | BMHDay1 | -9.73146E-013 | 2.77469E-012 | .728 | -6.6568E-012 | 4.7105E-012 |
| | | PMHDay1 | -1.95911E-012 | 2.77469E-012 | .486 | -7.6428E-012 | 3.7246E-012 |
| | | BFHDay2 | -8.49016E-013 | 2.61600E-012 | .748 | -6.2077E-012 | 4.5096E-012 |
| | | PFHDay2 | -1.15398E-012 | 2.77469E-012 | .681 | -6.8377E-012 | 4.5297E-012 |
| | | BMHDay2 | -1.20881E-012 | 2.61600E-012 | .648 | -6.5674E-012 | 4.1498E-012 |
| oleTrypt | BFHDay1 | PFHDay1 | 1.66806E-012 | 2.70036E-012 | .545 | -4.0292E-012 | 7.3653E-012 |
| | | BMHDay1 | -2.27367E-013 | 2.38149E-012 | .925 | -5.2519E-012 | 4.7971E-012 |
| | | PMHDay1 | -2.79937E-012 | 2.09169E-012 | .198 | -7.2124E-012 | 1.6137E-012 |
| | | BFHDay2 | -1.25626E-012 | 2.38149E-012 | .605 | -6.2808E-012 | 3.7682E-012 |

| | | | | | | |
|---|---|---|---|---|---|---|
| | PFHDay2 | 1.17979E-012 | 2.38149E-012 | .627 | -3.8447E-012 | 6.2043E-012 |
| | BMHDay2 | -6.02285E-012* | 2.70036E-012 | .039 | -1.1720E-011 | -3.2559E-013 |
| | PMHDay2 | 1.75448E-012 | 2.38149E-012 | .471 | -3.2700E-012 | 6.7790E-012 |
| PFHDay1 | BFHDay1 | -1.66806E-012 | 2.70036E-012 | .545 | -7.3653E-012 | 4.0292E-012 |
| | BMHDay1 | -1.89543E-012 | 2.84643E-012 | .514 | -7.9009E-012 | 4.1100E-012 |
| | PMHDay1 | -4.46743E-012 | 2.60879E-012 | .105 | -9.9715E-012 | 1.0366E-012 |
| | BFHDay2 | -2.92433E-012 | 2.84643E-012 | .319 | -8.9298E-012 | 3.0811E-012 |
| | PFHDay2 | -4.88270E-013 | 2.84643E-012 | .866 | -6.4937E-012 | 5.5172E-012 |
| | BMHDay2 | -7.69091E-012* | 3.11810E-012 | .025 | -1.4270E-011 | -1.1123E-012 |
| | PMHDay2 | 8.64133E-014 | 2.84643E-012 | .976 | -5.9190E-012 | 6.0919E-012 |
| BMHDay1 | BFHDay1 | 2.27367E-013 | 2.38149E-012 | .925 | -4.7971E-012 | 5.2519E-012 |
| | PFHDay1 | 1.89543E-012 | 2.84643E-012 | .514 | -4.1100E-012 | 7.9009E-012 |
| | PMHDay1 | -2.57200E-012 | 2.27714E-012 | .274 | -7.3763E-012 | 2.2323E-012 |
| | BFHDay2 | -1.02890E-012 | 2.54592E-012 | .691 | -6.4003E-012 | 4.3425E-012 |
| | PFHDay2 | 1.40716E-012 | 2.54592E-012 | .588 | -3.9643E-012 | 6.7786E-012 |
| | BMHDay2 | -5.79548E-012 | 2.84643E-012 | .058 | -1.1801E-011 | 2.0996E-013 |
| | PMHDay2 | 1.98184E-012 | 2.54592E-012 | .447 | -3.3896E-012 | 7.3533E-012 |
| PMHDay1 | BFHDay1 | 2.79937E-012 | 2.09169E-012 | .198 | -1.6137E-012 | 7.2124E-012 |
| | PFHDay1 | 4.46743E-012 | 2.60879E-012 | .105 | -1.0366E-012 | 9.9715E-012 |

| | | | | | | |
|---|---|---|---|---|---|---|
| | BMHDay1 | 2.57200E-012 | 2.27714E-012 | .274 | -2.2323E-012 | 7.3763E-012 |
| | BFHDay2 | 1.54310E-012 | 2.27714E-012 | .507 | -3.2612E-012 | 6.3475E-012 |
| | PFHDay2 | 3.97916E-012 | 2.27714E-012 | .099 | -8.2519E-013 | 8.7835E-012 |
| | BMHDay2 | -3.22348E-012 | 2.60879E-012 | .233 | -8.7276E-012 | 2.2806E-012 |
| | PMHDay2 | 4.55384E-012 | 2.27714E-012 | .062 | -2.5051E-013 | 9.3582E-012 |
| BFHDay2 | BFHDay1 | 1.25626E-012 | 2.38149E-012 | .605 | -3.7682E-012 | 6.2808E-012 |
| | PFHDay1 | 2.92433E-012 | 2.84643E-012 | .319 | -3.0811E-012 | 8.9298E-012 |
| | BMHDay1 | 1.02890E-012 | 2.54592E-012 | .691 | -4.3425E-012 | 6.4003E-012 |
| | PMHDay1 | -1.54310E-012 | 2.27714E-012 | .507 | -6.3475E-012 | 3.2612E-012 |
| | PFHDay2 | 2.43606E-012 | 2.54592E-012 | .352 | -2.9354E-012 | 7.8075E-012 |
| | BMHDay2 | -4.76658E-012 | 2.84643E-012 | .112 | -1.0772E-011 | 1.2389E-012 |
| | PMHDay2 | 3.01074E-012 | 2.54592E-012 | .253 | -2.3607E-012 | 8.3822E-012 |
| PFHDay2 | BFHDay1 | -1.17979E-012 | 2.38149E-012 | .627 | -6.2043E-012 | 3.8447E-012 |
| | PFHDay1 | 4.88270E-013 | 2.84643E-012 | .866 | -5.5172E-012 | 6.4937E-012 |
| | BMHDay1 | -1.40716E-012 | 2.54592E-012 | .588 | -6.7786E-012 | 3.9643E-012 |
| | PMHDay1 | -3.97916E-012 | 2.27714E-012 | .099 | -8.7835E-012 | 8.2519E-013 |
| | BFHDay2 | -2.43606E-012 | 2.54592E-012 | .352 | -7.8075E-012 | 2.9354E-012 |
| | BMHDay2 | -7.20264E-012* | 2.84643E-012 | .022 | -1.3208E-011 | -1.1972E-012 |
| | PMHDay2 | 5.74683E-013 | 2.54592E-012 | .824 | -4.7967E-012 | 5.9461E-012 |

| | | | | | | | |
|---|---|---|---|---|---|---|---|
| | BMHDay2 | BFHDay1 | 6.02285E-012* | 2.70036E-012 | .039 | 3.2559E-013 | 1.1720E-011 |
| | | PFHDay1 | 7.69091E-012* | 3.11810E-012 | .025 | 1.1123E-012 | 1.4270E-011 |
| | | BMHDay1 | 5.79548E-012 | 2.84643E-012 | .058 | -2.0996E-013 | 1.1801E-011 |
| | | PMHDay1 | 3.22348E-012 | 2.60879E-012 | .233 | -2.2806E-012 | 8.7276E-012 |
| | | BFHDay2 | 4.76658E-012 | 2.84643E-012 | .112 | -1.2389E-012 | 1.0772E-011 |
| | | PFHDay2 | 7.20264E-012* | 2.84643E-012 | .022 | 1.1972E-012 | 1.3208E-011 |
| | | PMHDay2 | 7.77732E-012* | 2.84643E-012 | .014 | 1.7719E-012 | 1.3783E-011 |
| | PMHDay2 | BFHDay1 | -1.75448E-012 | 2.38149E-012 | .471 | -6.7790E-012 | 3.2700E-012 |
| | | PFHDay1 | -8.64133E-014 | 2.84643E-012 | .976 | -6.0919E-012 | 5.9190E-012 |
| | | BMHDay1 | -1.98184E-012 | 2.54592E-012 | .447 | -7.3533E-012 | 3.3896E-012 |
| | | PMHDay1 | -4.55384E-012 | 2.27714E-012 | .062 | -9.3582E-012 | 2.5051E-013 |
| | | BFHDay2 | -3.01074E-012 | 2.54592E-012 | .253 | -8.3822E-012 | 2.3607E-012 |
| | | PFHDay2 | -5.74683E-013 | 2.54592E-012 | .824 | -5.9461E-012 | 4.7967E-012 |
| | | BMHDay2 | -7.77732E-012* | 2.84643E-012 | .014 | -1.3783E-011 | -1.7719E-012 |
| palTyr | BFHDay1 | PFHDay1 | 5.14298E-012 | 4.20896E-012 | .231 | -3.4304E-012 | 1.3716E-011 |
| | | BMHDay1 | 3.36885E-012 | 4.20896E-012 | .429 | -5.2045E-012 | 1.1942E-011 |
| | | PMHDay1 | -3.14395E-012 | 4.20896E-012 | .461 | -1.1717E-011 | 5.4294E-012 |
| | | BFHDay2 | 3.37267E-012 | 4.20896E-012 | .429 | -5.2007E-012 | 1.1946E-011 |
| | | PFHDay2 | 1.48825E-012 | 4.20896E-012 | .726 | -7.0851E-012 | 1.0062E-011 |

| | | | | | | | |
|---|---|---|---|---|---|---|---|
| | | BMHDay2 | -2.15440E-012 | 4.20896E-012 | .612 | -1.0728E-011 | 6.4190E-012 |
| | | PMHDay2 | -6.49028E-012 | 4.20896E-012 | .133 | -1.5064E-011 | 2.0831E-012 |
| | PFHDay1 | BFHDay1 | -5.14298E-012 | 4.20896E-012 | .231 | -1.3716E-011 | 3.4304E-012 |
| | | BMHDay1 | -1.77413E-012 | 4.20896E-012 | .676 | -1.0348E-011 | 6.7992E-012 |
| | | PMHDay1 | -8.28693E-012 | 4.20896E-012 | .058 | -1.6860E-011 | 2.8644E-013 |
| | | BFHDay2 | -1.77032E-012 | 4.20896E-012 | .677 | -1.0344E-011 | 6.8031E-012 |
| | | PFHDay2 | -3.65474E-012 | 4.20896E-012 | .392 | -1.2228E-011 | 4.9186E-012 |
| | | BMHDay2 | -7.29738E-012 | 4.20896E-012 | .093 | -1.5871E-011 | 1.2760E-012 |
| | | PMHDay2 | -1.16333E-011* | 4.20896E-012 | .009 | -2.0207E-011 | -3.0599E-012 |
| | BMHDay1 | BFHDay1 | -3.36885E-012 | 4.20896E-012 | .429 | -1.1942E-011 | 5.2045E-012 |
| | | PFHDay1 | 1.77413E-012 | 4.20896E-012 | .676 | -6.7992E-012 | 1.0348E-011 |
| | | PMHDay1 | -6.51280E-012 | 4.20896E-012 | .132 | -1.5086E-011 | 2.0606E-012 |
| | | BFHDay2 | 3.81600E-015 | 4.20896E-012 | .999 | -8.5696E-012 | 8.5772E-012 |
| | | PFHDay2 | -1.88060E-012 | 4.20896E-012 | .658 | -1.0454E-011 | 6.6928E-012 |
| | | BMHDay2 | -5.52325E-012 | 4.20896E-012 | .199 | -1.4097E-011 | 3.0501E-012 |
| | | PMHDay2 | -9.85913E-012* | 4.20896E-012 | .026 | -1.8433E-011 | -1.2858E-012 |
| | PMHDay1 | BFHDay1 | 3.14395E-012 | 4.20896E-012 | .461 | -5.4294E-012 | 1.1717E-011 |
| | | PFHDay1 | 8.28693E-012 | 4.20896E-012 | .058 | -2.8644E-013 | 1.6860E-011 |
| | | BMHDay1 | 6.51280E-012 | 4.20896E-012 | .132 | -2.0606E-012 | 1.5086E-011 |

| | | | | | | |
|---|---|---|---|---|---|---|
| | BFHDay2 | 6.51661E-012 | 4.20896E-012 | .131 | -2.0568E-012 | 1.5090E-011 |
| | PFHDay2 | 4.63219E-012 | 4.20896E-012 | .279 | -3.9412E-012 | 1.3206E-011 |
| | BMHDay2 | 9.89548E-013 | 4.20896E-012 | .816 | -7.5838E-012 | 9.5629E-012 |
| | PMHDay2 | -3.34633E-012 | 4.20896E-012 | .432 | -1.1920E-011 | 5.2270E-012 |
| BFHDay2 | BFHDay1 | -3.37267E-012 | 4.20896E-012 | .429 | -1.1946E-011 | 5.2007E-012 |
| | PFHDay1 | 1.77032E-012 | 4.20896E-012 | .677 | -6.8031E-012 | 1.0344E-011 |
| | BMHDay1 | -3.81600E-015 | 4.20896E-012 | .999 | -8.5772E-012 | 8.5696E-012 |
| | PMHDay1 | -6.51661E-012 | 4.20896E-012 | .131 | -1.5090E-011 | 2.0568E-012 |
| | PFHDay2 | -1.88442E-012 | 4.20896E-012 | .657 | -1.0458E-011 | 6.6889E-012 |
| | BMHDay2 | -5.52707E-012 | 4.20896E-012 | .198 | -1.4100E-011 | 3.0463E-012 |
| | PMHDay2 | -9.86295E-012* | 4.20896E-012 | .025 | -1.8436E-011 | -1.2896E-012 |
| PFHDay2 | BFHDay1 | -1.48825E-012 | 4.20896E-012 | .726 | -1.0062E-011 | 7.0851E-012 |
| | PFHDay1 | 3.65474E-012 | 4.20896E-012 | .392 | -4.9186E-012 | 1.2228E-011 |
| | BMHDay1 | 1.88060E-012 | 4.20896E-012 | .658 | -6.6928E-012 | 1.0454E-011 |
| | PMHDay1 | -4.63219E-012 | 4.20896E-012 | .279 | -1.3206E-011 | 3.9412E-012 |
| | BFHDay2 | 1.88442E-012 | 4.20896E-012 | .657 | -6.6889E-012 | 1.0458E-011 |
| | BMHDay2 | -3.64265E-012 | 4.20896E-012 | .393 | -1.2216E-011 | 4.9307E-012 |
| | PMHDay2 | -7.97853E-012 | 4.20896E-012 | .067 | -1.6552E-011 | 5.9484E-013 |
| BMHDay2 | BFHDay1 | 2.15440E-012 | 4.20896E-012 | .612 | -6.4190E-012 | 1.0728E-011 |

| | | | | | | | |
|---|---|---|---|---|---|---|---|
| | | PFHDay1 | 7.29738E-012 | 4.20896E-012 | .093 | -1.2760E-012 | 1.5871E-011 |
| | | BMHDay1 | 5.52325E-012 | 4.20896E-012 | .199 | -3.0501E-012 | 1.4097E-011 |
| | | PMHDay1 | -9.89548E-013 | 4.20896E-012 | .816 | -9.5629E-012 | 7.5838E-012 |
| | | BFHDay2 | 5.52707E-012 | 4.20896E-012 | .198 | -3.0463E-012 | 1.4100E-011 |
| | | PFHDay2 | 3.64265E-012 | 4.20896E-012 | .393 | -4.9307E-012 | 1.2216E-011 |
| | | PMHDay2 | -4.33588E-012 | 4.20896E-012 | .311 | -1.2909E-011 | 4.2375E-012 |
| | PMHDay2 | BFHDay1 | 6.49028E-012 | 4.20896E-012 | .133 | -2.0831E-012 | 1.5064E-011 |
| | | PFHDay1 | 1.16333E-011* | 4.20896E-012 | .009 | 3.0599E-012 | 2.0207E-011 |
| | | BMHDay1 | 9.85913E-012* | 4.20896E-012 | .026 | 1.2858E-012 | 1.8433E-011 |
| | | PMHDay1 | 3.34633E-012 | 4.20896E-012 | .432 | -5.2270E-012 | 1.1920E-011 |
| | | BFHDay2 | 9.86295E-012* | 4.20896E-012 | .025 | 1.2896E-012 | 1.8436E-011 |
| | | PFHDay2 | 7.97853E-012 | 4.20896E-012 | .067 | -5.9484E-013 | 1.6552E-011 |
| | | BMHDay2 | 4.33588E-012 | 4.20896E-012 | .311 | -4.2375E-012 | 1.2909E-011 |
| steTyr | BFHDay1 | PFHDay1 | 1.30486E-012 | 1.53798E-012 | .403 | -1.8279E-012 | 4.4376E-012 |
| | | BMHDay1 | -1.30861E-013 | 1.53798E-012 | .933 | -3.2636E-012 | 3.0019E-012 |
| | | PMHDay1 | -8.41945E-013 | 1.53798E-012 | .588 | -3.9747E-012 | 2.2908E-012 |
| | | BFHDay2 | 5.94081E-013 | 1.53798E-012 | .702 | -2.5387E-012 | 3.7269E-012 |
| | | PFHDay2 | 1.30468E-014 | 1.53798E-012 | .993 | -3.1197E-012 | 3.1458E-012 |
| | | BMHDay2 | -1.63022E-012 | 1.53798E-012 | .297 | -4.7630E-012 | 1.5025E-012 |

| | | | | | | |
|---|---|---|---|---|---|---|
| | PMHDay2 | -2.09563E-012 | 1.53798E-012 | .183 | -5.2284E-012 | 1.0371E-012 |
| PFHDay1 | BFHDay1 | -1.30486E-012 | 1.53798E-012 | .403 | -4.4376E-012 | 1.8279E-012 |
| | BMHDay1 | -1.43572E-012 | 1.53798E-012 | .358 | -4.5685E-012 | 1.6971E-012 |
| | PMHDay1 | -2.14680E-012 | 1.53798E-012 | .172 | -5.2796E-012 | 9.8597E-013 |
| | BFHDay2 | -7.10778E-013 | 1.53798E-012 | .647 | -3.8435E-012 | 2.4220E-012 |
| | PFHDay2 | -1.29181E-012 | 1.53798E-012 | .407 | -4.4246E-012 | 1.8410E-012 |
| | BMHDay2 | -2.93508E-012 | 1.53798E-012 | .065 | -6.0679E-012 | 1.9769E-013 |
| | PMHDay2 | -3.40049E-012* | 1.53798E-012 | .034 | -6.5333E-012 | -2.6772E-013 |
| BMHDay1 | BFHDay1 | 1.30861E-013 | 1.53798E-012 | .933 | -3.0019E-012 | 3.2636E-012 |
| | PFHDay1 | 1.43572E-012 | 1.53798E-012 | .358 | -1.6971E-012 | 4.5685E-012 |
| | PMHDay1 | -7.11084E-013 | 1.53798E-012 | .647 | -3.8439E-012 | 2.4217E-012 |
| | BFHDay2 | 7.24942E-013 | 1.53798E-012 | .641 | -2.4078E-012 | 3.8577E-012 |
| | PFHDay2 | 1.43908E-013 | 1.53798E-012 | .926 | -2.9889E-012 | 3.2767E-012 |
| | BMHDay2 | -1.49936E-012 | 1.53798E-012 | .337 | -4.6321E-012 | 1.6334E-012 |
| | PMHDay2 | -1.96477E-012 | 1.53798E-012 | .211 | -5.0975E-012 | 1.1680E-012 |
| PMHDay1 | BFHDay1 | 8.41945E-013 | 1.53798E-012 | .588 | -2.2908E-012 | 3.9747E-012 |
| | PFHDay1 | 2.14680E-012 | 1.53798E-012 | .172 | -9.8597E-013 | 5.2796E-012 |
| | BMHDay1 | 7.11084E-013 | 1.53798E-012 | .647 | -2.4217E-012 | 3.8439E-012 |
| | BFHDay2 | 1.43603E-012 | 1.53798E-012 | .357 | -1.6967E-012 | 4.5688E-012 |

| | | | | | | |
|---|---|---|---|---|---|---|
| | PFHDay2 | 8.54992E-013 | 1.53798E-012 | .582 | -2.2778E-012 | 3.9878E-012 |
| | BMHDay2 | -7.88280E-013 | 1.53798E-012 | .612 | -3.9211E-012 | 2.3445E-012 |
| | PMHDay2 | -1.25369E-012 | 1.53798E-012 | .421 | -4.3865E-012 | 1.8791E-012 |
| BFHDay2 | BFHDay1 | -5.94081E-013 | 1.53798E-012 | .702 | -3.7269E-012 | 2.5387E-012 |
| | PFHDay1 | 7.10778E-013 | 1.53798E-012 | .647 | -2.4220E-012 | 3.8435E-012 |
| | BMHDay1 | -7.24942E-013 | 1.53798E-012 | .641 | -3.8577E-012 | 2.4078E-012 |
| | PMHDay1 | -1.43603E-012 | 1.53798E-012 | .357 | -4.5688E-012 | 1.6967E-012 |
| | PFHDay2 | -5.81034E-013 | 1.53798E-012 | .708 | -3.7138E-012 | 2.5517E-012 |
| | BMHDay2 | -2.22431E-012 | 1.53798E-012 | .158 | -5.3571E-012 | 9.0847E-013 |
| | PMHDay2 | -2.68971E-012 | 1.53798E-012 | .090 | -5.8225E-012 | 4.4306E-013 |
| PFHDay2 | BFHDay1 | -1.30468E-014 | 1.53798E-012 | .993 | -3.1458E-012 | 3.1197E-012 |
| | PFHDay1 | 1.29181E-012 | 1.53798E-012 | .407 | -1.8410E-012 | 4.4246E-012 |
| | BMHDay1 | -1.43908E-013 | 1.53798E-012 | .926 | -3.2767E-012 | 2.9889E-012 |
| | PMHDay1 | -8.54992E-013 | 1.53798E-012 | .582 | -3.9878E-012 | 2.2778E-012 |
| | BFHDay2 | 5.81034E-013 | 1.53798E-012 | .708 | -2.5517E-012 | 3.7138E-012 |
| | BMHDay2 | -1.64327E-012 | 1.53798E-012 | .293 | -4.7760E-012 | 1.4895E-012 |
| | PMHDay2 | -2.10868E-012 | 1.53798E-012 | .180 | -5.2415E-012 | 1.0241E-012 |
| BMHDay2 | BFHDay1 | 1.63022E-012 | 1.53798E-012 | .297 | -1.5025E-012 | 4.7630E-012 |
| | PFHDay1 | 2.93508E-012 | 1.53798E-012 | .065 | -1.9769E-013 | 6.0679E-012 |

| | | | | | | | |
|---|---|---|---|---|---|---|---|
| | | BMHDay1 | 1.49936E-012 | 1.53798E-012 | .337 | -1.6334E-012 | 4.6321E-012 |
| | | PMHDay1 | 7.88280E-013 | 1.53798E-012 | .612 | -2.3445E-012 | 3.9211E-012 |
| | | BFHDay2 | 2.22431E-012 | 1.53798E-012 | .158 | -9.0847E-013 | 5.3571E-012 |
| | | PFHDay2 | 1.64327E-012 | 1.53798E-012 | .293 | -1.4895E-012 | 4.7760E-012 |
| | | PMHDay2 | -4.65408E-013 | 1.53798E-012 | .764 | -3.5982E-012 | 2.6674E-012 |
| | PMHDay2 | BFHDay1 | 2.09563E-012 | 1.53798E-012 | .183 | -1.0371E-012 | 5.2284E-012 |
| | | PFHDay1 | 3.40049E-012* | 1.53798E-012 | .034 | 2.6772E-013 | 6.5333E-012 |
| | | BMHDay1 | 1.96477E-012 | 1.53798E-012 | .211 | -1.1680E-012 | 5.0975E-012 |
| | | PMHDay1 | 1.25369E-012 | 1.53798E-012 | .421 | -1.8791E-012 | 4.3865E-012 |
| | | BFHDay2 | 2.68971E-012 | 1.53798E-012 | .090 | -4.4306E-013 | 5.8225E-012 |
| | | PFHDay2 | 2.10868E-012 | 1.53798E-012 | .180 | -1.0241E-012 | 5.2415E-012 |
| | | BMHDay2 | 4.65408E-013 | 1.53798E-012 | .764 | -2.6674E-012 | 3.5982E-012 |
| oleTyr | BFHDay1 | PFHDay1 | 6.07408E-012 | 7.03852E-012 | .395 | -8.2629E-012 | 2.0411E-011 |
| | | BMHDay1 | 4.62970E-012 | 7.03852E-012 | .515 | -9.7073E-012 | 1.8967E-011 |
| | | PMHDay1 | -2.30978E-012 | 7.03852E-012 | .745 | -1.6647E-011 | 1.2027E-011 |
| | | BFHDay2 | 2.71857E-012 | 7.03852E-012 | .702 | -1.1618E-011 | 1.7056E-011 |
| | | PFHDay2 | -5.08130E-013 | 7.03852E-012 | .943 | -1.4845E-011 | 1.3829E-011 |
| | | BMHDay2 | -8.23176E-012 | 7.03852E-012 | .251 | -2.2569E-011 | 6.1052E-012 |
| | | PMHDay2 | -1.91172E-011* | 7.03852E-012 | .011 | -3.3454E-011 | -4.7802E-012 |

| | | | | | | |
|---|---|---|---|---|---|---|
| | PFHDay1 | BFHDay1 | -6.07408E-012 | 7.03852E-012 | .395 | -2.0411E-011 | 8.2629E-012 |
| | | BMHDay1 | -1.44438E-012 | 7.03852E-012 | .839 | -1.5781E-011 | 1.2893E-011 |
| | | PMHDay1 | -8.38386E-012 | 7.03852E-012 | .242 | -2.2721E-011 | 5.9531E-012 |
| | | BFHDay2 | -3.35551E-012 | 7.03852E-012 | .637 | -1.7692E-011 | 1.0981E-011 |
| | | PFHDay2 | -6.58221E-012 | 7.03852E-012 | .357 | -2.0919E-011 | 7.7548E-012 |
| | | BMHDay2 | -1.43058E-011 | 7.03852E-012 | .050 | -2.8643E-011 | 3.1147E-014 |
| | | PMHDay2 | -2.51912E-011* | 7.03852E-012 | .001 | -3.9528E-011 | -1.0854E-011 |
| | BMHDay1 | BFHDay1 | -4.62970E-012 | 7.03852E-012 | .515 | -1.8967E-011 | 9.7073E-012 |
| | | PFHDay1 | 1.44438E-012 | 7.03852E-012 | .839 | -1.2893E-011 | 1.5781E-011 |
| | | PMHDay1 | -6.93947E-012 | 7.03852E-012 | .332 | -2.1276E-011 | 7.3975E-012 |
| | | BFHDay2 | -1.91112E-012 | 7.03852E-012 | .788 | -1.6248E-011 | 1.2426E-011 |
| | | PFHDay2 | -5.13783E-012 | 7.03852E-012 | .471 | -1.9475E-011 | 9.1992E-012 |
| | | BMHDay2 | -1.28615E-011 | 7.03852E-012 | .077 | -2.7198E-011 | 1.4755E-012 |
| | | PMHDay2 | -2.37469E-011* | 7.03852E-012 | .002 | -3.8084E-011 | -9.4099E-012 |
| | PMHDay1 | BFHDay1 | 2.30978E-012 | 7.03852E-012 | .745 | -1.2027E-011 | 1.6647E-011 |
| | | PFHDay1 | 8.38386E-012 | 7.03852E-012 | .242 | -5.9531E-012 | 2.2721E-011 |
| | | BMHDay1 | 6.93947E-012 | 7.03852E-012 | .332 | -7.3975E-012 | 2.1276E-011 |
| | | BFHDay2 | 5.02835E-012 | 7.03852E-012 | .480 | -9.3086E-012 | 1.9365E-011 |
| | | PFHDay2 | 1.80165E-012 | 7.03852E-012 | .800 | -1.2535E-011 | 1.6139E-011 |

| | | | | | | |
|---|---|---|---|---|---|---|
| | BMHDay2 | -5.92199E-012 | 7.03852E-012 | .406 | -2.0259E-011 | 8.4150E-012 |
| | PMHDay2 | -1.68074E-011* | 7.03852E-012 | .023 | -3.1144E-011 | -2.4704E-012 |
| BFHDay2 | BFHDay1 | -2.71857E-012 | 7.03852E-012 | .702 | -1.7056E-011 | 1.1618E-011 |
| | PFHDay1 | 3.35551E-012 | 7.03852E-012 | .637 | -1.0981E-011 | 1.7692E-011 |
| | BMHDay1 | 1.91112E-012 | 7.03852E-012 | .788 | -1.2426E-011 | 1.6248E-011 |
| | PMHDay1 | -5.02835E-012 | 7.03852E-012 | .480 | -1.9365E-011 | 9.3086E-012 |
| | PFHDay2 | -3.22670E-012 | 7.03852E-012 | .650 | -1.7564E-011 | 1.1110E-011 |
| | BMHDay2 | -1.09503E-011 | 7.03852E-012 | .130 | -2.5287E-011 | 3.3867E-012 |
| | PMHDay2 | -2.18357E-011* | 7.03852E-012 | .004 | -3.6173E-011 | -7.4988E-012 |
| PFHDay2 | BFHDay1 | 5.08130E-013 | 7.03852E-012 | .943 | -1.3829E-011 | 1.4845E-011 |
| | PFHDay1 | 6.58221E-012 | 7.03852E-012 | .357 | -7.7548E-012 | 2.0919E-011 |
| | BMHDay1 | 5.13783E-012 | 7.03852E-012 | .471 | -9.1992E-012 | 1.9475E-011 |
| | PMHDay1 | -1.80165E-012 | 7.03852E-012 | .800 | -1.6139E-011 | 1.2535E-011 |
| | BFHDay2 | 3.22670E-012 | 7.03852E-012 | .650 | -1.1110E-011 | 1.7564E-011 |
| | BMHDay2 | -7.72363E-012 | 7.03852E-012 | .281 | -2.2061E-011 | 6.6134E-012 |
| | PMHDay2 | -1.86090E-011* | 7.03852E-012 | .013 | -3.2946E-011 | -4.2720E-012 |
| BMHDay2 | BFHDay1 | 8.23176E-012 | 7.03852E-012 | .251 | -6.1052E-012 | 2.2569E-011 |
| | PFHDay1 | 1.43058E-011 | 7.03852E-012 | .050 | -3.1147E-014 | 2.8643E-011 |
| | BMHDay1 | 1.28615E-011 | 7.03852E-012 | .077 | -1.4755E-012 | 2.7198E-011 |

| | | | | | | | |
|---|---|---|---|---|---|---|---|
| | | PMHDay1 | 5.92199E-012 | 7.03852E-012 | .406 | -8.4150E-012 | 2.0259E-011 |
| | | BFHDay2 | 1.09503E-011 | 7.03852E-012 | .130 | -3.3867E-012 | 2.5287E-011 |
| | | PFHDay2 | 7.72363E-012 | 7.03852E-012 | .281 | -6.6134E-012 | 2.2061E-011 |
| | | PMHDay2 | -1.08854E-011 | 7.03852E-012 | .132 | -2.5222E-011 | 3.4516E-012 |
| | PMHDay2 | BFHDay1 | 1.91172E-011* | 7.03852E-012 | .011 | 4.7802E-012 | 3.3454E-011 |
| | | PFHDay1 | 2.51912E-011* | 7.03852E-012 | .001 | 1.0854E-011 | 3.9528E-011 |
| | | BMHDay1 | 2.37469E-011* | 7.03852E-012 | .002 | 9.4099E-012 | 3.8084E-011 |
| | | PMHDay1 | 1.68074E-011* | 7.03852E-012 | .023 | 2.4704E-012 | 3.1144E-011 |
| | | BFHDay2 | 2.18357E-011* | 7.03852E-012 | .004 | 7.4988E-012 | 3.6173E-011 |
| | | PFHDay2 | 1.86090E-011* | 7.03852E-012 | .013 | 4.2720E-012 | 3.2946E-011 |
| | | BMHDay2 | 1.08854E-011 | 7.03852E-012 | .132 | -3.4516E-012 | 2.5222E-011 |
| linTyr | BFHDay1 | PFHDay1 | 7.14354E-012 | 7.33274E-012 | .337 | -7.7928E-012 | 2.2080E-011 |
| | | BMHDay1 | 1.03807E-012 | 7.33274E-012 | .888 | -1.3898E-011 | 1.5974E-011 |
| | | PMHDay1 | -4.68397E-012 | 7.33274E-012 | .528 | -1.9620E-011 | 1.0252E-011 |
| | | BFHDay2 | 5.67680E-013 | 7.33274E-012 | .939 | -1.4369E-011 | 1.5504E-011 |
| | | PFHDay2 | 3.39378E-012 | 7.33274E-012 | .647 | -1.1543E-011 | 1.8330E-011 |
| | | BMHDay2 | -6.04289E-012 | 7.33274E-012 | .416 | -2.0979E-011 | 8.8934E-012 |
| | | PMHDay2 | -1.66764E-011* | 7.33274E-012 | .030 | -3.1613E-011 | -1.7401E-012 |
| | PFHDay1 | BFHDay1 | -7.14354E-012 | 7.33274E-012 | .337 | -2.2080E-011 | 7.7928E-012 |

| | | | | | | |
|---|---|---|---|---|---|---|
| | BMHDay1 | -6.10546E-012 | 7.33274E-012 | .411 | -2.1042E-011 | 8.8308E-012 |
| | PMHDay1 | -1.18275E-011 | 7.33274E-012 | .117 | -2.6764E-011 | 3.1088E-012 |
| | BFHDay2 | -6.57586E-012 | 7.33274E-012 | .377 | -2.1512E-011 | 8.3604E-012 |
| | PFHDay2 | -3.74975E-012 | 7.33274E-012 | .613 | -1.8686E-011 | 1.1187E-011 |
| | BMHDay2 | -1.31864E-011 | 7.33274E-012 | .082 | -2.8123E-011 | 1.7499E-012 |
| | PMHDay2 | -2.38199E-011* | 7.33274E-012 | .003 | -3.8756E-011 | -8.8836E-012 |
| BMHDay1 | BFHDay1 | -1.03807E-012 | 7.33274E-012 | .888 | -1.5974E-011 | 1.3898E-011 |
| | PFHDay1 | 6.10546E-012 | 7.33274E-012 | .411 | -8.8308E-012 | 2.1042E-011 |
| | PMHDay1 | -5.72205E-012 | 7.33274E-012 | .441 | -2.0658E-011 | 9.2143E-012 |
| | BFHDay2 | -4.70394E-013 | 7.33274E-012 | .949 | -1.5407E-011 | 1.4466E-011 |
| | PFHDay2 | 2.35571E-012 | 7.33274E-012 | .750 | -1.2581E-011 | 1.7292E-011 |
| | BMHDay2 | -7.08096E-012 | 7.33274E-012 | .341 | -2.2017E-011 | 7.8553E-012 |
| | PMHDay2 | -1.77144E-011* | 7.33274E-012 | .022 | -3.2651E-011 | -2.7781E-012 |
| PMHDay1 | BFHDay1 | 4.68397E-012 | 7.33274E-012 | .528 | -1.0252E-011 | 1.9620E-011 |
| | PFHDay1 | 1.18275E-011 | 7.33274E-012 | .117 | -3.1088E-012 | 2.6764E-011 |
| | BMHDay1 | 5.72205E-012 | 7.33274E-012 | .441 | -9.2143E-012 | 2.0658E-011 |
| | BFHDay2 | 5.25165E-012 | 7.33274E-012 | .479 | -9.6847E-012 | 2.0188E-011 |
| | PFHDay2 | 8.07775E-012 | 7.33274E-012 | .279 | -6.8585E-012 | 2.3014E-011 |
| | BMHDay2 | -1.35891E-012 | 7.33274E-012 | .854 | -1.6295E-011 | 1.3577E-011 |

| | | | | | | |
|---|---|---|---|---|---|---|
| | PMHDay2 | -1.19924E-011 | 7.33274E-012 | .112 | -2.6929E-011 | 2.9439E-012 |
| BFHDay2 | BFHDay1 | -5.67680E-013 | 7.33274E-012 | .939 | -1.5504E-011 | 1.4369E-011 |
| | PFHDay1 | 6.57586E-012 | 7.33274E-012 | .377 | -8.3604E-012 | 2.1512E-011 |
| | BMHDay1 | 4.70394E-013 | 7.33274E-012 | .949 | -1.4466E-011 | 1.5407E-011 |
| | PMHDay1 | -5.25165E-012 | 7.33274E-012 | .479 | -2.0188E-011 | 9.6847E-012 |
| | PFHDay2 | 2.82610E-012 | 7.33274E-012 | .702 | -1.2110E-011 | 1.7762E-011 |
| | BMHDay2 | -6.61057E-012 | 7.33274E-012 | .374 | -2.1547E-011 | 8.3257E-012 |
| | PMHDay2 | -1.72440E-011* | 7.33274E-012 | .025 | -3.2180E-011 | -2.3077E-012 |
| PFHDay2 | BFHDay1 | -3.39378E-012 | 7.33274E-012 | .647 | -1.8330E-011 | 1.1543E-011 |
| | PFHDay1 | 3.74975E-012 | 7.33274E-012 | .613 | -1.1187E-011 | 1.8686E-011 |
| | BMHDay1 | -2.35571E-012 | 7.33274E-012 | .750 | -1.7292E-011 | 1.2581E-011 |
| | PMHDay1 | -8.07775E-012 | 7.33274E-012 | .279 | -2.3014E-011 | 6.8585E-012 |
| | BFHDay2 | -2.82610E-012 | 7.33274E-012 | .702 | -1.7762E-011 | 1.2110E-011 |
| | BMHDay2 | -9.43667E-012 | 7.33274E-012 | .207 | -2.4373E-011 | 5.4996E-012 |
| | PMHDay2 | -2.00701E-011* | 7.33274E-012 | .010 | -3.5006E-011 | -5.1338E-012 |
| BMHDay2 | BFHDay1 | 6.04289E-012 | 7.33274E-012 | .416 | -8.8934E-012 | 2.0979E-011 |
| | PFHDay1 | 1.31864E-011 | 7.33274E-012 | .082 | -1.7499E-012 | 2.8123E-011 |
| | BMHDay1 | 7.08096E-012 | 7.33274E-012 | .341 | -7.8553E-012 | 2.2017E-011 |
| | PMHDay1 | 1.35891E-012 | 7.33274E-012 | .854 | -1.3577E-011 | 1.6295E-011 |

| | | | | | | | |
|---|---|---|---|---|---|---|---|
| | | BFHDay2 | 6.61057E-012 | 7.33274E-012 | .374 | -8.3257E-012 | 2.1547E-011 |
| | | PFHDay2 | 9.43667E-012 | 7.33274E-012 | .207 | -5.4996E-012 | 2.4373E-011 |
| | | PMHDay2 | -1.06335E-011 | 7.33274E-012 | .157 | -2.5570E-011 | 4.3028E-012 |
| | PMHDay2 | BFHDay1 | 1.66764E-011* | 7.33274E-012 | .030 | 1.7401E-012 | 3.1613E-011 |
| | | PFHDay1 | 2.38199E-011* | 7.33274E-012 | .003 | 8.8836E-012 | 3.8756E-011 |
| | | BMHDay1 | 1.77144E-011* | 7.33274E-012 | .022 | 2.7781E-012 | 3.2651E-011 |
| | | PMHDay1 | 1.19924E-011 | 7.33274E-012 | .112 | -2.9439E-012 | 2.6929E-011 |
| | | BFHDay2 | 1.72440E-011* | 7.33274E-012 | .025 | 2.3077E-012 | 3.2180E-011 |
| | | PFHDay2 | 2.00701E-011* | 7.33274E-012 | .010 | 5.1338E-012 | 3.5006E-011 |
| | | BMHDay2 | 1.06335E-011 | 7.33274E-012 | .157 | -4.3028E-012 | 2.5570E-011 |
| araTyr | BFHDay1 | PFHDay1 | 1.26134E-012 | 8.93206E-013 | .173 | -6.0185E-013 | 3.1245E-012 |
| | | BMHDay1 | 8.16597E-013 | 8.26948E-013 | .335 | -9.0839E-013 | 2.5416E-012 |
| | | PMHDay1 | 7.41386E-013 | 8.26948E-013 | .381 | -9.8360E-013 | 2.4664E-012 |
| | | BFHDay2 | 6.01773E-013 | 8.26948E-013 | .475 | -1.1232E-012 | 2.3268E-012 |
| | | PFHDay2 | 9.36419E-013 | 8.93206E-013 | .307 | -9.2678E-013 | 2.7996E-012 |
| | | BMHDay2 | -1.73782E-013 | 1.01280E-012 | .865 | -2.2864E-012 | 1.9389E-012 |
| | | PMHDay2 | 6.51246E-013 | 8.26948E-013 | .440 | -1.0737E-012 | 2.3762E-012 |
| | PFHDay1 | BFHDay1 | -1.26134E-012 | 8.93206E-013 | .173 | -3.1245E-012 | 6.0185E-013 |
| | | BMHDay1 | -4.44745E-013 | 8.93206E-013 | .624 | -2.3079E-012 | 1.4185E-012 |

| | | | | | | |
|---|---|---|---|---|---|---|
| | PMHDay1 | -5.19956E-013 | 8.93206E-013 | .567 | -2.3832E-012 | 1.3432E-012 |
| | BFHDay2 | -6.59569E-013 | 8.93206E-013 | .469 | -2.5228E-012 | 1.2036E-012 |
| | PFHDay2 | -3.24922E-013 | 9.54878E-013 | .737 | -2.3168E-012 | 1.6669E-012 |
| | BMHDay2 | -1.43512E-012 | 1.06759E-012 | .194 | -3.6621E-012 | 7.9182E-013 |
| | PMHDay2 | -6.10095E-013 | 8.93206E-013 | .502 | -2.4733E-012 | 1.2531E-012 |
| BMHDay1 | BFHDay1 | -8.16597E-013 | 8.26948E-013 | .335 | -2.5416E-012 | 9.0839E-013 |
| | PFHDay1 | 4.44745E-013 | 8.93206E-013 | .624 | -1.4185E-012 | 2.3079E-012 |
| | PMHDay1 | -7.52110E-014 | 8.26948E-013 | .928 | -1.8002E-012 | 1.6498E-012 |
| | BFHDay2 | -2.14824E-013 | 8.26948E-013 | .798 | -1.9398E-012 | 1.5102E-012 |
| | PFHDay2 | 1.19823E-013 | 8.93206E-013 | .895 | -1.7434E-012 | 1.9830E-012 |
| | BMHDay2 | -9.90379E-013 | 1.01280E-012 | .340 | -3.1030E-012 | 1.1223E-012 |
| | PMHDay2 | -1.65350E-013 | 8.26948E-013 | .844 | -1.8903E-012 | 1.5596E-012 |
| PMHDay1 | BFHDay1 | -7.41386E-013 | 8.26948E-013 | .381 | -2.4664E-012 | 9.8360E-013 |
| | PFHDay1 | 5.19956E-013 | 8.93206E-013 | .567 | -1.3432E-012 | 2.3832E-012 |
| | BMHDay1 | 7.52110E-014 | 8.26948E-013 | .928 | -1.6498E-012 | 1.8002E-012 |
| | BFHDay2 | -1.39613E-013 | 8.26948E-013 | .868 | -1.8646E-012 | 1.5854E-012 |
| | PFHDay2 | 1.95034E-013 | 8.93206E-013 | .829 | -1.6682E-012 | 2.0582E-012 |
| | BMHDay2 | -9.15168E-013 | 1.01280E-012 | .377 | -3.0278E-012 | 1.1975E-012 |
| | PMHDay2 | -9.01393E-014 | 8.26948E-013 | .914 | -1.8151E-012 | 1.6348E-012 |

| | | | | | | |
|---|---|---|---|---|---|---|
| BFHDay2 | BFHDay1 | -6.01773E-013 | 8.26948E-013 | .475 | -2.3268E-012 | 1.1232E-012 |
| | PFHDay1 | 6.59569E-013 | 8.93206E-013 | .469 | -1.2036E-012 | 2.5228E-012 |
| | BMHDay1 | 2.14824E-013 | 8.26948E-013 | .798 | -1.5102E-012 | 1.9398E-012 |
| | PMHDay1 | 1.39613E-013 | 8.26948E-013 | .868 | -1.5854E-012 | 1.8646E-012 |
| | PFHDay2 | 3.34646E-013 | 8.93206E-013 | .712 | -1.5285E-012 | 2.1978E-012 |
| | BMHDay2 | -7.75555E-013 | 1.01280E-012 | .453 | -2.8882E-012 | 1.3371E-012 |
| | PMHDay2 | 4.94735E-014 | 8.26948E-013 | .953 | -1.6755E-012 | 1.7745E-012 |
| PFHDay2 | BFHDay1 | -9.36419E-013 | 8.93206E-013 | .307 | -2.7996E-012 | 9.2678E-013 |
| | PFHDay1 | 3.24922E-013 | 9.54878E-013 | .737 | -1.6669E-012 | 2.3168E-012 |
| | BMHDay1 | -1.19823E-013 | 8.93206E-013 | .895 | -1.9830E-012 | 1.7434E-012 |
| | PMHDay1 | -1.95034E-013 | 8.93206E-013 | .829 | -2.0582E-012 | 1.6682E-012 |
| | BFHDay2 | -3.34646E-013 | 8.93206E-013 | .712 | -2.1978E-012 | 1.5285E-012 |
| | BMHDay2 | -1.11020E-012 | 1.06759E-012 | .311 | -3.3371E-012 | 1.1167E-012 |
| | PMHDay2 | -2.85173E-013 | 8.93206E-013 | .753 | -2.1484E-012 | 1.5780E-012 |
| BMHDay2 | BFHDay1 | 1.73782E-013 | 1.01280E-012 | .865 | -1.9389E-012 | 2.2864E-012 |
| | PFHDay1 | 1.43512E-012 | 1.06759E-012 | .194 | -7.9182E-013 | 3.6621E-012 |
| | BMHDay1 | 9.90379E-013 | 1.01280E-012 | .340 | -1.1223E-012 | 3.1030E-012 |
| | PMHDay1 | 9.15168E-013 | 1.01280E-012 | .377 | -1.1975E-012 | 3.0278E-012 |
| | BFHDay2 | 7.75555E-013 | 1.01280E-012 | .453 | -1.3371E-012 | 2.8882E-012 |

| | | | | | | | |
|---|---|---|---|---|---|---|---|
| | | PFHDay2 | 1.11020E-012 | 1.06759E-012 | .311 | -1.1167E-012 | 3.3371E-012 |
| | | PMHDay2 | 8.25028E-013 | 1.01280E-012 | .425 | -1.2876E-012 | 2.9377E-012 |
| | PMHDay2 | BFHDay1 | -6.51246E-013 | 8.26948E-013 | .440 | -2.3762E-012 | 1.0737E-012 |
| | | PFHDay1 | 6.10095E-013 | 8.93206E-013 | .502 | -1.2531E-012 | 2.4733E-012 |
| | | BMHDay1 | 1.65350E-013 | 8.26948E-013 | .844 | -1.5596E-012 | 1.8903E-012 |
| | | PMHDay1 | 9.01393E-014 | 8.26948E-013 | .914 | -1.6348E-012 | 1.8151E-012 |
| | | BFHDay2 | -4.94735E-014 | 8.26948E-013 | .953 | -1.7745E-012 | 1.6755E-012 |
| | | PFHDay2 | 2.85173E-013 | 8.93206E-013 | .753 | -1.5780E-012 | 2.1484E-012 |
| | | BMHDay2 | -8.25028E-013 | 1.01280E-012 | .425 | -2.9377E-012 | 1.2876E-012 |
| palVal | BFHDay1 | PFHDay1 | -1.37446E-011 | 1.14049E-011 | .237 | -3.7005E-011 | 9.5158E-012 |
| | | BMHDay1 | -1.65458E-011 | 1.14049E-011 | .157 | -3.9806E-011 | 6.7146E-012 |
| | | PMHDay1 | -2.53047E-011* | 1.14049E-011 | .034 | -4.8565E-011 | -2.0443E-012 |
| | | BFHDay2 | -2.84670E-012 | 1.14049E-011 | .805 | -2.6107E-011 | 2.0414E-011 |
| | | PFHDay2 | -2.02005E-011 | 1.14049E-011 | .086 | -4.3461E-011 | 3.0599E-012 |
| | | BMHDay2 | -2.67485E-012 | 1.14049E-011 | .816 | -2.5935E-011 | 2.0586E-011 |
| | | PMHDay2 | -1.11979E-011 | 1.20967E-011 | .362 | -3.5869E-011 | 1.3474E-011 |
| | PFHDay1 | BFHDay1 | 1.37446E-011 | 1.14049E-011 | .237 | -9.5158E-012 | 3.7005E-011 |
| | | BMHDay1 | -2.80123E-012 | 1.14049E-011 | .808 | -2.6062E-011 | 2.0459E-011 |
| | | PMHDay1 | -1.15601E-011 | 1.14049E-011 | .319 | -3.4821E-011 | 1.1700E-011 |

| | | | | | | |
|---|---|---|---|---|---|---|
| | BFHDay2 | 1.08979E-011 | 1.14049E-011 | .347 | -1.2363E-011 | 3.4158E-011 |
| | PFHDay2 | -6.45594E-012 | 1.14049E-011 | .575 | -2.9716E-011 | 1.6805E-011 |
| | BMHDay2 | 1.10697E-011 | 1.14049E-011 | .339 | -1.2191E-011 | 3.4330E-011 |
| | PMHDay2 | 2.54673E-012 | 1.20967E-011 | .835 | -2.2125E-011 | 2.7218E-011 |
| BMHDay1 | BFHDay1 | 1.65458E-011 | 1.14049E-011 | .157 | -6.7146E-012 | 3.9806E-011 |
| | PFHDay1 | 2.80123E-012 | 1.14049E-011 | .808 | -2.0459E-011 | 2.6062E-011 |
| | PMHDay1 | -8.75891E-012 | 1.14049E-011 | .448 | -3.2019E-011 | 1.4502E-011 |
| | BFHDay2 | 1.36991E-011 | 1.14049E-011 | .239 | -9.5613E-012 | 3.6960E-011 |
| | PFHDay2 | -3.65471E-012 | 1.14049E-011 | .751 | -2.6915E-011 | 1.9606E-011 |
| | BMHDay2 | 1.38710E-011 | 1.14049E-011 | .233 | -9.3895E-012 | 3.7131E-011 |
| | PMHDay2 | 5.34796E-012 | 1.20967E-011 | .661 | -1.9323E-011 | 3.0019E-011 |
| PMHDay1 | BFHDay1 | 2.53047E-011* | 1.14049E-011 | .034 | 2.0443E-012 | 4.8565E-011 |
| | PFHDay1 | 1.15601E-011 | 1.14049E-011 | .319 | -1.1700E-011 | 3.4821E-011 |
| | BMHDay1 | 8.75891E-012 | 1.14049E-011 | .448 | -1.4502E-011 | 3.2019E-011 |
| | BFHDay2 | 2.24580E-011 | 1.14049E-011 | .058 | -8.0241E-013 | 4.5718E-011 |
| | PFHDay2 | 5.10420E-012 | 1.14049E-011 | .658 | -1.8156E-011 | 2.8365E-011 |
| | BMHDay2 | 2.26299E-011 | 1.14049E-011 | .056 | -6.3056E-013 | 4.5890E-011 |
| | PMHDay2 | 1.41069E-011 | 1.20967E-011 | .252 | -1.0565E-011 | 3.8778E-011 |
| BFHDay2 | BFHDay1 | 2.84670E-012 | 1.14049E-011 | .805 | -2.0414E-011 | 2.6107E-011 |

| | | | | | | |
|---|---|---|---|---|---|---|
| | PFHDay1 | -1.08979E-011 | 1.14049E-011 | .347 | -3.4158E-011 | 1.2363E-011 |
| | BMHDay1 | -1.36991E-011 | 1.14049E-011 | .239 | -3.6960E-011 | 9.5613E-012 |
| | PMHDay1 | -2.24580E-011 | 1.14049E-011 | .058 | -4.5718E-011 | 8.0241E-013 |
| | PFHDay2 | -1.73538E-011 | 1.14049E-011 | .138 | -4.0614E-011 | 5.9066E-012 |
| | BMHDay2 | 1.71850E-013 | 1.14049E-011 | .988 | -2.3089E-011 | 2.3432E-011 |
| | PMHDay2 | -8.35116E-012 | 1.20967E-011 | .495 | -3.3023E-011 | 1.6320E-011 |
| PFHDay2 | BFHDay1 | 2.02005E-011 | 1.14049E-011 | .086 | -3.0599E-012 | 4.3461E-011 |
| | PFHDay1 | 6.45594E-012 | 1.14049E-011 | .575 | -1.6805E-011 | 2.9716E-011 |
| | BMHDay1 | 3.65471E-012 | 1.14049E-011 | .751 | -1.9606E-011 | 2.6915E-011 |
| | PMHDay1 | -5.10420E-012 | 1.14049E-011 | .658 | -2.8365E-011 | 1.8156E-011 |
| | BFHDay2 | 1.73538E-011 | 1.14049E-011 | .138 | -5.9066E-012 | 4.0614E-011 |
| | BMHDay2 | 1.75257E-011 | 1.14049E-011 | .135 | -5.7348E-012 | 4.0786E-011 |
| | PMHDay2 | 9.00267E-012 | 1.20967E-011 | .462 | -1.5669E-011 | 3.3674E-011 |
| BMHDay2 | BFHDay1 | 2.67485E-012 | 1.14049E-011 | .816 | -2.0586E-011 | 2.5935E-011 |
| | PFHDay1 | -1.10697E-011 | 1.14049E-011 | .339 | -3.4330E-011 | 1.2191E-011 |
| | BMHDay1 | -1.38710E-011 | 1.14049E-011 | .233 | -3.7131E-011 | 9.3895E-012 |
| | PMHDay1 | -2.26299E-011 | 1.14049E-011 | .056 | -4.5890E-011 | 6.3056E-013 |
| | BFHDay2 | -1.71850E-013 | 1.14049E-011 | .988 | -2.3432E-011 | 2.3089E-011 |
| | PFHDay2 | -1.75257E-011 | 1.14049E-011 | .135 | -4.0786E-011 | 5.7348E-012 |

| | | | | | | | |
|---|---|---|---|---|---|---|---|
| | | PMHDay2 | -8.52301E-012 | 1.20967E-011 | .486 | -3.3194E-011 | 1.6148E-011 |
| | PMHDay2 | BFHDay1 | 1.11979E-011 | 1.20967E-011 | .362 | -1.3474E-011 | 3.5869E-011 |
| | | PFHDay1 | -2.54673E-012 | 1.20967E-011 | .835 | -2.7218E-011 | 2.2125E-011 |
| | | BMHDay1 | -5.34796E-012 | 1.20967E-011 | .661 | -3.0019E-011 | 1.9323E-011 |
| | | PMHDay1 | -1.41069E-011 | 1.20967E-011 | .252 | -3.8778E-011 | 1.0565E-011 |
| | | BFHDay2 | 8.35116E-012 | 1.20967E-011 | .495 | -1.6320E-011 | 3.3023E-011 |
| | | PFHDay2 | -9.00267E-012 | 1.20967E-011 | .462 | -3.3674E-011 | 1.5669E-011 |
| | | BMHDay2 | 8.52301E-012 | 1.20967E-011 | .486 | -1.6148E-011 | 3.3194E-011 |
| steVal | BFHDay1 | PFHDay1 | -3.50027E-012 | 2.13459E-012 | .112 | -7.8660E-012 | 8.6545E-013 |
| | | BMHDay1 | -1.62135E-012 | 2.13459E-012 | .454 | -5.9871E-012 | 2.7444E-012 |
| | | PMHDay1 | -3.03157E-012 | 2.13459E-012 | .166 | -7.3973E-012 | 1.3342E-012 |
| | | BFHDay2 | -1.02486E-012 | 2.46481E-012 | .681 | -6.0660E-012 | 4.0162E-012 |
| | | PFHDay2 | -4.70450E-012* | 2.13459E-012 | .036 | -9.0702E-012 | -3.3878E-013 |
| | | BMHDay2 | -1.07424E-013 | 2.13459E-012 | .960 | -4.4731E-012 | 4.2583E-012 |
| | | PMHDay2 | 2.47983E-013 | 2.26407E-012 | .914 | -4.3826E-012 | 4.8785E-012 |
| | PFHDay1 | BFHDay1 | 3.50027E-012 | 2.13459E-012 | .112 | -8.6545E-013 | 7.8660E-012 |
| | | BMHDay1 | 1.87891E-012 | 2.13459E-012 | .386 | -2.4868E-012 | 6.2446E-012 |
| | | PMHDay1 | 4.68702E-013 | 2.13459E-012 | .828 | -3.8970E-012 | 4.8344E-012 |
| | | BFHDay2 | 2.47541E-012 | 2.46481E-012 | .324 | -2.5657E-012 | 7.5165E-012 |

| | | | | | | |
|---|---|---|---|---|---|---|
| | PFHDay2 | -1.20423E-012 | 2.13459E-012 | .577 | -5.5699E-012 | 3.1615E-012 |
| | BMHDay2 | 3.39284E-012 | 2.13459E-012 | .123 | -9.7287E-013 | 7.7586E-012 |
| | PMHDay2 | 3.74825E-012 | 2.26407E-012 | .109 | -8.8229E-013 | 8.3788E-012 |
| BMHDay1 | BFHDay1 | 1.62135E-012 | 2.13459E-012 | .454 | -2.7444E-012 | 5.9871E-012 |
| | PFHDay1 | -1.87891E-012 | 2.13459E-012 | .386 | -6.2446E-012 | 2.4868E-012 |
| | PMHDay1 | -1.41021E-012 | 2.13459E-012 | .514 | -5.7759E-012 | 2.9555E-012 |
| | BFHDay2 | 5.96499E-013 | 2.46481E-012 | .810 | -4.4446E-012 | 5.6376E-012 |
| | PFHDay2 | -3.08314E-012 | 2.13459E-012 | .159 | -7.4489E-012 | 1.2826E-012 |
| | BMHDay2 | 1.51393E-012 | 2.13459E-012 | .484 | -2.8518E-012 | 5.8796E-012 |
| | PMHDay2 | 1.86934E-012 | 2.26407E-012 | .416 | -2.7612E-012 | 6.4999E-012 |
| PMHDay1 | BFHDay1 | 3.03157E-012 | 2.13459E-012 | .166 | -1.3342E-012 | 7.3973E-012 |
| | PFHDay1 | -4.68702E-013 | 2.13459E-012 | .828 | -4.8344E-012 | 3.8970E-012 |
| | BMHDay1 | 1.41021E-012 | 2.13459E-012 | .514 | -2.9555E-012 | 5.7759E-012 |
| | BFHDay2 | 2.00671E-012 | 2.46481E-012 | .422 | -3.0344E-012 | 7.0478E-012 |
| | PFHDay2 | -1.67293E-012 | 2.13459E-012 | .440 | -6.0386E-012 | 2.6928E-012 |
| | BMHDay2 | 2.92414E-012 | 2.13459E-012 | .181 | -1.4416E-012 | 7.2899E-012 |
| | PMHDay2 | 3.27955E-012 | 2.26407E-012 | .158 | -1.3510E-012 | 7.9101E-012 |
| BFHDay2 | BFHDay1 | 1.02486E-012 | 2.46481E-012 | .681 | -4.0162E-012 | 6.0660E-012 |
| | PFHDay1 | -2.47541E-012 | 2.46481E-012 | .324 | -7.5165E-012 | 2.5657E-012 |

| | | | | | | |
|---|---|---|---|---|---|---|
| | BMHDay1 | -5.96499E-013 | 2.46481E-012 | .810 | -5.6376E-012 | 4.4446E-012 |
| | PMHDay1 | -2.00671E-012 | 2.46481E-012 | .422 | -7.0478E-012 | 3.0344E-012 |
| | PFHDay2 | -3.67964E-012 | 2.46481E-012 | .146 | -8.7207E-012 | 1.3615E-012 |
| | BMHDay2 | 9.17431E-013 | 2.46481E-012 | .712 | -4.1237E-012 | 5.9585E-012 |
| | PMHDay2 | 1.27284E-012 | 2.57776E-012 | .625 | -3.9993E-012 | 6.5449E-012 |
| PFHDay2 | BFHDay1 | 4.70450E-012* | 2.13459E-012 | .036 | 3.3878E-013 | 9.0702E-012 |
| | PFHDay1 | 1.20423E-012 | 2.13459E-012 | .577 | -3.1615E-012 | 5.5699E-012 |
| | BMHDay1 | 3.08314E-012 | 2.13459E-012 | .159 | -1.2826E-012 | 7.4489E-012 |
| | PMHDay1 | 1.67293E-012 | 2.13459E-012 | .440 | -2.6928E-012 | 6.0386E-012 |
| | BFHDay2 | 3.67964E-012 | 2.46481E-012 | .146 | -1.3615E-012 | 8.7207E-012 |
| | BMHDay2 | 4.59707E-012* | 2.13459E-012 | .040 | 2.3135E-013 | 8.9628E-012 |
| | PMHDay2 | 4.95248E-012* | 2.26407E-012 | .037 | 3.2194E-013 | 9.5830E-012 |
| BMHDay2 | BFHDay1 | 1.07424E-013 | 2.13459E-012 | .960 | -4.2583E-012 | 4.4731E-012 |
| | PFHDay1 | -3.39284E-012 | 2.13459E-012 | .123 | -7.7586E-012 | 9.7287E-013 |
| | BMHDay1 | -1.51393E-012 | 2.13459E-012 | .484 | -5.8796E-012 | 2.8518E-012 |
| | PMHDay1 | -2.92414E-012 | 2.13459E-012 | .181 | -7.2899E-012 | 1.4416E-012 |
| | BFHDay2 | -9.17431E-013 | 2.46481E-012 | .712 | -5.9585E-012 | 4.1237E-012 |
| | PFHDay2 | -4.59707E-012* | 2.13459E-012 | .040 | -8.9628E-012 | -2.3135E-013 |
| | PMHDay2 | 3.55407E-013 | 2.26407E-012 | .876 | -4.2751E-012 | 4.9860E-012 |

| | | | | | | | |
|---|---|---|---|---|---|---|---|
| | PMHDay2 | BFHDay1 | -2.47983E-013 | 2.26407E-012 | .914 | -4.8785E-012 | 4.3826E-012 |
| | | PFHDay1 | -3.74825E-012 | 2.26407E-012 | .109 | -8.3788E-012 | 8.8229E-013 |
| | | BMHDay1 | -1.86934E-012 | 2.26407E-012 | .416 | -6.4999E-012 | 2.7612E-012 |
| | | PMHDay1 | -3.27955E-012 | 2.26407E-012 | .158 | -7.9101E-012 | 1.3510E-012 |
| | | BFHDay2 | -1.27284E-012 | 2.57776E-012 | .625 | -6.5449E-012 | 3.9993E-012 |
| | | PFHDay2 | -4.95248E-012* | 2.26407E-012 | .037 | -9.5830E-012 | -3.2194E-013 |
| | | BMHDay2 | -3.55407E-013 | 2.26407E-012 | .876 | -4.9860E-012 | 4.2751E-012 |
| oleVal | BFHDay1 | PFHDay1 | -1.10953E-012 | 1.16533E-012 | .349 | -3.4895E-012 | 1.2704E-012 |
| | | BMHDay1 | -1.71864E-012 | 1.16533E-012 | .151 | -4.0986E-012 | 6.6128E-013 |
| | | PMHDay1 | -3.00700E-012* | 1.16533E-012 | .015 | -5.3869E-012 | -6.2708E-013 |
| | | BFHDay2 | -2.11652E-013 | 1.23602E-012 | .865 | -2.7359E-012 | 2.3126E-012 |
| | | PFHDay2 | -1.97486E-012 | 1.16533E-012 | .100 | -4.3548E-012 | 4.0506E-013 |
| | | BMHDay2 | -1.32511E-013 | 1.16533E-012 | .910 | -2.5124E-012 | 2.2474E-012 |
| | | PMHDay2 | -1.12973E-012 | 1.23602E-012 | .368 | -3.6540E-012 | 1.3946E-012 |
| | PFHDay1 | BFHDay1 | 1.10953E-012 | 1.16533E-012 | .349 | -1.2704E-012 | 3.4895E-012 |
| | | BMHDay1 | -6.09102E-013 | 1.16533E-012 | .605 | -2.9890E-012 | 1.7708E-012 |
| | | PMHDay1 | -1.89747E-012 | 1.16533E-012 | .114 | -4.2774E-012 | 4.8245E-013 |
| | | BFHDay2 | 8.97882E-013 | 1.23602E-012 | .473 | -1.6264E-012 | 3.4222E-012 |
| | | PFHDay2 | -8.65323E-013 | 1.16533E-012 | .464 | -3.2452E-012 | 1.5146E-012 |

| | | | | | | |
|---|---|---|---|---|---|---|
| | | BMHDay2 | 9.77024E-013 | 1.16533E-012 | .408 | -1.4029E-012 | 3.3569E-012 |
| | | PMHDay2 | -2.01973E-014 | 1.23602E-012 | .987 | -2.5445E-012 | 2.5041E-012 |
| | BMHDay1 | BFHDay1 | 1.71864E-012 | 1.16533E-012 | .151 | -6.6128E-013 | 4.0986E-012 |
| | | PFHDay1 | 6.09102E-013 | 1.16533E-012 | .605 | -1.7708E-012 | 2.9890E-012 |
| | | PMHDay1 | -1.28836E-012 | 1.16533E-012 | .278 | -3.6683E-012 | 1.0916E-012 |
| | | BFHDay2 | 1.50698E-012 | 1.23602E-012 | .232 | -1.0173E-012 | 4.0313E-012 |
| | | PFHDay2 | -2.56221E-013 | 1.16533E-012 | .827 | -2.6361E-012 | 2.1237E-012 |
| | | BMHDay2 | 1.58613E-012 | 1.16533E-012 | .184 | -7.9379E-013 | 3.9660E-012 |
| | | PMHDay2 | 5.88904E-013 | 1.23602E-012 | .637 | -1.9354E-012 | 3.1132E-012 |
| | PMHDay1 | BFHDay1 | 3.00700E-012* | 1.16533E-012 | .015 | 6.2708E-013 | 5.3869E-012 |
| | | PFHDay1 | 1.89747E-012 | 1.16533E-012 | .114 | -4.8245E-013 | 4.2774E-012 |
| | | BMHDay1 | 1.28836E-012 | 1.16533E-012 | .278 | -1.0916E-012 | 3.6683E-012 |
| | | BFHDay2 | 2.79535E-012* | 1.23602E-012 | .031 | 2.7106E-013 | 5.3196E-012 |
| | | PFHDay2 | 1.03214E-012 | 1.16533E-012 | .383 | -1.3478E-012 | 3.4121E-012 |
| | | BMHDay2 | 2.87449E-012* | 1.16533E-012 | .020 | 4.9457E-013 | 5.2544E-012 |
| | | PMHDay2 | 1.87727E-012 | 1.23602E-012 | .139 | -6.4702E-013 | 4.4016E-012 |
| | BFHDay2 | BFHDay1 | 2.11652E-013 | 1.23602E-012 | .865 | -2.3126E-012 | 2.7359E-012 |
| | | PFHDay1 | -8.97882E-013 | 1.23602E-012 | .473 | -3.4222E-012 | 1.6264E-012 |
| | | BMHDay1 | -1.50698E-012 | 1.23602E-012 | .232 | -4.0313E-012 | 1.0173E-012 |

| | | | | | | | |
|---|---|---|---|---|---|---|---|
| | | PMHDay1 | -2.79535E-012* | 1.23602E-012 | .031 | -5.3196E-012 | -2.7106E-013 |
| | | PFHDay2 | -1.76320E-012 | 1.23602E-012 | .164 | -4.2875E-012 | 7.6108E-013 |
| | | BMHDay2 | 7.91417E-014 | 1.23602E-012 | .949 | -2.4451E-012 | 2.6034E-012 |
| | | PMHDay2 | -9.18079E-013 | 1.30288E-012 | .486 | -3.5789E-012 | 1.7428E-012 |
| | PFHDay2 | BFHDay1 | 1.97486E-012 | 1.16533E-012 | .100 | -4.0506E-013 | 4.3548E-012 |
| | | PFHDay1 | 8.65323E-013 | 1.16533E-012 | .464 | -1.5146E-012 | 3.2452E-012 |
| | | BMHDay1 | 2.56221E-013 | 1.16533E-012 | .827 | -2.1237E-012 | 2.6361E-012 |
| | | PMHDay1 | -1.03214E-012 | 1.16533E-012 | .383 | -3.4121E-012 | 1.3478E-012 |
| | | BFHDay2 | 1.76320E-012 | 1.23602E-012 | .164 | -7.6108E-013 | 4.2875E-012 |
| | | BMHDay2 | 1.84235E-012 | 1.16533E-012 | .124 | -5.3757E-013 | 4.2223E-012 |
| | | PMHDay2 | 8.45126E-013 | 1.23602E-012 | .499 | -1.6792E-012 | 3.3694E-012 |
| | BMHDay2 | BFHDay1 | 1.32511E-013 | 1.16533E-012 | .910 | -2.2474E-012 | 2.5124E-012 |
| | | PFHDay1 | -9.77024E-013 | 1.16533E-012 | .408 | -3.3569E-012 | 1.4029E-012 |
| | | BMHDay1 | -1.58613E-012 | 1.16533E-012 | .184 | -3.9660E-012 | 7.9379E-013 |
| | | PMHDay1 | -2.87449E-012* | 1.16533E-012 | .020 | -5.2544E-012 | -4.9457E-013 |
| | | BFHDay2 | -7.91417E-014 | 1.23602E-012 | .949 | -2.6034E-012 | 2.4451E-012 |
| | | PFHDay2 | -1.84235E-012 | 1.16533E-012 | .124 | -4.2223E-012 | 5.3757E-013 |
| | | PMHDay2 | -9.97221E-013 | 1.23602E-012 | .426 | -3.5215E-012 | 1.5271E-012 |
| | PMHDay2 | BFHDay1 | 1.12973E-012 | 1.23602E-012 | .368 | -1.3946E-012 | 3.6540E-012 |

| | | | | | | | |
|---|---|---|---|---|---|---|---|
| | | PFHDay1 | 2.01973E-014 | 1.23602E-012 | .987 | -2.5041E-012 | 2.5445E-012 |
| | | BMHDay1 | -5.88904E-013 | 1.23602E-012 | .637 | -3.1132E-012 | 1.9354E-012 |
| | | PMHDay1 | -1.87727E-012 | 1.23602E-012 | .139 | -4.4016E-012 | 6.4702E-013 |
| | | BFHDay2 | 9.18079E-013 | 1.30288E-012 | .486 | -1.7428E-012 | 3.5789E-012 |
| | | PFHDay2 | -8.45126E-013 | 1.23602E-012 | .499 | -3.3694E-012 | 1.6792E-012 |
| | | BMHDay2 | 9.97221E-013 | 1.23602E-012 | .426 | -1.5271E-012 | 3.5215E-012 |
| linVal | BFHDay1 | PFHDay1 | -1.27350E-012 | 1.89569E-012 | .507 | -5.1567E-012 | 2.6097E-012 |
| | | BMHDay1 | -1.71528E-012 | 1.78728E-012 | .345 | -5.3764E-012 | 1.9458E-012 |
| | | PMHDay1 | -4.55197E-012* | 1.78728E-012 | .017 | -8.2130E-012 | -8.9090E-013 |
| | | BFHDay2 | 1.21314E-013 | 1.78728E-012 | .946 | -3.5398E-012 | 3.7824E-012 |
| | | PFHDay2 | -8.77974E-013 | 1.78728E-012 | .627 | -4.5390E-012 | 2.7831E-012 |
| | | BMHDay2 | 7.86695E-013 | 2.06377E-012 | .706 | -3.4407E-012 | 5.0141E-012 |
| | | PMHDay2 | -3.03148E-013 | 1.89569E-012 | .874 | -4.1863E-012 | 3.5800E-012 |
| | PFHDay1 | BFHDay1 | 1.27350E-012 | 1.89569E-012 | .507 | -2.6097E-012 | 5.1567E-012 |
| | | BMHDay1 | -4.41786E-013 | 1.89569E-012 | .817 | -4.3249E-012 | 3.4414E-012 |
| | | PMHDay1 | -3.27847E-012 | 1.89569E-012 | .095 | -7.1616E-012 | 6.0468E-013 |
| | | BFHDay2 | 1.39481E-012 | 1.89569E-012 | .468 | -2.4883E-012 | 5.2780E-012 |
| | | PFHDay2 | 3.95524E-013 | 1.89569E-012 | .836 | -3.4876E-012 | 4.2787E-012 |
| | | BMHDay2 | 2.06019E-012 | 2.15834E-012 | .348 | -2.3610E-012 | 6.4814E-012 |

| | | | | | | |
|---|---|---|---|---|---|---|
| | PMHDay2 | 9.70350E-013 | 1.99824E-012 | .631 | -3.1229E-012 | 5.0636E-012 |
| BMHDay1 | BFHDay1 | 1.71528E-012 | 1.78728E-012 | .345 | -1.9458E-012 | 5.3764E-012 |
| | PFHDay1 | 4.41786E-013 | 1.89569E-012 | .817 | -3.4414E-012 | 4.3249E-012 |
| | PMHDay1 | -2.83669E-012 | 1.78728E-012 | .124 | -6.4978E-012 | 8.2438E-013 |
| | BFHDay2 | 1.83660E-012 | 1.78728E-012 | .313 | -1.8245E-012 | 5.4977E-012 |
| | PFHDay2 | 8.37310E-013 | 1.78728E-012 | .643 | -2.8238E-012 | 4.4984E-012 |
| | BMHDay2 | 2.50198E-012 | 2.06377E-012 | .236 | -1.7255E-012 | 6.7294E-012 |
| | PMHDay2 | 1.41214E-012 | 1.89569E-012 | .463 | -2.4710E-012 | 5.2953E-012 |
| PMHDay1 | BFHDay1 | 4.55197E-012* | 1.78728E-012 | .017 | 8.9090E-013 | 8.2130E-012 |
| | PFHDay1 | 3.27847E-012 | 1.89569E-012 | .095 | -6.0468E-013 | 7.1616E-012 |
| | BMHDay1 | 2.83669E-012 | 1.78728E-012 | .124 | -8.2438E-013 | 6.4978E-012 |
| | BFHDay2 | 4.67329E-012* | 1.78728E-012 | .014 | 1.0122E-012 | 8.3344E-012 |
| | PFHDay2 | 3.67400E-012* | 1.78728E-012 | .049 | 1.2927E-014 | 7.3351E-012 |
| | BMHDay2 | 5.33867E-012* | 2.06377E-012 | .015 | 1.1112E-012 | 9.5661E-012 |
| | PMHDay2 | 4.24882E-012* | 1.89569E-012 | .033 | 3.6567E-013 | 8.1320E-012 |
| BFHDay2 | BFHDay1 | -1.21314E-013 | 1.78728E-012 | .946 | -3.7824E-012 | 3.5398E-012 |
| | PFHDay1 | -1.39481E-012 | 1.89569E-012 | .468 | -5.2780E-012 | 2.4883E-012 |
| | BMHDay1 | -1.83660E-012 | 1.78728E-012 | .313 | -5.4977E-012 | 1.8245E-012 |
| | PMHDay1 | -4.67329E-012* | 1.78728E-012 | .014 | -8.3344E-012 | -1.0122E-012 |

| | | | | | | |
|---|---|---|---|---|---|---|
| | | PFHDay2 | -9.99288E-013 | 1.78728E-012 | .581 | -4.6604E-012 | 2.6618E-012 |
| | | BMHDay2 | 6.65381E-013 | 2.06377E-012 | .750 | -3.5621E-012 | 4.8928E-012 |
| | | PMHDay2 | -4.24462E-013 | 1.89569E-012 | .824 | -4.3076E-012 | 3.4587E-012 |
| | PFHDay2 | BFHDay1 | 8.77974E-013 | 1.78728E-012 | .627 | -2.7831E-012 | 4.5390E-012 |
| | | PFHDay1 | -3.95524E-013 | 1.89569E-012 | .836 | -4.2787E-012 | 3.4876E-012 |
| | | BMHDay1 | -8.37310E-013 | 1.78728E-012 | .643 | -4.4984E-012 | 2.8238E-012 |
| | | PMHDay1 | -3.67400E-012* | 1.78728E-012 | .049 | -7.3351E-012 | -1.2927E-014 |
| | | BFHDay2 | 9.99288E-013 | 1.78728E-012 | .581 | -2.6618E-012 | 4.6604E-012 |
| | | BMHDay2 | 1.66467E-012 | 2.06377E-012 | .427 | -2.5628E-012 | 5.8921E-012 |
| | | PMHDay2 | 5.74826E-013 | 1.89569E-012 | .764 | -3.3083E-012 | 4.4580E-012 |
| | BMHDay2 | BFHDay1 | -7.86695E-013 | 2.06377E-012 | .706 | -5.0141E-012 | 3.4407E-012 |
| | | PFHDay1 | -2.06019E-012 | 2.15834E-012 | .348 | -6.4814E-012 | 2.3610E-012 |
| | | BMHDay1 | -2.50198E-012 | 2.06377E-012 | .236 | -6.7294E-012 | 1.7255E-012 |
| | | PMHDay1 | -5.33867E-012* | 2.06377E-012 | .015 | -9.5661E-012 | -1.1112E-012 |
| | | BFHDay2 | -6.65381E-013 | 2.06377E-012 | .750 | -4.8928E-012 | 3.5621E-012 |
| | | PFHDay2 | -1.66467E-012 | 2.06377E-012 | .427 | -5.8921E-012 | 2.5628E-012 |
| | | PMHDay2 | -1.08984E-012 | 2.15834E-012 | .618 | -5.5110E-012 | 3.3313E-012 |
| | PMHDay2 | BFHDay1 | 3.03148E-013 | 1.89569E-012 | .874 | -3.5800E-012 | 4.1863E-012 |
| | | PFHDay1 | -9.70350E-013 | 1.99824E-012 | .631 | -5.0636E-012 | 3.1229E-012 |

| | | | | | | | |
|---|---|---|---|---|---|---|---|
| | | BMHDay1 | -1.41214E-012 | 1.89569E-012 | .463 | -5.2953E-012 | 2.4710E-012 |
| | | PMHDay1 | -4.24882E-012* | 1.89569E-012 | .033 | -8.1320E-012 | -3.6567E-013 |
| | | BFHDay2 | 4.24462E-013 | 1.89569E-012 | .824 | -3.4587E-012 | 4.3076E-012 |
| | | PFHDay2 | -5.74826E-013 | 1.89569E-012 | .764 | -4.4580E-012 | 3.3083E-012 |
| | | BMHDay2 | 1.08984E-012 | 2.15834E-012 | .618 | -3.3313E-012 | 5.5110E-012 |
| docVal | BFHDay1 | PFHDay1 | -1.10267E-012 | 1.79209E-012 | .544 | -4.7936E-012 | 2.5882E-012 |
| | | BMHDay1 | 1.35929E-012 | 1.57401E-012 | .396 | -1.8825E-012 | 4.6010E-012 |
| | | PMHDay1 | 1.02540E-012 | 1.57401E-012 | .521 | -2.2163E-012 | 4.2671E-012 |
| | | BFHDay2 | 1.36028E-012 | 1.79209E-012 | .455 | -2.3306E-012 | 5.0512E-012 |
| | | PFHDay2 | 3.62600E-013 | 1.65916E-012 | .829 | -3.0545E-012 | 3.7797E-012 |
| | | BMHDay2 | 5.33709E-013 | 1.57401E-012 | .737 | -2.7080E-012 | 3.7755E-012 |
| | | PMHDay2 | -2.30443E-012 | 1.65916E-012 | .177 | -5.7215E-012 | 1.1127E-012 |
| | PFHDay1 | BFHDay1 | 1.10267E-012 | 1.79209E-012 | .544 | -2.5882E-012 | 4.7936E-012 |
| | | BMHDay1 | 2.46196E-012 | 1.71357E-012 | .163 | -1.0672E-012 | 5.9911E-012 |
| | | PMHDay1 | 2.12807E-012 | 1.71357E-012 | .226 | -1.4011E-012 | 5.6572E-012 |
| | | BFHDay2 | 2.46295E-012 | 1.91583E-012 | .210 | -1.4828E-012 | 6.4087E-012 |
| | | PFHDay2 | 1.46527E-012 | 1.79209E-012 | .421 | -2.2256E-012 | 5.1562E-012 |
| | | BMHDay2 | 1.63638E-012 | 1.71357E-012 | .349 | -1.8928E-012 | 5.1655E-012 |
| | | PMHDay2 | -1.20176E-012 | 1.79209E-012 | .509 | -4.8926E-012 | 2.4891E-012 |

| | | | | | | | |
|---|---|---|---|---|---|---|---|
| | BMHDay1 | BFHDay1 | -1.35929E-012 | 1.57401E-012 | .396 | -4.6010E-012 | 1.8825E-012 |
| | | PFHDay1 | -2.46196E-012 | 1.71357E-012 | .163 | -5.9911E-012 | 1.0672E-012 |
| | | PMHDay1 | -3.33892E-013 | 1.48400E-012 | .824 | -3.3902E-012 | 2.7225E-012 |
| | | BFHDay2 | 9.87333E-016 | 1.71357E-012 | 1.000 | -3.5282E-012 | 3.5302E-012 |
| | | PFHDay2 | -9.96691E-013 | 1.57401E-012 | .532 | -4.2384E-012 | 2.2451E-012 |
| | | BMHDay2 | -8.25582E-013 | 1.48400E-012 | .583 | -3.8819E-012 | 2.2308E-012 |
| | | PMHDay2 | -3.66372E-012* | 1.57401E-012 | .028 | -6.9055E-012 | -4.2197E-013 |
| | PMHDay1 | BFHDay1 | -1.02540E-012 | 1.57401E-012 | .521 | -4.2671E-012 | 2.2163E-012 |
| | | PFHDay1 | -2.12807E-012 | 1.71357E-012 | .226 | -5.6572E-012 | 1.4011E-012 |
| | | BMHDay1 | 3.33892E-013 | 1.48400E-012 | .824 | -2.7225E-012 | 3.3902E-012 |
| | | BFHDay2 | 3.34879E-013 | 1.71357E-012 | .847 | -3.1943E-012 | 3.8640E-012 |
| | | PFHDay2 | -6.62799E-013 | 1.57401E-012 | .677 | -3.9045E-012 | 2.5789E-012 |
| | | BMHDay2 | -4.91690E-013 | 1.48400E-012 | .743 | -3.5480E-012 | 2.5647E-012 |
| | | PMHDay2 | -3.32983E-012* | 1.57401E-012 | .045 | -6.5716E-012 | -8.8082E-014 |
| | BFHDay2 | BFHDay1 | -1.36028E-012 | 1.79209E-012 | .455 | -5.0512E-012 | 2.3306E-012 |
| | | PFHDay1 | -2.46295E-012 | 1.91583E-012 | .210 | -6.4087E-012 | 1.4828E-012 |
| | | BMHDay1 | -9.87333E-016 | 1.71357E-012 | 1.000 | -3.5302E-012 | 3.5282E-012 |
| | | PMHDay1 | -3.34879E-013 | 1.71357E-012 | .847 | -3.8640E-012 | 3.1943E-012 |
| | | PFHDay2 | -9.97678E-013 | 1.79209E-012 | .583 | -4.6886E-012 | 2.6932E-012 |

| | | | | | | |
|---|---|---|---|---|---|---|
| | BMHDay2 | -8.26569E-013 | 1.71357E-012 | .634 | -4.3557E-012 | 2.7026E-012 |
| | PMHDay2 | -3.66471E-012 | 1.79209E-012 | .052 | -7.3556E-012 | 2.6183E-014 |
| PFHDay2 | BFHDay1 | -3.62600E-013 | 1.65916E-012 | .829 | -3.7797E-012 | 3.0545E-012 |
| | PFHDay1 | -1.46527E-012 | 1.79209E-012 | .421 | -5.1562E-012 | 2.2256E-012 |
| | BMHDay1 | 9.96691E-013 | 1.57401E-012 | .532 | -2.2451E-012 | 4.2384E-012 |
| | PMHDay1 | 6.62799E-013 | 1.57401E-012 | .677 | -2.5789E-012 | 3.9045E-012 |
| | BFHDay2 | 9.97678E-013 | 1.79209E-012 | .583 | -2.6932E-012 | 4.6886E-012 |
| | BMHDay2 | 1.71109E-013 | 1.57401E-012 | .914 | -3.0706E-012 | 3.4129E-012 |
| | PMHDay2 | -2.66703E-012 | 1.65916E-012 | .121 | -6.0841E-012 | 7.5007E-013 |
| BMHDay2 | BFHDay1 | -5.33709E-013 | 1.57401E-012 | .737 | -3.7755E-012 | 2.7080E-012 |
| | PFHDay1 | -1.63638E-012 | 1.71357E-012 | .349 | -5.1655E-012 | 1.8928E-012 |
| | BMHDay1 | 8.25582E-013 | 1.48400E-012 | .583 | -2.2308E-012 | 3.8819E-012 |
| | PMHDay1 | 4.91690E-013 | 1.48400E-012 | .743 | -2.5647E-012 | 3.5480E-012 |
| | BFHDay2 | 8.26569E-013 | 1.71357E-012 | .634 | -2.7026E-012 | 4.3557E-012 |
| | PFHDay2 | -1.71109E-013 | 1.57401E-012 | .914 | -3.4129E-012 | 3.0706E-012 |
| | PMHDay2 | -2.83814E-012 | 1.57401E-012 | .083 | -6.0799E-012 | 4.0361E-013 |
| PMHDay2 | BFHDay1 | 2.30443E-012 | 1.65916E-012 | .177 | -1.1127E-012 | 5.7215E-012 |
| | PFHDay1 | 1.20176E-012 | 1.79209E-012 | .509 | -2.4891E-012 | 4.8926E-012 |
| | BMHDay1 | 3.66372E-012* | 1.57401E-012 | .028 | 4.2197E-013 | 6.9055E-012 |

| | | | | | | |
|---|---|---|---|---|---|---|
| | PMHDay1 | 3.32983E-012* | 1.57401E-012 | .045 | 8.8082E-014 | 6.5716E-012 |
| | BFHDay2 | 3.66471E-012 | 1.79209E-012 | .052 | -2.6183E-014 | 7.3556E-012 |
| | PFHDay2 | 2.66703E-012 | 1.65916E-012 | .121 | -7.5007E-013 | 6.0841E-012 |
| | BMHDay2 | 2.83814E-012 | 1.57401E-012 | .083 | -4.0361E-013 | 6.0799E-012 |

*. The mean difference is significant at the 0.05 level.
