# Supplementary material for: Sex differences in plasma endocannabinoids and related lipids before and after single and repeated mTBI: an exploratory study of endolipid plasma biomarkers
Source: Front Mol Neurosci. 2026 Jun 18;19:1707732. doi: 10.3389/fnmol.2026.1707732 (PMC13323484; doi:10.3389/fnmol.2026.1707732)
Supplement: Supplementary file 2 [file Data_Sheet_2.pdf]

Supplemental Figures

	Sex Difference Analysis				mTBI Analysis by Genetic Sex				Combinded Groups	
	B F Day 1 *vs* B M Day 1	PH F D1 *vs* PH M D1	B F D2 *vs* B M D2	PH F D2 *vs* PH M D2	PH F D1 relative to B F D1	PH M D1 relative to B M D1	PH F D2 relative to B F D2	PH M D2 relative to B M D2	PH D1 relative to B D1	PH D2 relative to B D2
N-acyl alanine										
N-palmitoyl alanine	0.0914989	0.11338	0.8012	0.7735923	0.266667	0.635538	0.41224	0.804086	0.300942	0.425334
N-stearoyl alanine	0.8085149	0.3835	0.2267	0.2863002	0.77542	0.975099	0.56357	0.895421	0.833867	0.672447
N-oleoyl alanine	0.1986729	0.212	0.81444	0.0890013	0.797045	0.006843	0.06203	0.16544	0.447152	0.053977
N-linoleoyl alanine	0.2970287	0.20297	0.67406	0.5897751	0.364262	0.658679	0.9407	0.098675	0.337372	0.576987
N-arachidonoyl alanine	0.009553	0.11488	0.287	BAL	0.054027	0.037398	BAL	0.09496	0.988047	0.037234
N-docosahexaenoyl alanine	0.4774381	0.51034	BAL	0.9360986	0.853715	0.666075	0.03221	BAL	0.753412	0.006771
N-acyl ethanolamine										
N-palmitoyl ethanolamine	0.6709869	0.81867	0.5763	0.6492657	0.280359	0.49693	0.83006	0.164523	0.202264	0.267469
N-stearoyl ethanolamine	0.1219766	0.00239	0.02265	0.0001628	0.589955	0.759095	0.22284	0.395816	0.951732	0.365617
N-oleoyl ethanolamine	0.9864747	0.94507	0.99823	0.6685717	0.791963	0.688914	0.61178	0.365937	0.617946	0.275936
N-linoleoyl ethanolamine	0.0205018	0.57082	0.8631	0.8142833	0.844235	0.051669	0.1847	0.337583	0.077857	0.081553
N-arachidonoyl ethanolamine	0.5752769	0.91817	0.62387	0.915751	0.548819	0.17171	0.99464	0.561924	0.179155	0.627138
N-docosahexaenoyl ethanolamine	0.57508	0.17066	0.73891	0.2300159	0.113727	0.72248	0.10053	0.9264	0.314844	0.189269
N-acyl GABA										
N-palmitoyl GABA	0.0855428	0.24004	0.85075	0.5157836	0.432208	0.433868	0.52518	0.784558	0.304282	0.544328
N-stearoyl GABA	0.2150089	0.91229	0.67248	0.8098876	0.162872	0.852409	0.98409	0.851263	0.347284	0.857218
N-oleoyl GABA	0.8722555	0.88669	0.48443	0.0847902	0.107177	0.366732	0.794	0.042109	0.087212	0.132448
N-linoleoyl GABA	0.9277775	0.10498	0.62386	0.2766856	0.822878	0.320367	0.05774	0.076148	0.299408	0.016863
N-arachidonoyl GABA	0.7404154	0.62245	0.92814	0.6340548	0.848857	0.468539	0.30429	0.391196	0.412893	0.144419
N-docosahexaenoyl GABA	0.8785978	0.84818	0.91664	0.2201941	0.977074	0.946626	0.79502	0.255117	0.976042	0.443387
N-acyl glycine										
N-palmitoyl glycine	0.0591935	0.87208	0.84912	0.9916782	0.161391	0.59942	0.04938	0.170831	0.151889	0.016346
N-stearoyl glycine	0.0411894	0.67171	0.22743	0.2067055	0.913289	0.025026	0.80218	0.032814	0.209226	0.165795
N-oleoyl glycine	0.0131839	0.25137	0.47028	0.229919	0.647748	0.049095	0.31477	0.129709	0.042055	0.067814
N-linoleoyl glycine	0.0116626	0.19117	0.54108	0.0570705	0.786058	0.014634	0.4647	0.027827	0.055027	0.035942
N-arachidonoyl glycine	0.2880452	BAL	0.86449	0.2767009	0.455076	BAL	0.77373	0.546077	0.190049	0.935043
N-docosahexaenoyl glycine	0.4762518	0.20439	0.17806	0.494413	0.475656	0.853542	0.35237	0.815988	0.533314	0.403885
N-acyl leucine										
N-palmitoyl leucine	0.0649264	0.40396	0.46855	0.722344	0.963387	0.057678	0.11594	0.411742	0.090061	0.112051
N-stearoyl leucine	0.1050655	0.11943	0.28744	0.2240368	0.385889	0.036617	0.23141	0.24662	0.244737	0.115952
N-oleoyl leucine	0.4260471	0.14288	0.46839	0.5511642	0.956173	0.031391	0.03341	0.4195	0.123803	0.134539
N-linoleoyl leucine	0.7266319	0.19905	0.11439	0.060133	0.300876	0.366487	0.24337	0.289814	0.825959	0.244366
N-docosahexaenoyl leucine	0.9352569	0.06676	0.63002	0.3536035	0.757157	0.029862	0.54263	0.287617	0.091875	0.93413
N-acyl methionine										
N-palmitoyl methionine	0.8227732	0.25523	0.02878	0.2339802	0.985681	0.196511	0.18127	0.743512	0.255927	0.401475
N-stearoyl methionine	0.1010347	0.0219	0.2453	0.8244894	0.43248	0.009103	0.27976	0.44556	0.086761	0.165839
N-oleoyl methionine	0.0959928	0.0563	0.20444	0.0615256	0.055845	0.063238	0.37905	0.183884	0.38348	0.189604
N-linoleoyl methionine	0.0519419	0.24764	0.09002	0.0097455	0.401557	0.124768	0.98877	0.133943	0.211558	0.24294
N-arachidonoyl methionine	0.975967	0.79425	0.2122	0.6138283	0.763083	0.953526	0.67028	0.379768	0.753345	0.381688
N-docosahexaenoyl methionine	0.010272	0.92373	0.2006	0.3477691	0.529653	0.192452	0.34367	0.512812	0.948032	0.261817
N- acyl phenylalanine										
N-palmitoyl phenylalanine	0.1767055	0.56366	0.62981	0.2200342	0.868	0.148925	0.27745	0.225765	0.16727	0.105411
N-stearoyl phenylalanine	0.9528506	0.17199	0.6447	0.2627822	0.684856	0.199814	0.77446	0.478436	0.337448	0.431433
N-oleoyl phenylalanine	0.0921463	0.23549	0.47708	0.0389219	0.184134	0.124954	0.77562	0.17255	0.755124	0.199291
N-linoleoyl phenylalanine	0.0488714	0.08905	0.07485	0.0221544	0.06337	0.071075	0.89088	0.452513	0.299115	0.589137
N-arachidonoyl phenylalanine	0.2907379	0.87382	0.39004	0.2359235	0.322738	0.807412	0.49928	0.718806	0.412574	0.513012
N-docosahexaenoyl phenylalanine	0.0574874	0.1459	0.29908	0.7629228	0.198821	0.04168	0.67375	0.805879	0.537375	0.793361

P=values of all interactions listed.

	Sex Difference Analysis				mTBI Analysis by Genetic Sex				Combinded Groups	
	B F Day 1 *vs* B M Day 1	PH F D1 *vs* PH M D1	B F D2 *vs* B M D2	PH F D2 *vs* PH M D2	PH F D1 relative to B F D1	PH M D1 relative to B M D1	PH F D2 relative to B F D2	PH M D2 relative to B M D2	PH D1 relative to B D1	PH D2 relative to B D2
***N*- acyl proline**										
N-palmitoyl proline	0.80348	0.599095	0.023191	0.405005	0.44687	0.109299	0.032497	0.89413	0.100727	0.11993
N-stearoyl proline	BAL	BAL	BAL	BAL	BAL	BAL	BAL	BAL	0.014864	0.396919
N-oleoyl proline	0.182925	0.0676	0.023787	0.81131	0.062058	0.171742	0.554169	0.250353	0.050241	0.659085
N-linoleoyl proline	0.177759	0.284585	0.491309	0.766931	0.172164	0.366521	0.282828	0.37928	0.640456	0.200887
N-arachidonoyl proline	0.82596	0.893055	0.014274	0.549048	0.918244	0.727803	0.438488	0.500007	0.752327	0.694637
N-docosahexaenoyl proline	0.526658	0.922449	0.576514	0.537944	0.658352	0.640282	0.971808	0.868348	0.812524	0.785997
N-acyl serine										
N-palmitoyl serine	0.309287	0.465478	0.218613	0.19618	0.380434	0.253589	0.641952	0.597976	0.234378	0.746309
N-stearoyl serine	0.831469	0.433992	0.934762	0.394288	0.777495	0.473277	0.356261	0.928052	0.701435	0.384456
N-oleoyl serine	0.151832	0.305855	0.367703	0.033514	0.634145	0.181561	0.04588	0.553294	0.15868	0.575248
N-linoleoyl serine	BAL	0.783349	0.35143	0.224011	0.110418	BAL	0.340052	0.71465	0.099657	0.472325
N-arachidonoyl serine	BAL	BAL	BAL	BAL	BAL	BAL	BAL	BAL	0.598752	0.475567
N-docosahexaenoyl serine	BAL	BAL	0.887362	BAL	BAL	BAL	BAL	0.949191	0.179906	0.277093
N-acyl taurine										
N-palmitoyl taurine	0.020723	0.318364	0.880112	0.914847	0.20875	0.077078	0.1123	0.076462	0.653982	0.010934
N-stearoyl taurine	0.230627	0.203998	0.206638	0.806366	0.670596	0.051201	0.479752	0.322056	0.054048	0.403719
N-oleoyl taurine	0.398912	0.607436	0.891555	0.168045	0.63696	0.09711	0.748573	0.026466	0.115407	0.113573
N-arachidonoyl taurine	0.371115	0.302235	0.415131	0.938669	0.273406	0.388786	0.761522	0.516088	0.712361	0.679061
N-acyl tryptophan										
N-palmitoyl tryptophan	0.39215	0.739492	0.746118	0.4225	0.898403	0.414397	0.878526	0.5685	0.49956	0.508918
N-stearoyl tryptophan	0.518025	0.604261	0.889812	0.620532	0.360812	0.78383	0.905116	0.618784	0.625332	0.751559
N-oleoyl tryptophan	0.888869	0.232148	0.305089	0.724921	0.097716	0.408913	0.026637	0.163666	0.427012	0.043395
N-linoleoyl tryptophan	BAL	BAL	BAL	0.758078	BAL	BAL	BAL	0.445158	0.267099	0.517199
N-arachidonoyl tryptophan	BAL	BAL	BAL	BAL	BAL	BAL	BAL	BAL	BAL	BAL
N-docosahexaenoyl tryptophan	BAL	BAL	BAL	BAL	BAL	BAL	BAL	BAL	BAL	BAL
N-acyl tyrosine										
N-palmitoyl tyrosine	0.399811	0.035359	0.185107	0.19126	0.219295	0.075844	0.590724	0.481165	0.809043	0.395883
N-stearoyl tyrosine	0.932152	0.011422	0.115657	0.383099	0.322523	0.520417	0.542888	0.853737	0.730047	0.699094
N-oleoyl tyrosine	0.317214	0.185047	0.142739	0.100529	0.155412	0.2885	0.429623	0.369175	0.90973	0.308727
N-linoleoyl tyrosine	0.856194	0.050422	0.288653	0.109218	0.233188	0.298736	0.468057	0.401588	0.863565	0.578065
N-arachidonoyl tyrosine	0.554551	0.106136	0.414005	0.616466	0.435368	0.861721	0.42204	0.4447	0.432251	0.361563
N-docosahexaenoyl tyrosine	0.914238	BAL	BAL	0.536073	BAL	0.762824	BAL	0.602439	0.757579	0.737982
N-acyl valine										
N-palmitoyl valine	0.541202	0.361286	0.152726	0.453991	0.868513	0.161056	0.059554	0.618637	0.185823	0.234406
N- stearoyl valine	0.944498	0.982431	0.308585	0.870956	0.156125	0.380906	0.169427	0.531923	0.112959	0.123495
N-oleoyl valine	0.762988	0.47681	0.211911	0.268612	0.238265	0.063272	0.160392	0.518424	0.02064	0.268957
N-linoleoyl valine	0.103169	0.237048	0.179819	0.162777	0.536002	0.090206	0.749571	0.293107	0.213514	0.300669
N-docosahexaenoyl valine	0.758426	0.265557	0.768434	0.923247	0.832905	0.242907	0.899128	0.764858	0.303743	0.980941
**2-acyl-sn-glycerol**										
2-palmitoyl-sn-glycerol	0.997327	0.112857	0.882771	0.518711	0.116983	0.94334	0.056548	0.518999	0.200301	0.090133
2-oleoyl-sn-glycerol	0.219627	0.488621	0.346903	0.357726	0.494455	0.424137	0.50839	0.180704	0.709664	0.132118
2-linoleoyl-sn-glycerol	0.019013	0.628299	0.068801	0.037569	0.513982	0.193971	0.81928	0.043222	0.647235	0.148422
2-arachidonoyl-sn-glycerol	0.006898	0.869403	0.576454	0.042974	0.090298	0.555761	0.281228	0.412267	0.751932	0.239502
**Free Fatty Acids**										
Oleic acid	0.030587	0.776932	0.591931	0.212559	0.875923	0.026508	0.046254	0.123226	0.168675	0.040026
Linoleic acid	0.005684	0.848046	0.318277	0.089935	0.490937	0.058091	0.097065	0.133351	0.341241	0.107855
Arachidonic acid	0.01566	0.043004	0.066499	0.070938	0.301848	0.054579	0.138313	0.367853	0.797528	0.137097
Eicosapentaenoic acid	0.023656	0.132021	0.870971	0.27117	0.238033	0.02033	0.330742	0.123526	0.305594	0.060198
Docosahexaenoic acid	0.037334	0.636596	0.343913	0.258797	0.145018	0.088146	0.235119	0.249597	0.658521	0.115852
**Prostaglandins**										
PGE_2	0.024026	0.036419	0.173964	0.297523	0.950321	0.843496	0.939287	0.729764	0.611016	0.779337
$PGF_{2\alpha}$	0.213595	0.118718	0.385731	0.109905	0.375166	0.258955	0.64172	0.019181	0.15962	0.348514
6-keto-$PGF_{1\alpha}$	BAL	BAL	BAL	BAL	BAL	BAL	BAL	BAL	BAL	BAL

	B F Day 1 *vs* B M Day 1	PH F D1 *vs* PH M D1	B F D2 *vs* B M D2	PH F D2 *vs* PH M D2	PH F D1 relative to B F D1	PH M D1 relative to B M D1	PH F D2 relative to B F D2	PH M D2 relative to B M D2	PH D1 relative to B D1	PH D2 relative to B D2
N-acyl alanine										
N-palmitoyl alanine	↑↑									
N-stearoyl alanine										
N-oleoyl alanine				↑↑↑↑		↑↑↑↑	↑↑↑↑			↑↑↑↑
N-linoleoyl alanine								↓		
N-arachidonoyl alanine	↑↑↑↑			BAL	↓↓↓	↑↑↑↑	BAL	↑↑		↑↑
N-docosahexaenoyl alanine			BAL				↓↓↓↓	BAL		↓↓↓↓
N-acyl ethanolamine										
N-palmitoyl ethanolamine										
N-stearoyl ethanolamine		↓↓	↓↓	↓↓						
N-oleoyl ethanolamine										
N-linoleoyl ethanolamine	↑↑					↑↑↑			↑	↑
N-arachidonoyl ethanolamine										
N-docosahexaenoyl ethanolamine										
N-acyl GABA										
N-palmitoyl GABA	↑↑									
N-stearoyl GABA										
N-oleoyl GABA				↓↓↓				↑↑↑	↑↑	
N-linoleoyl GABA							↑↑↑	↑↑↑		↑↑↑
N-arachidonoyl GABA										
N-docosahexaenoyl GABA										
N-acyl glycine										
N-palmitoyl glycine	↓↓						↑↑↑			↑↑↑
N-stearoyl glycine	↑↑					↑↑↑		↑↑		
N-oleoyl glycine	↑↑↑					↑↑↑↑			↑↑↑	↑↑↑
N-linoleoyl glycine	↑↑↑↑			↓↓↓		↑↑↑↑		↑↑↑	↑↑	↑↑↑
N-arachidonoyl glycine		BAL				BAL				
N-docosahexaenoyl glycine										
N-acyl leucine										
N-palmitoyl leucine	↑↑					↑↑↑			↑	
N-stearoyl leucine						↑↑↑				
N-oleoyl leucine						↑↑↑	↑↑			
N-linoleoyl leucine				↓↓↓						
N-docosahexaenoyl leucine		↓↓				↑↑			↑↑	
N-acyl methionine										
N-palmitoyl methionine			↓↓↓							
N-stearoyl methionine		↓↓↓				↑↑↑			↑↑	
N-oleoyl methionine	↑↑	↓↓↓		↓↓↓↓	↓↓	↑↑↑				
N-linoleoyl methionine	↑↑↑		↓↓↓↓	↓↓↓↓						
N-arachidonoyl methionine										
N-docosahexaenoyl methionine	↑↑									
N- acyl phenylalanine										
N-palmitoyl phenylalanine										
N-stearoyl phenylalanine										
N-oleoyl phenylalanine	↑↑			↓↓↓						
N-linoleoyl phenylalanine	↑↑	↓↓↓	↓↓↓	↓↓↓	↓↓	↑↑↑				
N-arachidonoyl phenylalanine										
N-docosahexaenoyl phenylalanine	↑↑↑					↑↑↑				

Heatmaps of all interactions listed.

	Sex Difference Analysis				mTBI Analysis by Genetic Sex				Combinded Groups	
	B F Day 1 *vs* B M Day 1	PH F D1 *vs* PH M D1	B F D2 *vs* B M D2	PH F D2 *vs* PH M D2	PH F D1 relative to B F D1	PH M D1 relative to B M D1	PH F D2 relative to B F D2	PH M D2 relative to B M D2	PH D1 relative to B D1	PH D2 relative to B D2
***N*-acyl proline**										
N-palmitoyl proline			↓				↑↑			
N-stearoyl proline	BAL	BAL	BAL	BAL	BAL	BAL	BAL	BAL	↑↑	
N-oleoyl proline		↓	↓		↑↑				↑	
N-linoleoyl proline										
N-arachidonoyl proline										
N-docosahexaenoyl proline										
N-acyl serine										
N-palmitoyl serine										
N-stearoyl serine										
N-oleoyl serine				↓↓↓↓			↑↑↑↑			
N-linoleoyl serine	BAL					BAL			↓↓↓↓	
N-arachidonoyl serine	BAL	BAL	BAL	BAL	BAL	BAL	BAL	BAL		
N-docosahexaenoyl serine	BAL	BAL		BAL	BAL	BAL	BAL			
N-acyl taurine										
N-palmitoyl taurine	↑↑↑↑					↑↑↑↑		↑↑		↑↑
N-stearoyl taurine						↑↑↑↑			↑↑↑	
N-oleoyl taurine						↑↑↑↑		↑↑↑		
N-arachidonoyl taurine										
N-acyl tryptophan										
N-palmitoyl tryptophan										
N-stearoyl tryptophan										
N-oleoyl tryptophan					↓		↓			↓↓
N-linoleoyl tryptophan	BAL	BAL	BAL		BAL	BAL	BAL			
N-arachidonoyl tryptophan	BAL	BAL	BAL	BAL	BAL	BAL	BAL	BAL	BAL	BAL
N-docosahexaenoyl tryptophan	BAL	BAL	BAL	BAL	BAL	BAL	BAL	BAL	BAL	BAL
N-acyl tyrosine										
N-palmitoyl tyrosine		↓↓↓				↑↑				
N-stearoyl tyrosine		↓↓								
N-oleoyl tyrosine										
N-linoleoyl tyrosine		↓↓↓↓								
N-arachidonoyl tyrosine										
N-docosahexaenoyl tyrosine		BAL	BAL		BAL		BAL			
N-acyl valine										
N-palmitoyl valine							↑↑↑			
N- stearoyl valine										
N-oleoyl valine						↑↑↑↑			↑↑↑	
N-linoleoyl valine						↑↑↑↑				
N-docosahexaenoyl valine										
**2-acyl-sn-glycerol**										
2-arachidonoyl-sn-glycerol							↓↓			↓↓
2-palmitoyl-sn-glycerol										
2-oleoyl-sn-glycerol	↑↑↑↑		↓↓	↓↓↓				↑↑		
2-linoleoyl-sn-glycerol	↑↑↑			↑↑↑	↑					
**Free Fatty Acids**										
Oleic acid	↑↑↑↑					↑↑↑↑	↑↑↑			↑↑↑
Linoleic acid	↑↑↑↑			↓↓↓↓		↑↑↑↑	↑↑↑			
Arachidonic acid	↑↑↑↑	↑↑↑	↑↑↑	↑↑		↑↑↑↑				
Eicosapentaenoic acid	↑↑↑↑					↑↑↑↑				↑↑↑
Docosahexaenoic acid	↑↑↑↑↑					↑↑↑↑↑				
**Prostaglandins**										
PGE_2	↑↑↑↑	↑↑↑↑								
$PGF_{2\alpha}$								↓		
6-keto-$PGF_{1\alpha}$	BAL	BAL	BAL	BAL	BAL	BAL	BAL	BAL	BAL	BAL
